# Real-Time Parallel Processing of Grammatical Structure in the Fronto-Striatal System: A Recurrent Network Simulation Study Using Reservoir Computing

**DOI:** 10.1371/journal.pone.0052946

**Published:** 2013-02-01

**Authors:** Xavier Hinaut, Peter Ford Dominey

**Affiliations:** 1 INSERM U846 Stem Cell and Brain Research Institute, Bron Cedex, France; 2 Université de Lyon, Université Lyon I, Lyon, France; Centre national de la recherche scientifique, France

## Abstract

Sentence processing takes place in real-time. Previous words in the sentence can influence the processing of the current word in the timescale of hundreds of milliseconds. Recent neurophysiological studies in humans suggest that the fronto-striatal system (frontal cortex, and striatum – the major input locus of the basal ganglia) plays a crucial role in this process. The current research provides a possible explanation of how certain aspects of this real-time processing can occur, based on the dynamics of recurrent cortical networks, and plasticity in the cortico-striatal system. We simulate prefrontal area BA47 as a recurrent network that receives on-line input about word categories during sentence processing, with plastic connections between cortex and striatum. We exploit the homology between the cortico-striatal system and reservoir computing, where recurrent frontal cortical networks are the reservoir, and plastic cortico-striatal synapses are the readout. The system is trained on sentence-meaning pairs, where meaning is coded as activation in the striatum corresponding to the roles that different nouns and verbs play in the sentences. The model learns an extended set of grammatical constructions, and demonstrates the ability to generalize to novel constructions. It demonstrates how early in the sentence, a parallel set of predictions are made concerning the meaning, which are then confirmed or updated as the processing of the input sentence proceeds. It demonstrates how on-line responses to words are influenced by previous words in the sentence, and by previous sentences in the discourse, providing new insight into the neurophysiology of the P600 ERP scalp response to grammatical complexity. This demonstrates that a recurrent neural network can decode grammatical structure from sentences in real-time in order to generate a predictive representation of the meaning of the sentences. This can provide insight into the underlying mechanisms of human cortico-striatal function in sentence processing.

## Introduction

One of the most remarkable aspects of language processing is the rapidity with which it takes place. This is revealed perhaps most clearly in event related brain potential (ERP) studies in which a word that violates predictions about the developing meaning or grammatical structure can yield brain responses to that word as rapidly as 200–600 ms [1]–[3]. Interestingly, it has been demonstrated that these effects can span multiple sentences. That is, information that is provided by a sentence earlier in the discourse can cause an on-line conflict response to a word that occurs several sentences later in the discourse [3]–[5]. This suggests that the brain is accumulating evidence on-line in real-time, and predicting or generating expectations about the subsequent structure of the incoming sentence. It also indicates that the brain is making that accumulated knowledge available in real-time, and that it is continuously revising its predictions based on the interaction between incoming information, and the context formed by earlier inputs.

Converging evidence indicates that it is not the cerebral cortex alone that is responsible for this processing, and that indeed the cortico-striatal system (made up of cortex and striatum, the cortical input nucleus of the basal ganglia) plays a significant role in language processing. Moro et al. [6] observed significant activation of left cortical area BA45 (part of Broca's area), and of the left caudate nucleus of the striatum under conditions that specifically required syntactic processing. Similarly, during the processing of syntactic anomalies, Friederici & Kotz [7] observed cortical activity in the left posterior frontal operculum adjacent to BA 44 (part of Broca's area) and in the putamen of the left basal ganglia. Likewise, studies of patients with dysfunction of the striatum provide support for the hypothesis that the cortico-striatal system plays a role in language processing. Hochstadt [8] examined performance in syntactic comprehension and cognitive set-switching, verbal working memory, and articulatory rehearsal in a population of 41 Parkinson patients and observed a syntactic comprehension deficit for complex sentences in these patients. This suggests that an intact basal ganglia is required for aspects of syntactic processing. In this context, Ullman has suggested, at the word level, that a form of procedural memory in the cortico-striatal system is responsible for the application of grammatical rules [9], [10].

Friederici and colleagues have taken a complimentary approach to investigating basal ganglia function in language processing through the analysis of brain activity in Parkinson's patients during syntactic processing [11]. These authors examined PD patients and age-matched controls in an auditory sentence comprehension task, and analysed ERP responses (recorded by scalp electrodes) related to early “first pass” syntactic processing (revealed by the early left anterior negativity or ELAN), and to the later syntactic integration (revealed by the P600). The P600 is a well-defined brain response to syntactic complexity and/or syntactic violations, around 600 ms after the violating word [2], [7], [12]–[14]. Interestingly, PD patients did not differ from controls with respect to the ELAN. However, their P600 response was significantly reduced compared to that of controls [11]. Similar studies in patients with lesions of the basal ganglia revealed that these subjects failed to produce a normal P600 in response to syntactic anomalies [12], [15]. These data argue that the intact striatum is required for generation of the P600 response. This raises the question of the role of striatum in the P600. It is likely that the P600 does not originate in the striatum, but rather in the cortex, possibly under the influence of the striatum via the cortico-striato-thalamo-cortical (CSTC) loop. Magneto-encephalogram (MEG) source localization studies have localized the origin of the P600 as bilateral cortical sources in the temporal lobe [16]. Temporal cortex has a strong anatomical connectivity with the striatum [17] which, via disinhibition in the striato-nigral circuit [18], can activate thalamic nuclei that project back to temporal cortex [19] in non-human primates. Via this CSTC loop, striatum influences temporal cortex. The functional correlate of this CSTC interaction has been demonstrated in humans using fMRI [20]. It is thus likely that the striatum contributes to the P600, as observed in human clinical studies [11], [12], [15], through striatal influence on cortex via the CSTC circuit. This would predict that activity within the striatum contributes to the generation of the cortical P600.

Interestingly, the P600 is not restricted to grammatically incorrect sentences, but has also been carefully studied during the processing of grammatically well formed sentences that have ambiguities that are resolved late in the sentence. Friederici et al. [2] thus observed P600 responses for words in well formed sentences that were critical for resolution of grammatical ambiguities. They suggested that their results were potentially consistent with parallel models of sentence parsing [21], [22], where multiple options of the parse are maintained in parallel. While there is accumulating evidence for the role of the cortico-striatal system in processing some aspects of the grammatical structure of language in real-time, the underlying mechanisms and their implementation in neural structures of the cortico-striatal system remains an important open research topic.

**Figure 1:**
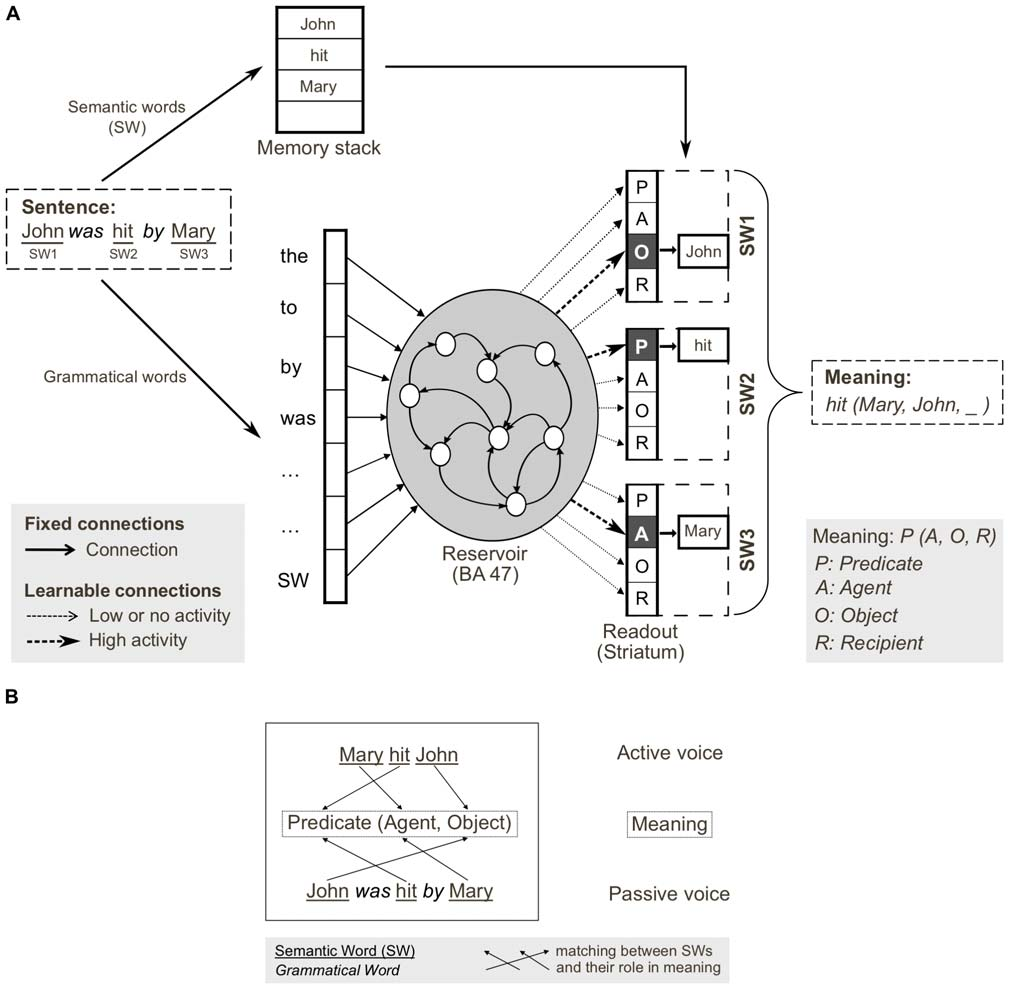
Thematic role assignment and correspondence between cortico-striatal and reservoir implementations of sentence processing. A. Grammatical construction processing in the reservoir framework. Semantic and grammatical words (i.e. open and closed class words, respectively) are separated on input. Semantic words (SW) are stored in a memory stack. Grammatical words and a single input for all SWs are inputs to the reservoir (analogous to prefrontal cortex area BA47). During training, input sentences are presented word-by-word, and readout units (corresponding to striatum) are forced to the corresponding coded meaning (i.e. SW1-Object, SW2-Predicate, SW3-Agent). In testing, readout units code the predicted role(s) of each semantic word, forming the coded meaning. The meaning (i.e. hit(Mary, John, _)) can be reconstructed from the coded meaning, as SWs in memory stack are reassigned to the thematic roles (predicate, agent, object, recipient) identified in the read-outs. B. Active and passive grammatical constructions (i.e. mapping from sentence form to meaning), and their shared meaning. Coded meaning (indicated by the arrows) corresponds to specific mapping from open class words to meaning, which defines the grammatical construction.

To begin to address this issue we previously developed neural network and more symbolic models of thematic role assignment in sentence processing [23]–[29]. Here, we extend this work in the framework of the recurrent neural network model illustrated in Figure 1. Thematic role assignment involves determining who did what to whom – or extracting the thematic roles (agent, object, recipient) for the verbs in a sentence. Thus for the sentence “The boy who took the ball from the man was bitten by the dog” *boy* is the agent of *took*, and the object of *bitten*.

Figure 1 (A) illustrates the functional organization of the model, and (B) the notion of thematic role assignment in grammatical constructions. A grammatical construction is the mapping between the surface form of a sentence (word order) and the meaning [30]. In the model sentences are presented word-by-word as input, and the model extracts the coded meaning, i.e. specification of the thematic role of each open class element in the sentence. This can then be used to specify the global meaning of the sentence. More specifically, while the two sentences “Mary hit John” and “John was hit by Mary” both have the same meaning *hit(Mary, John)*, their coded meanings are different, i.e. in the first sentence Semantic Word 1 (Mary) is the agent, while for the second sentence, Semantic Word 1 (John) is the object, and so on. This is represented in Figure 1B which illustrates grammatical constructions as mappings from sentence form onto meaning [30], [31], here for the active and passive constructions. Our models are based on the principle that the information necessary to perform this thematic role assignment is encoded in the sentence by the configuration of grammatical function words (e.g. determiners, auxiliary verbs, prepositions) within the sentence. This is based on the cue competition hypothesis of Bates & MacWhinney [32], which holds that across languages, a limited set of cues including the configuration of grammatical function words (closed class morphology in general), word order and prosody are used to encode the grammatical structure that allows thematic role assignment to take place. We thus implement the cue competition hypothesis [32], [33] focusing on word order and grammatical morphology. In our modeling, the notion is that the sequence of closed class words forms a pattern of activity within the recurrent network, and that this pattern can be associated with the corresponding thematic role specification. Figure 1A illustrates the modeling of this approach. Closed class information is input to a recurrent prefrontal network (the reservoir) corresponding to prefrontal cortical area BA47. Resulting patterns of activity in this recurrent network can be decoded in the caudate nucleus of the striatum (the readout), based on connections between cortex and striatum that are appropriately modified by learning. We thus propose that the cortico-striatal system plays a specific role in language processing, with recurrent connections in cortex encoding the ongoing grammatical structure, and the striatum decoding this structure as rules for mapping open class elements onto their appropriate thematic roles. This is consistent with Ullman's proposal that the cortico-striatal system implements a rule-based procedural system for the application of grammatical rules at the word level [9], [10].

In previous work, we demonstrated the feasibility of this concept in a recurrent network, with two significant limitations [24], [27]. The first limitation was that we used a restricted set of nine grammatical constructions as defined by the Caplan protocol [34] which had been designed for testing patients for syntactic comprehension deficits. The second restriction was that, following the Caplan protocol, the model was trained to identify the agent, object and recipient, in that order, *after* the complete presentation of the sentence. Thus, we had no insight into the online processing of the meaning of the sentence. Both of these limitations were in part related to a technical limitation at the time of the development of this model, related to the learning mechanism.

This technical limitation was manifest in the training. After each sentence presentation the model was trained by trial and error to produce the agent, object and recipient of the sentence. This required extensive training, and the incremental learning method we employed prohibited experiments with large corpora. In the current research we overcome this limitation, by employing the reservoir computing (RC) approach. Reservoir computing is a machine learning technique in which a recurrent network with fixed connections is used to encode the spatiotemporal structure of an input sequence, and connections to a readout layer are trained using efficient algorithms to produce a desired output in response to input sequences [35], [36]. The central concept of reservoir computing is to project low dimensional inputs onto a recurrently connected “reservoir” of neurons which creates a high-dimensional projection of the low-dimensional input space, thus increasing the input separability. The recurrent connections provide sensitivity to events over time, thus yielding the desired sensitivity to sequential and temporal organization. This projection and the dynamics of the reservoir serve as a form of kernel in the machine learning sense, thus allow one to perform complex non-linear computational tasks with simple linear readouts [37]. Interestingly, it was recently noted by Jaeger and his colleagues [38] that our earlier work that modeled prefrontal cortex as a recurrent network with fixed connections, and modifiable cortico-striatal connections to learn the desired output sequences [28], [39] was in fact the first expression of the reservoir principal, implemented in a proposed neurophysiological substrate (i.e. the cortico-striatal system). Exploiting this homology as illustrated in Figure 1B, we can now take advantage of the machine learning techniques that are standard in the RC domain, and thus exploit a significant speedup in the learning required for our experiments, and a more mature theoretical framework. In particular, we now exploit the use of regression techniques for learning the connection weights between reservoir and readout units [35], [38], [40]. The training corpus is first presented, sentence by sentence, word by word, to the reservoir, and the population activation state is recorded for each time step during presentation of the corpus. The reservoir state is reset before the presentation of each sentence. We also generate the desired activation pattern in the output neurons that code the meanings for each sentence. Linear regression is then used to learn the output weights that will produce the correct mapping between reservoir activity and desired readout response. We test two modes of learning. For continuous learning, the regression is applied starting at the onset of the first word on each time step: thus we ask the readout neurons to provide the coded meaning of the sentence from the onset of the sentence. For sentence final learning, the regression is applied only at the end of the sentence.

The aim of the current research is to test the hypothesis that a recurrent architecture as illustrated in Figure 1 can be used to learn a set of grammatical constructions, and to test the following derived predictions:

1. Real-time processing: Striatal (readout) activity should reflect a real-time estimation of the grammatical structure of the sentence. The final parse may be predicted before the end of the sentence, and this may change as new words invalidate the current parse. The changes in neural activity in the striatal readout may reflect language related ERPs recorded during human sentence processing.
2. Generalization: To a limited extent, the system should be capable of generalizing grammatical processing to new constructions if these new constructions adhere to the same grammatical structure that is present in the training corpus.
3. Prior knowledge in discourse. In the context of multiple sentences, the system should be capable of exploiting prior information (i.e. from an earlier sentence) in the interpretation of the ongoing sentence.
4. Scaling to larger more varied corpora. The system should display some scaling capabilities that reflect its ability to extract grammatical structure and generalize this to new grammatical structures.

## Results

### Experiment 1: Basic Syntactic Comprehension

**Figure 2:**
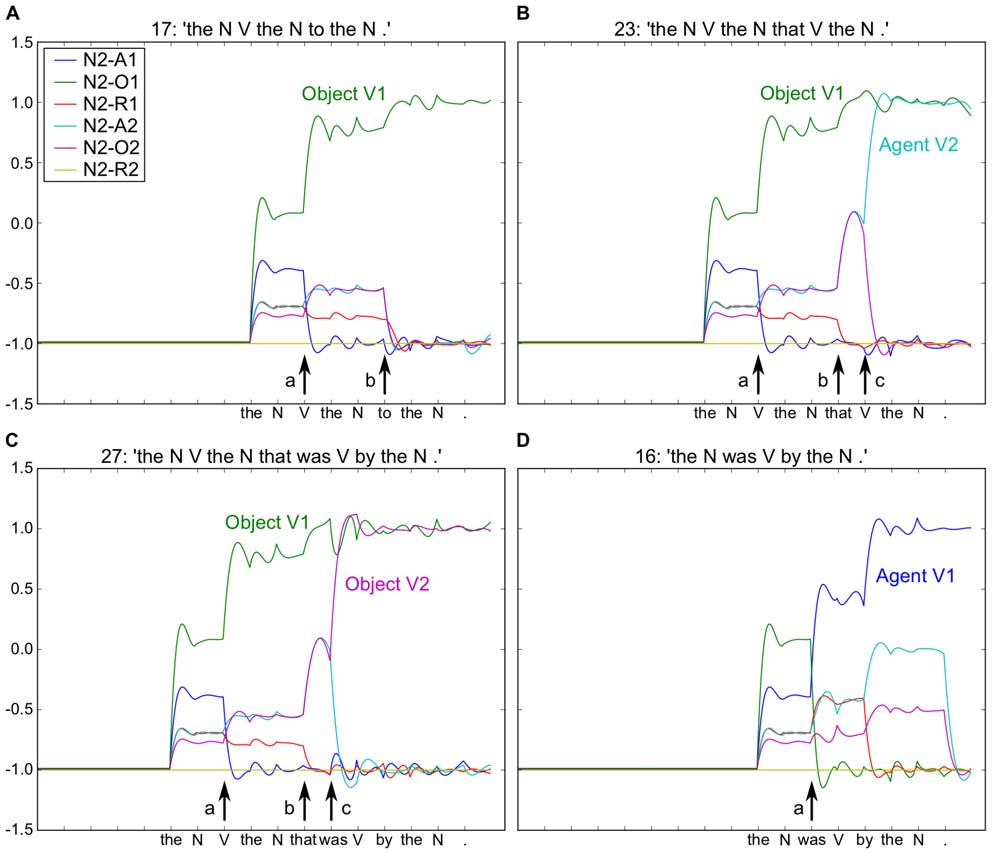
Output of the “striatal” readout neurons for Noun 2 for four example sentences. Neurons coding different thematic roles indicated by colored traces (see inserted legend). For all four sentences (see period before arrow (a)), the model initially predicts that Noun 2 is the Object of Action 1 (green trace). In B and C this remains true, but Noun 2 is also the Agent and Object of Action 2 in B and C respectively. At point (b), arrival of “to” confirms the correct prediction of N2-O1 (green trace) in A, and the arrival of “that” induces a change in activity in B and C, with increased prediction of both Agent and Object roles for V2, respectively. Note that this is resolved at the arrival of the “V” and “was” in B and C respectively (arrow (c)). In D the arrival of “was” provokes a new analysis with Noun 2 as the Agent of Action 1. Embedded legend: N2-A1 – Noun 2 is the agent of Action 1. A – Agent, O – Object, R – Recipient. Simulation conditions, activation time, AT  = 20, and number of reservoir units, N = 300.

Syntactic comprehension is the ability to determine who did what to whom based purely on syntactic or grammatical information in the sentence [34]. We first tested the syntactic comprehension ability of the model by determining whether it could learn 26 distinct grammatical constructions that were used in Dominey et al. (2006) (see Text S1, structures 15 to 40). These constructions demonstrate different surface forms (e.g. active, passive, dative-passive, subject-relative, etc.) including single verb, and double verb relative surface forms. Sentences are presented word-by-word as input to the model which has been trained to produce the coded meaning. Recall that the coded meaning is the specification for each ordered semantic word of its thematic role, for the first (and optionally) second action. We thus consider that this coded meaning, revealed by the readout neural activity, is the system's analysis of the meaning. From the outset of this processing the readout neurons encode the currently predicted meaning of the sentence. Figure 2 illustrates the behaviour of the model for 4 example sentences.

A. The N V the N to the N.

B. The N V the N that V the N.

C. The N V the N that was V by the N.

D. The N was V by the N.

The six neurons shown represent the possible thematic roles of the second noun (Noun 2) in these sentences (which are Agent, Object or Recipient for Action 1 or Action 2). Following the presentation in all four sentences of “The N”, the neural activity represents the expected probabilities for each of the possible thematic roles, and this activity is identical at this point for the four sentences. As successive words arrive, these probabilities are updated, illustrating the on-line reanalysis (i.e. change in coded meaning) of the sentences. More precisely, for sentences A–C, the prediction that the second noun (N2) is the object of the first verb is confirmed with the arrival of “V” (see arrow marked (a), and green trace in Fig. 2A, B, C). At this same point the arrival of “was” in D yields a dramatic shift in the neuronal activity, with N2 finally coded as the agent of V1 (see (a) and blue trace in Fig. 2D). The arrival of “to” in A, versus the “that” in B and C (see arrow (b)) illustrates that the predicted analysis was for the more common sentence form in A, with a shift in activity in B and C. For B and C the arrival of “that” indicates that a second verb exists, activating the agent and object roles for verb 2. The final decision on the role of noun 2 as Agent or Object of verb 2 is marked at arrow (c). The reanalysis is in fact a continuous analysis that can continue on the predicted trajectory, or in the reanalysis case, be perturbed into a new trajectory. It is noteworthy that this real-time shift in coding was not trained explicitly, but rather reflects the inherent properties of the reservoir and read-out neurons working together. The reservoir encodes the ongoing trajectory of grammatical structure as words successively arrive. The trained read-out neurons extract this structure in real-time, in a predictive manner. This readout activity reflects the current probabilities for each of multiple possible parses in parallel.

The model was able to learn this set of constructions without error. This result is thus consistent with the closed class hypothesis for these 26 distinct grammatical constructions, i.e. that the ordered set of closed class elements is sufficient to uniquely identify each distinct grammatical construction [23], [25], [31], [32]. Perhaps more interestingly, the results of this experiment indicate that the recurrent network encodes an incremental representation of the grammatical structure of the input sentences, which is then demonstrated in the readout neurons. This can be observed by the values of the readout neurons, which encode the meaning, and their on-line modifications over the course of the word-by-word presentation of the input sentences. As seen for example in 2D the meaning is initially incorrect, and then resolved at the arrival of *was*, which indicates the passive form. We examine this on-line reanalysis, and a possible neurophysiological correlate more closely in Experiment 2.

### Experiment 2: A Neural Coding Explanation of the P600

The meaning of a sentence may be ambiguous at some intermediate point, with the correct meaning becoming certain only later in the sentence. This can be observed in reading of sentences generated from “relative” constructions. “Relative” constructions contain two verbs – the main and the relative. ERP experiments [2] indicate that object-relative sentences, like (b) below are more difficult to process than subject-relative sentences like (a).

a. The dog that bit the cat chased the boy. (Subject-relative).

b. The dog that the cat bit chased the boy. (Object-relative).

This difficulty is revealed in part as a larger P600 event related brain potential (ERP) response to the differentiating word in the object-relative sentences. Thus, when exposed to sentences (a) and (b), subjects will typically display a larger P600 response to “the” in (b) than to “bit” in (a). Part of the explanation of the difficulty in processing lies in the fact that “dog” is the subject of “chased”, but the object of “bit” in (b), while it is the subject of both verbs in (a). The perspective shift from subject to object is presumed to require additional processing effort [41]. It is also the case that the object-relative sentences are less frequent in general [42], suggesting that individuals may have less experience with such sentences. Thus, with respect to ambiguity, at the arrival of “that” the initial noun phrase “the dog” can either be the subject of the upcoming verb (the more frequent case) or the object. This ambiguity is resolved with the next word.

**Figure 3:**
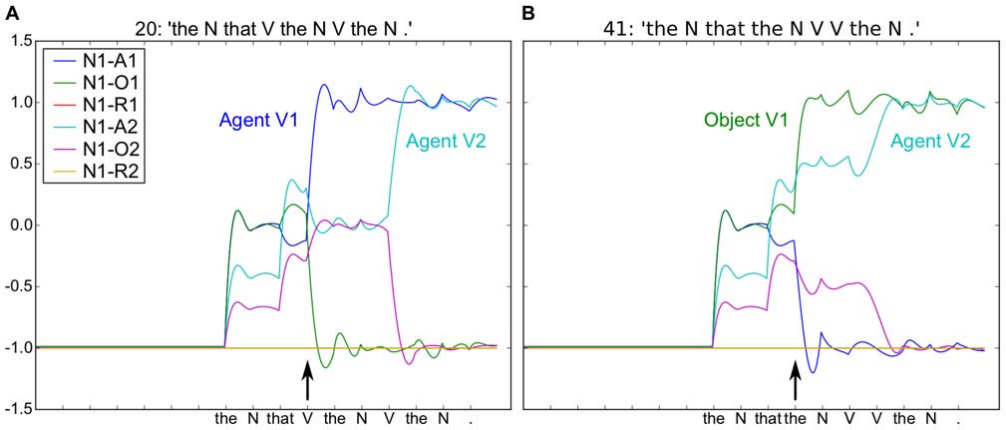
Processing relative phrases. Both sentences begin with “The N that...”. A. Subject-relative (first noun is subject of principal and relative clauses) “The N that **V** the N was V by the N”. Arrival of the “V” following “that” produces a shift in activity coding for N1-A1, i.e. Noun 1 is the Agent of Verb 1. At the arrival of the second “V”, we observe the increase in activity in N1-A2, coding N1 as the Agent of Verb 2. B. Object-relative (first noun is subject of principal and object of relative clause) “The N that the N V V the N.” Arrival of the second “the” generates a shift in the coded meaning with N1 assigned the role of Object of Action 1 (N1-O1), and subsequently Agent of Action 2 (N1-A2). It is of interest to compare the responses in to the second “V” in sentence 20 (Fig. 3A) to the responses at the same point to “was” in sentence 22 “The N that V the N was V by the N” in Text S2, where the two neurons coding Agent and Object of Verb 2 shift in the opposite sense. Simulation conditions, activation time, AT  = 20, and number of reservoir units, N = 300.

In this context, we want to investigate how the model's sensitivity to the statistical structure of the training corpus could help to account for observed P600 responses. One way of evaluating this sensitivity is to observe the level of activity in the read-out neurons that code the meaning. Observing Figures 2 and 3, we can see that the arrival of certain words causes changes in these levels of activity. These changes correspond to an updating of the predicted coded meaning. Comparing sentences A and B in Figure 3, if the object-relative is more difficult (or less frequent) we would expect to see more changes in the readout activity for sentence B when the critical word “the” or “V” arrives. That is, if the predicted meaning of a temporarily ambiguous sentence is resolved by a word indicating a low frequency grammatical construction, there will be more instantaneous change in neural activity, than if it is resolved by a word indicating a high-frequency construction. Interestingly, we observe the opposite. That is, the instantaneous change at the arrival of the “bit” (V) in (a) seems to be at least as great as the instantaneous change associated with the arrival of “the” in (b). Further investigation reveals that our corpus (constructions 15–44 in Text S1) has a large distribution of complex (non-canonical) sentence types, including the relative passive (e.g. sentence 24 in Text S1), in which the first noun is not the agent of the first verb. Thus, our corpus does not respect the standard distribution relative frequencies for subject- and object-relatives [42].

**Figure 4:**
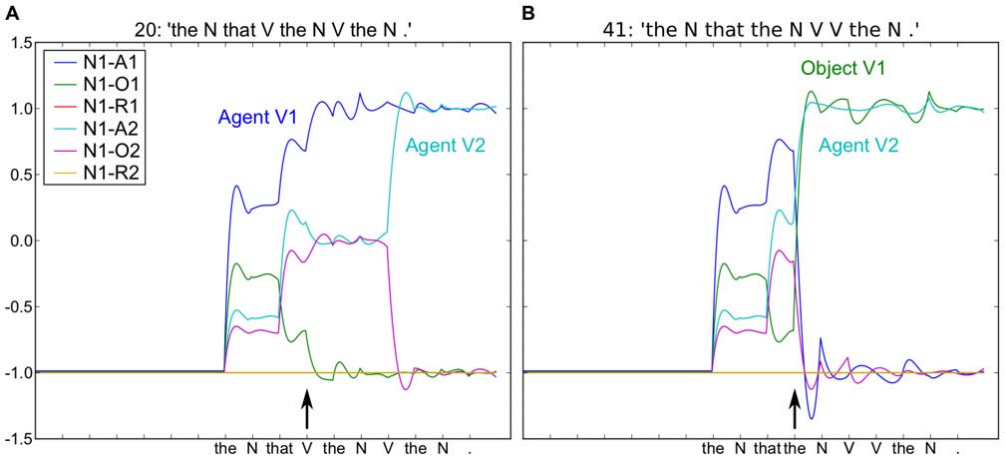
Relative sentences with modified corpus distribution such that subject-relatives are more frequent that object-relatives. A. Subject-relative. For the word “V” following “that”, there is relatively small change in the readout neurons, indicating that the predictions of the model were essentially confirmed. B. Object-relative. For the “the” following “that”, there is a significant shift in activity, corresponding to a re-assignment of the most probable coded meaning. Simulation conditions, activation time, AT  = 20, and number of reservoir units, N = 300.

In order to address this issue, we modified our corpus such that the frequency of the subject-relatives is greater than that of the object-relatives. In these conditions one would expect that sentences (such as the object-relative) which employ a less frequent (non-canonical) form would produce changes in the readout activity in response to the word that indicates the non-canonical form. In order to determine if these modified corpus statistics would influence the changes in readout activity in this way, we trained the model on this modified corpus where all the relative passives and all but one object-relatives are eliminated. The eliminated sentences are marked with * in the corpus in the Text S1. The results are illustrated in Figure 4.

With this updated distribution of subject- and object-relatives that more closely matches that found in human language [42], we now find the two expected effects. First, from the outset, N1 is predicted to be the agent of V1 (see the blue trace in Figure 4, vs. the overlapping blue and green traces in Fig. 3). Second, in the object-relative, the arrival of “the” after “that” produces a change in the predicted values, deviating from the initial prediction. For the subject-relative, the changes are much smaller with the arrival of “V”. We can consider that this relative difference in the changes of activity in the two conditions may have some functional relation to the P600 ERP that occurs in similar circumstances. This change in neural activity can thus be considered to reflect the degree of reanalysis, and a form of neuronal processing cost (from an energy perspective).

**Figure 5:**
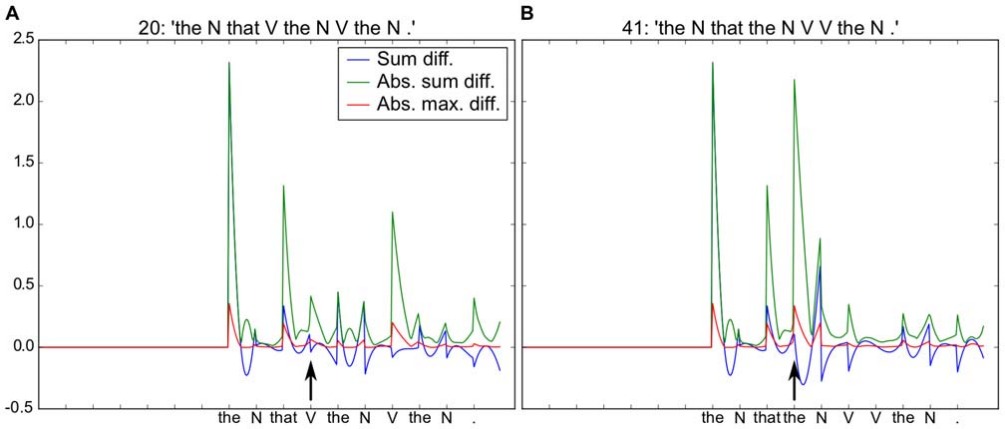
Global instantaneous changes in output activity. Green trace: Calculated sum of change (instantaneous temporal derivative) in neural activity over two successive time steps for Subject- and Object-relative sentences in A and B respectively. Corresponds to neural “effort” serving as an index related to human ERPs. This activity change can be compared with the human P600 response. For A and B sentence onset generates a significant change in activity. Arrival of relativizer “that” generates another significant change (indicating that this is a relatively low probability event in the corpus). The crucial comparison is for the next word (“V” and “the” respectively in A and B, marked with arrow). Arrival of “V” in A indicates with the subject-relative structure that is of higher probability in the corpus, and leads to small activity change. Arrival of “the” in B indicates the low frequency (only one in the corpus) object-relative, and generates a greater activity change. Blue trace – Sum diff. – simple sum of activity differences between two time steps. Green trace – Abs. sum diff. – sum of absolute values of activity differences. Red trace – Abs. max. diff. – absolute value of the maximum activity differences between two time steps. Blue and red traces are provided as additional information. Simulation conditions, activation time, AT  = 20, and number of reservoir units, N = 300.

In order to generate an analog to a “processing cost” signal, we can take a form of the time derivative of this neural activity in the readout neurons. We calculate this as follows: for each readout neuron, we measure the absolute value of the change in activity between two time steps, and take the sum of these changes. This can be visualized in Figure 5, where we calculate and display this change in neural activity. We focus on the arrival of the word following “The N that”, marked with an arrow in Fig. 5 A and B. In the right panel B, corresponding to the less frequent object-relative, there is a greater change in activity, analogous to the observed increased P600 effect in these conditions.

The system thus can be seen to generate on-line predictions about the evolving meaning of the sentence, such that changes in these predictions exhibit properties comparable to the P600. In particular, the changes in neural activity reflect corpus frequency, which is correlated (inversely) with processing difficulty and P600 amplitude [2], [42]. While this will be of potential interest in understanding the neurophysiology of the P600, it also implies that the system is extracting some of the underlying structure of the corpus.

### Experiment 3: Grammatical Generalization

A crucial notion in language learning is that extraction of the inherent grammatical structure from the training corpus during learning will allow the system to accommodate novel constructions that were not explicitly present in the training set. This notion is present both in computational, e.g. [43]–[45] and developmental [46], [47] contexts.

**Figure 6:**
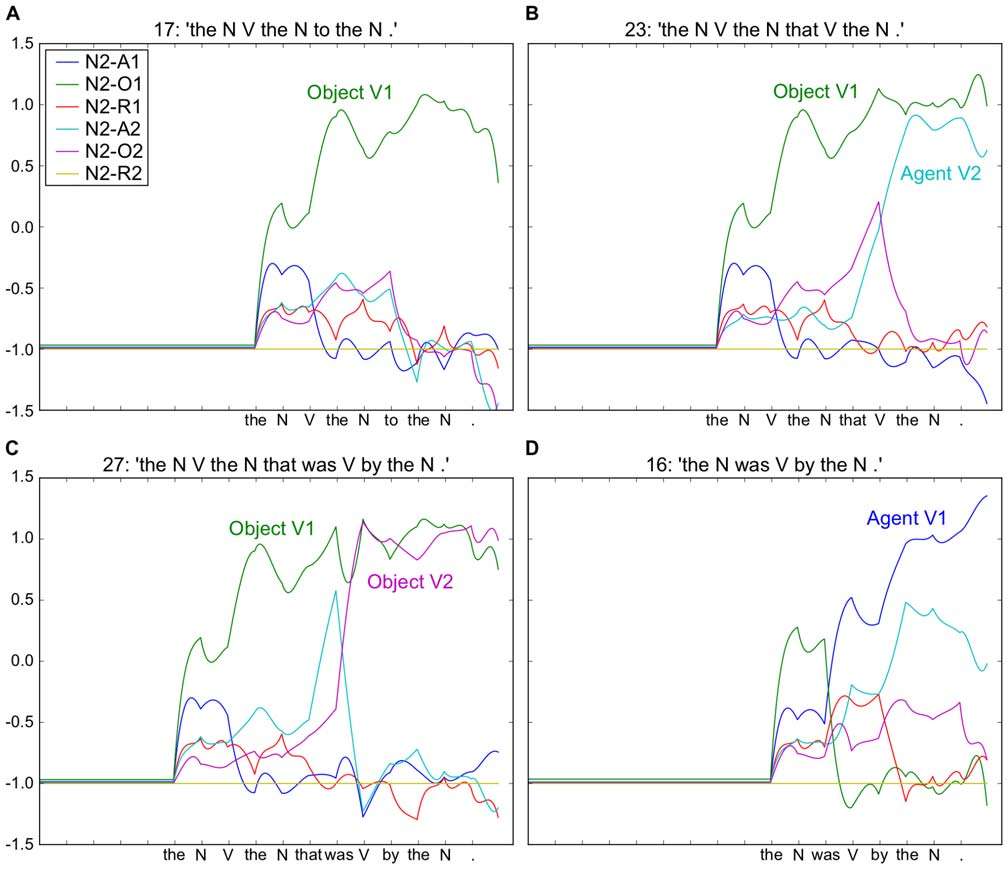
Generalization to new grammatical constructions that were not present in the training set. Same sentences as illustrated in Figure 2. In all cases, the model correctly determines the appropriate thematic roles for Noun 2 (and Noun 1 – not illustrated). Simulation conditions, activation time, AT  = 20, and number of reservoir units, N = 100.

In order to determine whether the learning performance on a fixed set of constructions, as observed above, can transfer to novel constructions, we performed a cross validation with the leaving-one-out method, where for each of the 26 constructions, the model was trained on the remaining 25 and then tested on the untrained construction. A reservoir of 100 units and activation time equal to 1 generalized to new test constructions (averaged over 100 reservoir instances) with 14.83% (±2.59) meaning error (i.e. % of all output neurons incorrect) and 42.73% (±7.19) sentence error (i.e. % of sentences with at least one output neuron incorrect – see methods – Training & Error Measures). This means that in the majority of the cases, the system is able correctly generalize to novel constructions. We verified performance with an activation time of 10 time steps, with 16.9% (+2.59) and 45.9% (+7.74) meaning and sentence error respectively, and an activation time of 20 time steps as well, with 17.2% (+2.63) and 46.4% (+7.74) meaning and sentence error respectively. This demonstrates the relative stability with respect to this parameter. Figure 6 illustrates successful generalization in these conditions (N = 100, AT  = 20) to the same constructions illustrated in Figure 2. While promising, this remains a form of “toy” demonstration, where the training set contains limited material and may constrain the ability to generalize. Thus, we will explore generalization with more extended corpora. First, however, we will consider the integration of information over the course of multiple sentences in short discourse segments.

### Experiment 4: Short discourse processing

**Figure 7:**
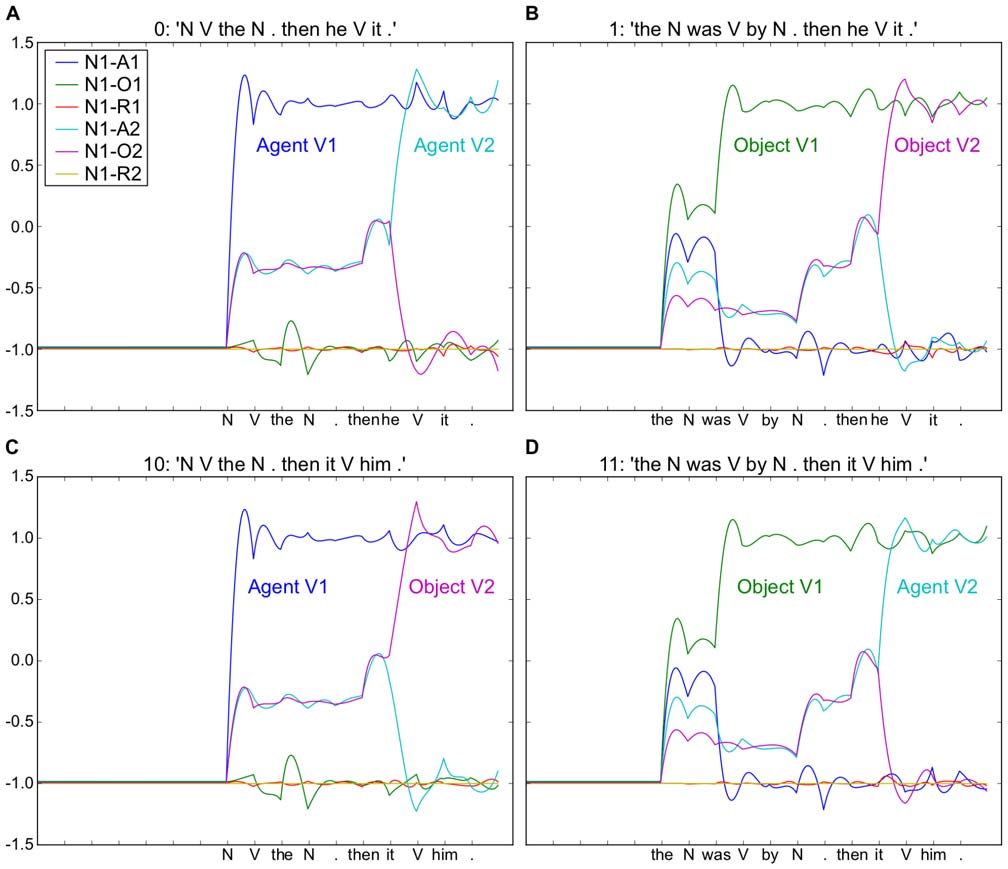
Two-sentence discourse processing. Four example discourses. A–C and B–D use the same first sentence form respectively, and A–B and C–D use the same second sentence form, respectively. This yields four distinct coded meaning patterns. For each, in the second sentence, the anaphoric reference of “he” and “it” must be resolved, such that “he” is associated with the proper noun, and “it” with the common noun (i.e. the one followed by “the”) from the first sentence. A. Noun 1 is the Agent of Action 1 and Action 2. B. Noun 1 is the Object of Action 1 and 2 C. Agent 1 Object 2 D. Object 1 Agent 2. Note that when comparing “then he hit it” in A and B, the coded meaning is different, dependant on the preceding sentence in the discourse. Similar for “then it hit him” in C and D. Simulation conditions, activation time, AT  = 20, and number of reservoir units, N = 300.

Information from sentences early in a discourse can influence subsequent processing in real-time [3]–[5]. For the prototypical example, a semantic anomaly N400 response [48] can be elicited to the word “salted” in the sentence “the peanut was salted,” if information earlier in the discourse has led to an expectation that the peanut is, in fact, in love [4]. This raises the question: how can information from a previous sentence be made available to modify in real-time the processing of the current sentence? To begin to respond to this question, the current experiment examines the ability of the model to use information from a previous sentence in order to determine the thematic roles of the second sentence in a two-sentence discourse segment. We trained and then tested the model on a total of 10 distinct two-sentence discourse segments (without cross validation). In all cases the second sentence employed pronouns “he” and “it” that required correct reference to the proper and common nouns, respectively, in the preceding sentence. The model was able to correctly exploit the information in the ten two-sentence discourse segments studied. Figure 7 illustrates the striatal readout responses for neurons coding the meaning of Noun 1 for 4 example discourses. This is an interesting set of discourse pairs that crosses two different first sentences with two different second sentences. Note that for all illustrative sentences, we include specific nouns and verbs for illustration, but the model is always processing input sequences where nouns and verbs are simply coded as N and V (or SW for Experiments 5–7).

A. John threw the boomerang. Then he caught it.

B. The boomerang was thrown by John. Then he caught it.

C. John threw the boomerang. Then it hit him.

D. The boomerang was thrown by John. Then it hit him.

That is, A and C both start with one sentence, B and D with another; then A and B have the same second sentence, with C and D sharing another. This allows a systematic comparison of the effects of first and second sentence on the neural encoding of meaning. Note that A and B have the same overall meaning, and that the way that meaning is coded in the output neurons, the coded meaning (see Figure 7), is different for these two discourse segments. We observed that in all ten discourse segments, the system correctly extracted the thematic roles specified in the two sentences. This includes extracting different meanings for the same sentence, depending on the two distinct sentences that arrived earlier in the two discourse segments. Interestingly, we can also detect that the processing of the ambiguous “he” in A and B is resolved in real-time even though it depends on information that occurred in the previous sentence. That is, there is no additional processing time associated with retrieval of information that occurred earlier in the two-sentence discourse.

These discourse processing results illustrate that the recurrent network can accumulate structural information from multiple sentences. In this case, that information was used to resolve the meaning of anaphoric references for sentences like “Then he threw it” differently, depending on the context specified in the earlier sentence. These results demonstrate how information accumulated over successive sentences can be made available on-line, providing insight into the discourse processing results of van Berkum and Hagoort.

### Experiment 5: Extended Corpus I

The previous experiments provide insight into real-time sentence processing in the model, but with a rather limited data set. The question immediately arises as to whether the system can accommodate a more extended set of constructions. Here we attempt to determine the performance with an extended corpus. Using a context free grammar we systematically generated a set of 462 novel grammatical constructions (see Methods), each expressing meanings consisting of between 1 and 6 nouns, with 1 to 2 levels of hierarchical structure (i.e. with only a main clause, or a main and relative clause, and 1 or 2 verbs, respectively). Each grammatical construction is a mapping between a unique surface form and a unique coded meaning. An exact mapping between inputs and outputs is thus possible. Using a model with 1000 neurons, the system learned the full “462 corpus” set perfectly (with no error when using the full corpus both as training and testing set) for *sentence final learning* (i.e. training only at the end of the input sentence, vs. continuous learning over the whole sentence – see Learning conditions and Error Measures in Materials and Methods). Even before considering generalization, this is already a significant result. It demonstrates that the corpus indeed adheres to the cue competition hypothesis [33], that the corpus is learnable, and that the system can exploit this learnability.

In order to determine the capacity of the system to generalize learned grammatical structure to new, untrained constructions, we then examined the behaviour in a ten-fold cross-validation. Ten per cent of the corpus (∼46 constructions) was removed from the training set, the model was trained on the remaining 90%, and then tested with the 10% not used in training. This was performed over 10 partitions so that all constructions were tested in cross validation. This procedure was averaged over 10 reservoir instances.

**Figure 8:**
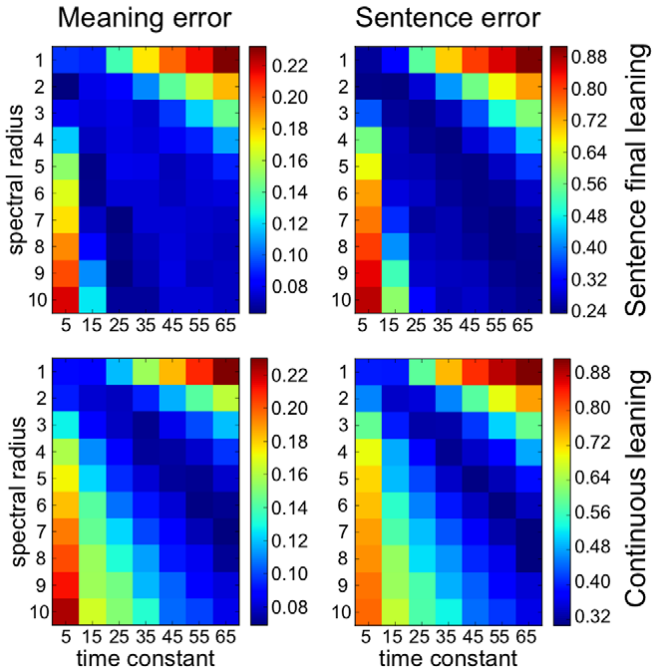
Parameter exploration (sensitivity analysis) for cross-validation performance with 462 grammatical constructions. Effects of spectral radius and time constant (τ) on performance. The error was averaged over 10 different reservoir instances. Colored cells indicate the mean error for those parameter setting. Note that in all four studies, there is a region along the diagonal where robust performance is observed. Number of units in the reservoir: N = 1000.

Figure 8 illustrates performance results in cross validation while we systematically explored the behavior of the system over a range of values for the spectral radius (SR) and time constant (τ) for a reservoir size of 1000 neurons. Together these parameters influence the relative degree of responsiveness of the system to inputs and to recurrent activity within the network. The spectral radius can be compared to the temperature of the dynamical system, and τ to the inertia or viscosity of the system (see Eqn. 1, Materials and Methods). Alternatively, the spectral radius is analogous to the gain in a feedback loop. More technically, the spectral radius is the largest absolute value of the eigenvalues of the reservoir weight matrix. For discrete linear time-invariant systems, a spectral radius smaller than one guarantees asymptotic stability, which means that the dynamics caused by an input pulse eventually die out [37]. As we are using leaky integrator (non-linear) neurons, the effective spectral radius may different from the actual value of SR. These and related issues are covered in [40], [49].

Figure 8 illustrates that the system is relatively stable to significant variation in these parameters, SR and τ, as there are large portions of the parameter space, along the top-left to bottom-right diagonal, where the system functions well, with graceful degradation of performance when deviating from that diagonal. During this exploration we identified parameters that yielded 9.2% meaning error and 24.4% sentence error in sentence final learning (SR = 1, τ = 6), and 7.4% and 32.1% for meaning and sentence error respectively for continuous learning (SR = 6, τ = 55) using cross validation.

**Figure 9:**
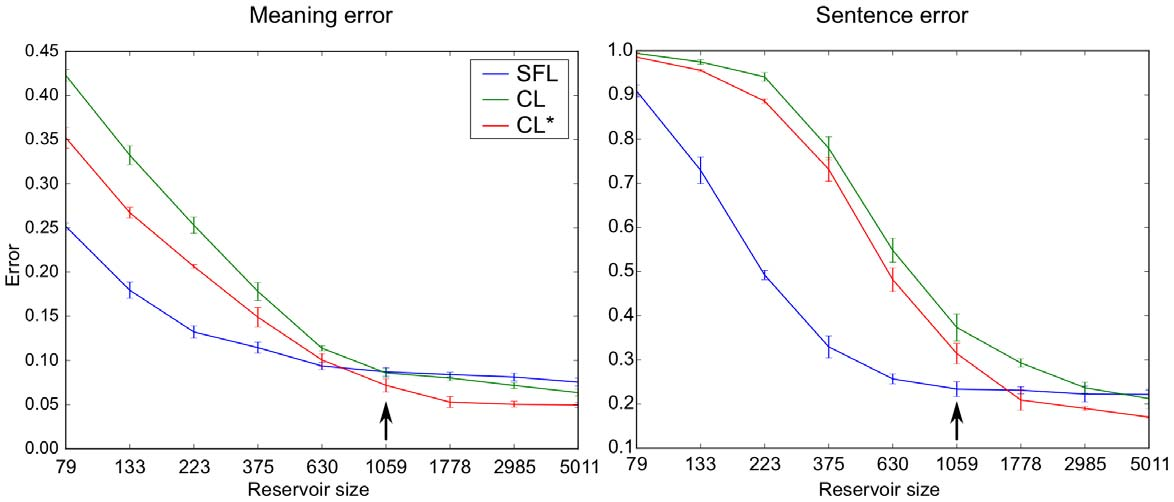
Effects of reservoir size on cross-validation with the 462 corpus. Meaning and Sentence Error (y-axis) as a function of the number of neurons (x-axis). Logarithmic scale. Each point is an average over 5 instances, with means and standard deviations shown. Cross-validation error reduces with reservoir size. This indicates that within the range of reservoir sizes studied, overfitting does not increase with reservoir size. Arrow marks reservoir size of N = 1059, for comparison with N = 1000 results in Table 1 and Experiment 5. CL – continuous learning (SR = 1, τ = 6), CL* optimized continuous learning (SR = 6, τ = 55), SFL – sentence final learning (SR = 1, τ = 6).

Another parameter that is essential in the performance of such systems in the number of neurons in the recurrent network, or reservoir. In order to determine how the performance is influenced by the number of neurons, we plotted the test error against the number of internal units. This can be seen in Figure 9.

Note the improvement seen beyond the 1000 neuron case, particularly for the continuous learning case. The asymptotic errors suggest a limit in the generalization possibility for this corpus. Note also that there is a continuous relation between reservoir size and performance, with progressive improvement as the size of the network increases. This is interesting in part because it potentially contradicts the notion that as the system size increases, so does the danger of overfitting and ensuing failure to generalize in the cross-validation [50], since we see continuous improvement with reservoir size. We address this issue in Experiment 6.

### Experiment 6: Effects of Corpus Structure

Because the corpus of 462 constructions contains inherent grammatical structure, based on the context free grammar used to create it, we believe that the ability to generalize demonstrated above in cross validation is due to some degree of learning of that underlying structure. To demonstrate that the system is generalizing on the grammatical structure of the training set (and not simply exploiting some arbitrary regularity in the data), we should submit the model to a test with a corpus that has the same constituent elements, but in the absence of this grammatical structure.

Such an experiment can also provide insight into what the system is actually learning – whether it is indeed learning some inherent structure, or in contrast whether it is in fact “overfitting” or memorizing the training set. Overfitting [50] can occur when the number of network parameters exceeds the size of the data set to be learned. The training set is thus memorized, and generalization to new items outside the training set will be poor. We are using 1000 reservoir units and 42 output units (see “Input and Output Coding” in Methods section), for a total of 42,000 trainable parameters. For the sentence final learning condition, in which reservoir to readout connections should be learned once at the end of the sentence, our corpus of 462 constructions corresponds to a data set size of 462 elements. For continuous learning, in which reservoir to readout connections should be learned during the presentation of each word in the sentence, this yields an upper limit on the data set size of 462 (number of constructions) ×19 (maximum sentence length)  = 8778 elements. This is significantly less than the size of the trainable parameter set. This is thus a situation that would be typical of overfitting. Again, to demonstrate that with this reservoir size the system is generalizing on the grammatical structure of the training set (and not overfitting the data), we submit the model to a test with a comparable corpus, but in the absence of this grammatical structure. We can then compare generalization in the presence and absence of inherent grammatical structure. If the observed effects of generalization are due to overfitting, then generalization as evaluated by cross-validation should not significantly vary in these two conditions. However, if grammatical structure is being learned, and generalized in the cross validation, then we should see better generalization when the training is based on a corpus that contains inherent grammatical structure. For the current Experiment 6, we thus scrambled the order of the words in surface forms of each of the 462 grammatical constructions, leaving all other conditions equivalent, and repeated Experiment 5 in the condition with 1000 neurons.

As illustrated in Table 1, we observe that while the scrambled set can be learned with no error, cross validation yields 73.4% *meaning* error and 99.9% *sentence* error rates for sentence final learning, and error rates of 70.6% and 99.9% for continuous learning, respectively. This success in learning, and failure in cross validation demonstrates generalization cannot be realized in the absence of underlying grammatical structure, which was eliminated in the scrambled data.

**Table 1:** Mean and standard deviation error in different learning conditions for train and test sets.

		462 corpus		462*6 corpus		462 Scrambled	
		m err	s err	m err	s err	m err	s err
SFL test	mean	9.178	24.370	8.154	24.953	73.391	99.913
	std	0.574	1.192	0.395	1.636	0.962	0.106
SFL train	mean	0.000	0.000	0.000	0.000	0.000	0.000
	std	0.000	0.000	0.000	0.000	0.000	0.000
CL test	mean	8.821	38.630	11.312	56.435	70.609	99.891
	std	0.353	1.029	0.214	1.403	0.747	0.146
CL train	mean	0.269	2.212	2.524	18.685	11.882	57.099
	std	0.050	0.421	0.084	0.606	0.604	2.015
CL test *	mean	7.433	32.130	9.490	48.351	74.154	99.891
	std	0.524	1.353	0.513	1.841	0.802	0.146
CL train *	mean	0.123	1.207	1.091	9.614	4.813	20.433
	std	0.029	0.297	0.083	0.730	0.299	1.251

Errors are given in percent. Different learning conditions: SFL – sentence final learning (SR = 1, τ = 6), CL – continuous learning (SR = 1, τ = 6), CL* optimized continuous learning (SR = 6, τ = 55). *m err* and *s err* are for *meaning* and *sentence* error respectively. std: standard deviation. Simulations were done with N = 1000 internal units.

We further examined the generalization in the grammatical (non-scrambled) corpus by adding words (adjectives and adverbs) that modify the sentences without changing the inherent structure, and observed the same significant level of generalization performance (see Table 1). Note that this modifies the overall timing of the inputs. The successful generalization thus demonstrates the relative robustness of the system to this time distortion.

### Experiment 7: Extended Corpus II

To pursue our investigation of the scaling capability of the model, we developed and extended the grammar to allow for 2043 distinct coded meanings, and then generated multiple different manners of expressing these meanings to generate a corpus of over 9*10^4^ constructions (corpus size  = 90,582 constructions) (see Corpora in Materials and Methods).

**Figure 10:**
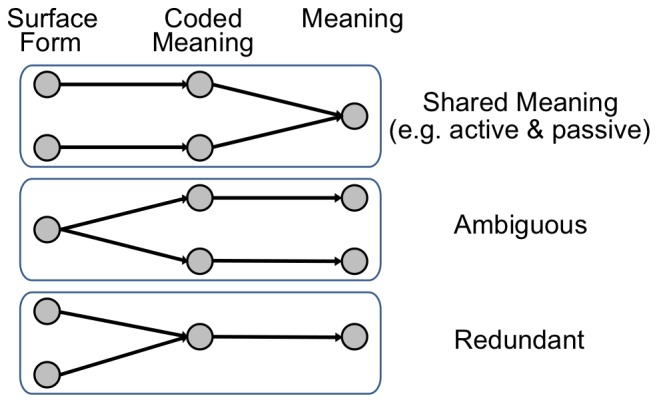
Different forms of meaning relations. Active and passive surface forms “John gave the ball to Mary” and “The ball was given to Mary by John” have different surface forms. They also have different coded meanings, e.g. the first semantic word (SW1) is the agent in the active, and the object in the passive. But these two coded meanings correspond to the same meaning *Gave(John, ball, Mary)* in the format *Predicate(Agent, Object, Recipient)*. Ambiguous sentences have the same surface form, but correspond to two different coded meanings and two different meanings. Finally, redundant sentences have different surface forms, but the same coded meaning and meaning.

This corpus has several properties that influence learning and generalization: (1) Multiple constructions can have the same coded meaning, thus the corpus can be considered to be “redundant”. For example, “The dog *by* whom Oliver is bitten ran” and “The dog whom Oliver is bitten *by* ran” are distinct surface forms but correspond to the same coded meaning. Redundancy is also present when substituting proper vs. common nouns (John/the man), and substituting relative pronouns (that/who). (2) Incomplete meanings were allowed (e.g. absence of an agent in the sentence: “The ball was given to Roger”). (3) Addition of reduced relative forms for relative clauses when possible (e.g. in “The ball that was bitten by the dog was thrown by Adam to Roger,” the “that was” can be removed to obtain a reduced relative). This also contributes to redundancy (i.e. multiple distinct constructions with the same coded meaning). (4) Constructions can be ambiguous. Twelve per cent of the constructions in the corpus are ambiguous. We define ambiguity as the case where two or three constructions have the same word order (surface form) corresponding to different meanings. For example, there are two constructions that correspond to a sentence “John who is introduced to the King by the Queen to England is ousted.” In these two constructions, the “Queen” can be the agent of “introduced” or of “ousted,” respectively. Figure 10 illustrates the distinctions between different such meaning relations involving the surface form, coded meaning, and meaning.

**Figure 11:**
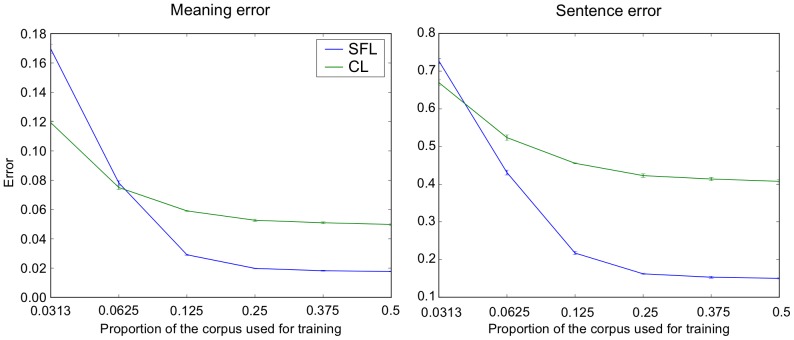
Effects of progressive exposure to 90K corpus. Meaning and Sentence Error for generalization to new construction (y-axis) as a function of the percentage of the corpus used for training. Generalization performance increases with percentage of corpus exposure in training. With exposure to only 12.5% of the corpus, the system generalizes with meaning error <4% and sentence error <22% for sentence final learning. Exposure to 50% of the corpus allowed generalization with 1.78% meaning error and 15.01% sentence error. Note that 12% of the sentences are ambiguous and will compete, rendering the real possible minimal sentence error ∼12%. Each point is an average over 5 instances, with means and standard deviations shown.

Because this corpus has internal grammatical structure and redundancy (i.e. multiple constructions have similar surface form and the same coded meaning), we expect that the ability to generalize will be greater than in the 462 construction corpus, despite the 12% ambiguous constructions which impair learning and generalization. In order to characterize the ability of the system to generalize based on exposure to different sample sizes, we randomly chose sub-corpora made up of 6%, 12%, 25%, 50%, 75% and 100% of the 90,582 element corpus. For each of these sub-corpora, we trained on half the sentences, and then tested on the remaining half, and then performed the reversal over these training and testing sets. Figure 11 illustrates generalization performance as a function of the training set size. Interestingly, we see that already when the sub-corpus is 25% of the total, and thus the training set is 12.5% of the corpus, the system can already generalize with only 22% sentence error and 4% meaning error for sentence final learning.

## Discussion

The stated goal of this research was to test the hypothesis that a recurrent network can encode the structure of sentences based on their closed class structure [33] and then (a) use this information to perform thematic role assignment, (b) generate predictions about the real-time effects [2], [12], (c) generalize to new constructions, and (d) encode prior knowledge from earlier sentences in a multiple sentence discourse [4], [5]. We address these questions in the computational framework of reservoir computing. Reservoir computing is a machine learning technique in which a recurrent network with fixed connections can encode the spatiotemporal structure of an input sequence, and connections to a readout layer can be trained using fast and powerful methods to produce a desired output [38]. This framework can be considered to define a form of equivalence class of problems that can be solved within the reservoir framework. The current results indicate that the task of thematic role assignment as we have specified it is a member of this equivalence class. This work was motivated in part by the postulate that the cortico-striatal system is perhaps at the heart of this processing in the human brain, with cortex and its inherent recurrent connectivity structure [51] playing a role analogous to the reservoir, and the striatum, with modifiable cortico-striatal connections [52]–[54] playing the role of the readout neurons. By extension, we can thus consider that the cortico-striatal system is functionally related to this equivalence class.

### Real-time processing

Part of the novelty of the current research is that the real-time processing of sentences by a recurrent neural network provides insight into the analogous real-time processing during human sentence processing as revealed by ERPs. Friederici et al. [2] observe ERP patterns indicating that human subjects demonstrate an on-line preference for subject-vs. object-relative sentences. Their P600 profile is of significantly reduced amplitude in the “simpler” subject-relative sentences. Observation of the read-out activity for words following the relativizer “that” in Figure 4 and 5 reveals a significantly greater modification of activity for the object-vs. subject-relative condition. Interestingly, this effect is reversed in Figure 3. As noted, this is due to a frequency effect in our training corpus. When trained with a corpus in which subject-relatives occur more often than object-relatives, as in the case in human language [42], we observe the corresponding P600-like effect. This provides a tentative simulation-based explanation of a P600 response. The readout neurons encode an online analysis or prediction of the meaning of the current sentence. When words arrive that cause a significant change in that predicted meaning, there is a significant change in the readout neural activity. This change can be codified in a temporal derivative, and the value of this temporal derivative can be compared with the P600. Clearly there are limits to this analogy. For example, the neural changes that we observe take place in the analog of the striatum (i.e. the readout layer), and they contain a mixture of increases and decreases in activation. In contrast, data from human striatal lesion studies [11], [12], [15] suggest that the P600 could be in part due to striatal activation that would produce synchronized activation of cortex through the cortico-striato-thalamo-cortical loop. Where the analogy does hold, however, is that words indicating low frequency constructions can evoke a P600 response in humans, and the same stimuli evoke a significant change in the neural activity in the model. Thus, this research should be of interest to the psycholinguistics community, as it provides a new tool for interpreting on-line electrophysiological responses during sentence processing. In particular, it demonstrates a system in which the activity of the read-out neurons represents the instantaneous probability estimation of the meaning of the sentence. Interestingly, this meaning is subject to change as successive words arrive.

### Neurophysiology of the P600

In this context, the current research contributes to the discussion concerning the nature and origin of electrophysiological responses to anomalous events during language processing. Recent studies have addressed ERP responses to non-standard perceptual events, both in the more general framework of the mismatch negativity (MMN) [55], and the language-specific P600 [56]. Wacongne et al. [55] implement a model that learns to predict a target signal, and that generates an MMN-like response to inputs that violate the predicted signal. This corresponds to a prediction error that is generated by comparing the predicted and actual input signals, consistent with the predictive coding framework proposed by Friston [57] in which cortical responses reflect the difference between internal predictions and the actual external input. The resulting prediction error is employed in a Bayesian model in order to update the predictive model in order to reduce the prediction error [55].

In the current work, the learning between the recurrent network (reservoir) and the readout neurons allows the system to predict the meaning of the sentence as its constituent words successively arrive. Our P600-like response (derived from the change in the readout prediction) can be considered as related to a prediction error, as it indicates the input-driven updates to the continuous prediction of the meaning. In both cases of Wacongne et al. and the current work, a memory trace (recurrent network or set of delay lines) maintains an ongoing representation of the task input context that is used to generate the on-line predictions.

Beim Graben et al. [56] take a related but different dynamical systems approach to explaining the P600. Based on sentences used in a P600 task, they generate a context free grammar and deterministic recognizers to parse these sentences, and implement these formal automata in nonlinear dynamic systems. Model ERPs are then obtained from principal components in the activation space, or by a measure of parsing entropy, of the resulting dynamical systems [56]. These measures are related to the notion of prediction error in that they represent a deviation from the input based on the current context. The fundamental difference with our work is that the dynamical system employed by beim Graben et al. is pre-structured based on a prior specification of the grammar, whereas our system is pre-structured only with the inherent properties of the recurrent reservoir network, and the open-closed class distinction. Interestingly, with no grammatical prior knowledge, this recurrent network can represent the grammatical structure of the corpus. Modifiable output connections then adapt to that structure in extracting the meaning, similar to [44], [58].

Frisch et al. [59] further explored the functional significance of the P600. In German they confirmed that when an argument disambiguates an earlier argument towards the non-preferred subject-object order a P600 is observed, reflecting cost associated with this revision. Strikingly, they also observed for the first time a P600 associated with a sentence-initial ambiguous argument. Since there is no reanalysis at work, these authors argue that the P600 must be considered as related to the cost of making two parallel predictions. They also suggest that while the parser is maintaining different parses in parallel, it has built up a measurable preference for the preferred structure, as revealed by the larger P600 for the non-preferred reading. This appears consistent with our model results, though this remains to be verified with specific tests.

### Serial vs. Parallel Sentence Processing

Friederici et al [2] likewise suggested that their results were potentially consistent with parallel models of sentence parsing [21], [22]. Interestingly, this is consistent with the neural activity we observe in the striatal readout neurons – multiple parses are maintained, based on their probability within the corpus – analogous to the notions of “preferred” and “non-preferred.” These results are pertinent within the psycholinguistics community, where a debate continues concerning whether human sentence processing is based on always maintaining the single, best current parse candidate (serial processing) or rather, maintaining multiple open options, and pruning and updating these options as the sentence unfolds (parallel processing) [22], [60], [61]. One of the arguments against parallel processing is related to the cost and difficulty of maintaining multiple, parallel parses. In symbolic parsing models, each parallel path corresponds to a complete parser. In contrast, the neural parsing we see in the current research maintains multiple parallel representations as an inherent function of the system, with no additional resources required for maintaining these multiple parses. Our model corresponds to a ranked parallel model, where multiple structures may be pursued, but one of them may be more highly preferred [22], [61]. Indeed, it is impossible to avoid this parallel processing. In the future such neural implementations of sentence processing can thus contribute to the serial vs. parallel debate.

### Prior knowledge in discourse

In addition to single sentence processing, we also hoped to shed light on how a language system might integrate such information over more than one sentence in discourse. Hagoort and van Berkum [4] review two-stage models of discourse processing, in which a sentence is first processed alone, and then integrated with the ongoing discourse. They hold, however, that their ERP data argue in favor of an alternative “immediacy assumption” in which every source of information that can contribute to sentence interpretation, including prior sentences in the discourse, does so, immediately (see [4] for review). The immediacy assumption poses the question – how is the information accumulated over the discourse made available for immediate access? Our simulations suggest that this information can be accumulated and made accessible within recurrent cortical networks (the reservoir), which indeed maintain the global context of the unfolding discourse structure. We demonstrate this here with two-sentence discourses, and illustrate how the maintained context can be used to resolve ambiguous anaphoric reference in the second sentence that is disambiguated by the first. In general, however, syntactic information may not always be sufficient to resolve anaphoric reference. Future work remains, to explore more extended discourse situations and generalization in discourse.

### Related Language Models

The study of language processing in recurrent networks has a long and rich history. A number of studies have examined the ability of recurrent networks to predict the next word or word-category in a sentence, initiated by the seminal work of Elman [62]. There, Elman showed how the simple recurrent network (SRN) with plastic recurrent connections formed a representation of the underlying syntactic structure of the learned language. Interestingly, Tong [44] showed that with no learning, echo state networks (ESNs, a category of reservoir computing) perform equally well in the task of predicting the next word during sentence processing. This is very pertinent to the current study as it provides evidence that indeed, without learning inside the reservoir, properly constructed recurrent networks can inherently encode grammatical structure with this encoding then exploited via modifiable readout connections. Indeed Frank and Bod have recently demonstrated that in this context, an ESN actually accounted for human reading-time data by estimating surprisal values better than a phrase structure grammar model [58].

The crucial distinction with respect to our current work is related to the task. Tong et al. follow the Elman approach of predicting the next word in the sentence. Our task is fundamentally different. It involves extracting the meaning of the sentence, in terms of determining the thematic roles of the open class elements, or extracting “who did what to whom”. This type of sentence comprehension with neural networks has an equally long and rich history. It was addressed as part of the “parallel distributed processing” (PDP) or connectionist research effort [63], [64], and more recently in a more structured cognitive architecture [65]. Similarly, Chang develops a model of language production to address real-time effects including structural priming [66], [67]. Miikkulainen develops a multicomponent parsing system that generalizes over relative phrase structure, and also provides a thorough review of language-related neural network processing [64]. It is of particular interest that related neural network approaches have recently made significant technical progress in the domain of natural language processing, specifically in semantic role labeling [68], [69]. While these studies have addressed issues of thematic role assignment, they tend to introduce significant additional processing machinery, and have not addressed the real-time neural activation during sentence processing that is central to the current work. The novelty of our work is to develop a model that generates real-time parallel predictions of the thematic roles (or semantic labels) that provide potential insight into the underlying neurophysiological processing of language in the cortico-striatal system of man.

### Generalization

We can consider how the network performs this task, and how it can generalize. The recurrent network should be capable of separating distinct input sentence forms as well as grouping together sentence forms that may have small variation but essentially equivalent coded meaning. These two properties of the recurrent network are referred to as kernel quality and generalization capability in the technical literature [70]. This generalization refers to the ability to recognize two objects with small variation as belonging to the same category. This was revealed in the last part of Experiment 6 with the inclusion of adjectives and adverbs that thus generate redundant constructions (with similar sentence forms) which should be judged as being similar.

In addition to this form of generalization, the current task requires a more structurally advanced form of generalization that operates on the grammatical structure as encoded in the corpora used in our experiments. That is, the system should be able to accommodate new constructions that are not present in the training set, but whose structure can be derived from constructions that were present in the training set. This is distinct from generalization capacity of the recurrent neural network specified above which operates over small variations being characterized as similar. Here, the generalization may allow the system to correctly process constructions that are large variations from those seen in training, but that derive from the common grammatical structure inherent in the training corpus.

We can refer to this as grammatical generalization. Indeed, we observe that the system is able to extract structural regularities inherent in the corpora, and then use this information to generalize to new constructions. In related work, the ability of recurrent networks to accommodate this form of grammatical generalization have been observed [44], [45], [62]–[64].

### Scaling to larger more varied corpora

A limitation in our previous work was the small set of constructions used, and the corresponding absence of significant demonstration of generalization [24]–[27]. In the current research, using corpora of 462, and up to 90,000 distinct constructions, we demonstrate that the model can learn and generalize on the grammatical structure that is inherent in the training corpora. Indeed, as illustrated in Figure 11, we observed that as the size of the training set increases, so does the generalization capability. As the system gains greater exposure to the structure that defines the corpus, it can better generalize within the context of that corpus. We should recall that there is inherent ambiguity (12% of the sentences) in the corpus which limits optimal sentence error to approximately 12%, and our minimal sentence error in two-fold cross validation is 15.01% (±0.22).

The system can also demonstrate flexibility in coding. In Experiments 1–4 we only required specification with respect to the thematic roles of the nouns, and then in Experiments 5–7 we additionally required the system to include the predicate for action. Likewise, in Experiments 1–4 the first action in the coded meaning was associated with the first verb, while in Experiments 5–7 the first action was associated with the verb in the main clause, even if it came second in the sentence (as in a relative phrase such as “The man that hit the ball chased the dog.”). More generally, the coding scheme is flexible, and if information concerning different dimensions (such as tense) are coded in the meaning, then the system could learn to extract this information in the meaning representation.

### A final disclaimer

We have taken a rather bold stance in suggesting such a specific role for the cortico-striatal system, with cortex as a recurrent network and striatum as a readout for thematic role assignment. While this is coherent with a substantial amount of empirical and theoretical work [2], [9], [12], [71], it could well be possible that the processing we are considering is taking place at a different level, e.g. within purely cortico-cortical networks, though this would make the observation of P600 loss with basal ganglia lesions more difficult to explain [12]. Indeed, in [26] we suggested that the cortico-striatal mechanism would be used for re-analysis, and a direct Cortico-cortical mechanism would be used for over-learned canonical forms. This remains to be established by more detailed neurophysiological studies. The important notion to be retained is that a recurrent network with appropriate dynamics can accumulate and process information pertaining to grammatical structure, such that this information can be read-out to perform thematic role assignment, and that this is consistent with a mapping of recurrent network and readout onto the cortico-striatal system and the corresponding human neurophysiology. Such use of reservoir computing to model the functional neurophysiology of cortico-striatal function in cognitive tasks, as in the current work, and in [72] is a very promising area for future research.

## Materials and Methods

Our model makes a parallel between cortico-striatal anatomy and the reservoir computing framework. Prefrontal cortex (Brodmann area 47) is modeled as a fixed recurrent network and striatum as a separate population connected to cortex via modifiable synapses, corresponding respectively to the reservoir and readout. The reservoir is composed of leaky integrator neurons with sigmoid output activation. Equation (1) describes the internal update of activity in the reservoir:
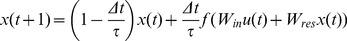
(1)where *x(t)* represents the reservoir state; *u(t)* denotes the input at time *t*; Δ*t* is the time precision; *τ* is the time constant; and *f(•)* is the hyperbolic tangent (tanh) activation function. The initial state of the internal state *x(0)* is zero. In reservoir computing terminology, the “leak rate” is equivalent to 1/*τ*. *W_in_* is the connection weight matrix from inputs to the reservoir and *W_res_* represents the recurrent connections between internal units of the reservoir. We will present input stimuli (words) one by one as input to the recurrent network. Each word is presented for an “activation time” (AT), which specifies the number of network time steps of the presentation. This AT enters into Equation 1 such that Δ*t* is equal to the reciprocal of AT (i.e. 1/AT). We empirically determine this in the methods section on Experiment 5, below, and provide simulation results in the Text S1. The linear readout layer is defined as:
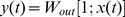
(2)Where 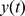 is the output (striatal) activity and 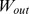 the output weight matrix. To learn the connection weights 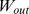 between the reservoir (BA 47) and the readout (striatum), we used ridge regression (see Training, below). The global readout activity represents the coded meaning of the input sentence.

The number of unit used in the reservoir, the spectral radius, the activation time, Δ*t* and the time constant are dependent on the experiment, but here we indicate the parameters that are common for most of them. The reservoir was typically composed of 1000 units. By definition, the matrices *W_in_* and *W_res_* are fixed and randomly generated. Internal weights (*W_res_*) are drawn from a normal distribution with mean 0 and standard deviation 1, with a connectivity of 10%. Then we rescale the spectral radius (SR), i.e. largest absolute eigenvalue of the generated matrix *W_res_* to 1. Input weight matrix *W_in_* has values chosen randomly to be 0.75 or −0.75 with equal probability. The density of the input connections is also 10%. A time constant *τ* of 6 (i.e. leak rate of ≈0.167) was used. The ridge regression parameter for which we observed correct performances was 10^−9^. SR and *τ* were varied in some cases, as described for specific experiments, below.

### Corpora

For experiments 1–4 a corpus of 45 constructions was used, presented in the Text S1. This was based on 26 of the constructions studied in [25], with extensions for discourse, relative clauses, etc.

For Experiments 5 and 6 an extended corpus of 462 constructions was created. Sentence forms (and the corresponding meanings) were generated from a context-free-grammar based on English phrase structure with verbs taking 1 to 3 arguments (e.g. *walk*, *cut* or *give* could have respectively 1, 2 or 3 arguments). The coded meanings were then generated for each construction, by matching the position of each semantic word in the sentence form with its role in the meaning. Each construction could have 0 or 1 relative clauses inserted after one of the nouns; the verbs of the relative clause could take 1 or 2 arguments. All possible semantic word orders were used. There are 6 possible semantic word orders for a verb with 2 arguments: APO, AOP, PAO, POA, OAP, OPA. Note that *Agent Predicate Object* (APO) is the most common in English. There are 24 possible semantic word orders for a verb with 3 arguments: APOR, APRO, AOPR, *etc*. All possible semantic word orders were generated both for main and relative clauses. All possible insertions of the relative clause were performed, i.e. after the 1st, 2nd or 3rd noun in the main clause. In total, 462 constructions were generated. In this corpus, all the nouns were preceded by “the” (i.e. they were common nouns).

For Experiment 7, as in Experiments 5 and 6, we constructed a context-free grammar that was used to generate a corpus of 90582 constructions. This corpus has 2043 distinct coded meanings, and thus it is redundant. In addition to complete meanings, this corpus contains “incomplete” *meanings* such as *give(_, Rex, John)* for a sentence like “Rex was given to John”, where there is no Agent in this *meaning*. In the “90582 corpus”, for each grammatical construction we generated similar constructions with variability. There are several types of variability as described in Experiment 7, above. The set of word orders employed was restricted to eliminate clearly non-intelligible constructions employing semantic word orders such as RP (i.e. Recipient-Predicate), or POAR. The corpus contains 12.24% ambiguous constructions (i.e. 11,088 constructions). Of these, 5,268 are ambiguous couples (pairs with the same surface form and different coded meanings), 184 are ambiguous triples (triplets with the same surface form and different coded meanings).

### Input and Output Coding

Here we will describe the general case for “462 corpus” that was used in Experiments 5 and 6. Given an input sentence, the model should assign appropriate thematic roles to each semantic word (in our case, nouns and verbs). Surface forms are presented to the model as input sequences, where specific instances of semantic words (SW) – noun and verb – are replaced by *SW* markers. Thus, a given surface form can code for multiple sentences, simply by filling in the *SW* markers with specific words. Input sequences are presented to the reservoir in a sequential order, one word at a time. Learning consists in modifying connections between reservoir and readout units, so as to specify the appropriate role assignments to each SW in the readout neurons, which are specified during learning. This is illustrated in Figure 1B.

Each dimension of the input codes one word or marker. Semantic words (also called open class or content words) are all coded with a single input neuron labeled SW. The input dimension is 13. This corresponds to: ‘-ed’, ‘-ing’, ‘-s’, ‘by’, ‘is’, ‘it’, ‘that’, ‘the’, ‘to’, ‘was’, with 10 input neurons for closed class words (CCW), 1 for SW, 1 for the comma and 1 for the period. Verb inflections (suffixes “-s”, “-ed”, “-ing”) are part of the CCWs because as grammatical morphemes they provide grammatical information. In the second part of Experiment 6 an additional input neuron was used to code adjectives and adverbs, thus yielding a total of 14 input neurons.

The number of readout units is 42 ( = 6*4*2–6): 6 semantic words, each of which could have one of the 4 possible thematic role (*predicate, agent object, recipient*) that could be related to both the main and relative clause. As a relative clause never employs a verb with more than 2 arguments (*i.e*. there is no *recipient*) we subtract 6 output units from the total. Inputs have value 1 when the corresponding word is presented, 0 otherwise. Teacher outputs has value 1 if the readout unit should be activated, −1 otherwise.

Experiments 1–4 used an equivalent but topologically modified coding. The total number of inputs was 14: 11 slots for close class words, 1 for nouns, 1 for verbs and 1 for the period. The read-out dimension for Experiments 1–4 is 4*3*2 = 24 (4 nouns that each could have 3 possible thematic role assignment, and each could have a role with at maximum 2 verbs).

For Experiment 7, we used a coding similar to that for the “462 corpus”. The input slots used are ‘by’, ‘is’, ‘to’, ‘that’, ‘the’, ‘who’, ‘-ed’, ‘-s’; ‘-m’, ‘*SW*’, *comma* and *period*; with the input dimension of 12. Note that ‘-m’ is the possible inflection of *who*, giving *whom*. The number of readout units is 56( = 7*4*2): 7 semantic words, each of which could have one of the 4 possible thematic roles (*predicate, agent object, recipient*), in the two predicate actions.

Recall that two sentences that have equivalent meaning (as illustrated in Figure 1) may have that meaning coded in different configurations of output neuron activation. Consider “John hit the ball” and “The ball was hit by John.” Both correspond to hit (John, ball). For the first sentence open-class word 1 is the agent, and in the second it is the object. We thus make the distinction between *meaning* and coded meaning. These two sentences have the same meaning, and different coded meanings.

The input signal for each word is coded as a square wave of AT (activation time) time steps. For experiments 1,2 and 4 we used an AT of 20, corresponding to Δ*t* = 0.05. For Experiment 3 we used ATs of 1, 10 and 20. We then we used an AT of 1, corresponding to Δ*t* = 1, for the remaining experiments (5–7). In other words for all experiments where generalization performance were computed using cross validation, we used an AT of 1 in order to increase the speed of the simulations. We verified that this did not change the behavior of the system. See details in the Text S1.

### Training

During training, inputs were presented to the reservoir, and the reservoir internal state trajectory was recorded. This data and the desired output was then used in a ridge regression (also known as Tikhonov regularization) to train the readout weights so as to minimize the mean squared error between the target output and the projection of the reservoir state through the trained readout weights. We used ridge regression rather than classic linear regression, as it effectively limits the magnitude of the weights, thus reducing the probability of overfitting and thus enhancing the generalization capabilities. See [38] for a review of reservoir learning methods.

We considered training conditions in which learning occurred starting at the presentation of the first word until the end of the sentence (*continuous learning*), or only at the end of the sentence (*sentence final learning*). In the first case, the system will begin to predict the meaning of the sentence from the outset of the sentence, and can thus display anticipatory and on-line activity. However, because the learning is distributed over the entire sentence, there can be a relative reduction in the final performance in terms of correctly learning the meaning.

### Error Measures

As stated above, we define the *coded meaning* of the sentence as the specification of the thematic roles for the semantic words (SW). This requires determining the individual coded meanings for each SW, as specified in the readout neurons. We refer to this fine grained level of word meaning as the *word level coded meaning*. A *word level coded meaning* is obtained from the concerned readout units in 2 steps. The activity is thresholded at 0, and a winner-takes-all between the 4 possible roles is performed. The winning role is considered as the *word level coded meaning* of the model for this SW. If there is no activity above the threshold, then no *word level coded meaning* is considered for this SW. During learning, input sentences are presented, and the corresponding readouts coding the meaning are activated, and the system should learn the weights from the reservoir to readout.

Two error measures were evaluated: the *meaning error* and the *sentence error*. Meaning error is the percentage of *word level coded meanings* whose activity was incorrect (i.e. the percentage of winning readout neurons whose activity is incorrect). Sentence error is the percentage of sentences that were not fully understood (i.e. sentences in which there is at least one erroneous *word level coded meaning*). We use these two comparative measures because sentence error gives a stricter measure while meaning error is more lenient and accounts for partial understanding. These two measures are related but not strictly correlated, as 10 wrong *word level coded meanings* could all be associated with one sentence, or with 10 different sentences. The analysis is restricted to readouts for semantic words that are relevant, i.e. for a sentence with 2 semantic words we analyse readouts only corresponding to the first two semantic words. For instance if there are only 2 semantic words in a construction, if one of them is erroneous, the *meaning* error will be 0.5 and the *sentence* error will be 1. In sentence 26 (see Text S4) “Walk-ing was the giraffe that think-s,” there are two clauses (main and relative), and three semantic words. Each of the three semantic words (walk, giraffe, think) can potentially take a role in either clause. Thus there are 6 relevant output mappings of semantic words to meanings, or *word level coded meanings*, for this sentence. This means that a word that participates in the main and relative clause will have two *word level coded meanings*, one for each clause. In the 462 corpus, 88% of the sentences have 10 or 12 relevant output mappings or *word level coded meanings*.

Thus, learning results will be presented in terms of these two factors: error type (sentence error and meaning error), and learning method (continuous learning and sentence final learning).

### Experiment-Specific Simulation Conditions and Parameters

Python code for Experiments 1–4 can be found in the Zipped Archive S1. The Text S3 has instructions on how to install and run the model. Below are details on specific parameters for the different experiments.

#### Experiment 1: Basic Syntactic Comprehension

Learning and on-line processing with a set of 26 constructions: constructions numbered 15–40 in the Text S1 (see results in Figure 2). The reservoir was composed of 300 internal units. For Experiments 1–4 semantic words were coded with two separate input neurons for nouns (N) and verbs (V). Meaning was evaluated only for nouns, in assigning the thematic roles of agent, object and recipient (see Input and Output Coding).

#### Experiment 2: Neural Coding Explanation of P600

On-line processing of subject- and object-relatives with different training corpora. The training set was augmented with four object-relative constructions corresponding to constructions 41–44. The final set thus consisted of constructions 15–44 in the Text S1 (see results in Figure 3). In order to render the corpus more similar in its distribution of construction types to that observed in human language [42], we used constructions 15–41 (thus keeping one object-relative – construction 41), and removed all passive-relatives (with the “…that was V” component), so that subject-relative was more frequent than object-relative and passive-relative (see Figure 4). All removed constructions are marked with “*” in the corpus in Text S1. 300 internal units were used. Figure 5 then presents the change in neural activity that can be compared with ERP P600 responses.

#### Experiment 3: Generalization

In order to test the ability of the system to generalize to constructions not presented in the training set, we performed “leaving one out” simulations, where each construction is systematically removed from the training set and then tested. Constructions numbered 15–40 were used (See Figure 6). 100 internal units were used.

#### Experiment 4: Short discourse processing

This involves the use of two successive sentences. This discourse sentences are 0–4, 10–14, and they were included in the whole 45 element corpus. (See Figure 7). 300 internal units were used. For all ten discourse fragments, the first sentence contained one proper noun and one common noun, and the second sentence contained the pronouns “he” and “it”. In the corpus, “he” systematically refers to the proper noun referent, and “it” to the common noun referent.

#### Experiment 5: Extended corpus I

Here we use the extended corpus of 462 constructions. Again, we considered conditions in which learning occurred starting at the presentation of the first word until the end of the sentence (*continuous learning*), or only at the end of the sentence (*sentence final learning*). In the first case, the system will begin to predict the meaning of the sentence from the outset of the sentence, and can thus display anticipatory and on-line activity. Because of these different learning conditions we selected the optimal coupled parameter set for sentence error in both conditions: spectral radius (SR) of 1 and time constant (τ) of 6 for sentence final learning (SFL), and spectral radius of 6 and time constant (τ) of 55 for continuous learning (CL*).

Because of increased simulation time with larger corpora in this and subsequent experiments, we tested whether reduction in the activating time for each input from 20 to 1 would yield equivalent results. Demonstration that this is equivalent is presented in the Text S1. For sake of simplicity and because the performance is robust on a wide range of parameters, in the rest of the experiments, we retain two sets of coupled parameters (spectral radius, time constant): (SR = 1, τ = 6) and (SR = 6, τ = 55).

This section includes (a) an initial test of learning (with no cross validation) with a reservoir size of 1000 units, (b) a parameter sensitivity test (grid search) using cross validation with this network, and (c) a test of the effect of the reservoir size, again with cross validation (see Figures 8 and 9).

#### Experiment 6: Effects of Training Corpus Structure

The purpose of this experiment was to test the ability of the system to generalize in the absence of grammatical structure. It is thus to be considered as a control experiment. In order to do this, we scrambled the word order in the 462 construction corpus, and then proceeded with training and testing as Experiment 5. As an additional control to demonstrate that the system is robust to noise in the training corpus, we added an input neuron corresponding to adjectives and adverbs that could be inserted before semantic words, without changing the meaning of the sentence. For each construction, 5 new variations were created by adding adjectives/adverbs randomly before SWs in the surface form. This yielded a corpus of size 462*6 = 2772. In the cross validation, we ensured that constructions from the same redundant set were not used in training and testing, i.e. that the cross-validation tested generalization to new constructions.

#### Experiment 7: Extended Corpus II

This experiment examines generalization performance as a function of the size of the training set. The large (>90K constructions) corpus was used, with progressive exposure to increasing proportions of the whole corpus during successive cross-validation testing. For this experiment we used two-fold cross validation with sub-sets of the corpus varying from 6.25% to 50% of the corpus.

## Supporting Information

Figure S1 **Simulation results with same conditions as Experiment 1 but with reservoir size N = 1000, and activation time AT  = 20.** Note that for each output neuron, the temporal profile of activation is the same as that in Figure 2, obtained with N = 300, AT  = 20.
(TIF)

Figure S2 **Simulation results with same conditions as Experiment 1 but with reservoir size N = 100, and activation time AT  = 20.** Note that when compared with Figure S1, the temporal profile of activation for the output neurons is globally the same, but with increased variability.
(TIF)

Figure S3 **Simulation results with same conditions as Experiment 1 but with reservoir size N = 100, and activation time AT  = 1.** Note that when compared with Figures S1 and S2, the temporal profile of activation for the output neurons is globally the same, but with increased variability.
(TIF)

Text S1 **Additional information about the set of grammatical constructions for Experiments 1–4, and tests with different input activation times.**
(DOC)

Text S2 **Detailed neural activity for all constructions tested in Experiment 1.** The format of this data is identical to that of Figures 2–7 of the main text.
(PDF)

Text S3 **Read-me file with instructions on how to install and run the model given in the Zipped Archive S1.**
(TXT)

Text S4 **The 462 construction corpus.**
(RTF)

Zipped Archive S1 **This is a zipped file containing python code using the Oger toolbox for running simulations corresponding to Experiments 1–4, along with documentation for installation and running the scripts.**
(ZIP)
