# Supplementary figures and images for: Real-Time Parallel Processing of Grammatical Structure in the Fronto-Striatal System: A Recurrent Network Simulation Study Using Reservoir Computing
Source: PLoS One. 2013 Feb 1;8(2):e52946. doi: 10.1371/journal.pone.0052946 (PMC3562282; doi:10.1371/journal.pone.0052946)

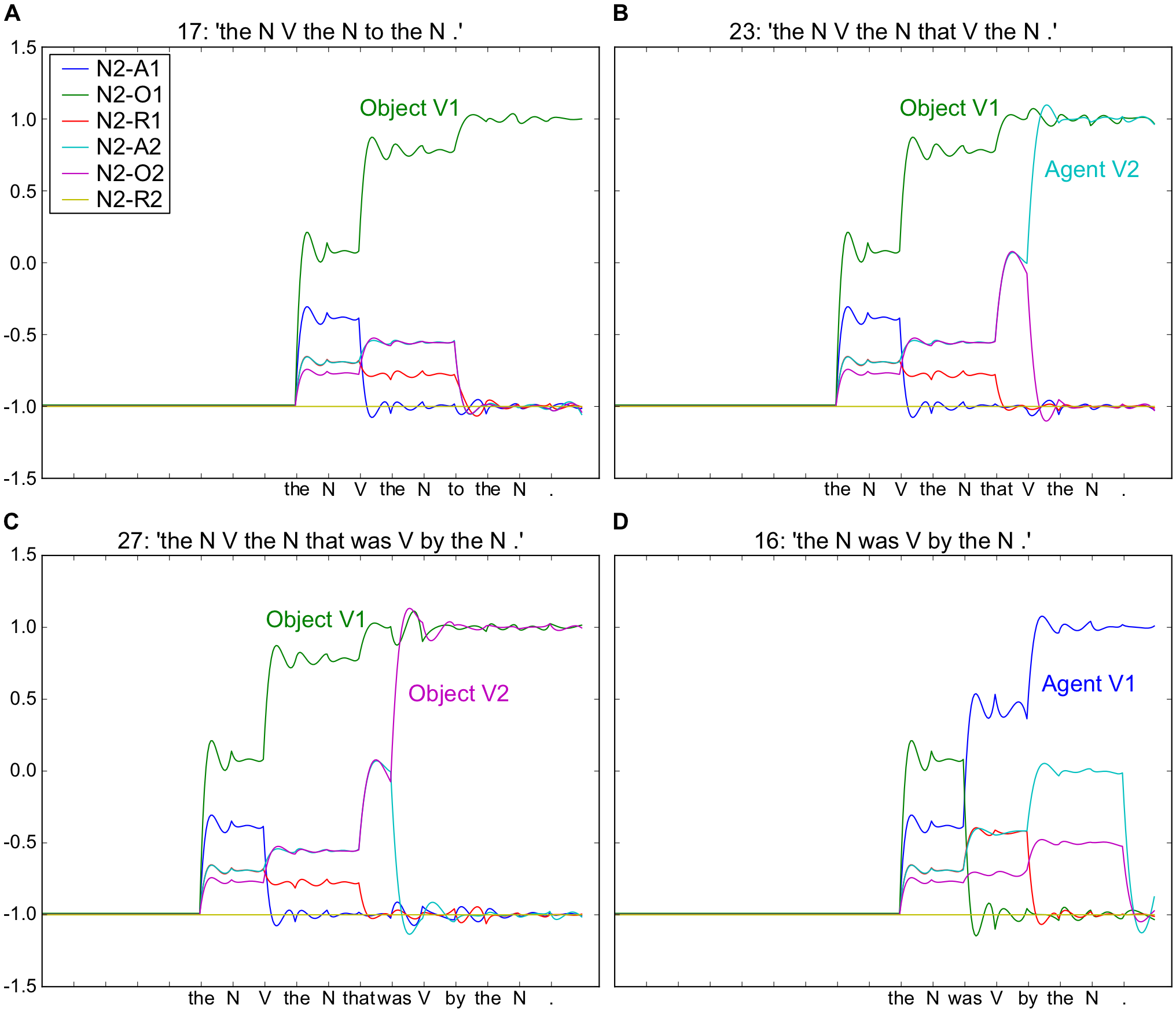

Supplement: Figure S1 — Simulation results with same conditions as Experiment 1 but with reservoir size N = 1000, and activation time AT = 20. Note that for each output neuron, the temporal profile of activation is the same as that in Figure 2, obtained with N = 300, AT = 20. (TIF) [file pone.0052946.s001.tif]

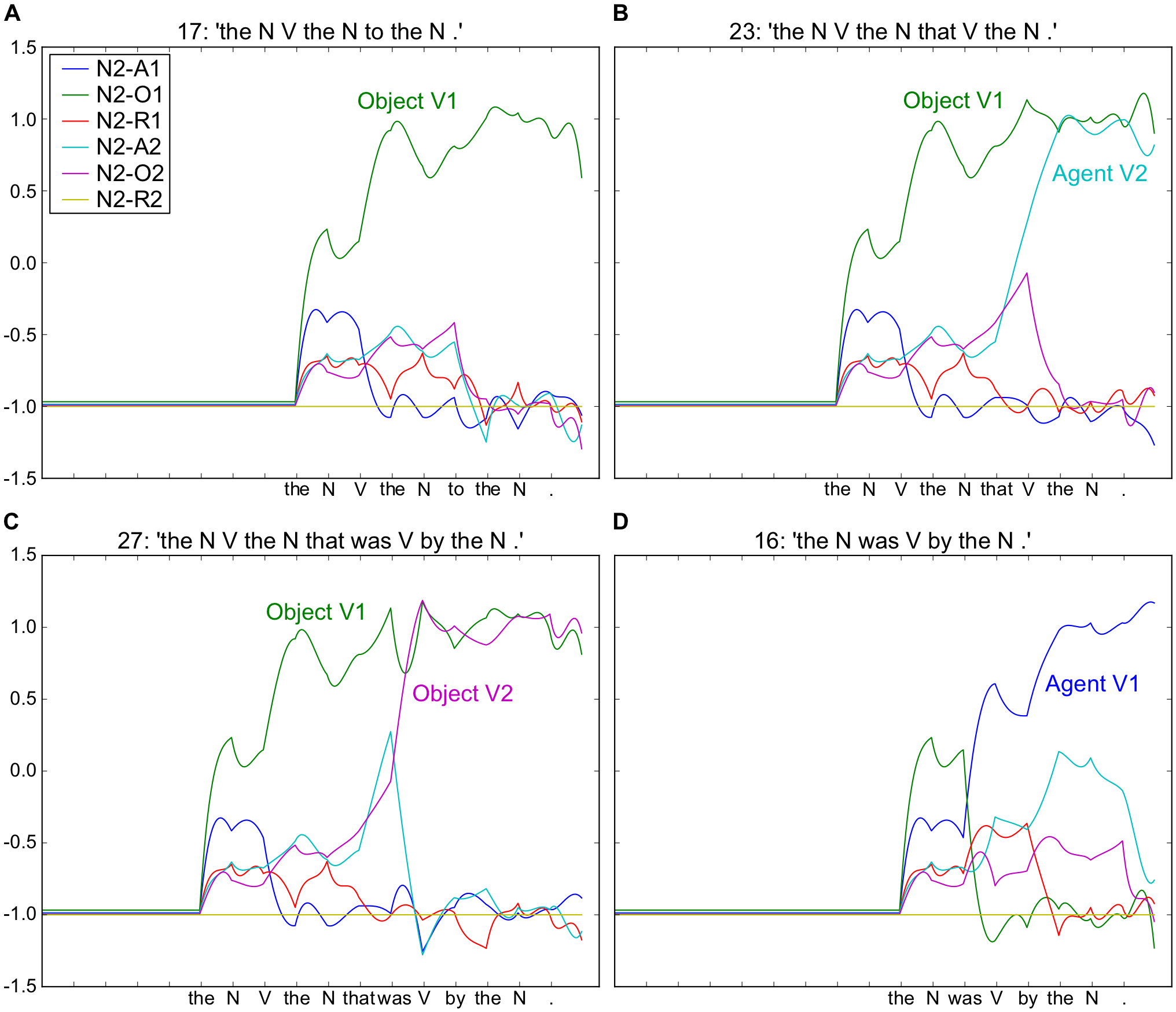

Supplement: Figure S2 — Simulation results with same conditions as Experiment 1 but with reservoir size N = 100, and activation time AT = 20. Note that when compared with Figure S1, the temporal profile of activation for the output neurons is globally the same, but with increased variability. (TIF) [file pone.0052946.s002.tif]

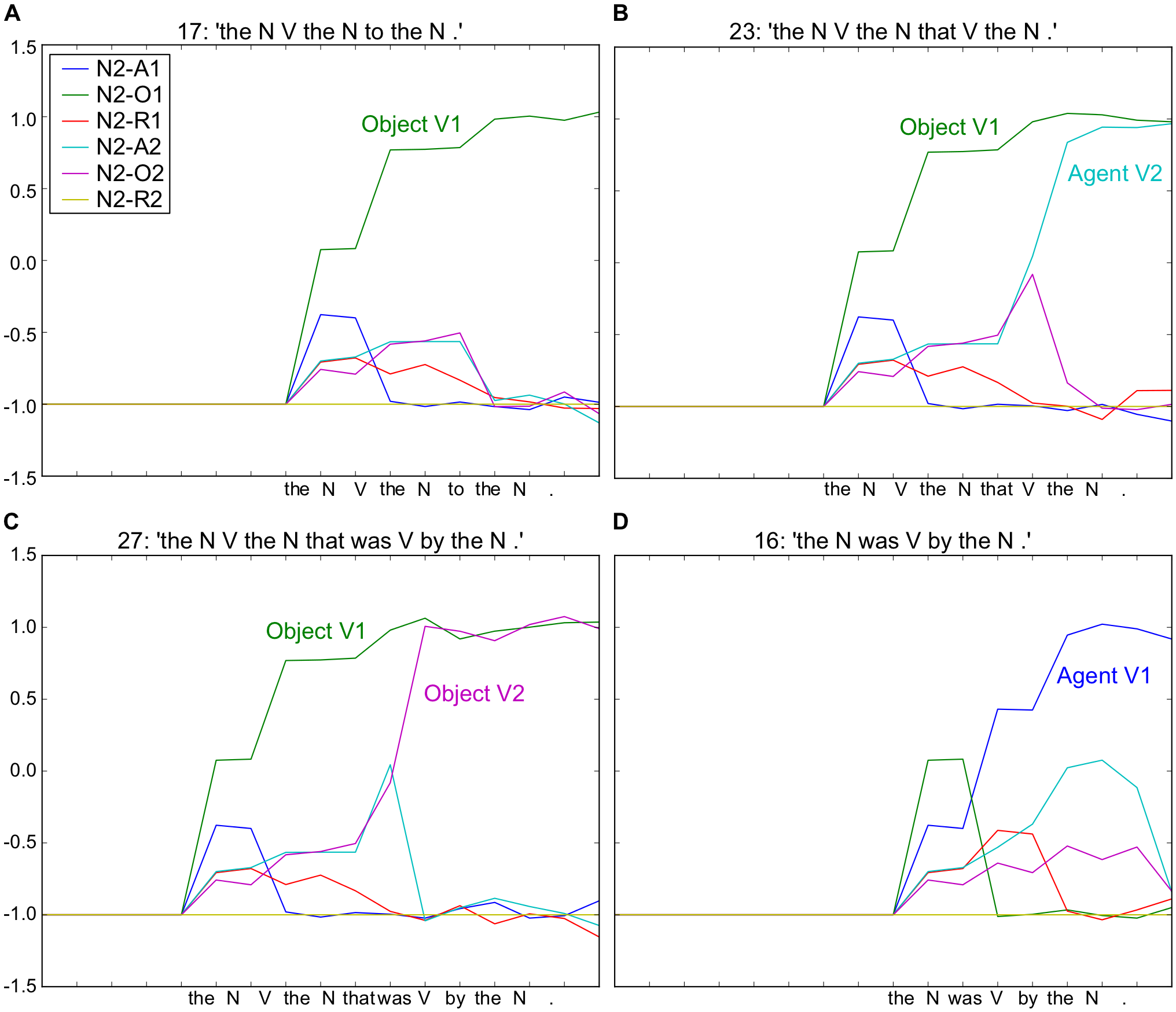

Supplement: Figure S3 — Simulation results with same conditions as Experiment 1 but with reservoir size N = 100, and activation time AT = 1. Note that when compared with Figures S1 and S2, the temporal profile of activation for the output neurons is globally the same, but with increased variability. (TIF) [file pone.0052946.s003.tif]
