# Supplementary material for: Real-Time Parallel Processing of Grammatical Structure in the Fronto-Striatal System: A Recurrent Network Simulation Study Using Reservoir Computing
Source: PLoS One. 2013 Feb 1;8(2):e52946. doi: 10.1371/journal.pone.0052946 (PMC3562282; doi:10.1371/journal.pone.0052946)
Supplement: Text S1 — Additional information about the set of grammatical constructions for Experiments 1–4, and tests with different input activation times. (DOC) [file pone.0052946.s004.doc]

**Supplementary Material S1**

This supplementary information contains 2 parts. Part 1 defines the details of the constructions used in Experiments 1-4. Part 2 exposes simulation results demonstrating equivalent performance with a variation of number of reservoir units and stimulus presentation durations.

**Part 1**

Here we provide the complete set of grammatical constructions (including their surface forms and coded meanings)for Experiments 1-4. Surface forms are specified with their corresponding thematic roles for the first, second, third and fourth nouns, labeled N1, N2, N3 and N4. Thematic roles correspond to Agent (A), Object (O) and recipient (R). Constructions are labeled 0 - 45. Secondary label in ()’s corresponds to the 26 constructions from . Items labeled with * are those that were removed in Experiment 3 in order to attain a distribution of subject- and object-relative and passive constructions corresponding with those in natural human corpora .

0 N V the N .then he V it . N1-A1 N2-O1 N1-A2 N2-O2

1 the N was V by N .then he V it . N2-A1 N1-O1 N2-A2 N1-O2

2 N V the N on the N .then he V it . N1-A1 N2-O1 N3-R1 N1-A2 N2-O2

3 the N was V on the N by N .then he V it . N3-A1 N1-O1 N2-R1 N3-A2 N1-O2

4 it was on the N that N V the N .then he V it . N2-A1 N3-O1 N1-R1 N2-A2 N3-O2

5 N V the N . N1-A1 N2-O1

6 the N was V by N . N2-A1 N1-O1

7 N V the N on the N . N1-A1 N2-O1 N3-R1

8 the N was V on the N by N . N3-A1 N1-O1 N2-R1

9 it was on the N that N V the N . N2-A1 N3-O1 N1-R1

10 N V the N .then it V him . N1-A1 N2-O1 N2-A2 N1-O2

11 the N was V by N .then it V him . N2-A1 N1-O1 N1-A2 N2-O2

12 N V the N on the N .then it V him . N1-A1 N2-O1 N3-R1 N2-A2 N1-O2

13 the N was V on the N by N .then it V him . N3-A1 N1-O1 N2-R1 N1-A2 N3-O2

14 it was on the N that N V the N .then it V him . N2-A1 N3-O1 N1-R1 N3-A2 N2-O2

15 (0) the N V the N . N1-A1 N2-O1

16 (1) the N was V by the N . N2-A1 N1-O1

17 (2) the N V the N to the N . N1-A1 N2-O1 N3-R1

18 (3) the N was V to the N by the N . N3-A1 N1-O1 N2-R1

19 (4) the N V the N the N . N1-A1 N3-O1 N2-R1

20 (5) the N that V the N V the N . N1-A1 N2-O1 N1-A2 N3-O2

21 (6) the N was V by the N that V the N . N2-A1 N1-O1 N2-A2 N3-O2

22 (7) the N that V the N was V by the N . N1-A1 N2-O1 N3-A2 N1-O2

23 (8) the N V the N that V the N . N1-A1 N2-O1 N2-A2 N3-O2

24 (9)* the N that was V by the N V the N . N2-A1 N1-O1 N1-A2 N3-O2

25 (10)* the N was V by the N that was V by the N . N2-A1 N1-O1 N3-A2 N2-O2

26 (11)* the N that was V by the N was V by the N . N2-A1 N1-O1 N3-A2 N1-O2

27 (12)* the N V the N that was V by the N . N1-A1 N2-O1 N3-A2 N2-O2

28 (13) the N was V to the N by the N that V the N .N3-A1 N1-O1 N2-R1 N3-A2 N4-O2

29 (14) the N that V the N was V to the N by the N .N1-A1 N2-O1 N4-A2 N1-O2 N3-R2

30 (15) the N V the N to the N that V the N .N1-A1 N2-O1 N3-R1 N3-A2 N4-O2

31 (16) the N was V from the N to the N that V the N . N2-A1 N1-O1 N3-R1 N3-A2 N4-O2

32 (17)* the N that was V by the N V the N to the N .N2-A1 N1-O1 N1-A2 N3-O2 N4-R2

33 (18)* the N V the N to the N that was V by the N .N1-A1 N2-O1 N3-R1 N4-A2 N3-O2

34 (19) the N that V the N to the N V the N .N1-A1 N2-O1 N3-R1 N1-A2 N4-O2

35 (20) the N was V by the N that V the N to the N .N2-A1 N1-O1 N2-A2 N3-O2 N4-R2

36 (21) the N V the N that V the N to the N .N1-A1 N2-O1 N2-A2 N3-O2 N4-R2

37 (22) the N that V the N to the N was V by the N .N1-A1 N2-O1 N3-R1 N4-A2 N1-O2

38 (23)* the N that was V to the N by the N V the N .N3-A1 N1-O1 N2-R1 N1-A2 N4-O2

39 (24)* the N V the N that was V by the N to the N .N1-A1 N2-O1 N3-A2 N2-O2 N4-R2

40 (25) the N that V the N V the N to the N .N1-A1 N2-O1 N1-A2 N3-O2 N4-R2

41 the N that the N V V the N . N1-O1 N1-A2 N2-A1 N3-O2

42* the N that the N V was V by the N . N1-O1 N1-O2 N2-A1 N3-A2

43* the N that the N V V the N to the N .N1-O1 N1-A2 N2-A2 N3-O2 N4-R2

44* the N that the N V V the N the N .N1-O1 N1-A2 N2-A2 N3-R2 N4-O2

**Part 2**

As the corpora sizes increased in Experiments 5-7, and the number of simulations required to perform cross-validation began to introduce significant increases in simulation time for Experiments 3 and 5-7, we examined the effects of reducing the number of simulation time steps per stimulus presentation. In Experiment 1 each element in the surface form input sequence was presented for 20 time steps. To achieve a speed-up of 20x, we considered reducing this value to 1 time step.

On Figures S1, S2 and S3 we display simulation results that replicate Experiment 1, but with different combinations of reservoir size (N), and activation times (AT) for the input stimuli.

In the original Experiment 1 illustrated in Figure 1 (main text), N=300, AT = 20.

In Figure S1 N = 1000, AT = 20.

In Figure S2 N = 100, AT = 20.

In Figure S3 N = 100, AT = 1.

(Note: these simulations can be performed using the model code provided and documented respectively in Supplementary Material Zipped Archive S3 and Text S3).

What we can observe across all four conditions (the three conditions here, and the original Experiment 1) is that the network learns with no error, and the topology or temporal form of activation of the readout neurons follows the same canonical trajectories. This illustrates that the coded meaning of constructions is not significantly modified with changes in N and AT. We took this as satisfactory justification to reduce AT in the generalization testing in Experiment 3 and in Experiments 5-7.

**References:**

1. Dominey PF, Hoen M, Inui T (2006) A neurolinguistic model of grammatical construction processing. J Cogn Neurosci 18: 2088-2107.

2. Roland D, Dick F, Elman JL (2007) Frequency of Basic English Grammatical Structures: A Corpus Analysis. J Mem Lang 57: 348-379.
