# Supplementary material for: Real-Time Parallel Processing of Grammatical Structure in the Fronto-Striatal System: A Recurrent Network Simulation Study Using Reservoir Computing
Source: PLoS One. 2013 Feb 1;8(2):e52946. doi: 10.1371/journal.pone.0052946 (PMC3562282; doi:10.1371/journal.pone.0052946)
Supplement: Text S2 — Detailed neural activity for all constructions tested in Experiment 1. The format of this data is identical to that of Figures 2–7 of the main text. (PDF) [file pone.0052946.s005.pdf]

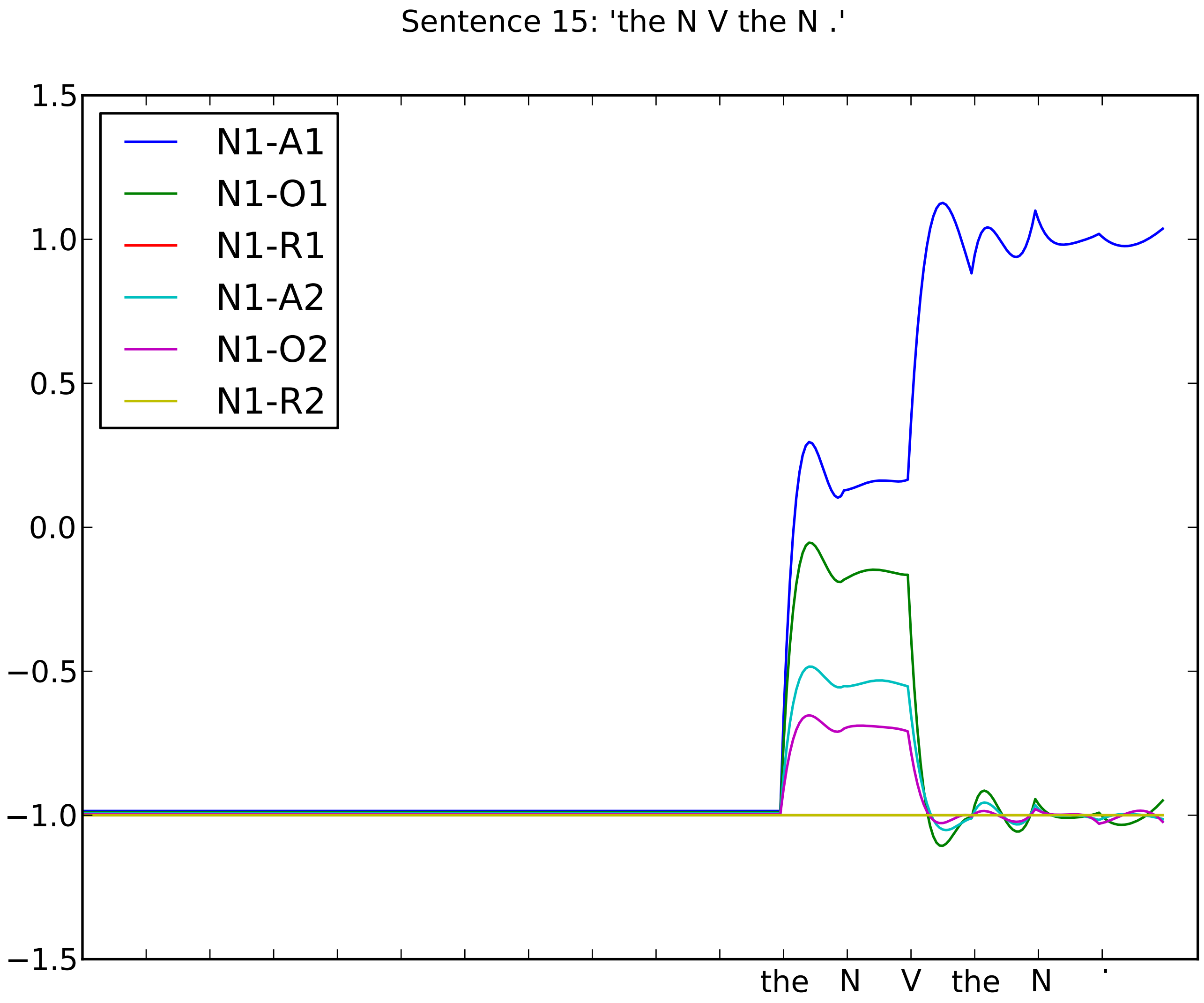

Sentence 15: 'the N V the N .'
1.5
1.0
0.5
0.0
−0.5
−1.0
−1.5
N1-A1
N1-O1
N1-R1
N1-A2
N1-O2
N1-R2
the
N
V
the
N
.

Sentence 15: 'the N V the N .'

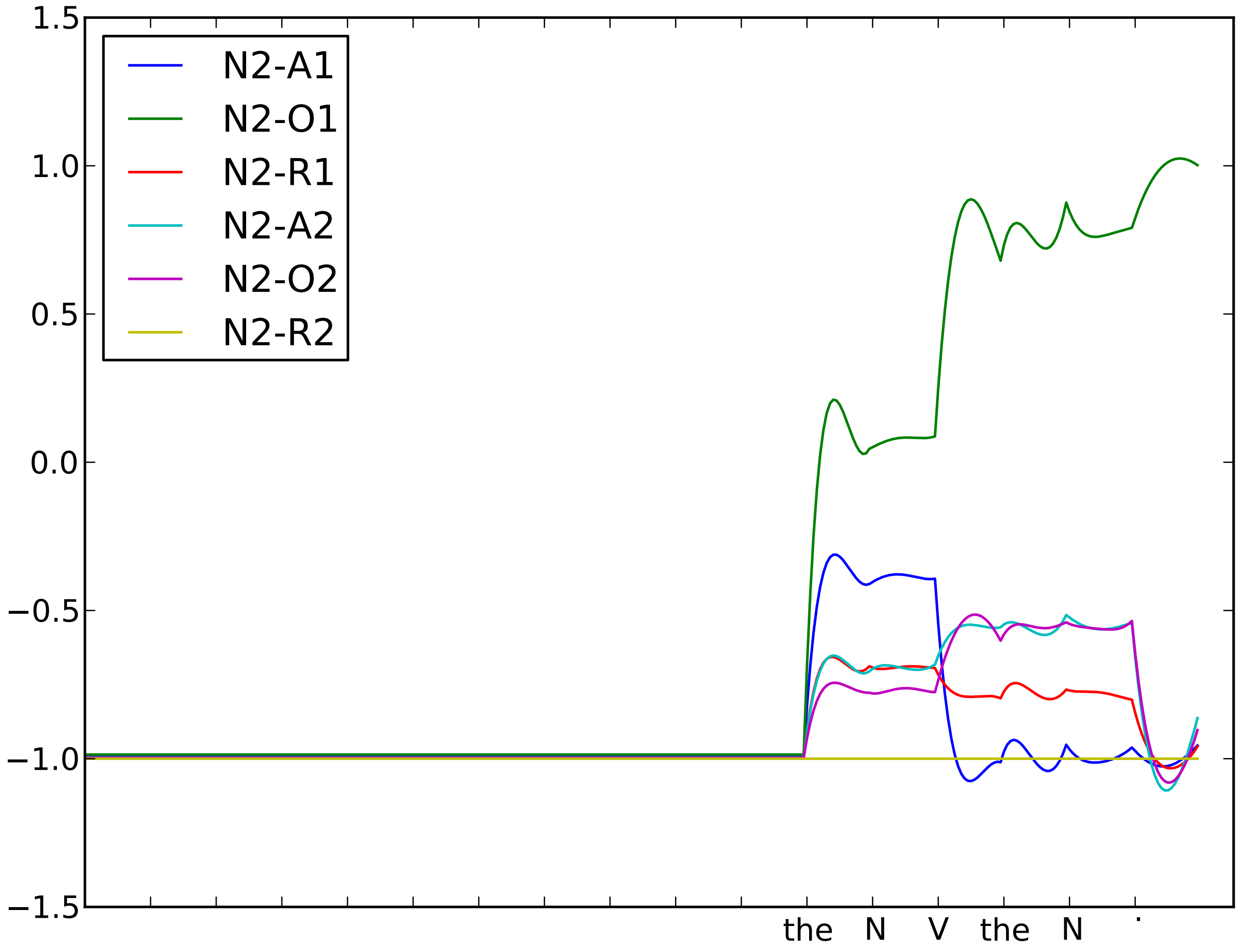

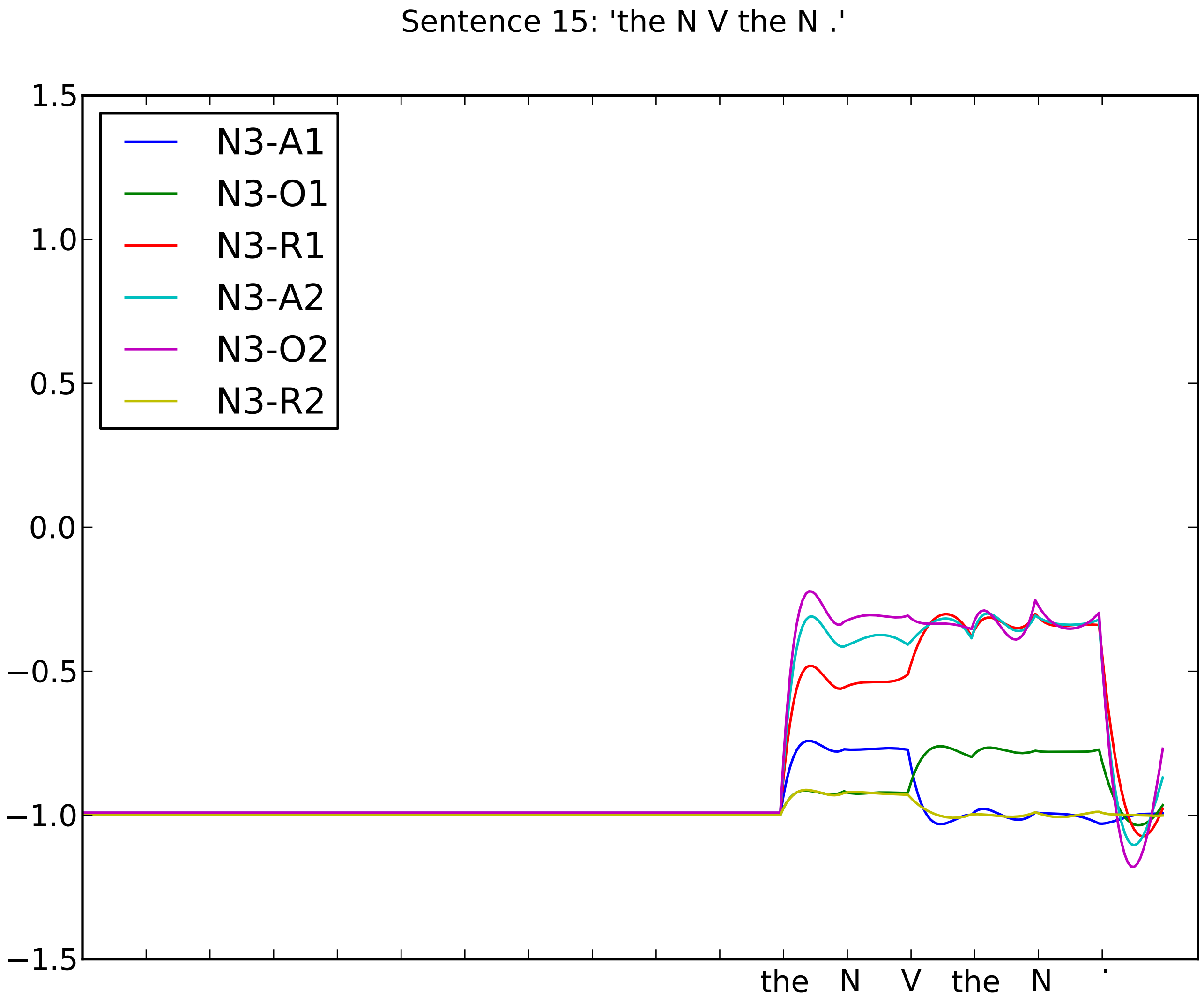

Sentence 15: 'the N V the N .'
1.5
1.0
0.5
0.0
−0.5
−1.0
−1.5
N3-A1
N3-O1
N3-R1
N3-A2
N3-O2
N3-R2
the
N
V
the
N
.

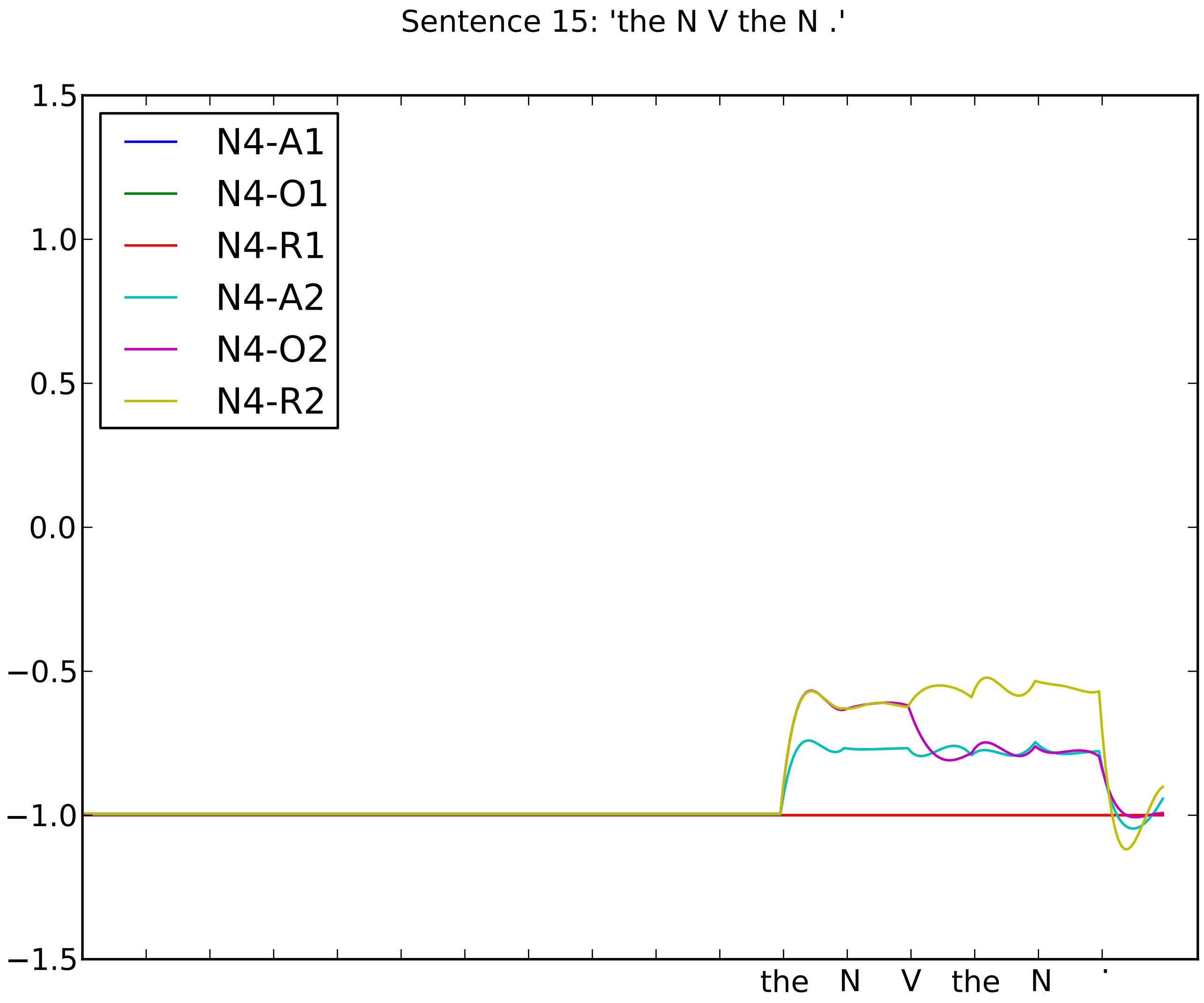

Sentence 15: 'the N V the N .'
N4-A1
N4-O1
N4-R1
N4-A2
N4-O2
N4-R2
1.5
1.0
0.5
0.0
−0.5
−1.0
−1.5
the
N
V
the
N
.

Sentence 16: 'the N was V by the N .'

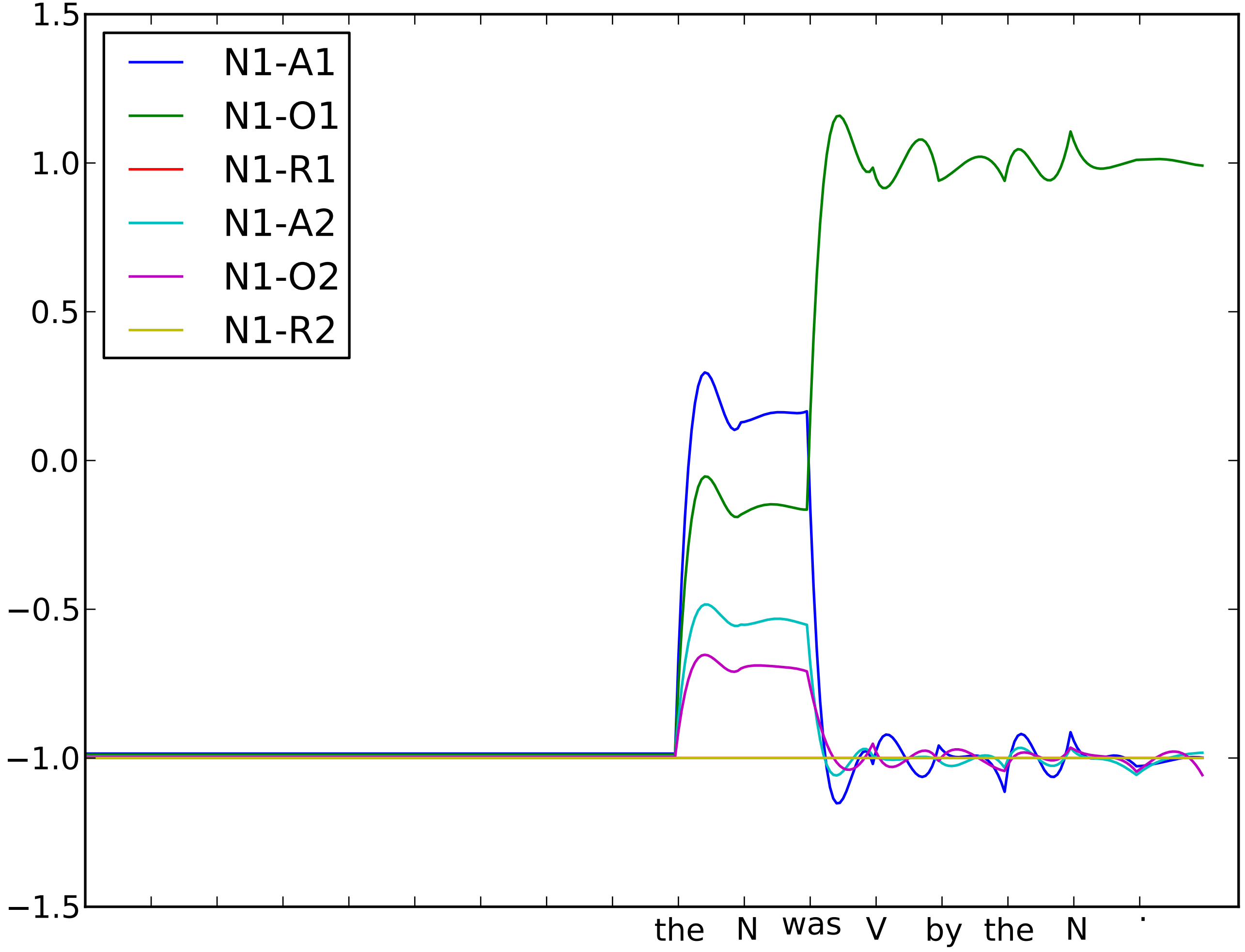

Sentence 16: 'the N was V by the N .'

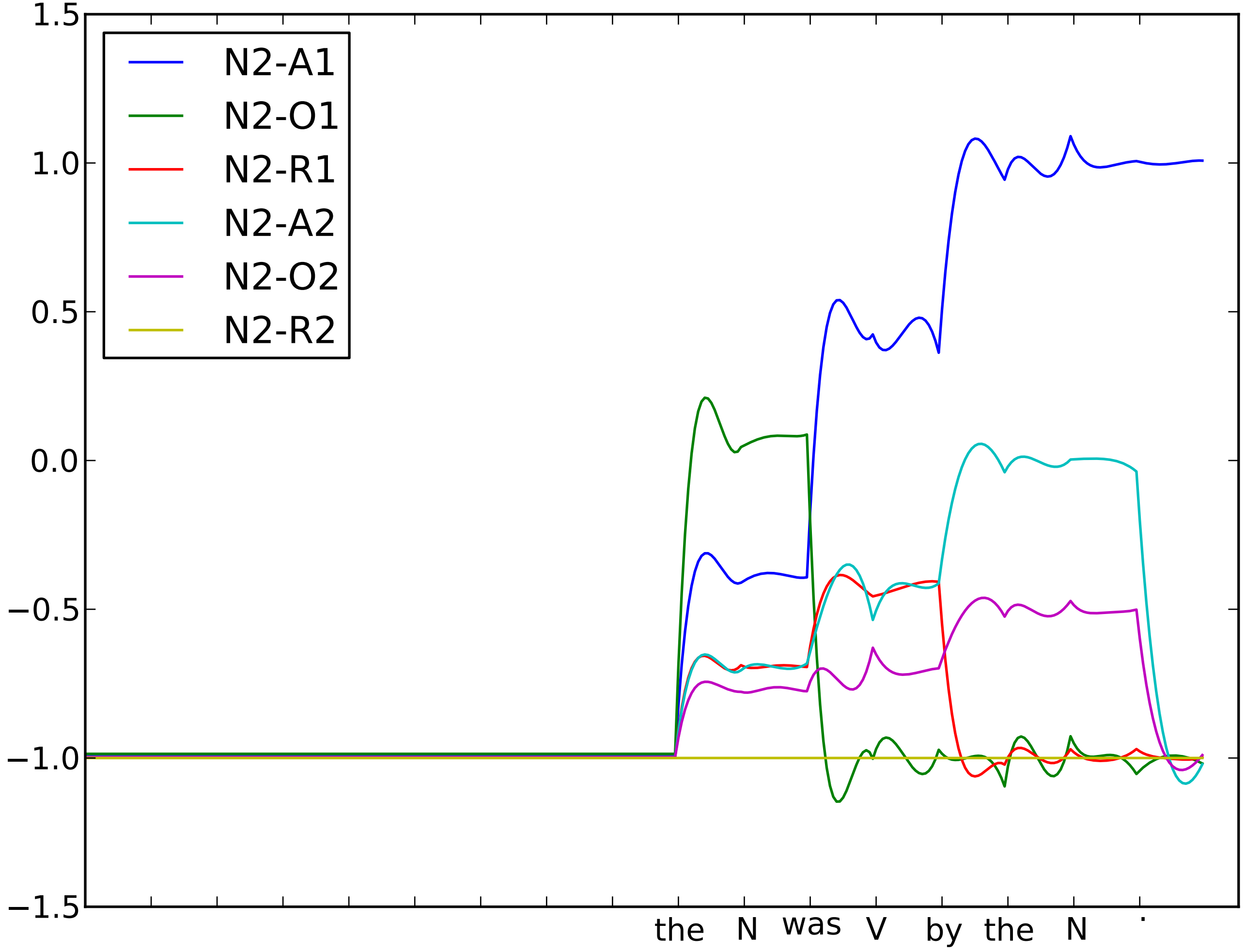

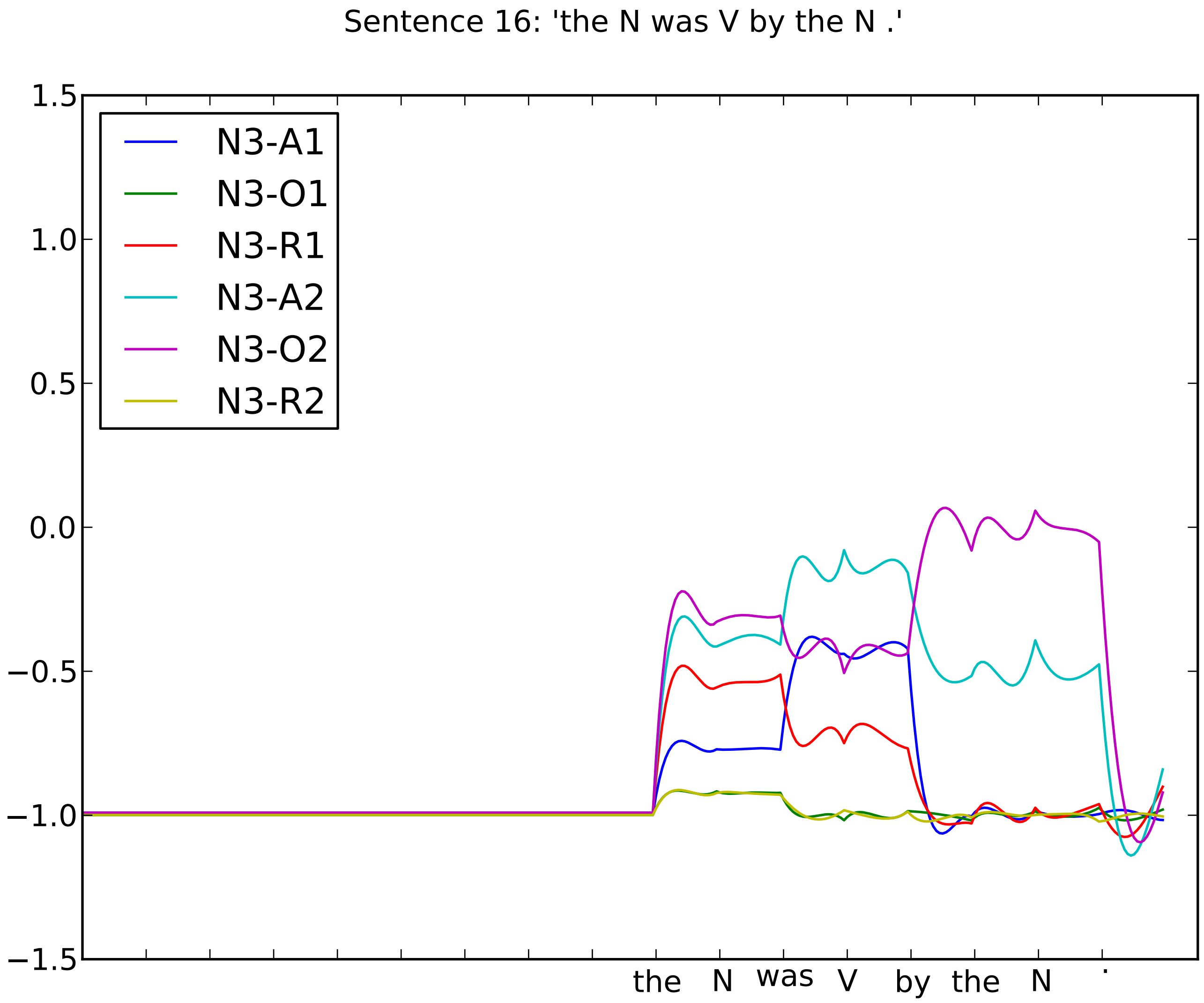
Sentence 16: 'the N was V by the N .'
1.5
1.0
0.5
0.0
−0.5
−1.0
−1.5
N3-A1
N3-O1
N3-R1
N3-A2
N3-O2
N3-R2
the
N
was
V
by
the
N
.

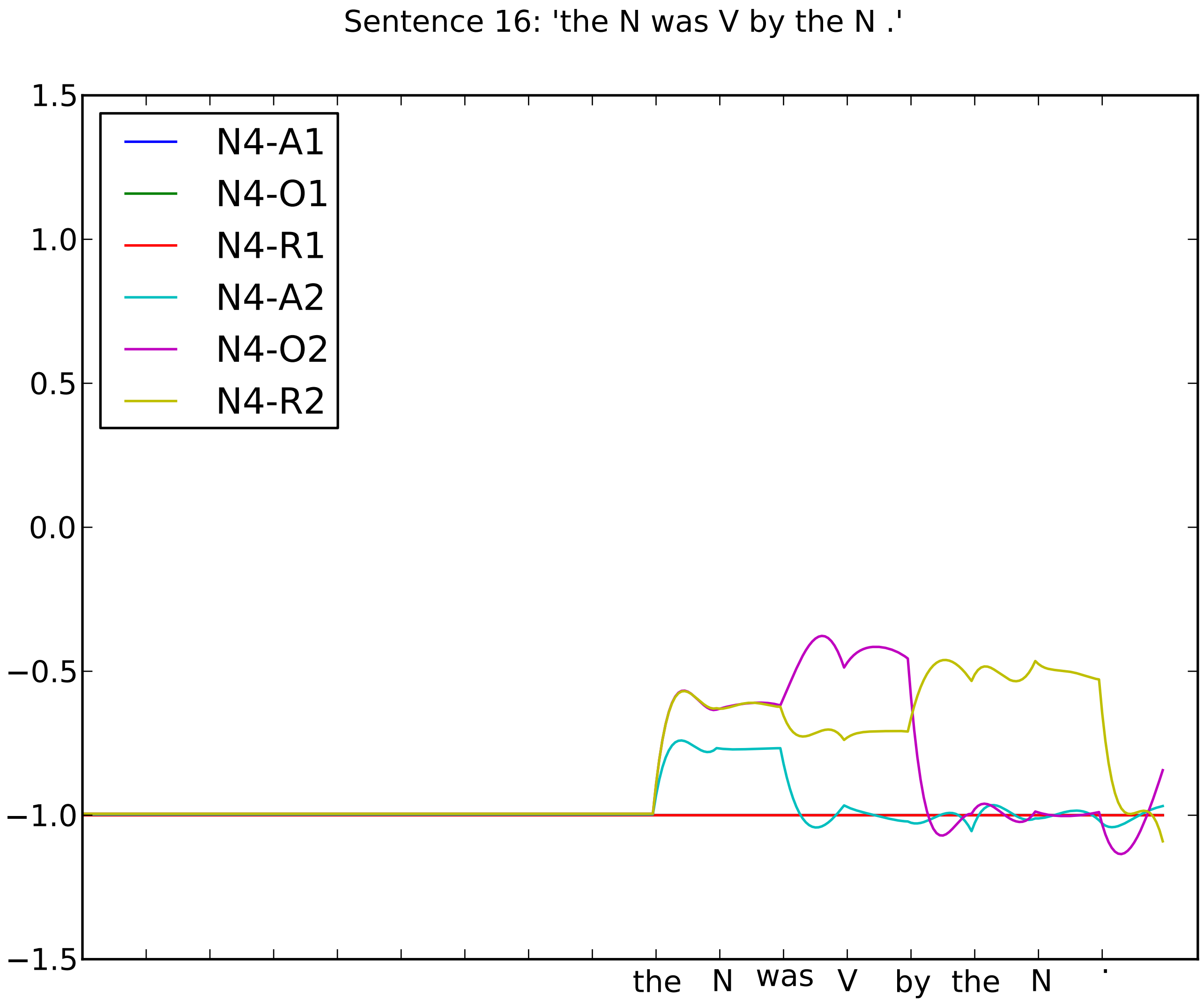
Sentence 16: 'the N was V by the N .'
1.5
1.0
0.5
0.0
−0.5
−1.0
−1.5
N4-A1
N4-O1
N4-R1
N4-A2
N4-O2
N4-R2
the
N
was
V
by
the
N
.

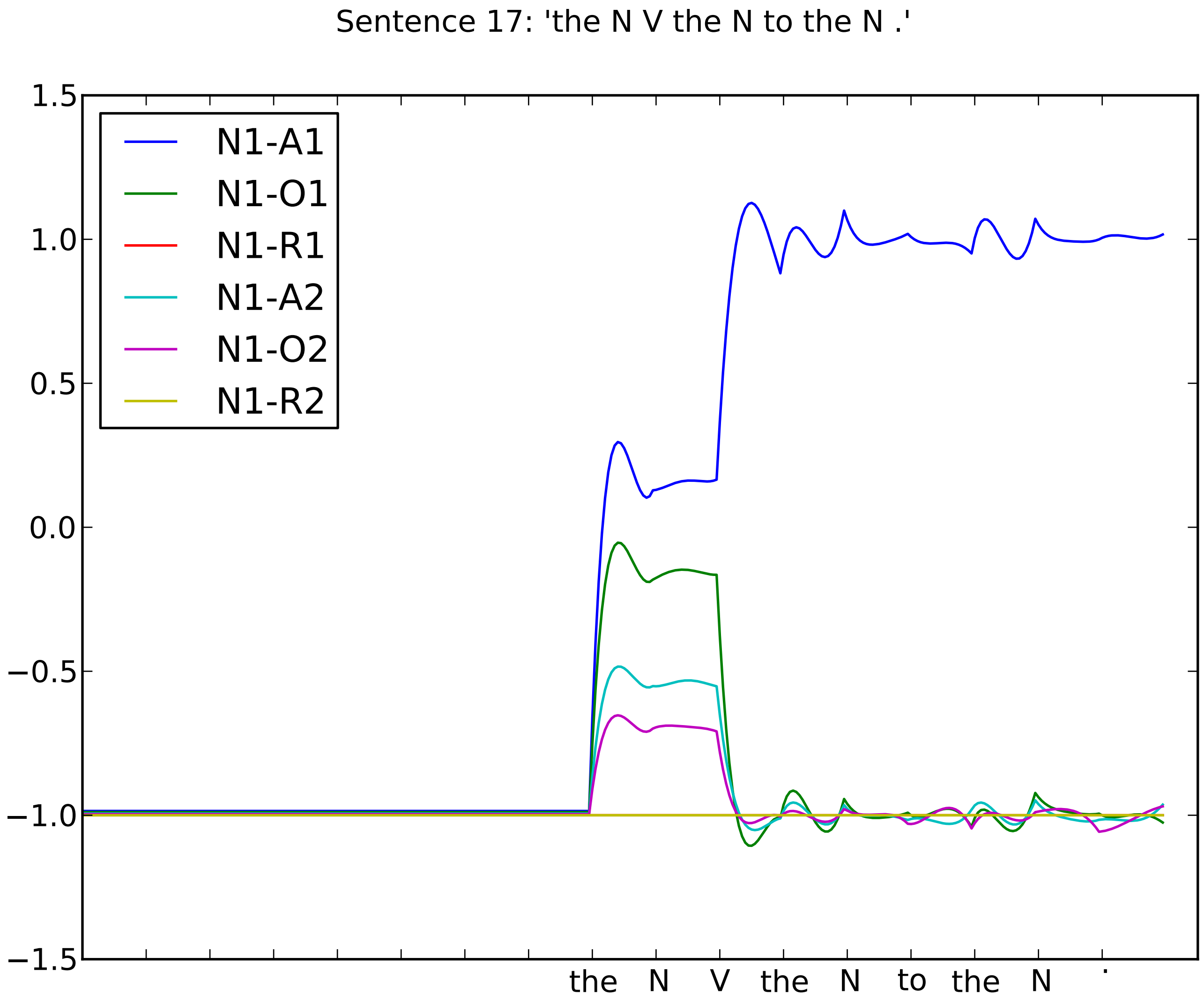
Sentence 17: 'the N V the N to the N .'
N1-A1
N1-O1
N1-R1
N1-A2
N1-O2
N1-R2
1.5
1.0
0.5
0.0
−0.5
−1.0
−1.5
the
N
V
the
N
to
the
N
.

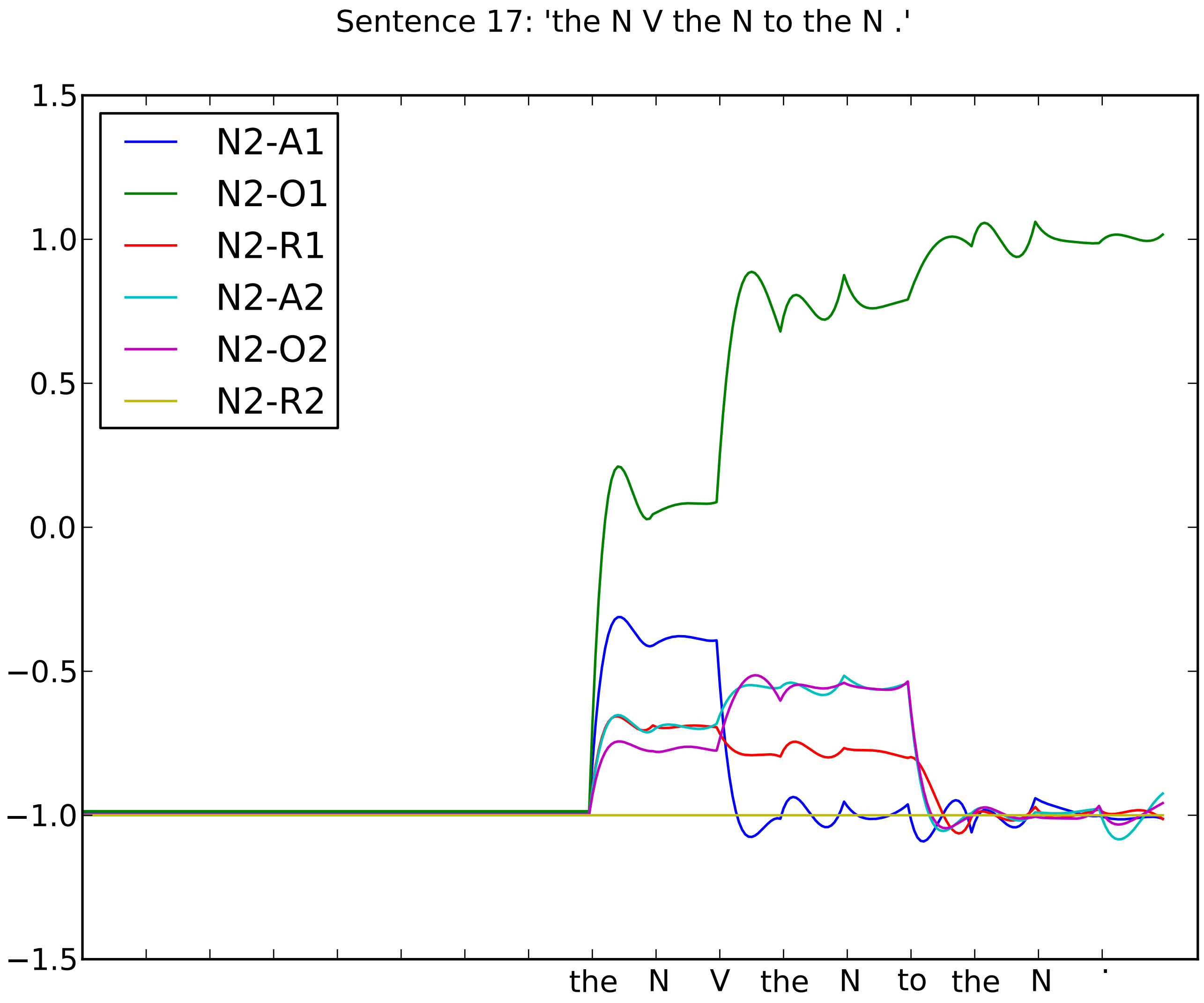

Sentence 17: 'the N V the N to the N .'
1.5
1.0
0.5
0.0
−0.5
−1.0
−1.5
N2-A1
N2-O1
N2-R1
N2-A2
N2-O2
N2-R2
the
N
V
the
N
to
the
N
.

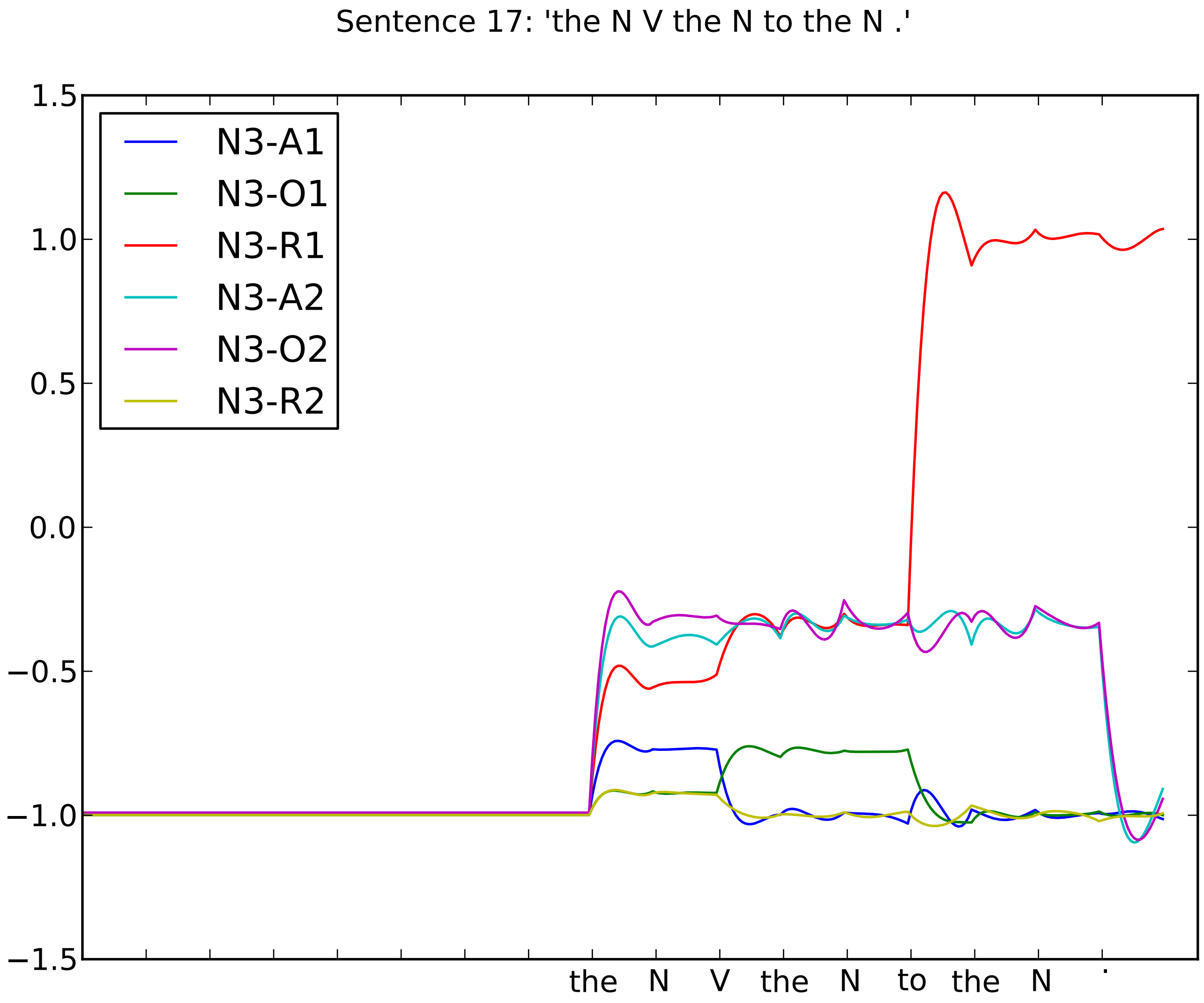

Sentence 17: 'the N V the N to the N .'
1.5
1.0
0.5
0.0
−0.5
−1.0
−1.5
N3-A1
N3-O1
N3-R1
N3-A2
N3-O2
N3-R2
the
N
V
the
N
to
the
N
.

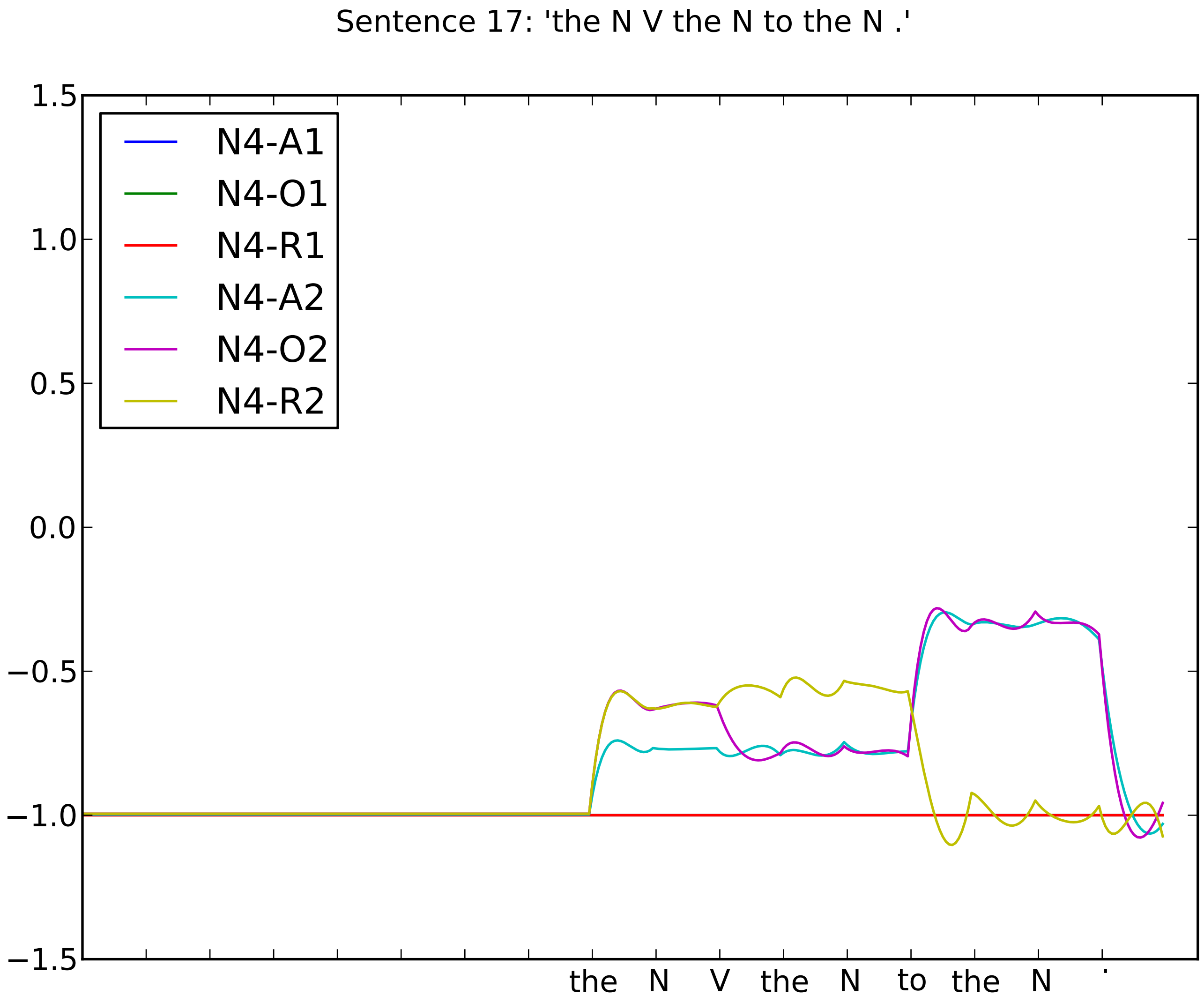
Sentence 17: 'the N V the N to the N .'
1.5
1.0
0.5
0.0
−0.5
−1.0
−1.5
N4-A1
N4-O1
N4-R1
N4-A2
N4-O2
N4-R2
the
N
V
the
N
to
the
N
.

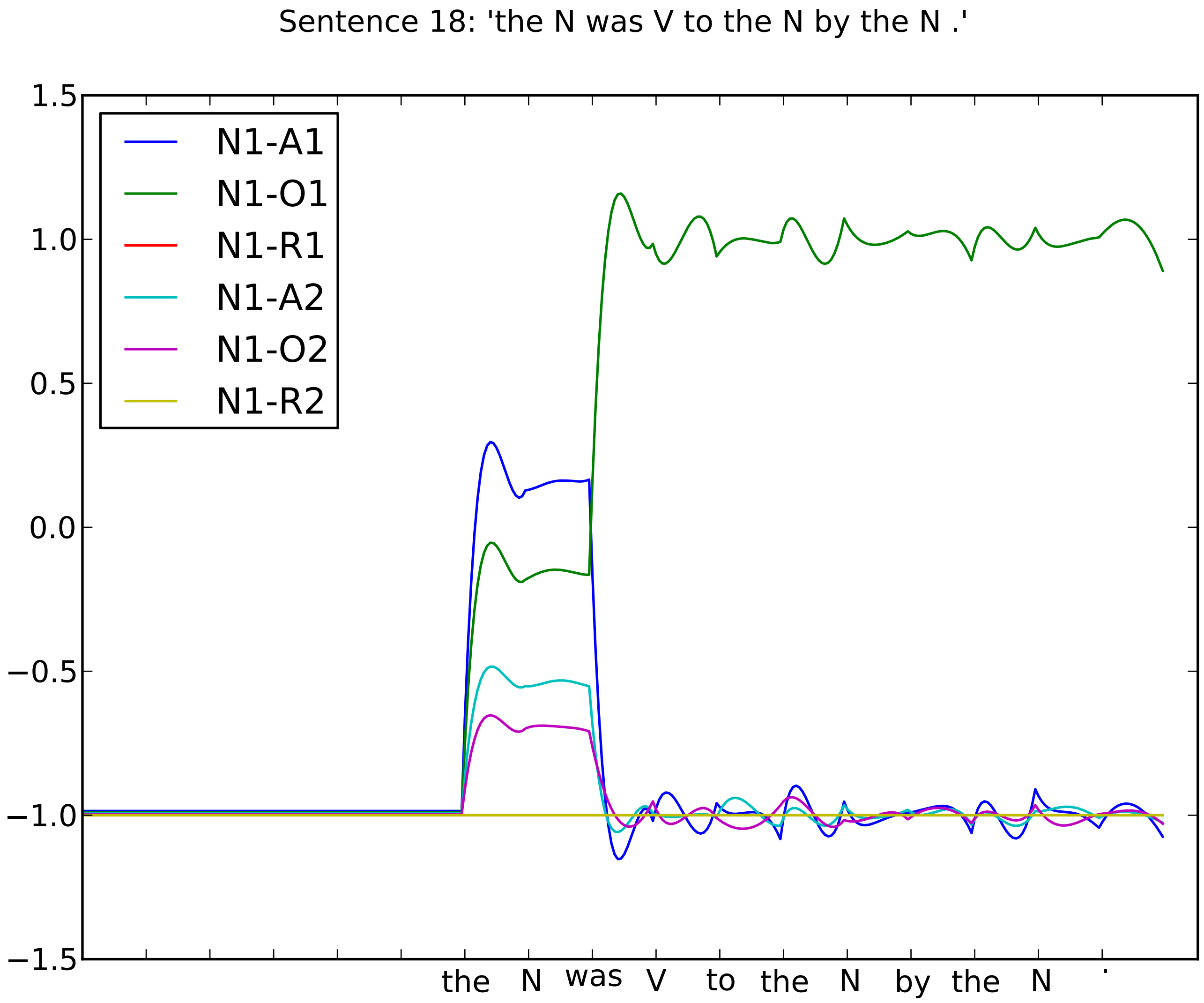

Sentence 18: 'the N was V to the N by the N .'
1.5
1.0
0.5
0.0
−0.5
−1.0
−1.5
N1-A1
N1-O1
N1-R1
N1-A2
N1-O2
N1-R2
the
N
was
V
to
the
N
by
the
N
.

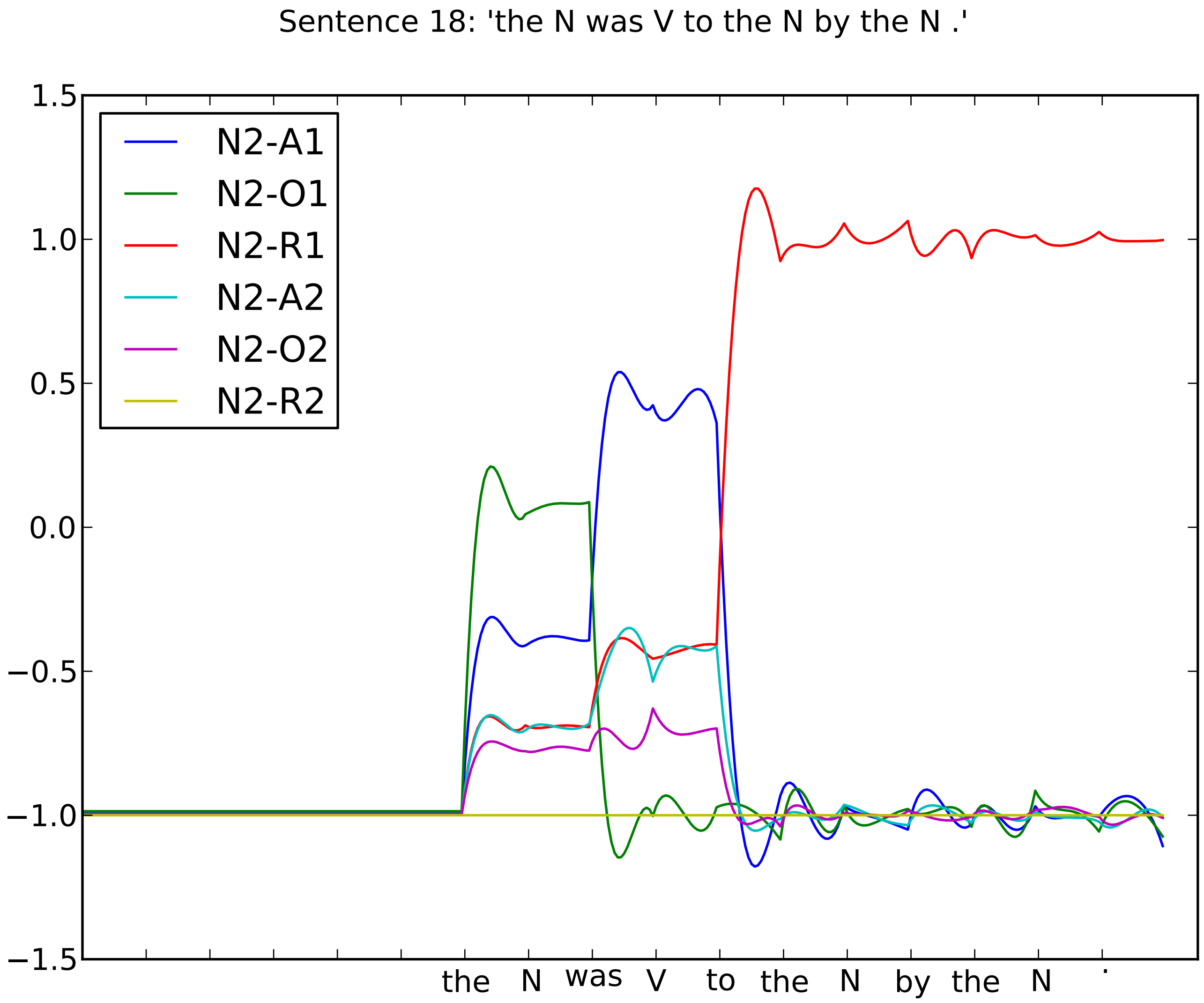
Sentence 18: 'the N was V to the N by the N .'
N2-A1
N2-O1
N2-R1
N2-A2
N2-O2
N2-R2
1.5
1.0
0.5
0.0
−0.5
−1.0
−1.5
the
N
was
V
to
the
N
by
the
N
.

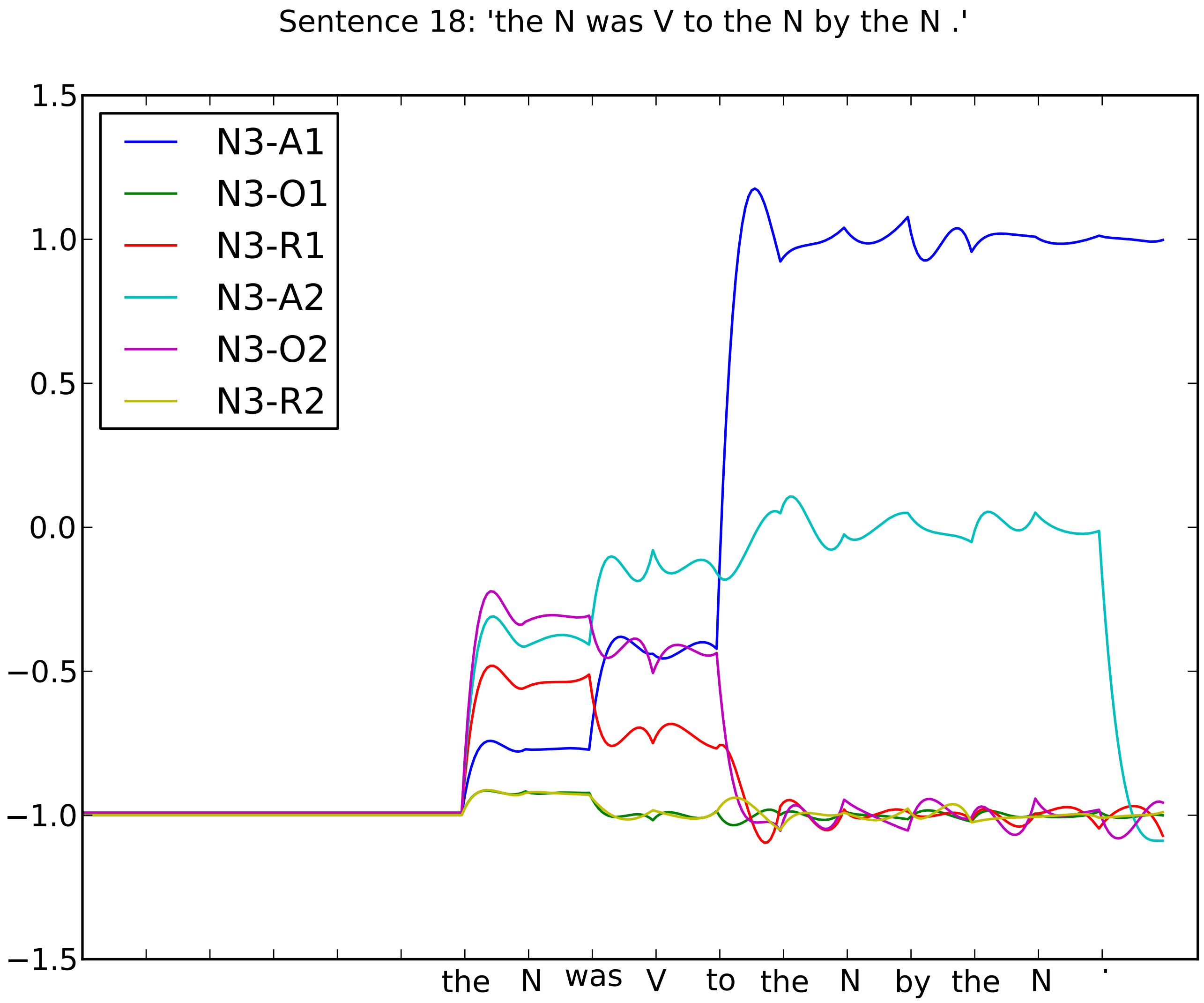

Sentence 18: 'the N was V to the N by the N .'
N3-A1
N3-O1
N3-R1
N3-A2
N3-O2
N3-R2
1.5
1.0
0.5
0.0
−0.5
−1.0
−1.5
the
N
was
V
to
the
N
by
the
N
.

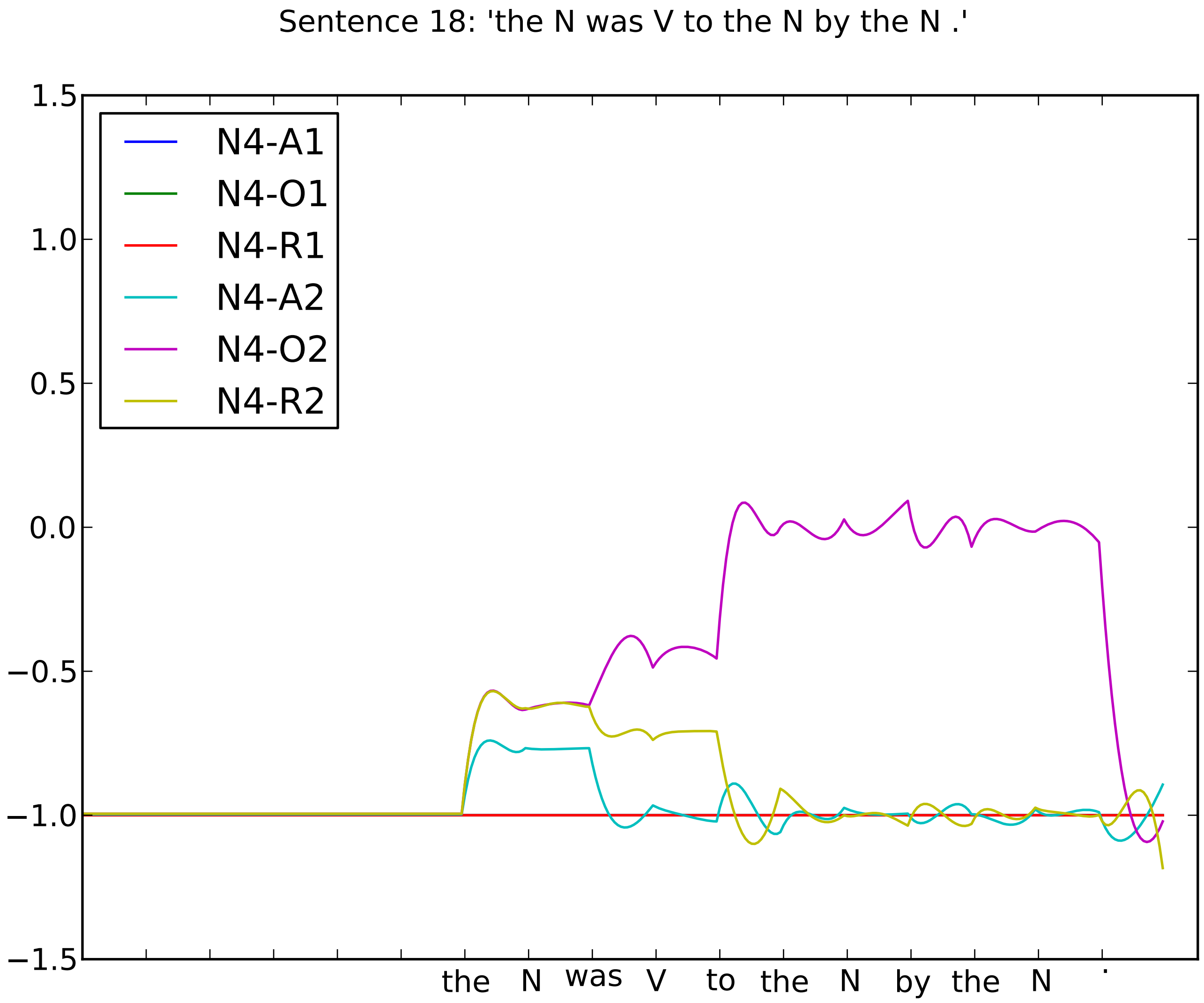

Sentence 18: 'the N was V to the N by the N .'
1.5
1.0
0.5
0.0
−0.5
−1.0
−1.5
N4-A1
N4-O1
N4-R1
N4-A2
N4-O2
N4-R2
the
N
was
V
to
the
N
by
the
N
.

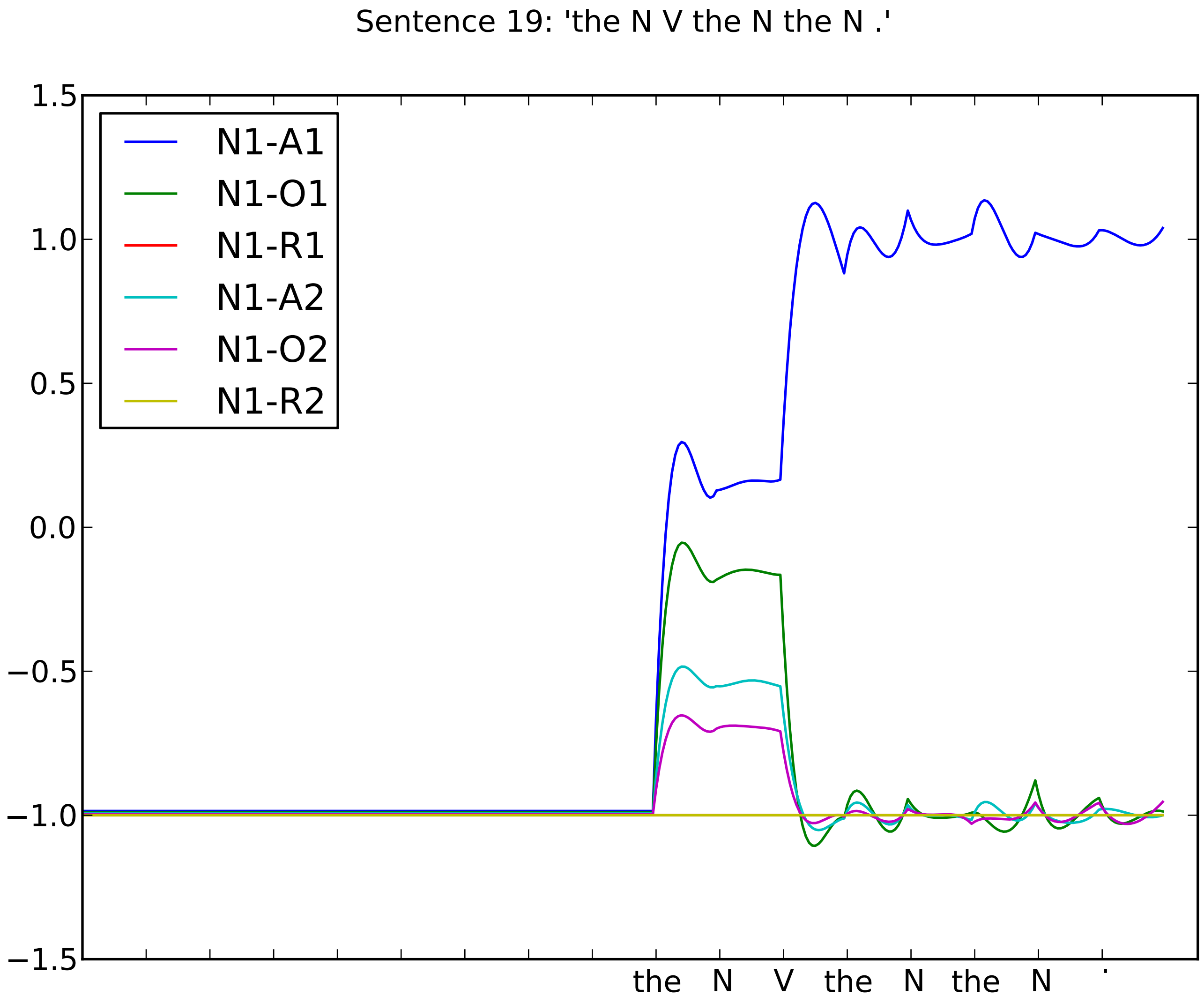
Sentence 19: 'the N V the N the N .'
1.5
1.0
0.5
0.0
−0.5
−1.0
−1.5
N1-A1
N1-O1
N1-R1
N1-A2
N1-O2
N1-R2
the
N
V
the
N
the
N
.

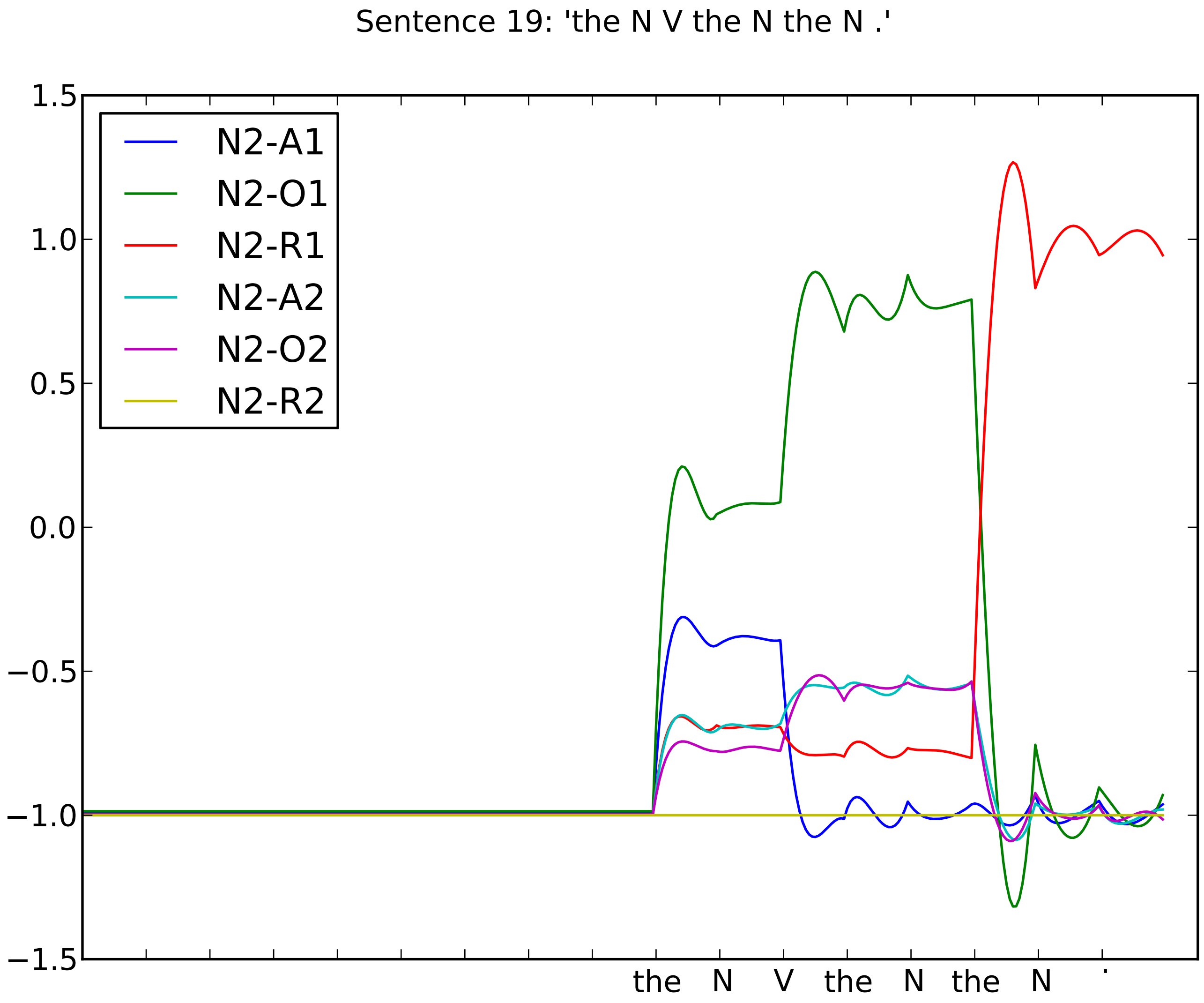
Sentence 19: 'the N V the N the N .'
1.5
1.0
0.5
0.0
−0.5
−1.0
−1.5
N2-A1
N2-O1
N2-R1
N2-A2
N2-O2
N2-R2
the
N
V
the
N
the
N
.

Sentence 19: 'the N V the N the N .'

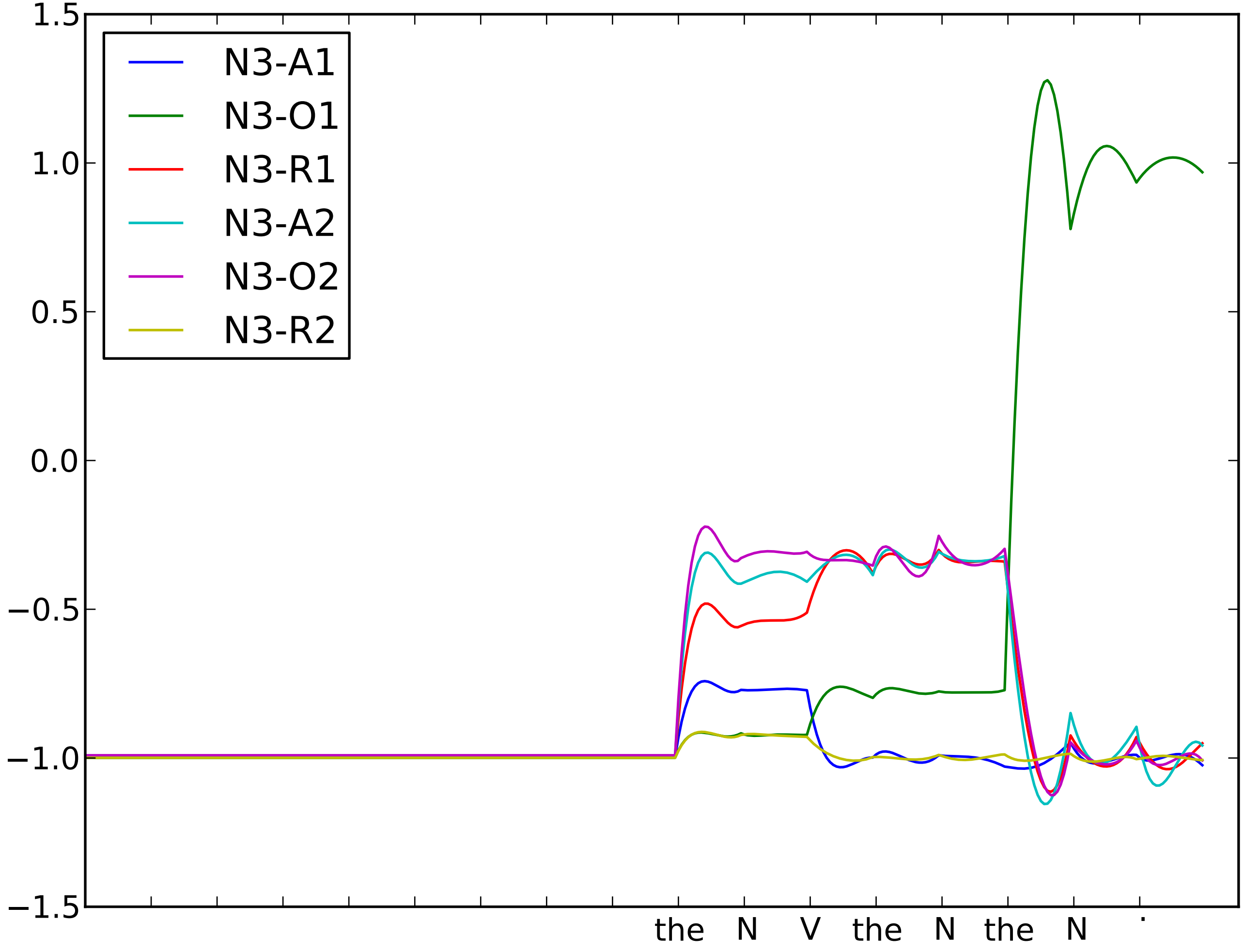

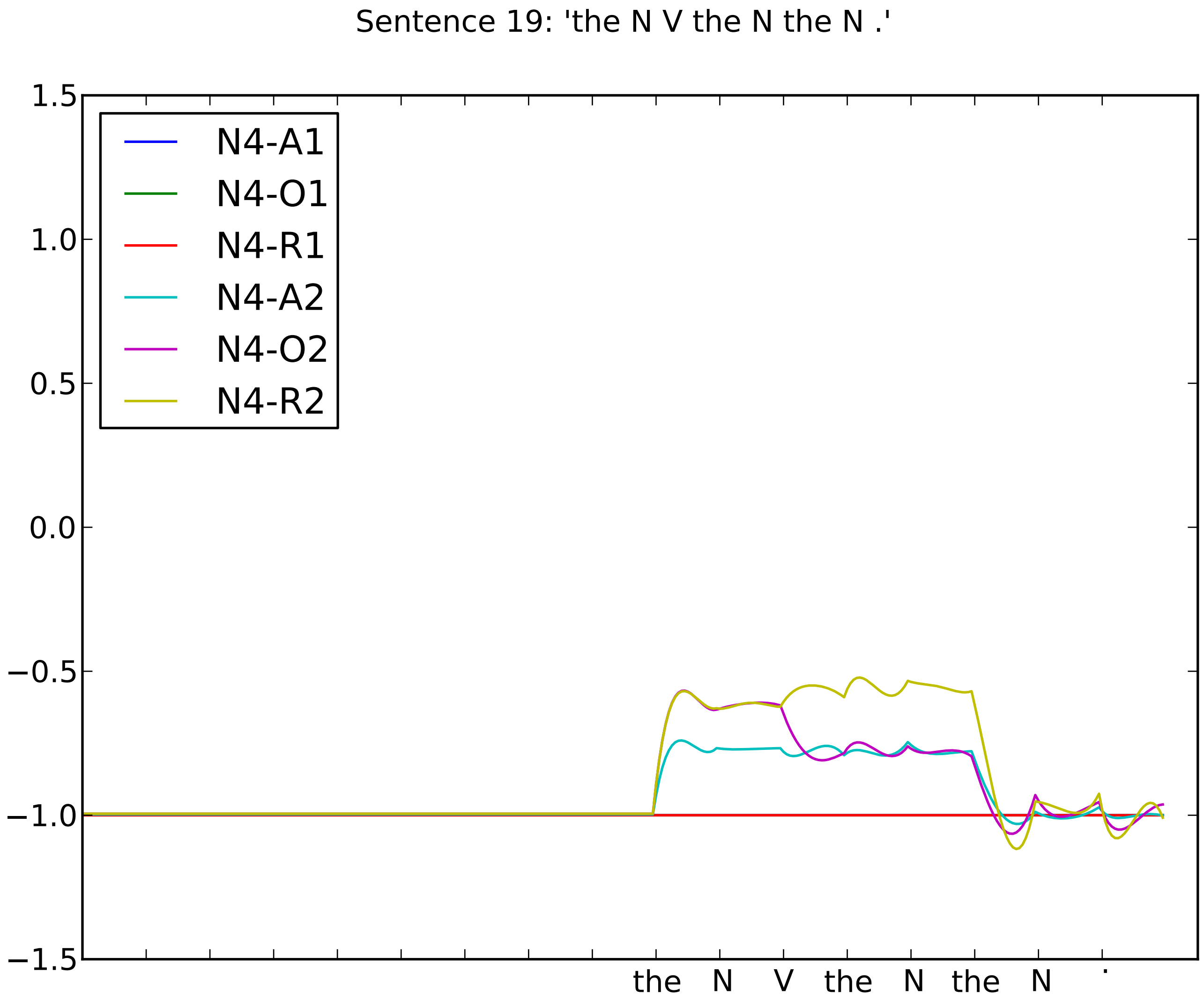

Sentence 19: 'the N V the N the N .'
1.5
1.0
0.5
0.0
−0.5
−1.0
−1.5
N4-A1
N4-O1
N4-R1
N4-A2
N4-O2
N4-R2
the
N
V
the
N
the
N
.

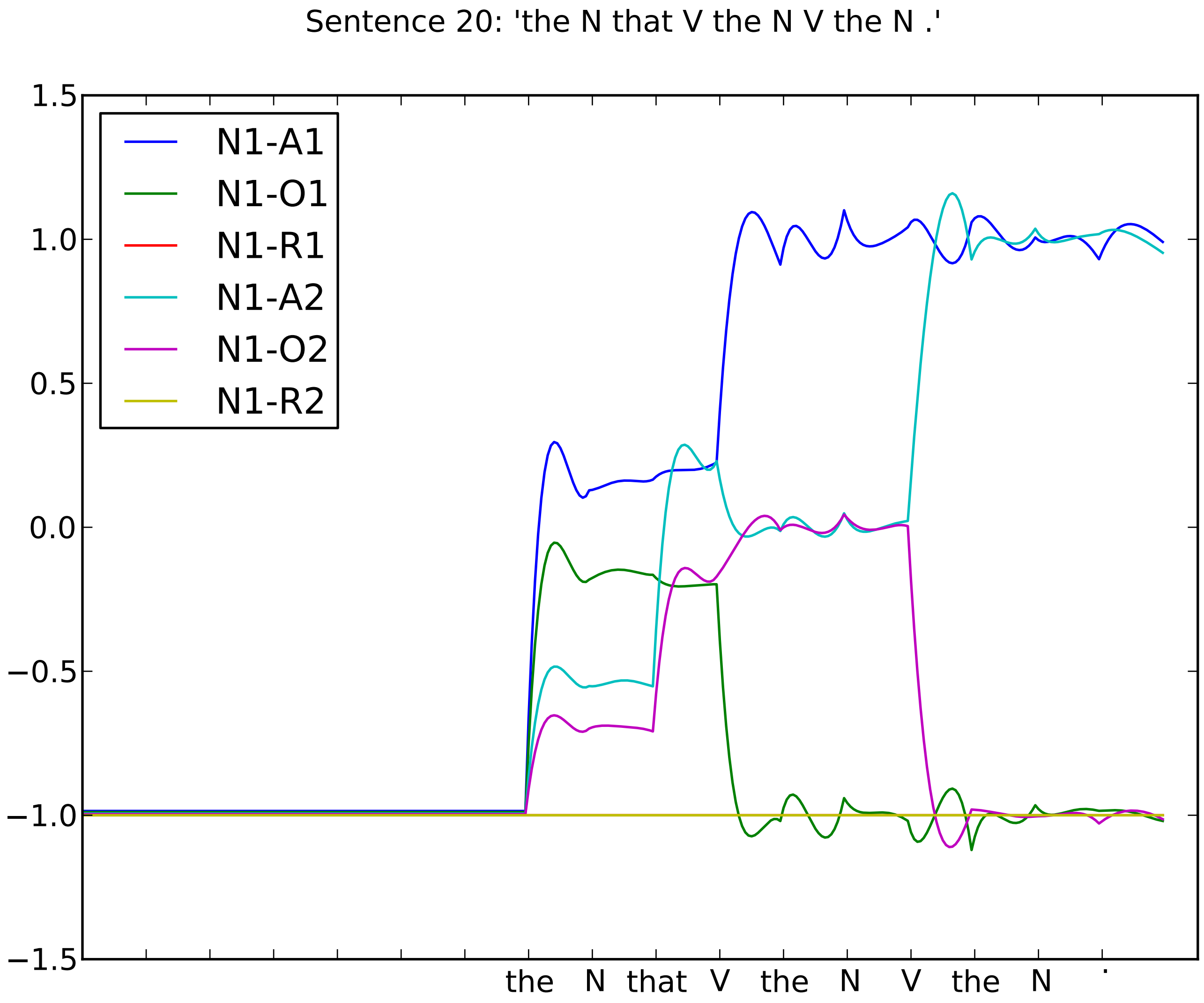
Sentence 20: 'the N that V the N V the N .'
1.5
1.0
0.5
0.0
−0.5
−1.0
−1.5
N1-A1
N1-O1
N1-R1
N1-A2
N1-O2
N1-R2
the
N
that
V
the
N
V
the
N
.

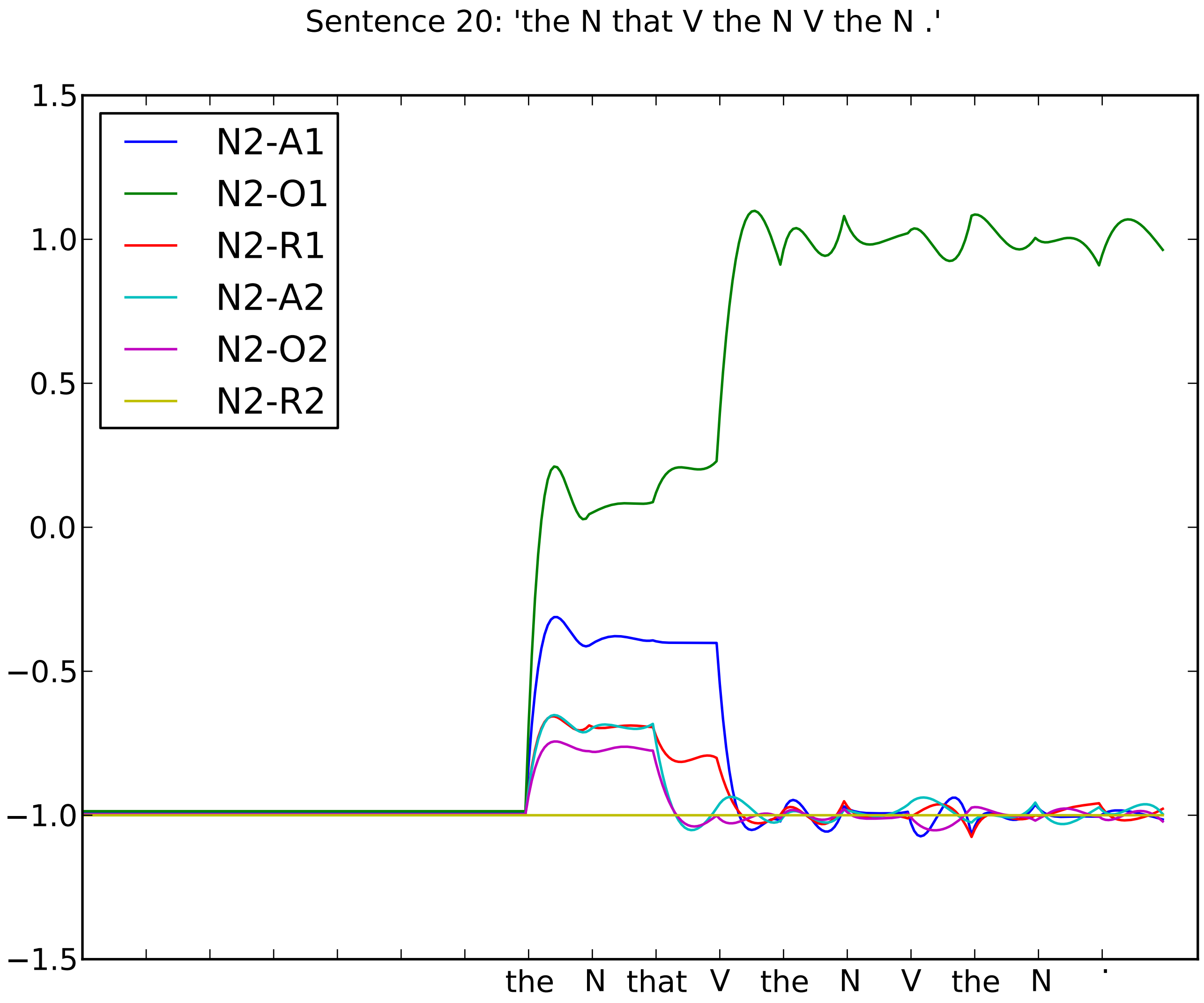

Sentence 20: 'the N that V the N V the N .'
1.5
1.0
0.5
0.0
−0.5
−1.0
−1.5
N2-A1
N2-O1
N2-R1
N2-A2
N2-O2
N2-R2
the N that V the N V the N .

Sentence 20: 'the N that V the N V the N .'

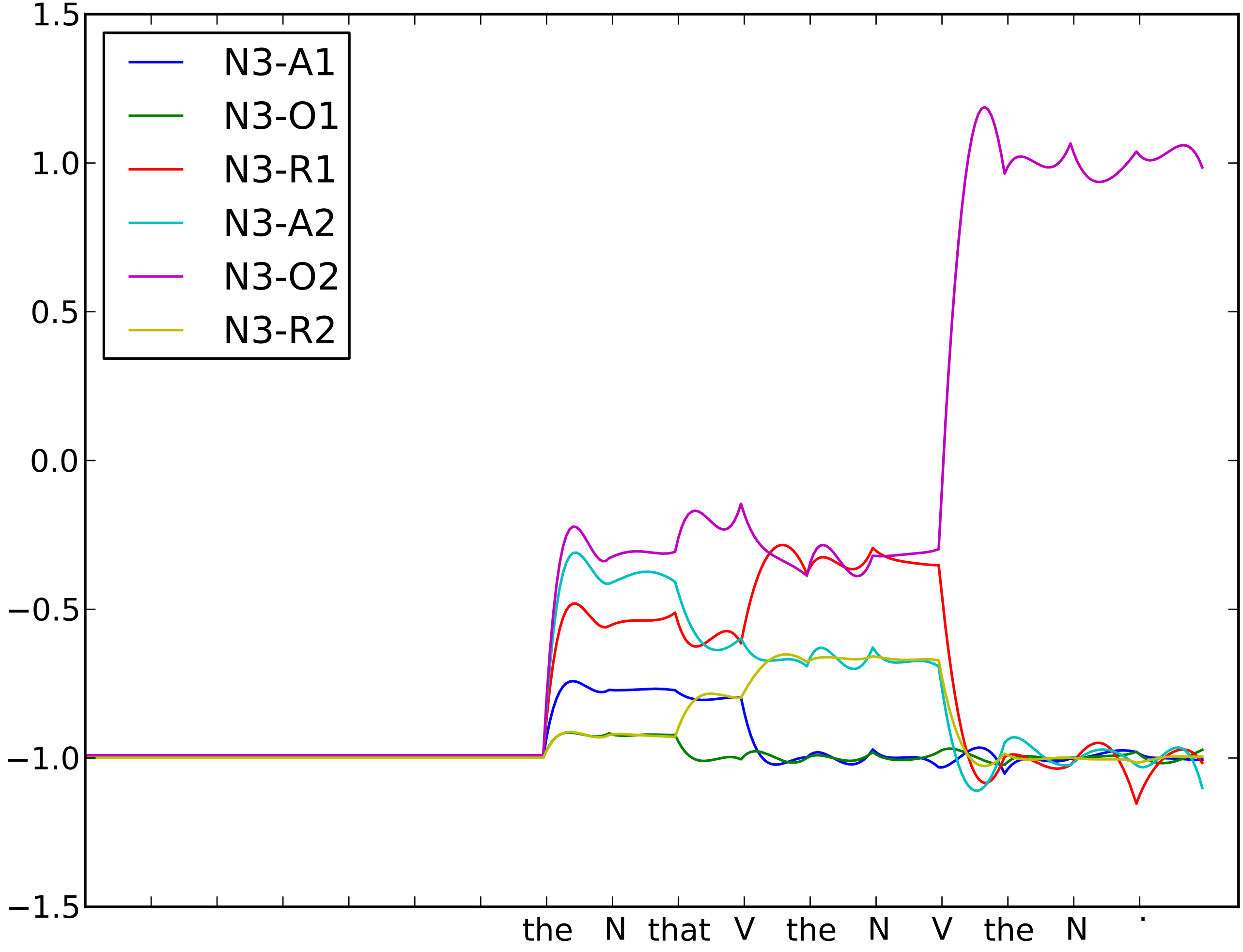

Sentence 20: 'the N that V the N V the N .'

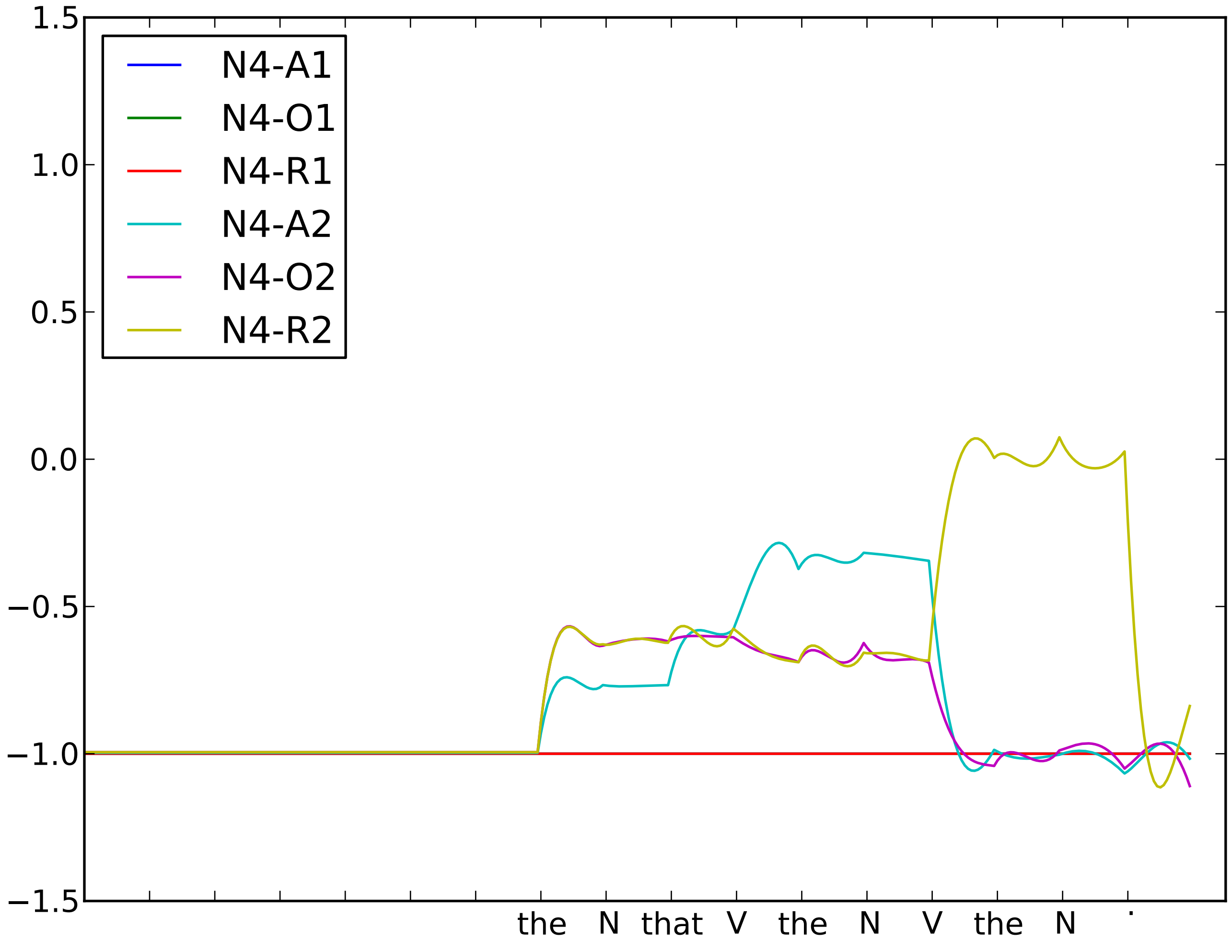

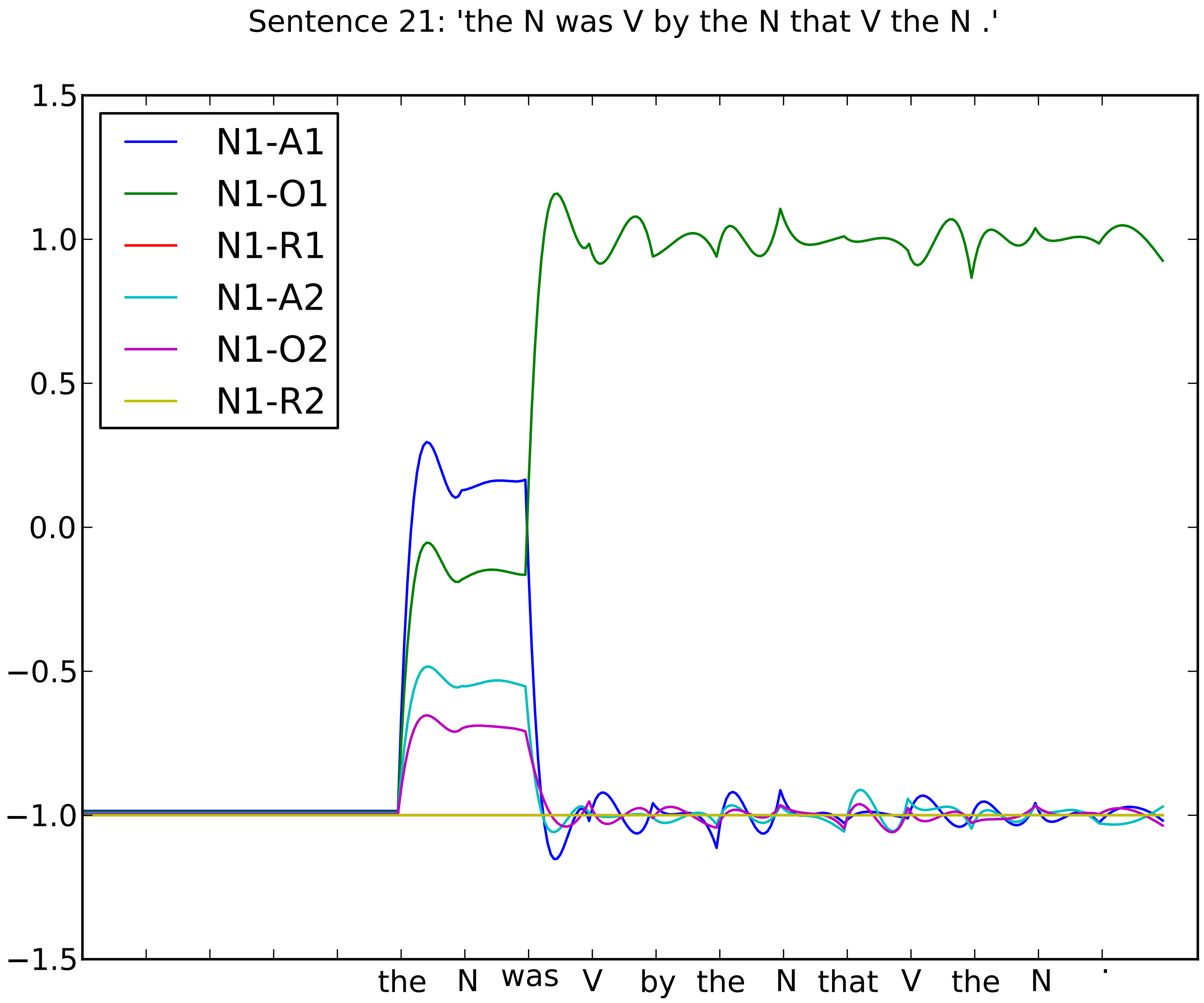

Sentence 21: 'the N was V by the N that V the N .'
1.5
1.0
0.5
0.0
−0.5
−1.0
−1.5
N1-A1
N1-O1
N1-R1
N1-A2
N1-O2
N1-R2
the N was V by the N that V the N .

Sentence 21: 'the N was V by the N that V the N .'

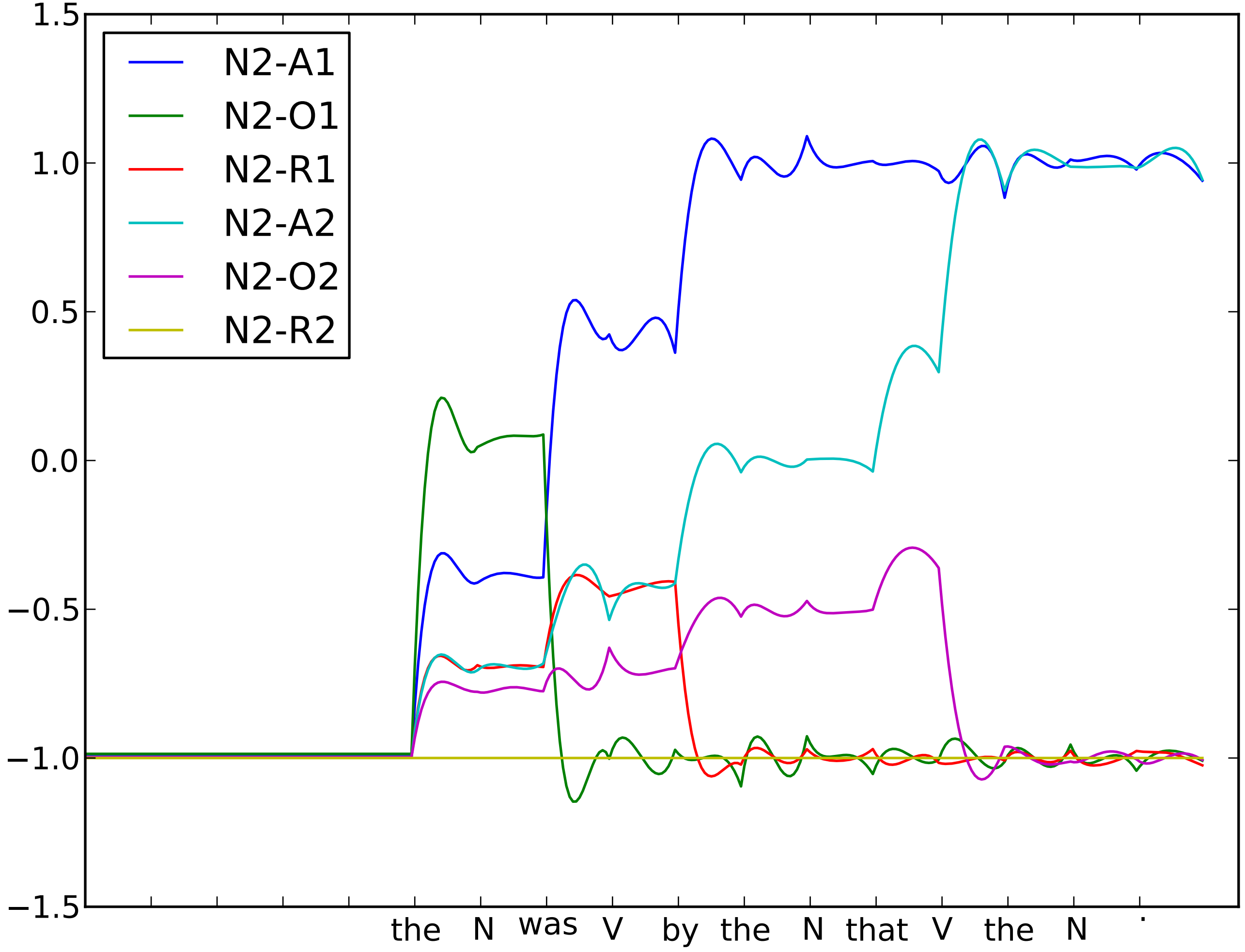

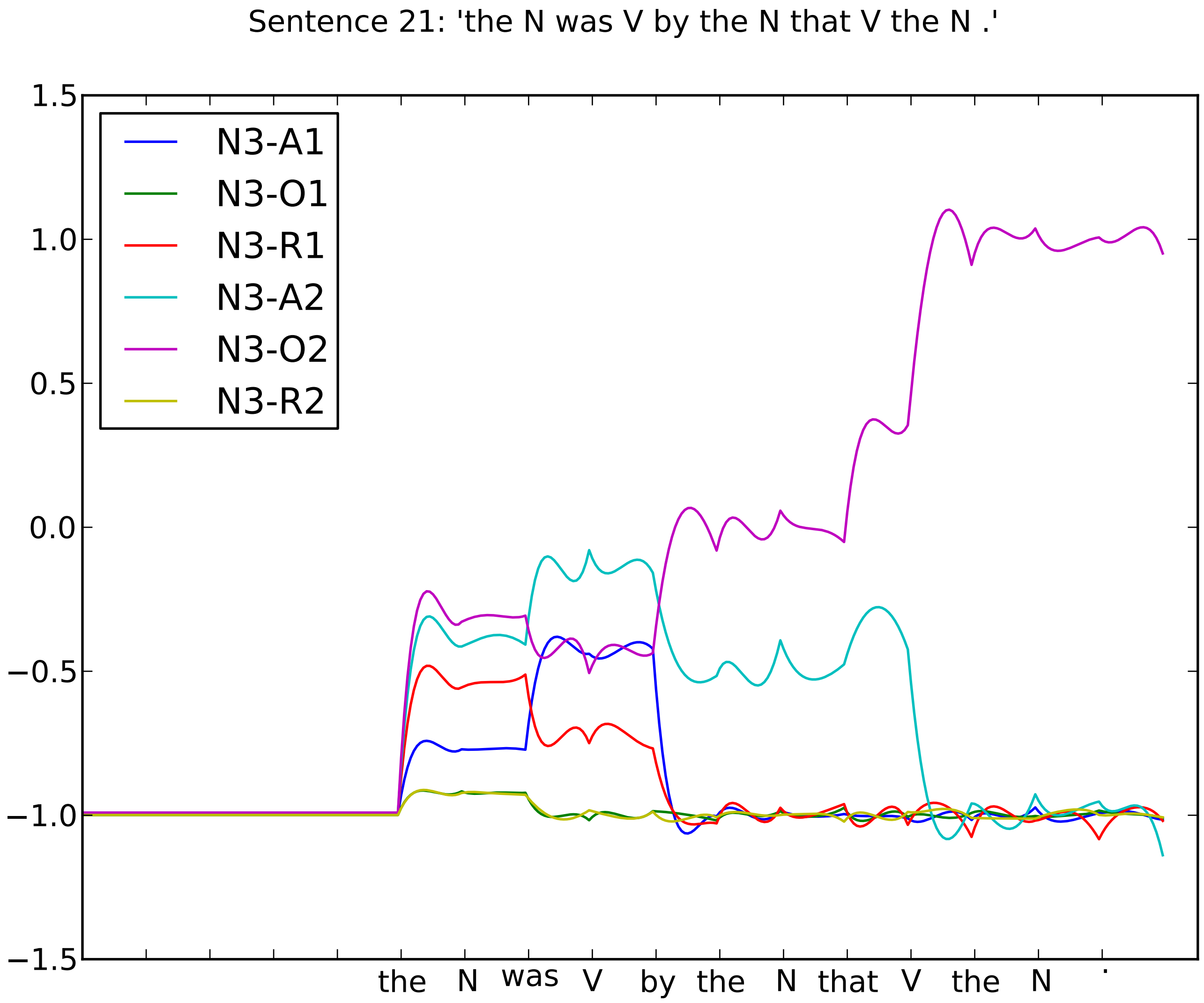

Sentence 21: 'the N was V by the N that V the N .'
1.5
1.0
0.5
0.0
−0.5
−1.0
−1.5
N3-A1
N3-O1
N3-R1
N3-A2
N3-O2
N3-R2
the
N
was
V
by
the
N
that
V
the
N
.

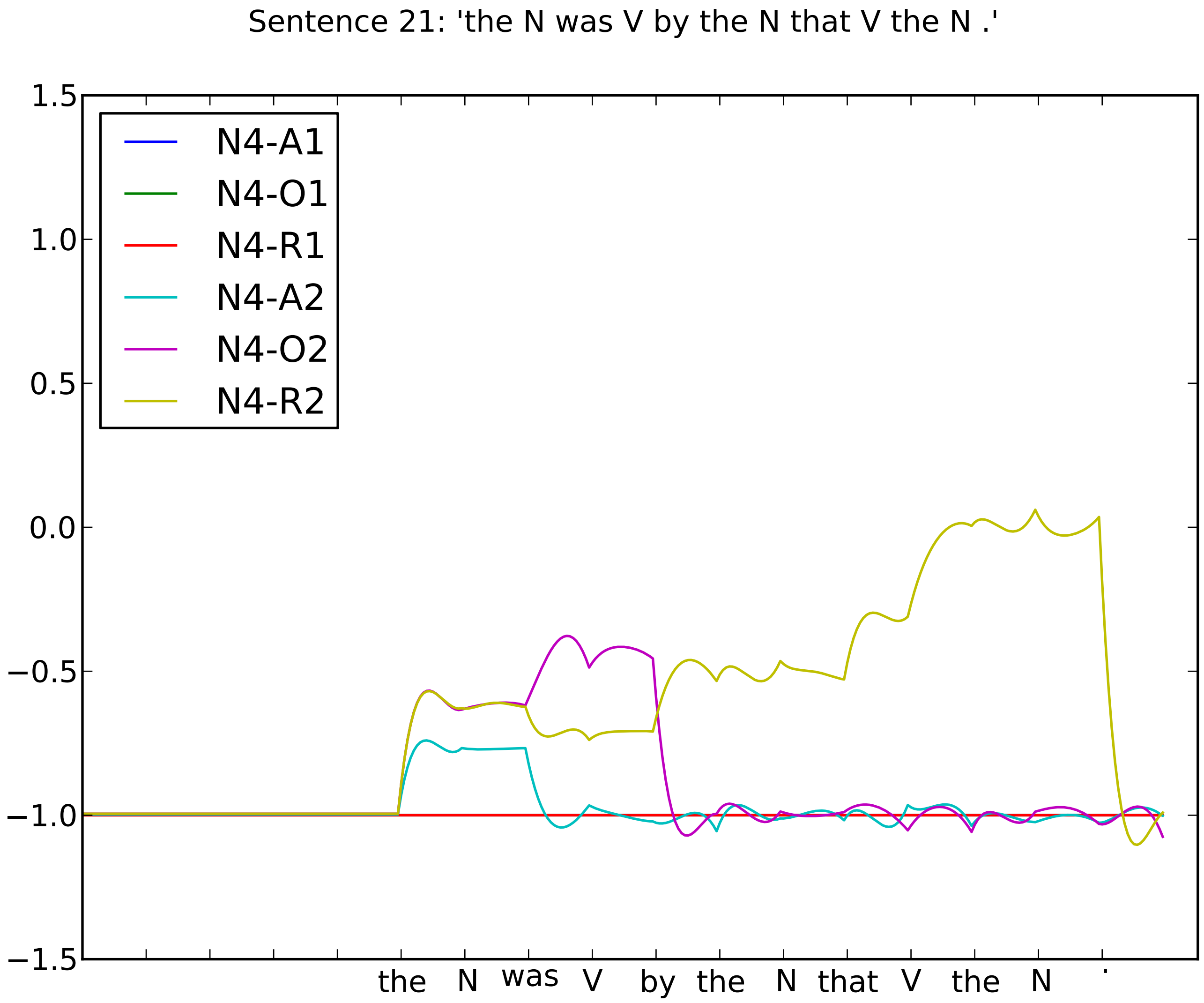
Sentence 21: 'the N was V by the N that V the N .'
1.5
1.0
0.5
0.0
−0.5
−1.0
−1.5
N4-A1
N4-O1
N4-R1
N4-A2
N4-O2
N4-R2
the
N
was
V
by
the
N
that
V
the
N
.

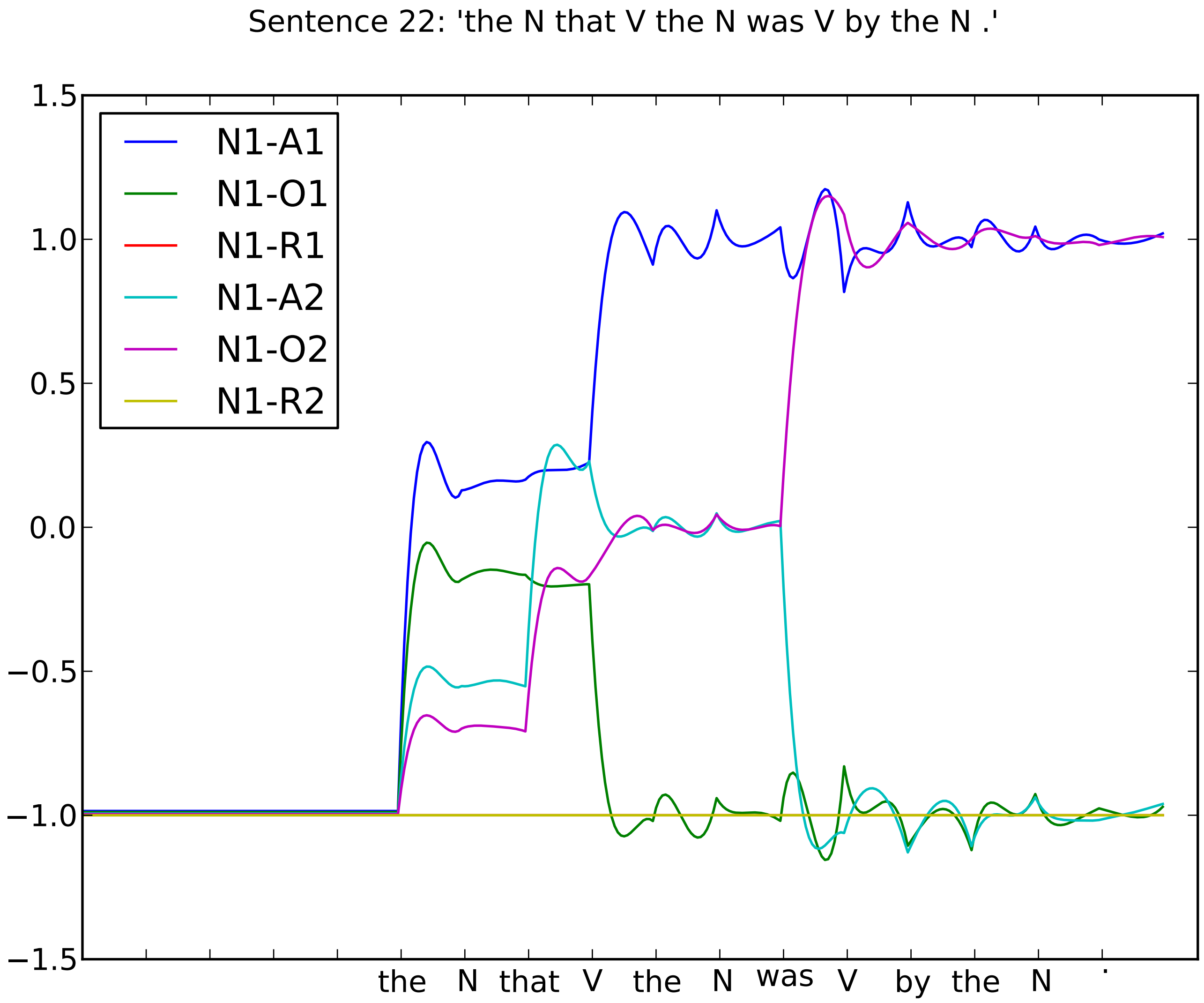
Sentence 22: 'the N that V the N was V by the N .'
N1-A1
N1-O1
N1-R1
N1-A2
N1-O2
N1-R2
1.5
1.0
0.5
0.0
−0.5
−1.0
−1.5
the N that V the N was V by the N .

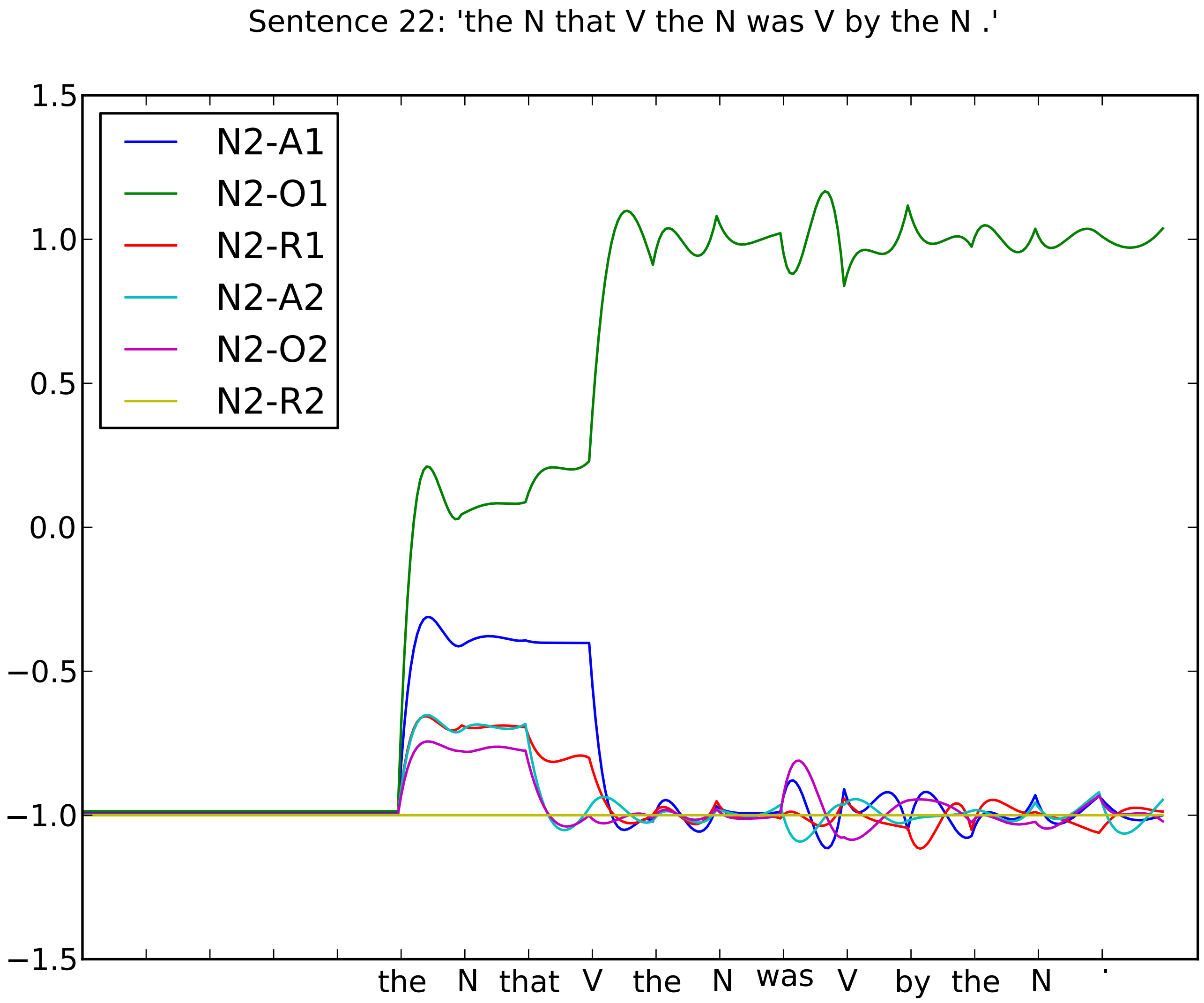
Sentence 22: 'the N that V the N was V by the N .'
N2-A1
N2-O1
N2-R1
N2-A2
N2-O2
N2-R2
1.5
1.0
0.5
0.0
−0.5
−1.0
−1.5
the
N
that
V
the
N
was
V
by
the
N
.

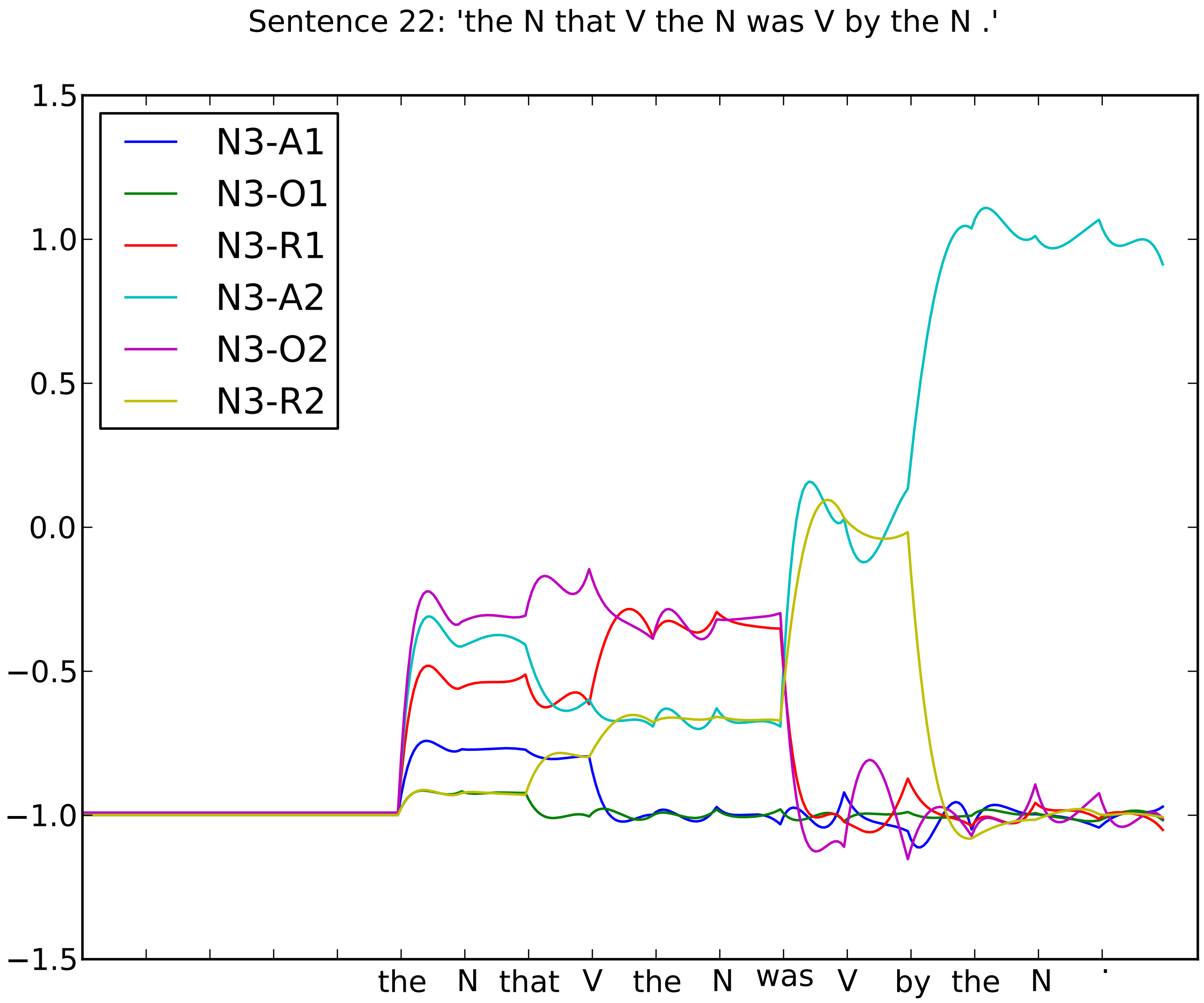

Sentence 22: 'the N that V the N was V by the N .'
1.5
1.0
0.5
0.0
−0.5
−1.0
−1.5
N3-A1
N3-O1
N3-R1
N3-A2
N3-O2
N3-R2
the N that V the N was V by the N .

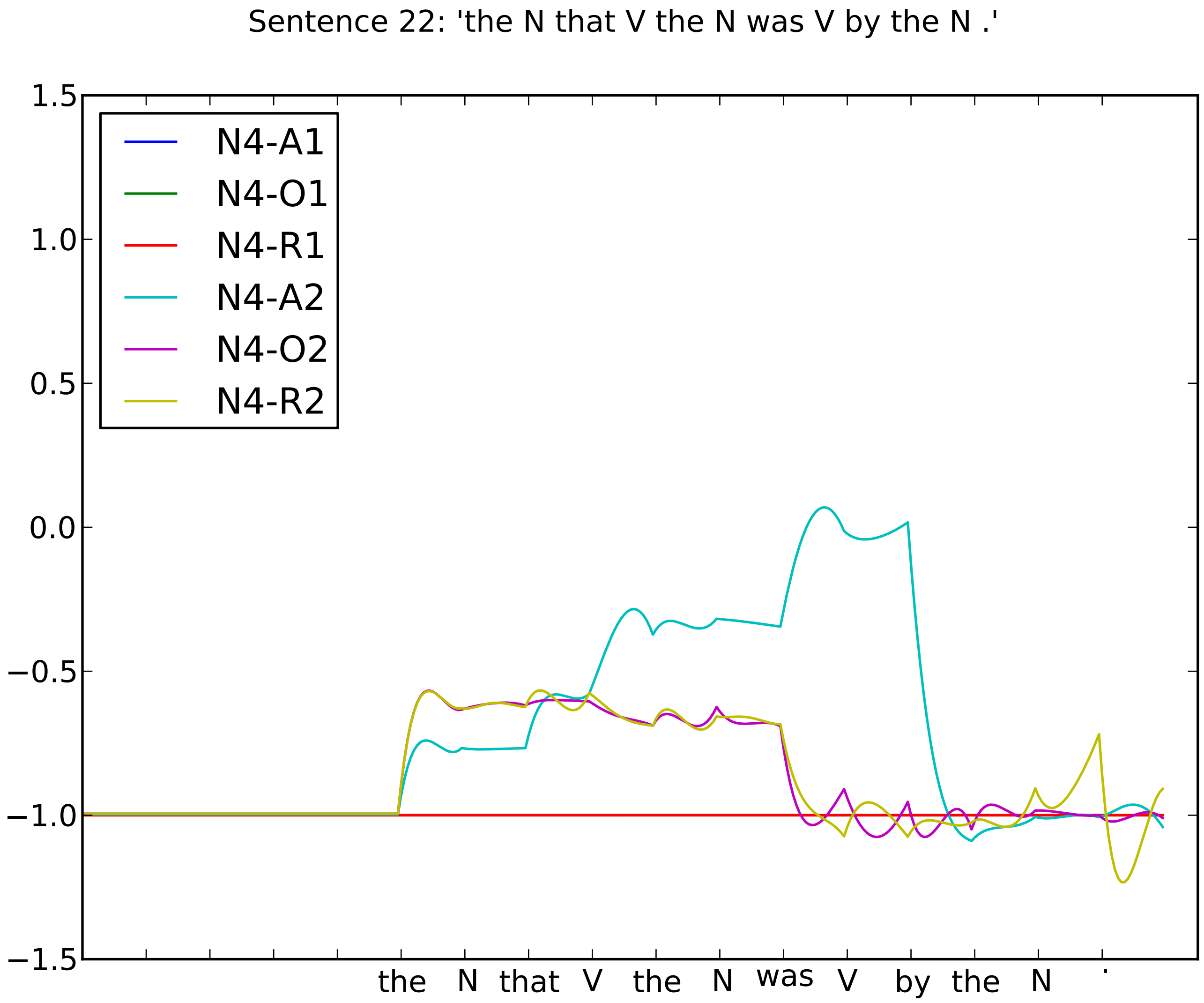
Sentence 22: 'the N that V the N was V by the N .'
N4-A1
N4-O1
N4-R1
N4-A2
N4-O2
N4-R2
1.5
1.0
0.5
0.0
−0.5
−1.0
−1.5
the
N
that
V
the
N
was
V
by
the
N
.

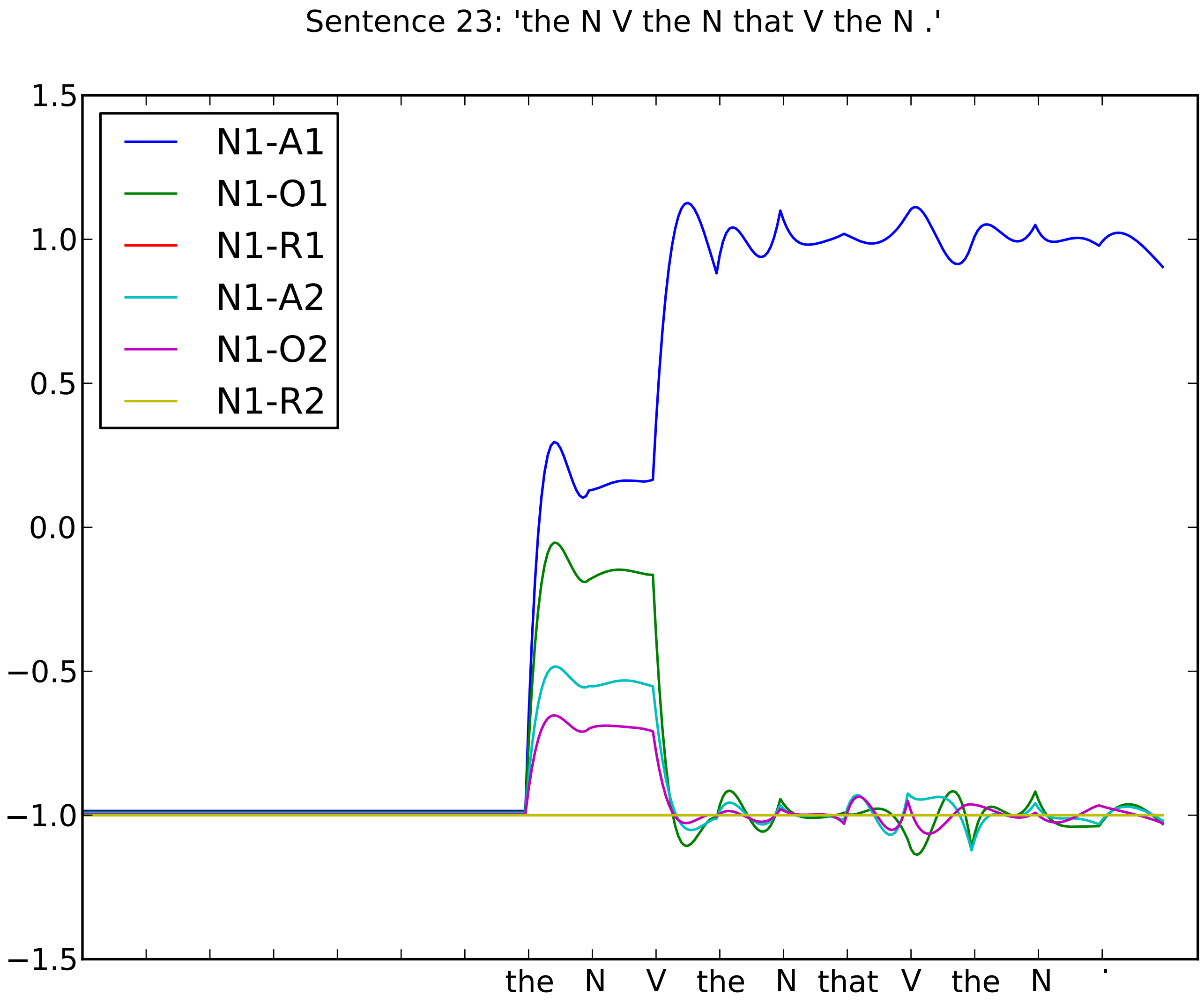
Sentence 23: 'the N V the N that V the N .'
1.5
1.0
0.5
0.0
−0.5
−1.0
−1.5
N1-A1
N1-O1
N1-R1
N1-A2
N1-O2
N1-R2
the
N
V
the
N
that
V
the
N
.

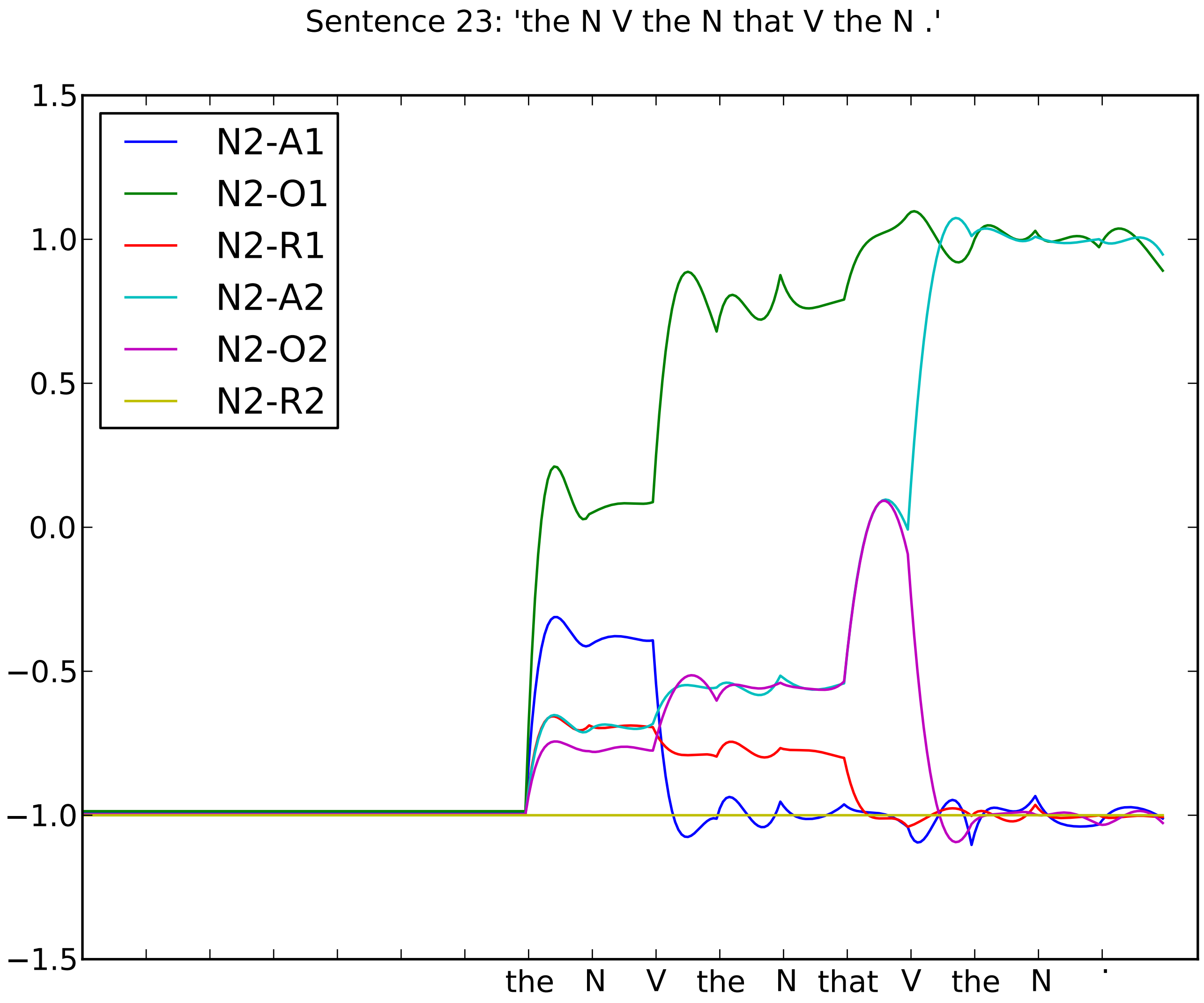
Sentence 23: 'the N V the N that V the N .'
N2-A1
N2-O1
N2-R1
N2-A2
N2-O2
N2-R2
1.5
1.0
0.5
0.0
−0.5
−1.0
−1.5
the
N
V
the
N
that
V
the
N
.

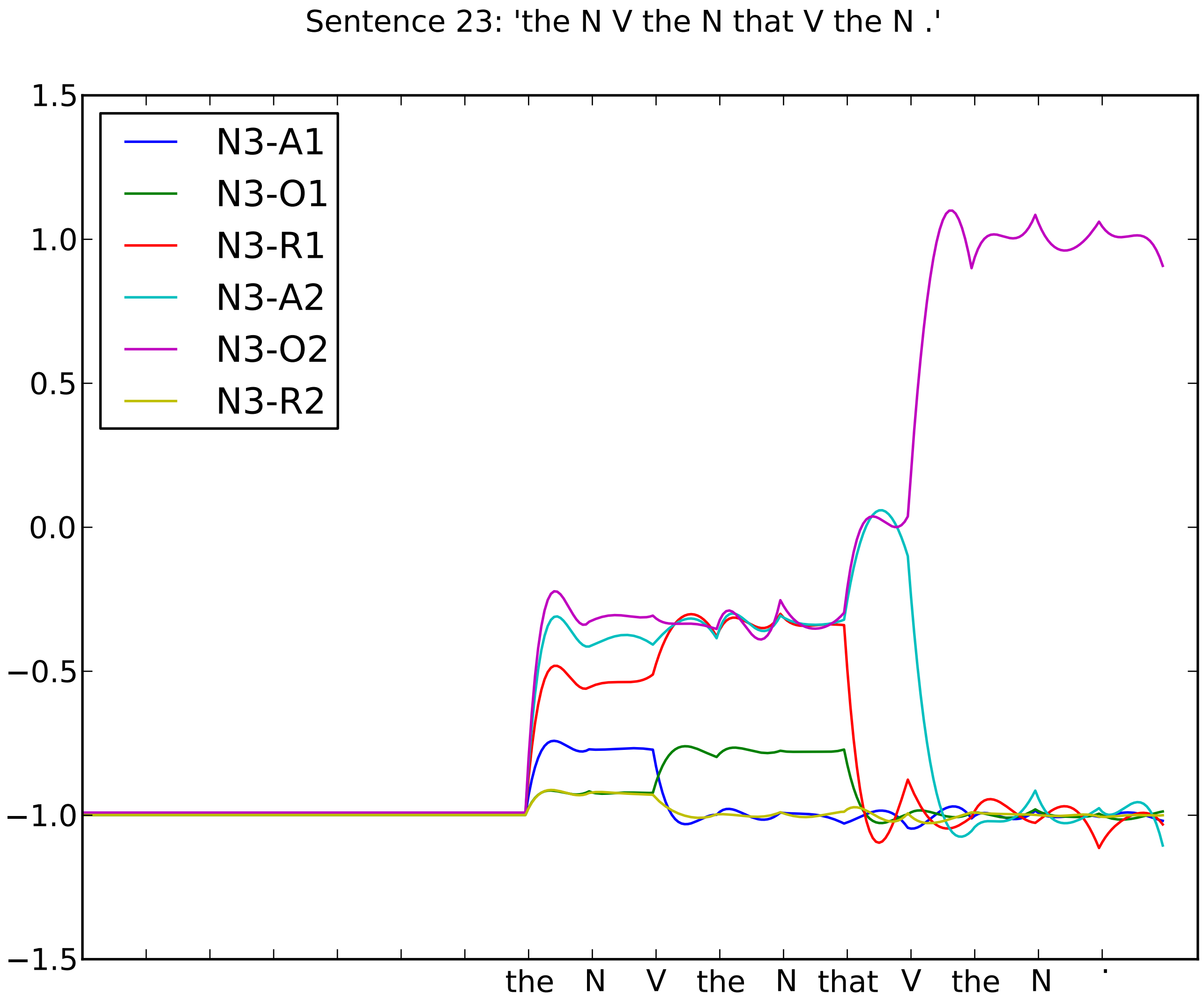

Sentence 23: 'the N V the N that V the N .'
1.5
1.0
0.5
0.0
−0.5
−1.0
−1.5
N3-A1
N3-O1
N3-R1
N3-A2
N3-O2
N3-R2
the
N
V
the
N
that
V
the
N
.

Sentence 23: 'the N V the N that V the N .'

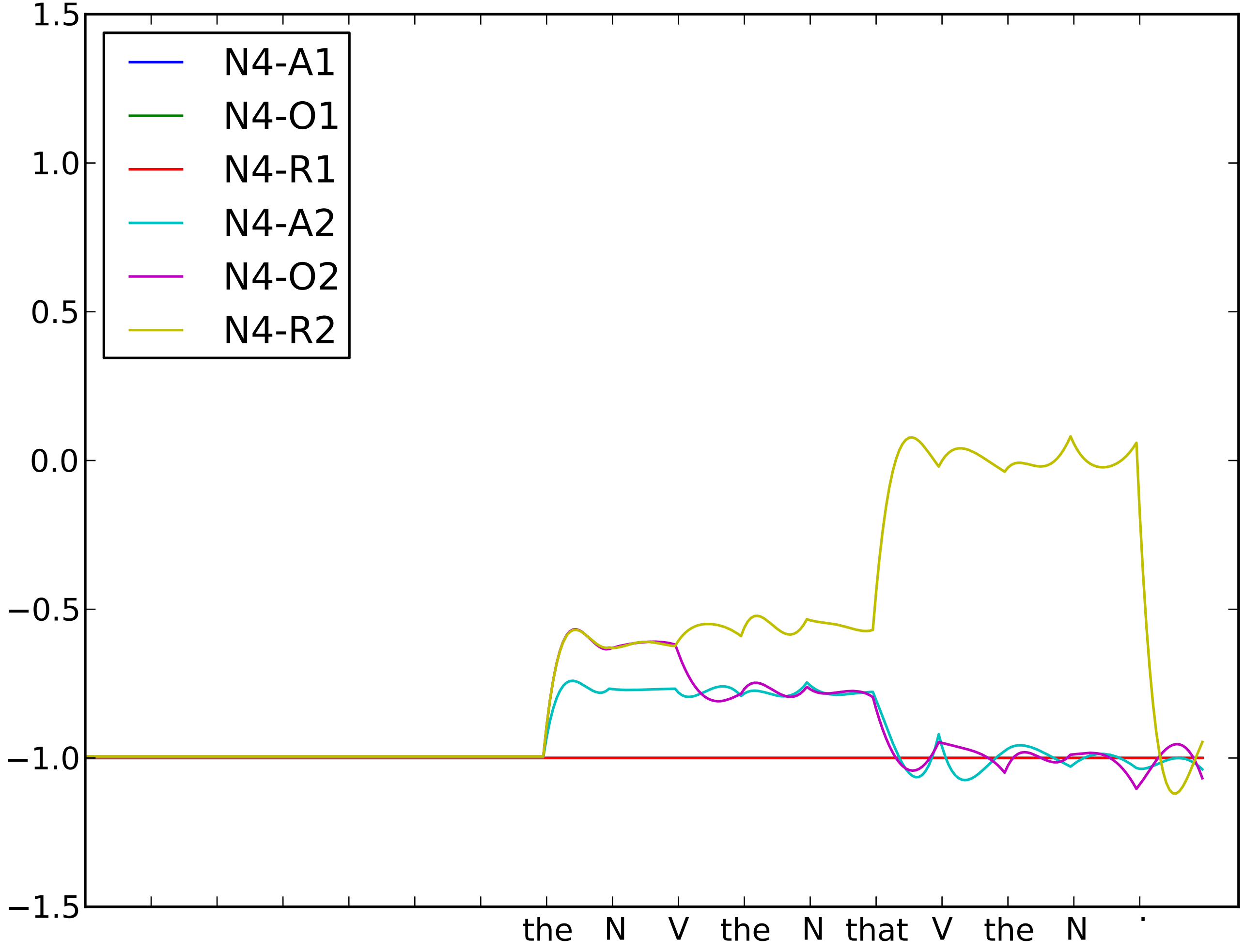

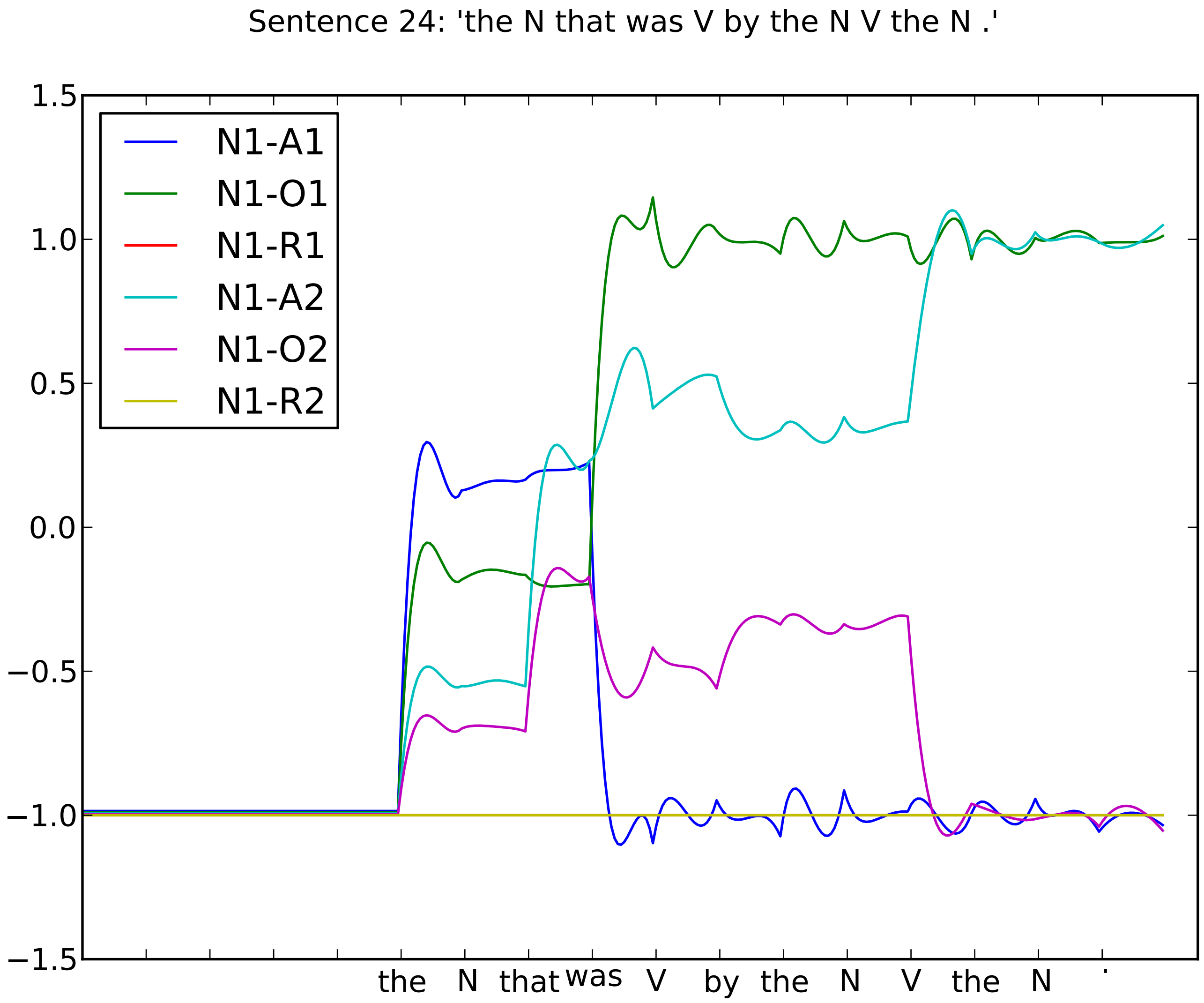

Sentence 24: 'the N that was V by the N V the N .'
1.5
1.0
0.5
0.0
−0.5
−1.0
−1.5
N1-A1
N1-O1
N1-R1
N1-A2
N1-O2
N1-R2
the
N
that
was
V
by
the
N
V
the
N
.

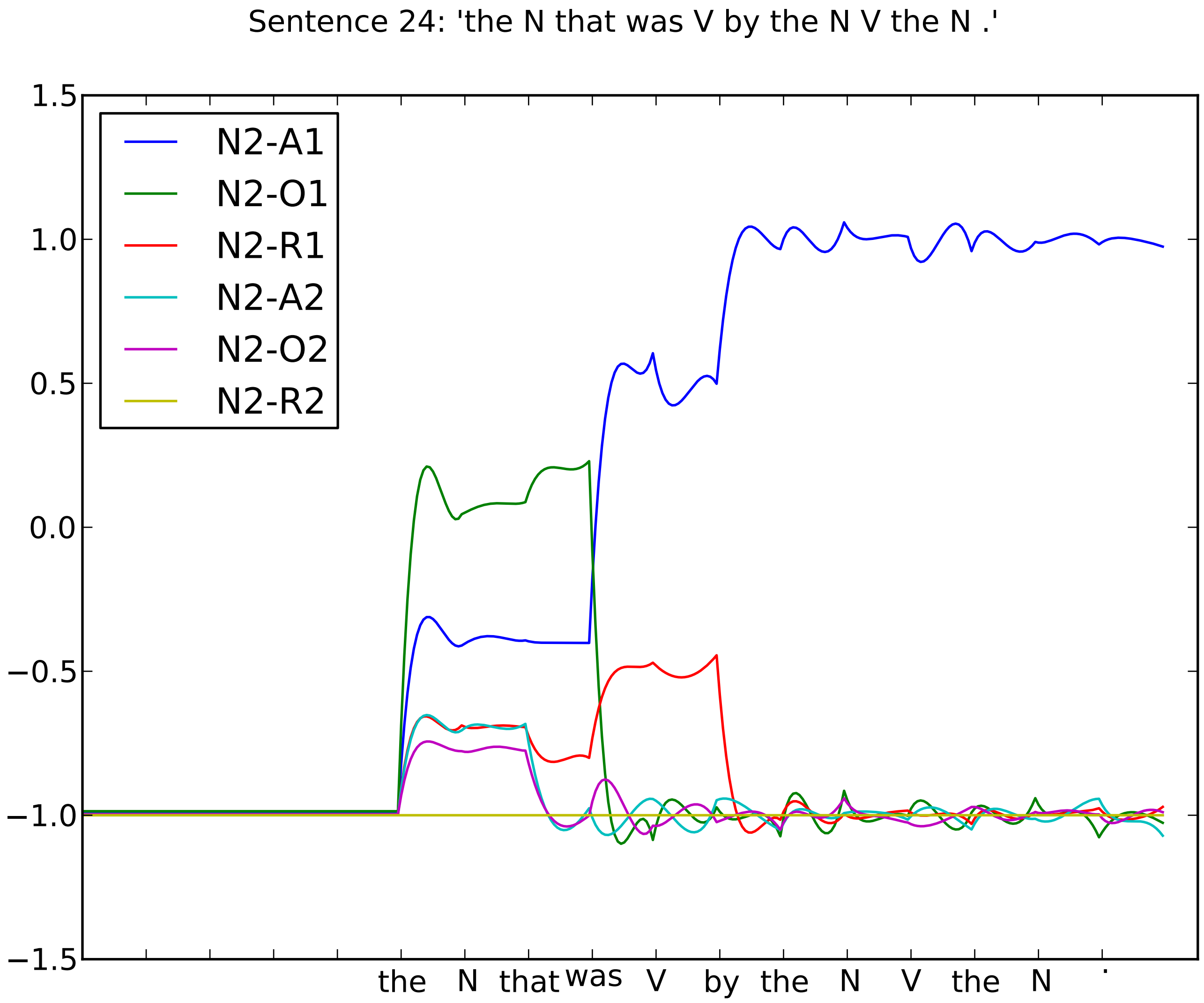

Sentence 24: 'the N that was V by the N V the N .'
1.5
1.0
0.5
0.0
−0.5
−1.0
−1.5
N2-A1
N2-O1
N2-R1
N2-A2
N2-O2
N2-R2
the
N
that
was
V
by
the
N
V
the
N
.

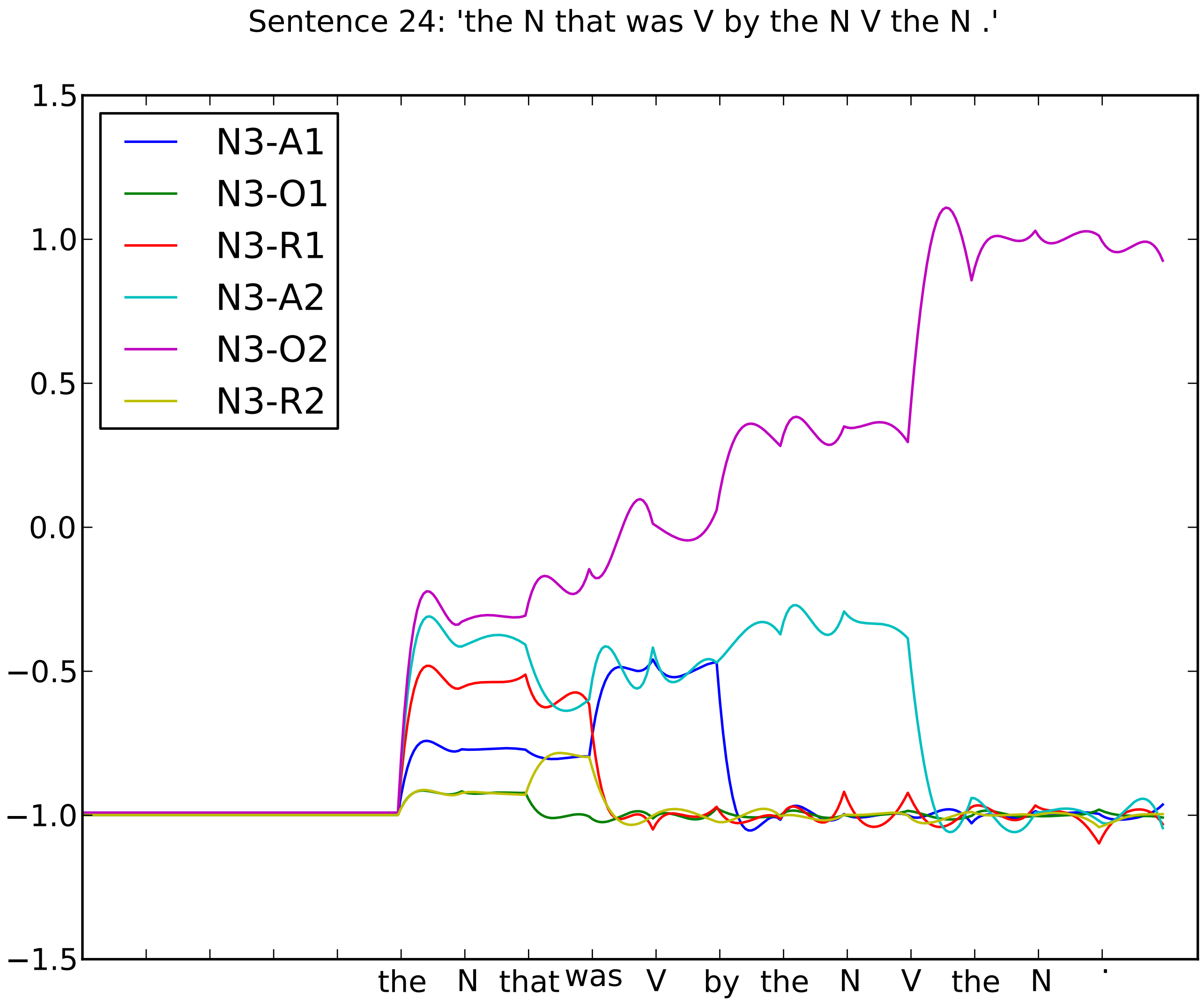
Sentence 24: 'the N that was V by the N V the N .'
1.5
1.0
0.5
0.0
−0.5
−1.0
−1.5
N3-A1
N3-O1
N3-R1
N3-A2
N3-O2
N3-R2
the
N
that
was
V
by
the
N
V
the
N
.

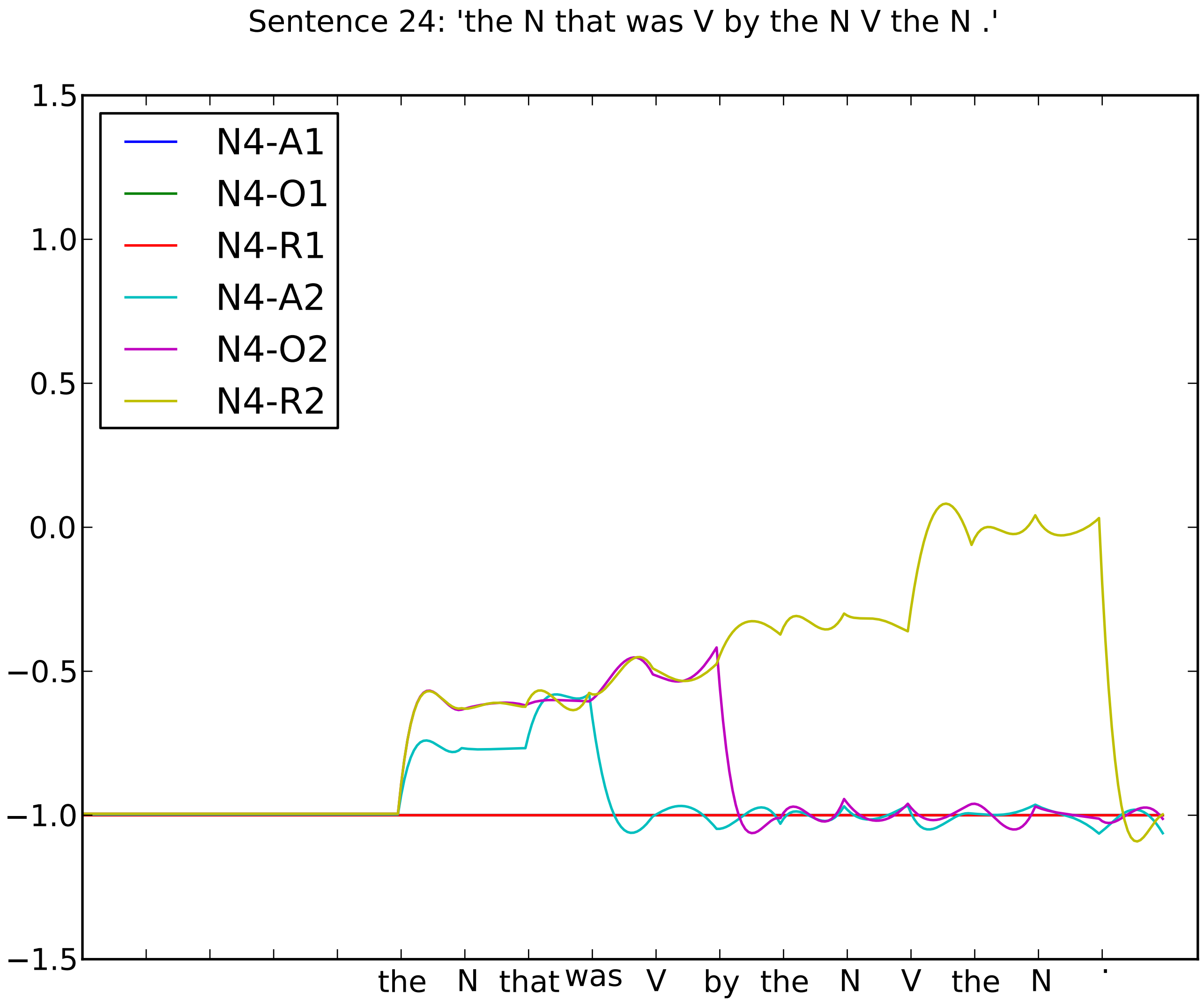

Sentence 24: 'the N that was V by the N V the N .'
1.5
1.0
0.5
0.0
−0.5
−1.0
−1.5
N4-A1
N4-O1
N4-R1
N4-A2
N4-O2
N4-R2
the
N
that
was
V
by
the
N
V
the
N
.

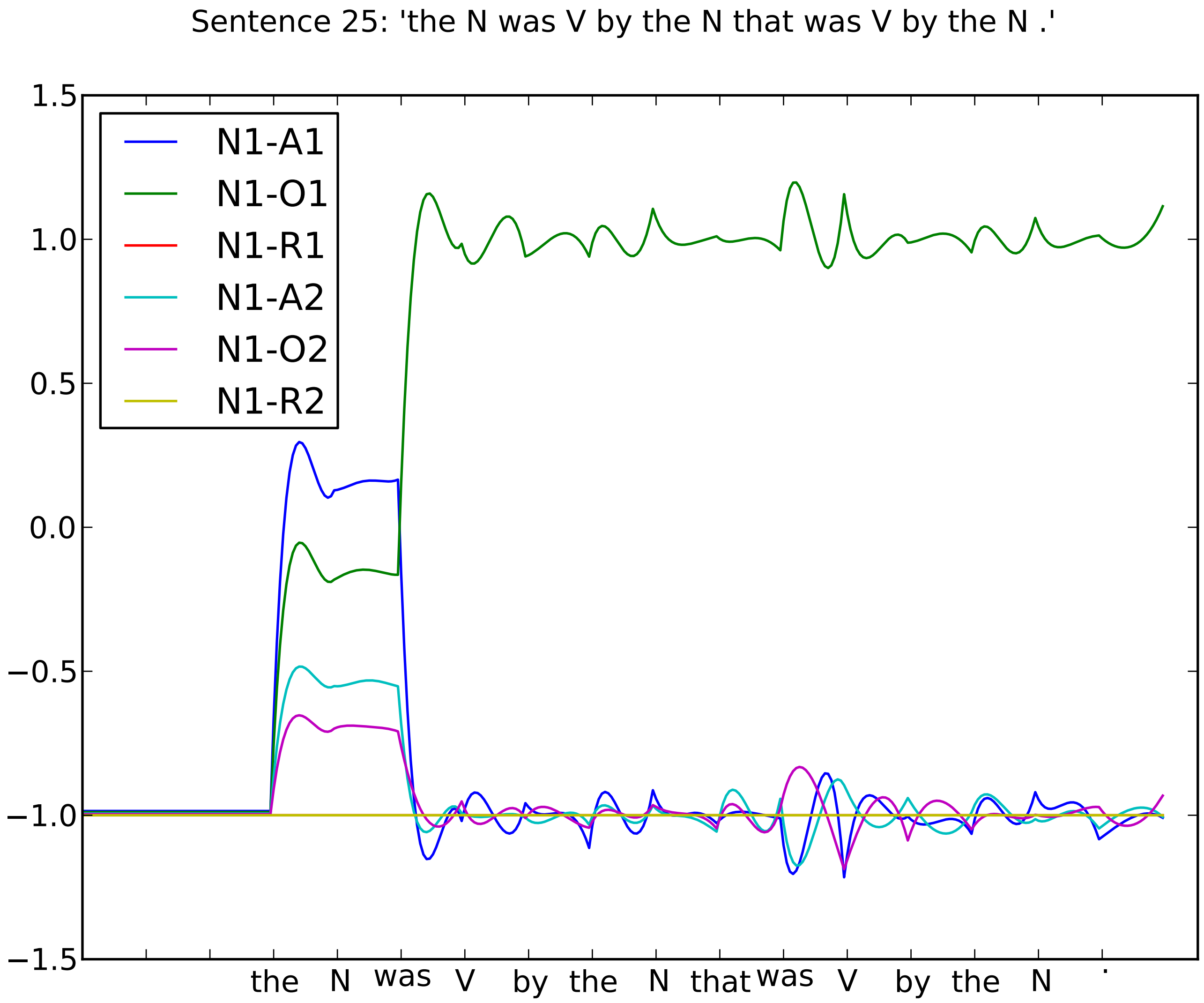
Sentence 25: 'the N was V by the N that was V by the N .'
N1-A1
N1-O1
N1-R1
N1-A2
N1-O2
N1-R2
1.5
1.0
0.5
0.0
−0.5
−1.0
−1.5
the N was V by the N that was V by the N .

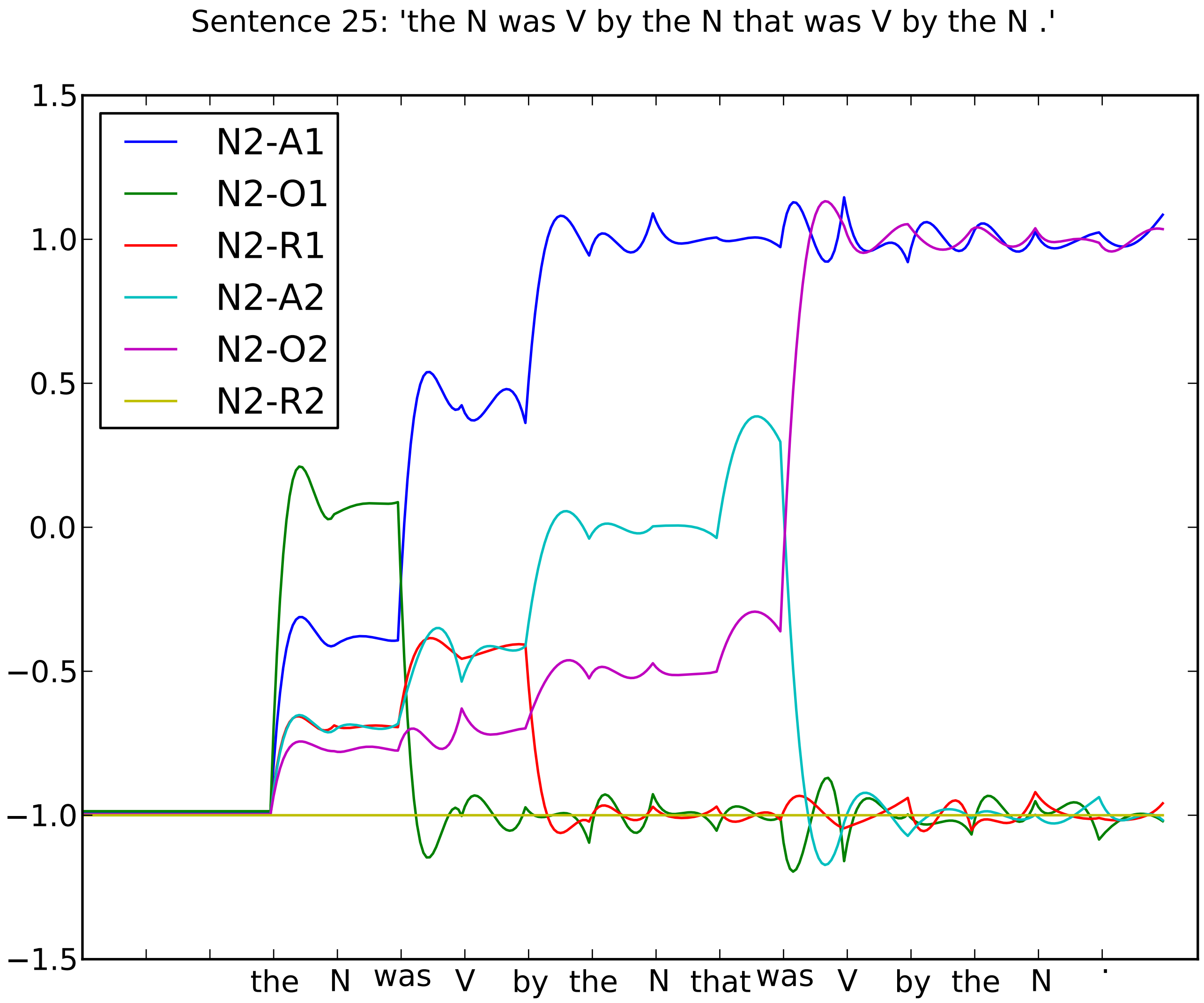

Sentence 25: 'the N was V by the N that was V by the N .'
N2-A1
N2-O1
N2-R1
N2-A2
N2-O2
N2-R2
1.5
1.0
0.5
0.0
−0.5
−1.0
−1.5
the
N
was
V
by
the
N
that
was
V
by
the
N
.

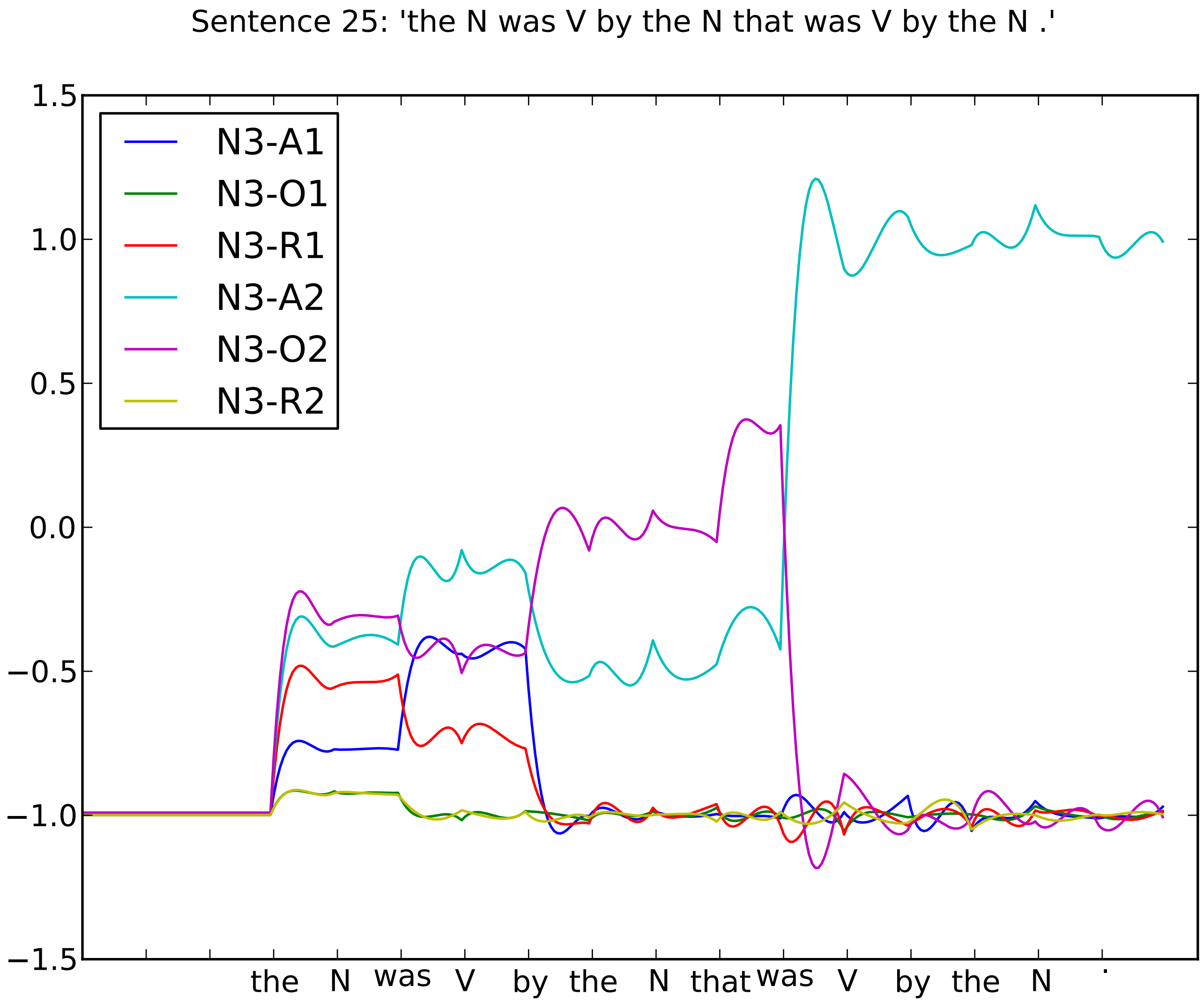
Sentence 25: 'the N was V by the N that was V by the N .'
1.5
1.0
0.5
0.0
−0.5
−1.0
−1.5
N3-A1
N3-O1
N3-R1
N3-A2
N3-O2
N3-R2
the
N
was
V
by
the
N
that
was
V
by
the
N
.

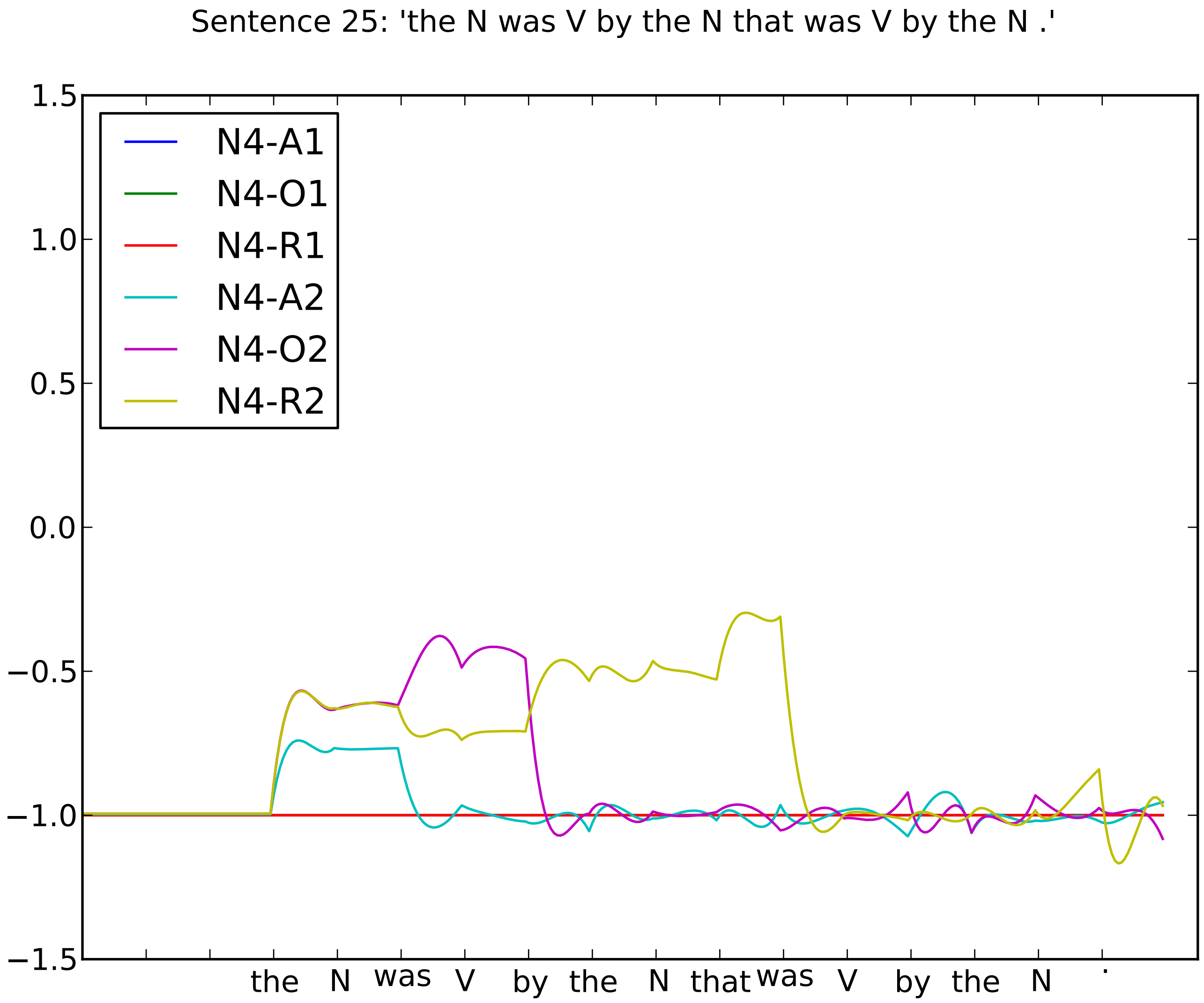
Sentence 25: 'the N was V by the N that was V by the N .'
1.5
1.0
0.5
0.0
−0.5
−1.0
−1.5
N4-A1
N4-O1
N4-R1
N4-A2
N4-O2
N4-R2
the N was V by the N that was V by the N .

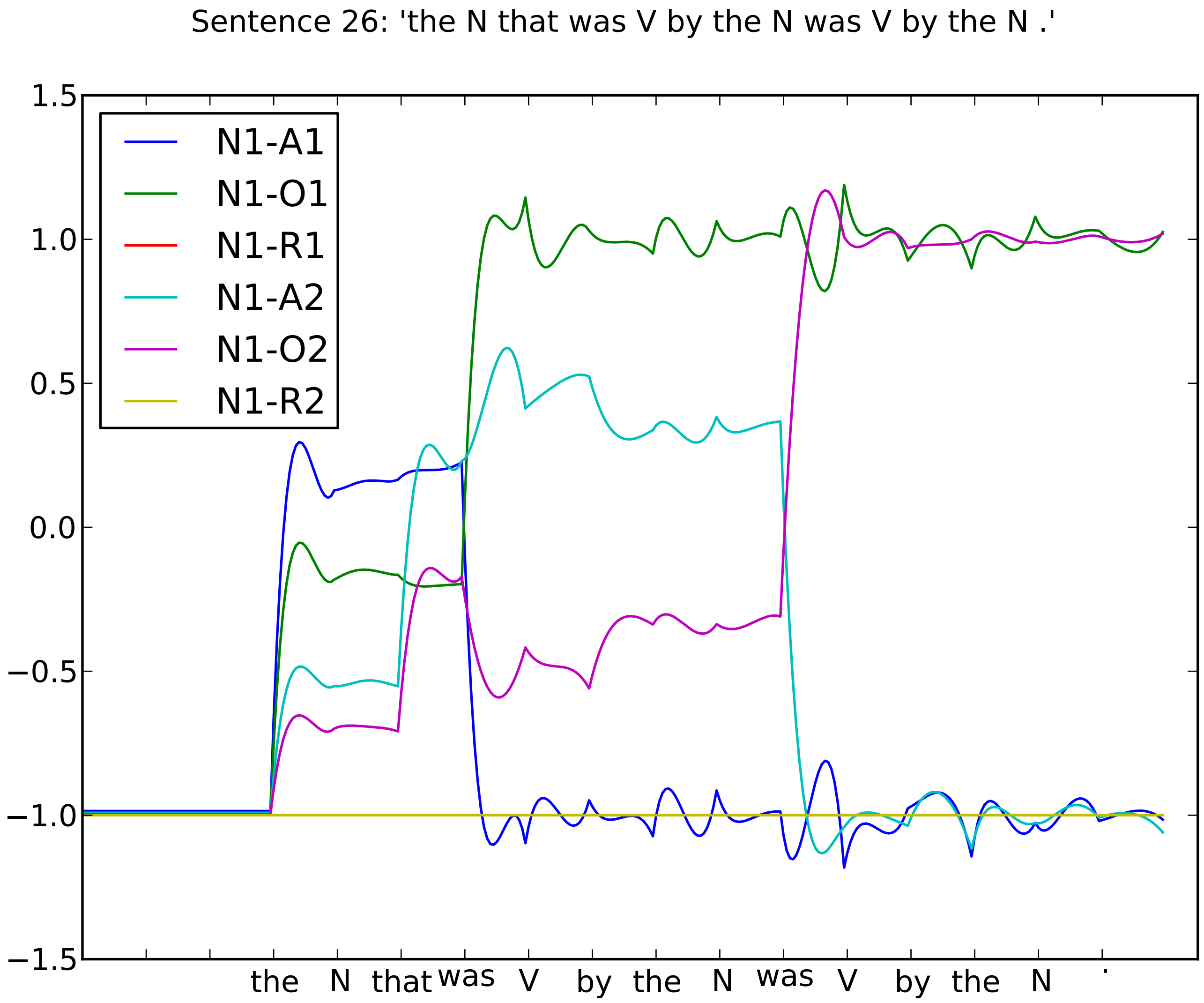

Sentence 26: 'the N that was V by the N was V by the N .'
N1-A1
N1-O1
N1-R1
N1-A2
N1-O2
N1-R2
1.5
1.0
0.5
0.0
−0.5
−1.0
−1.5
the
N
that
was
V
by
the
N
was
V
by
the
N
.

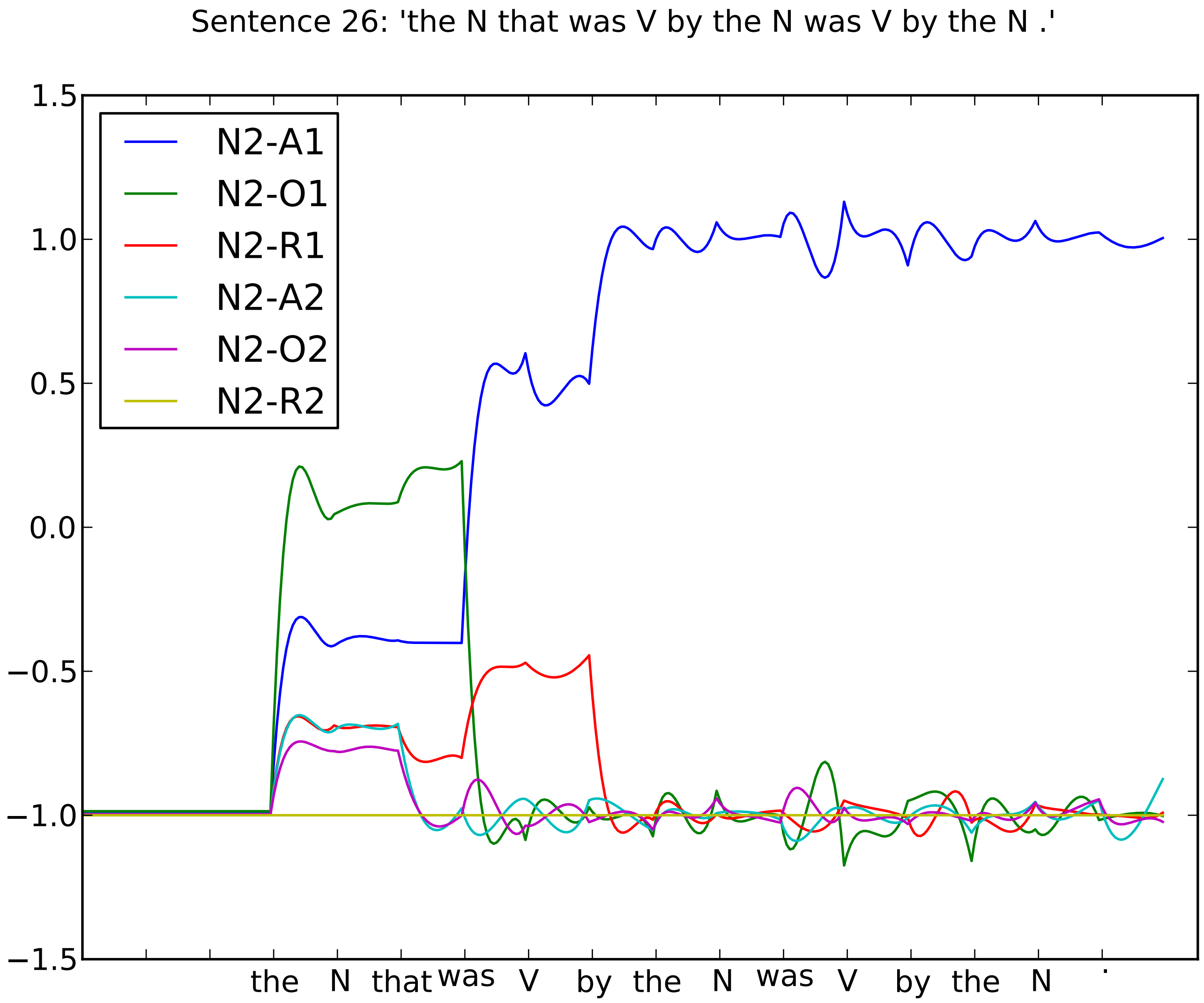

Sentence 26: 'the N that was V by the N was V by the N .'
N2-A1
N2-O1
N2-R1
N2-A2
N2-O2
N2-R2
1.5
1.0
0.5
0.0
−0.5
−1.0
−1.5
the
N
that
was
V
by
the
N
was
V
by
the
N
.

Sentence 26: 'the N that was V by the N was V by the N .'

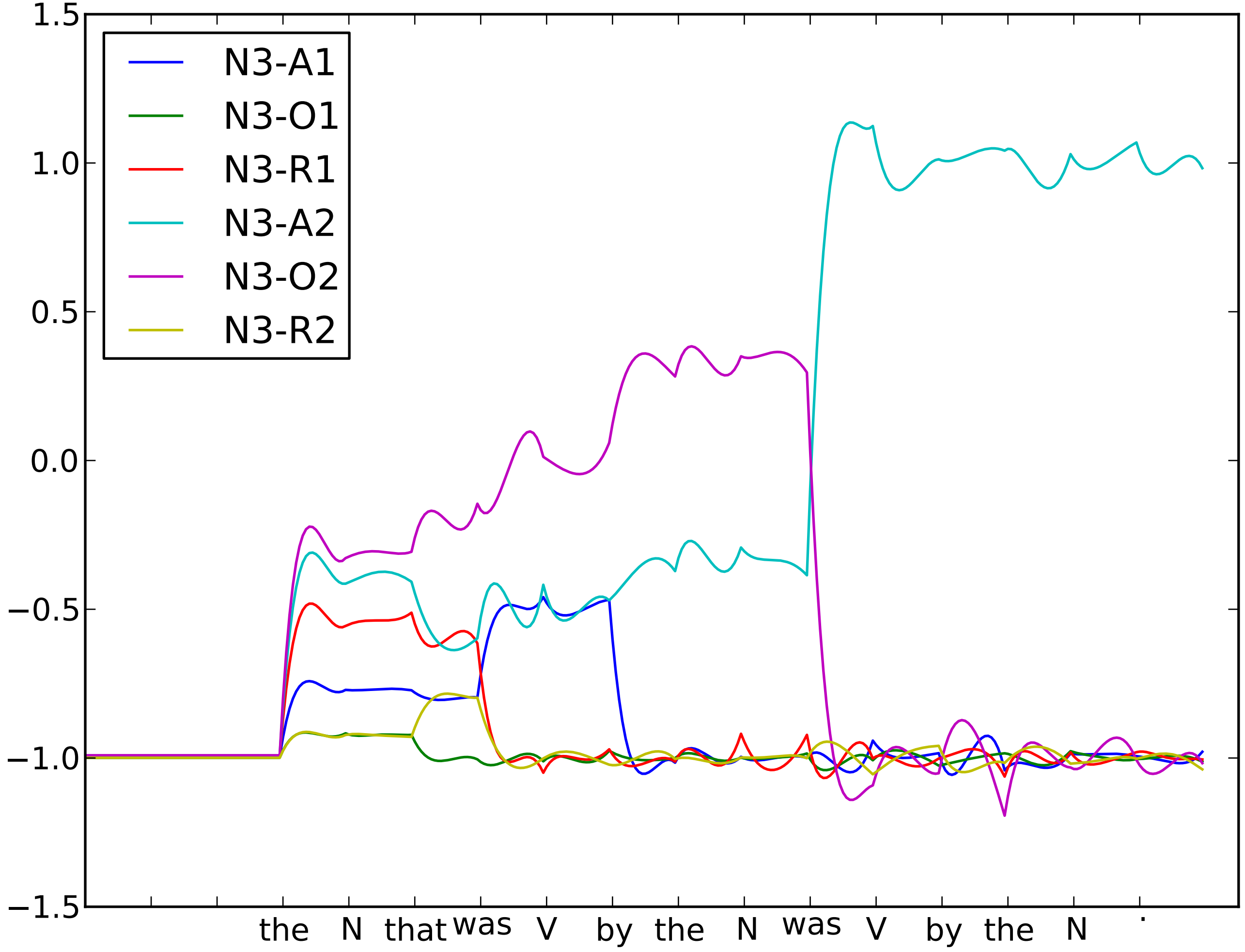

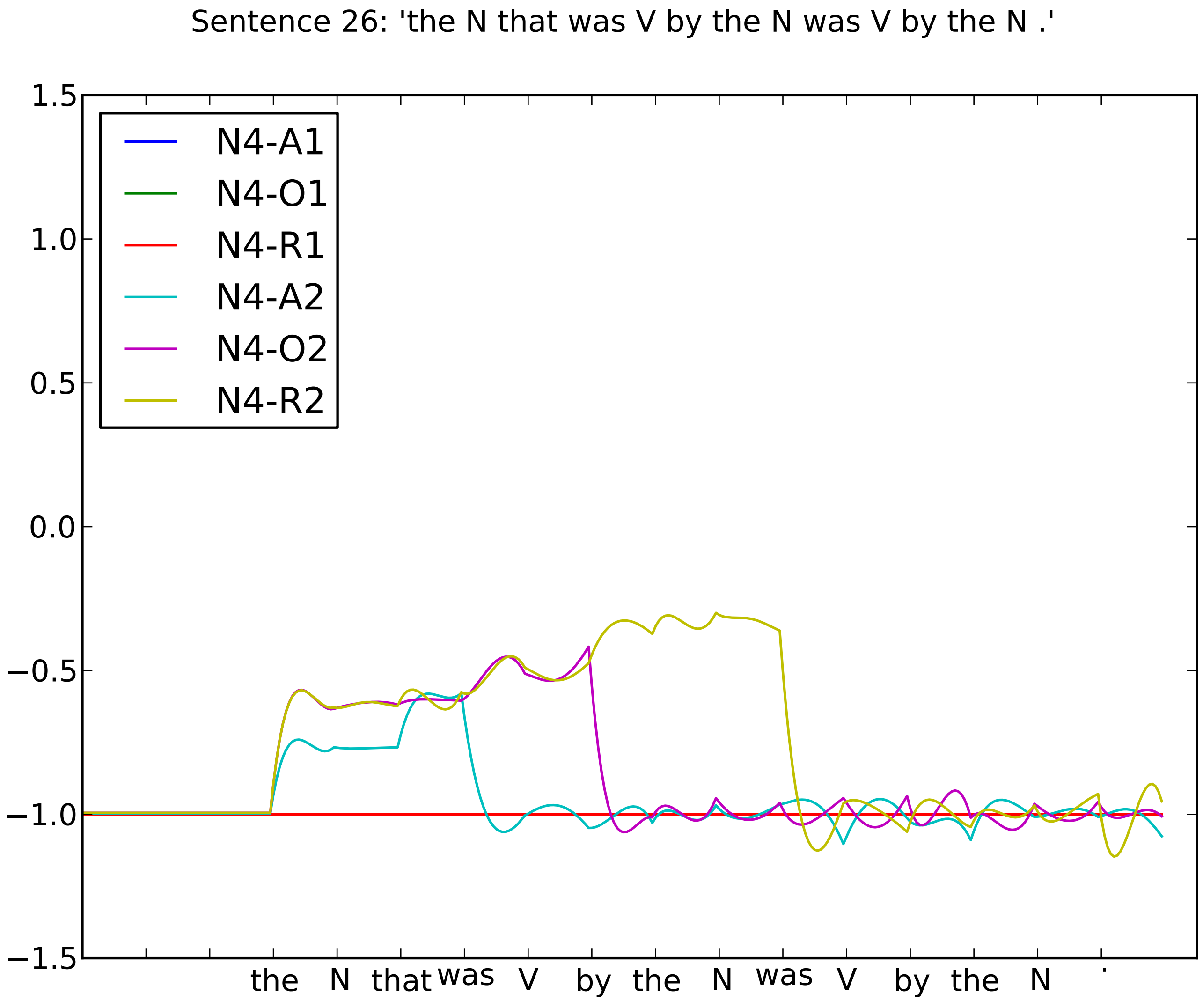
Sentence 26: 'the N that was V by the N was V by the N .'
1.5
1.0
0.5
0.0
−0.5
−1.0
−1.5
N4-A1
N4-O1
N4-R1
N4-A2
N4-O2
N4-R2
the N that was V by the N was V by the N .

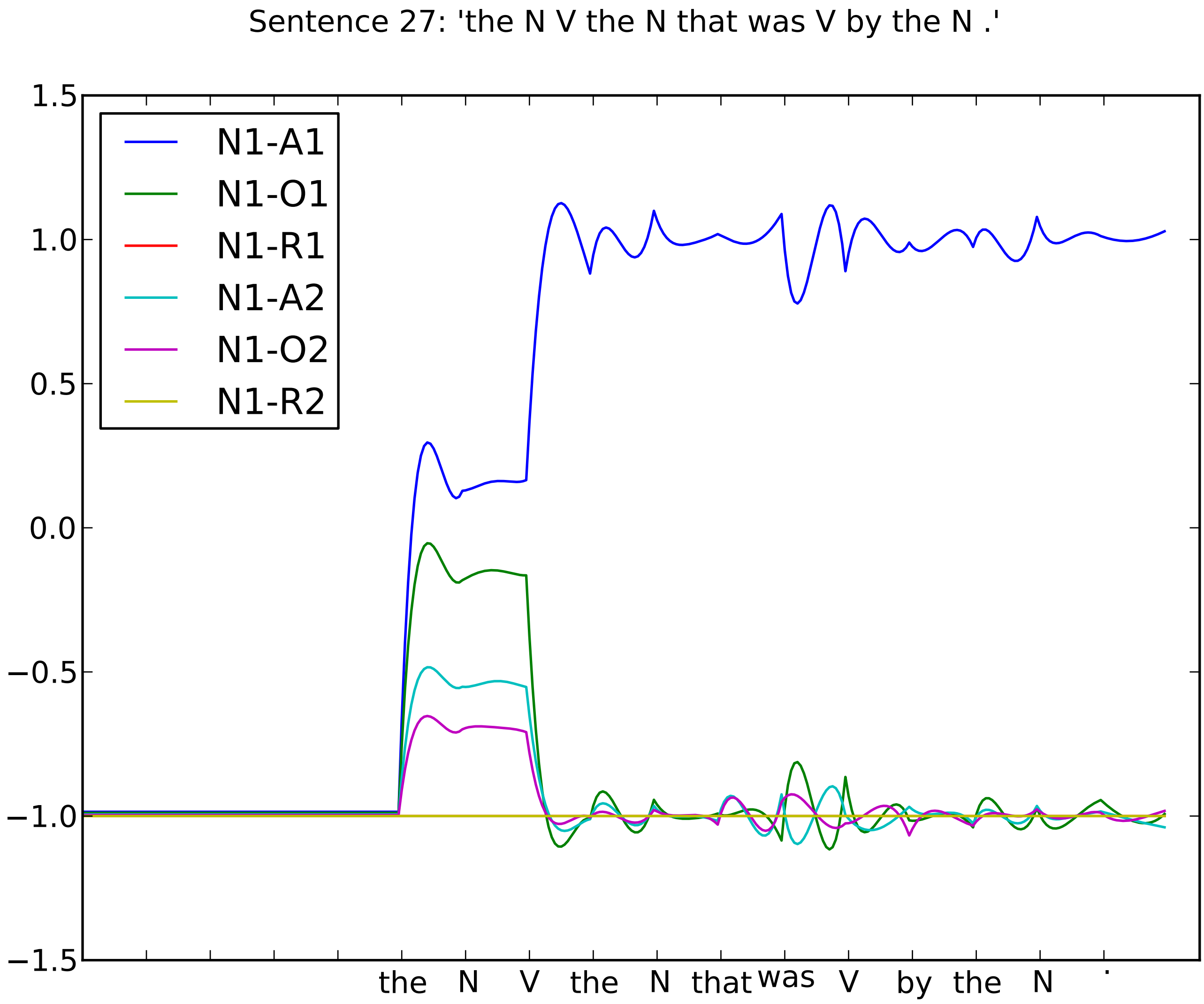
Sentence 27: 'the N V the N that was V by the N .'
N1-A1
N1-O1
N1-R1
N1-A2
N1-O2
N1-R2
1.5
1.0
0.5
0.0
−0.5
−1.0
−1.5
the
N
V
the
N
that
was
V
by
the
N
.

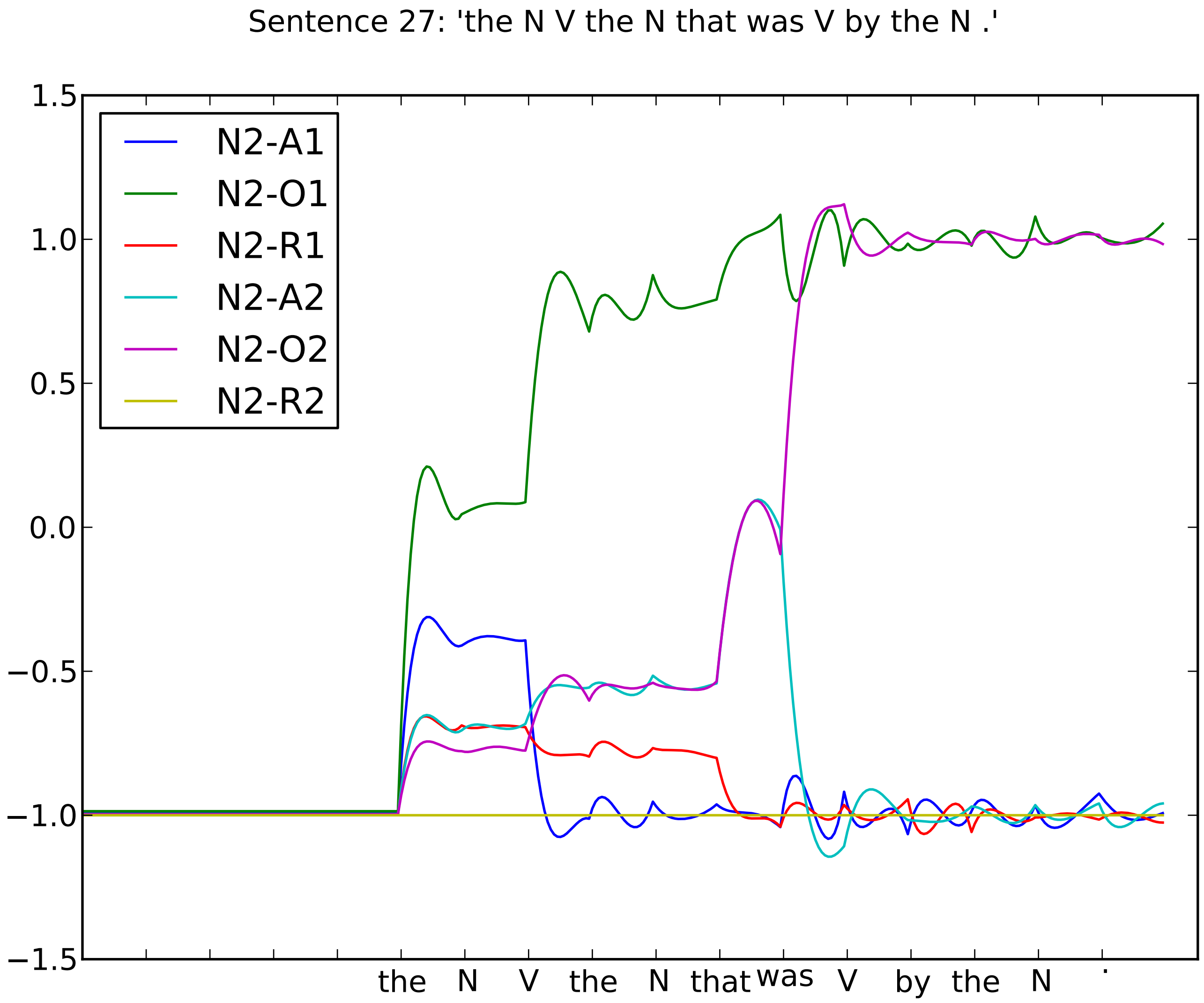

Sentence 27: 'the N V the N that was V by the N .'
N2-A1
N2-O1
N2-R1
N2-A2
N2-O2
N2-R2
1.5
1.0
0.5
0.0
−0.5
−1.0
−1.5
the N V the N that was V by the N .

Sentence 27: 'the N V the N that was V by the N .'

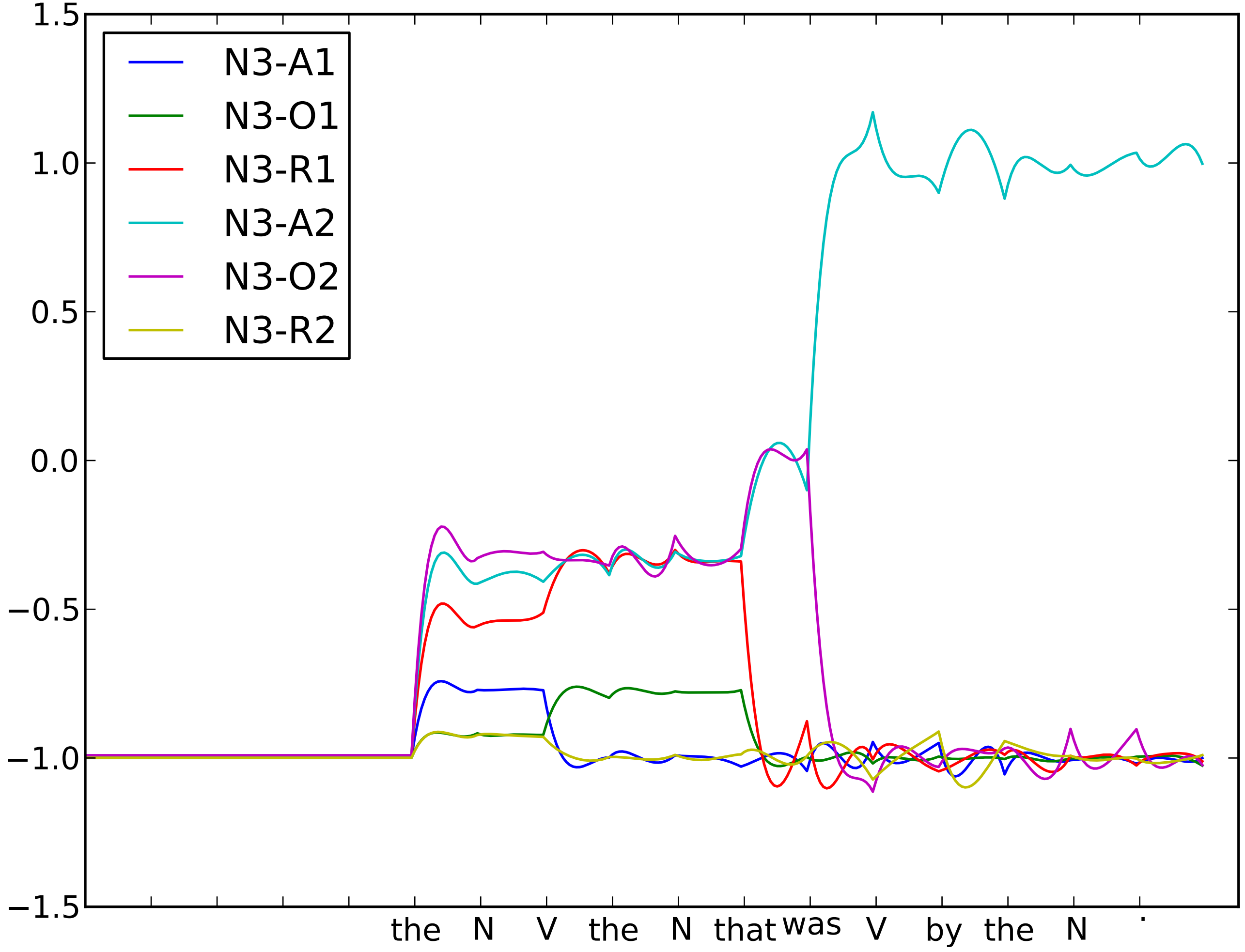

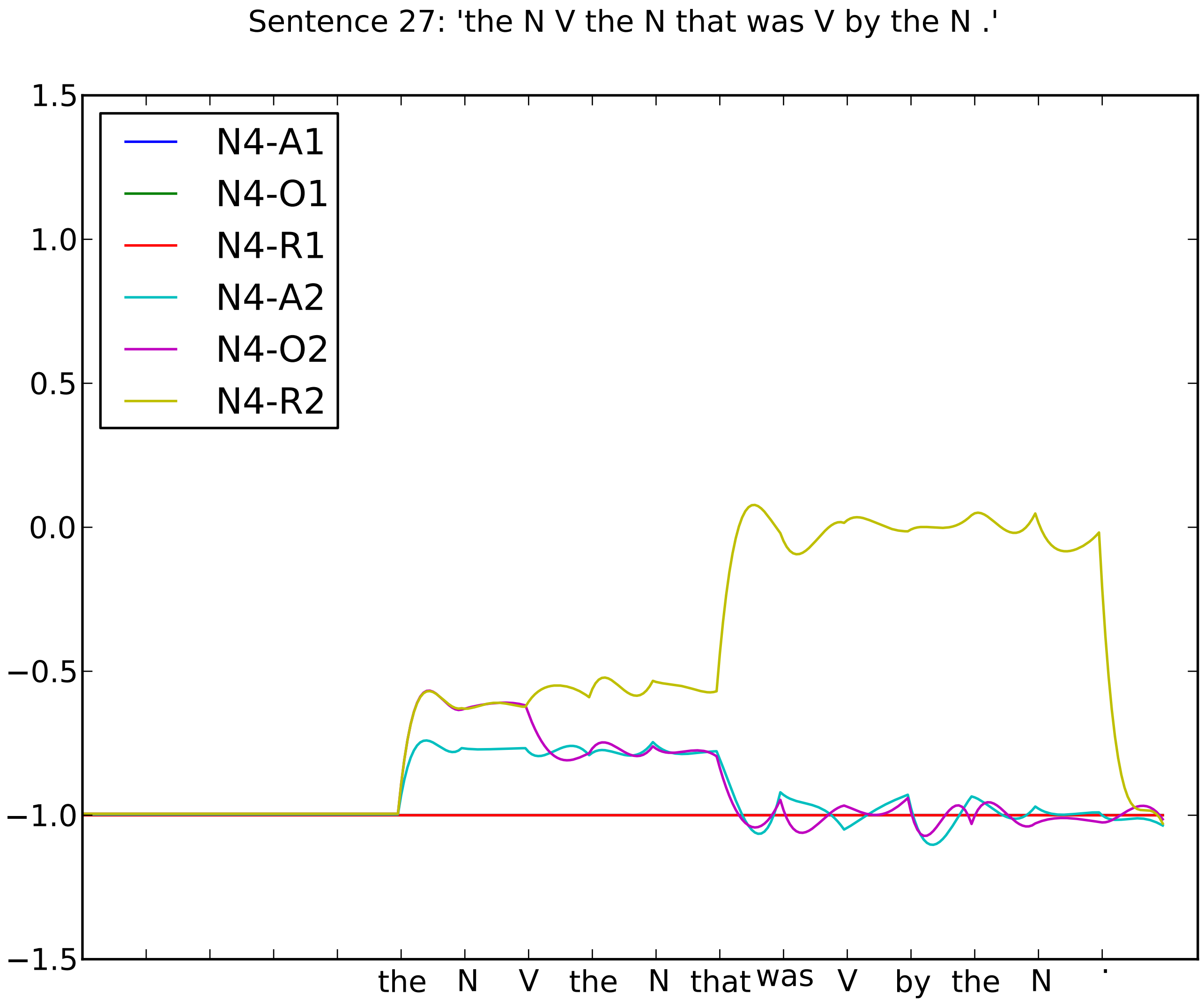

Sentence 27: 'the N V the N that was V by the N .'
N4-A1
N4-O1
N4-R1
N4-A2
N4-O2
N4-R2
1.5
1.0
0.5
0.0
−0.5
−1.0
−1.5
the
N
V
the
N
that
was
V
by
the
N
.

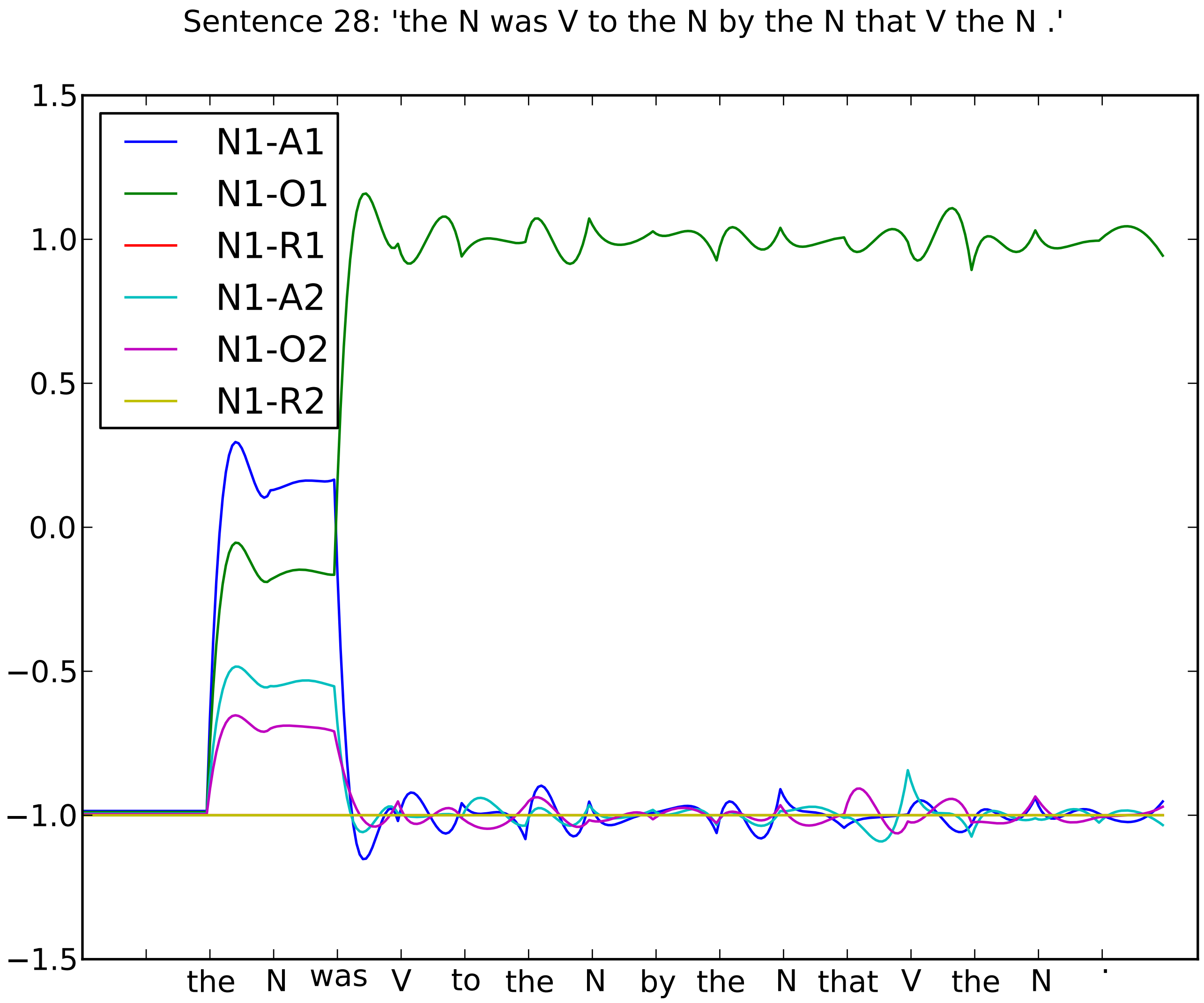

Sentence 28: 'the N was V to the N by the N that V the N .'
1.5
1.0
0.5
0.0
−0.5
−1.0
−1.5
N1-A1
N1-O1
N1-R1
N1-A2
N1-O2
N1-R2
the N was V to the N by the N that V the N .

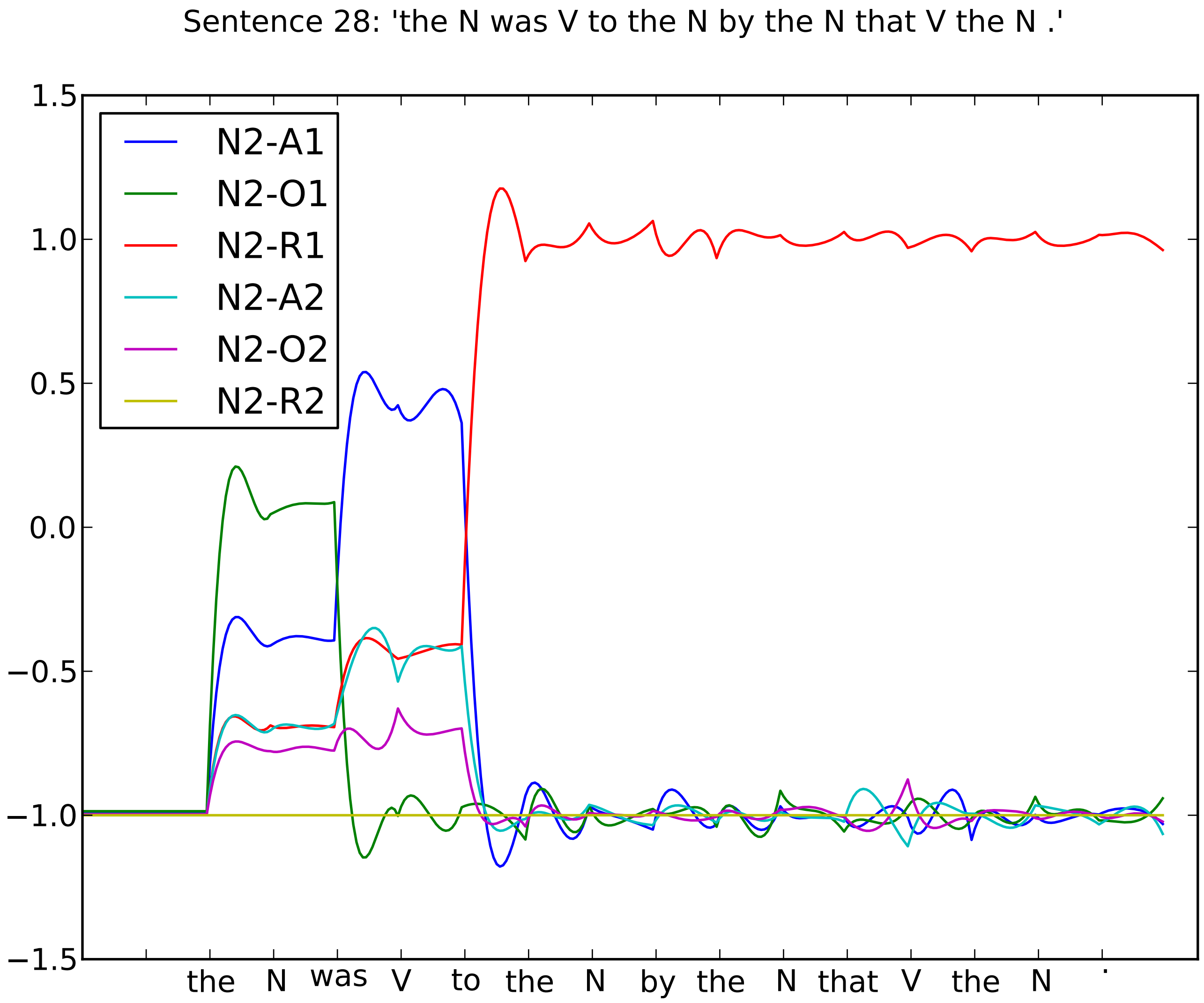

Sentence 28: 'the N was V to the N by the N that V the N .'
N2-A1
N2-O1
N2-R1
N2-A2
N2-O2
N2-R2
1.5
1.0
0.5
0.0
−0.5
−1.0
−1.5
the
N
was
V
to
the
N
by
the
N
that
V
the
N
.

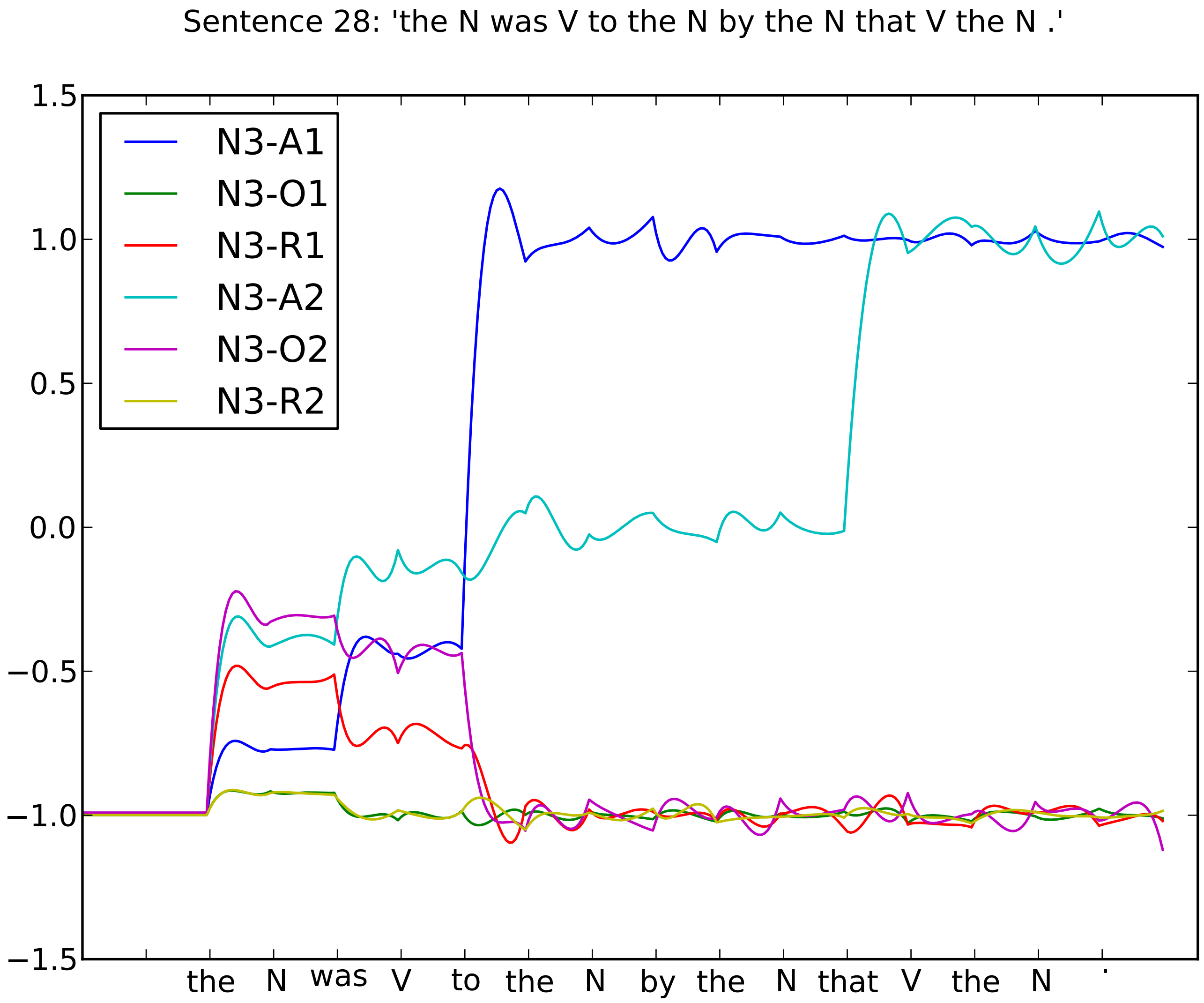

Sentence 28: 'the N was V to the N by the N that V the N .'
1.5
1.0
0.5
0.0
−0.5
−1.0
−1.5
N3-A1
N3-O1
N3-R1
N3-A2
N3-O2
N3-R2
the
N
was
V
to
the
N
by
the
N
that
V
the
N
.

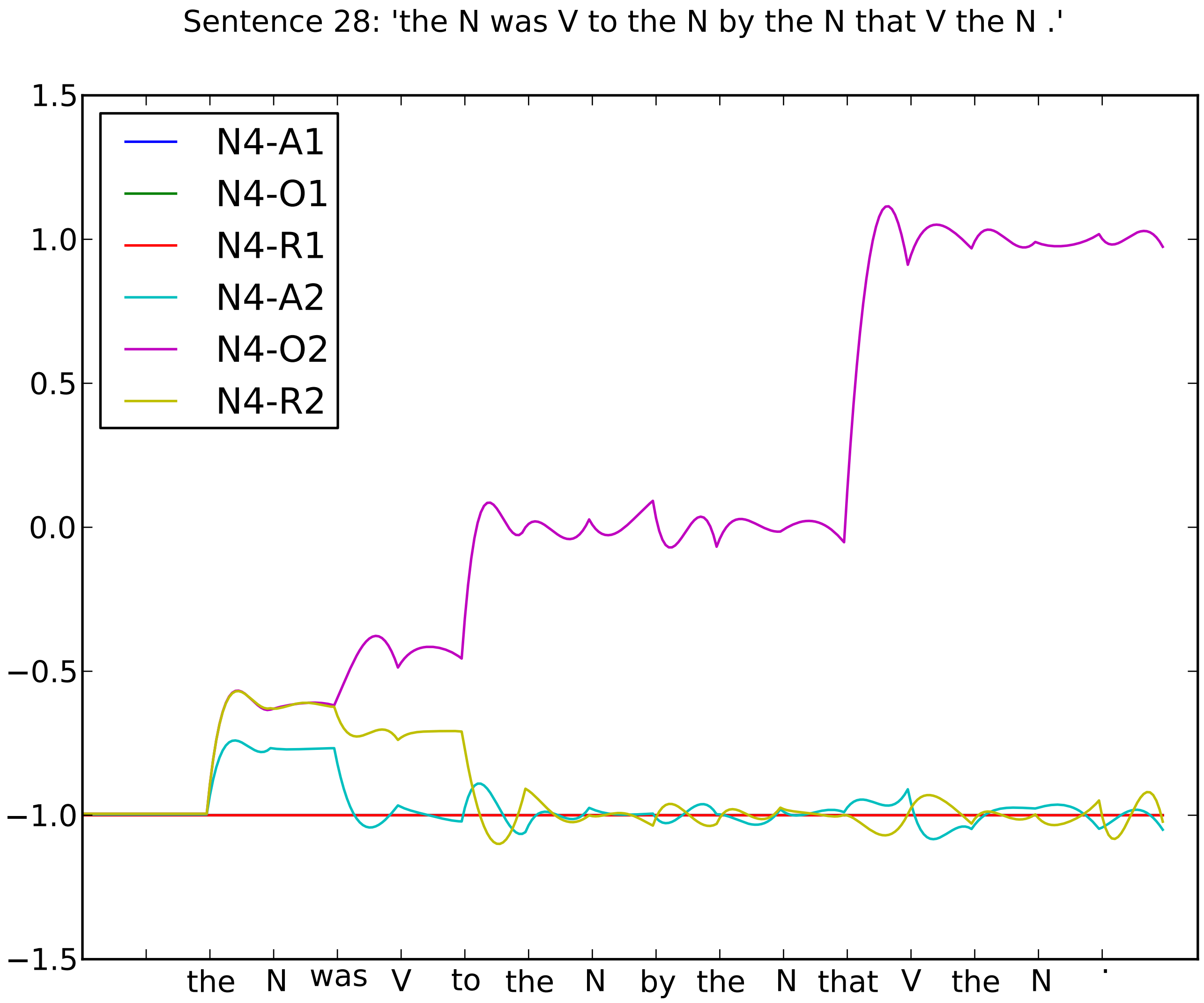
Sentence 28: 'the N was V to the N by the N that V the N .'
1.5
1.0
0.5
0.0
−0.5
−1.0
−1.5
N4-A1
N4-O1
N4-R1
N4-A2
N4-O2
N4-R2
the N was V to the N by the N that V the N .

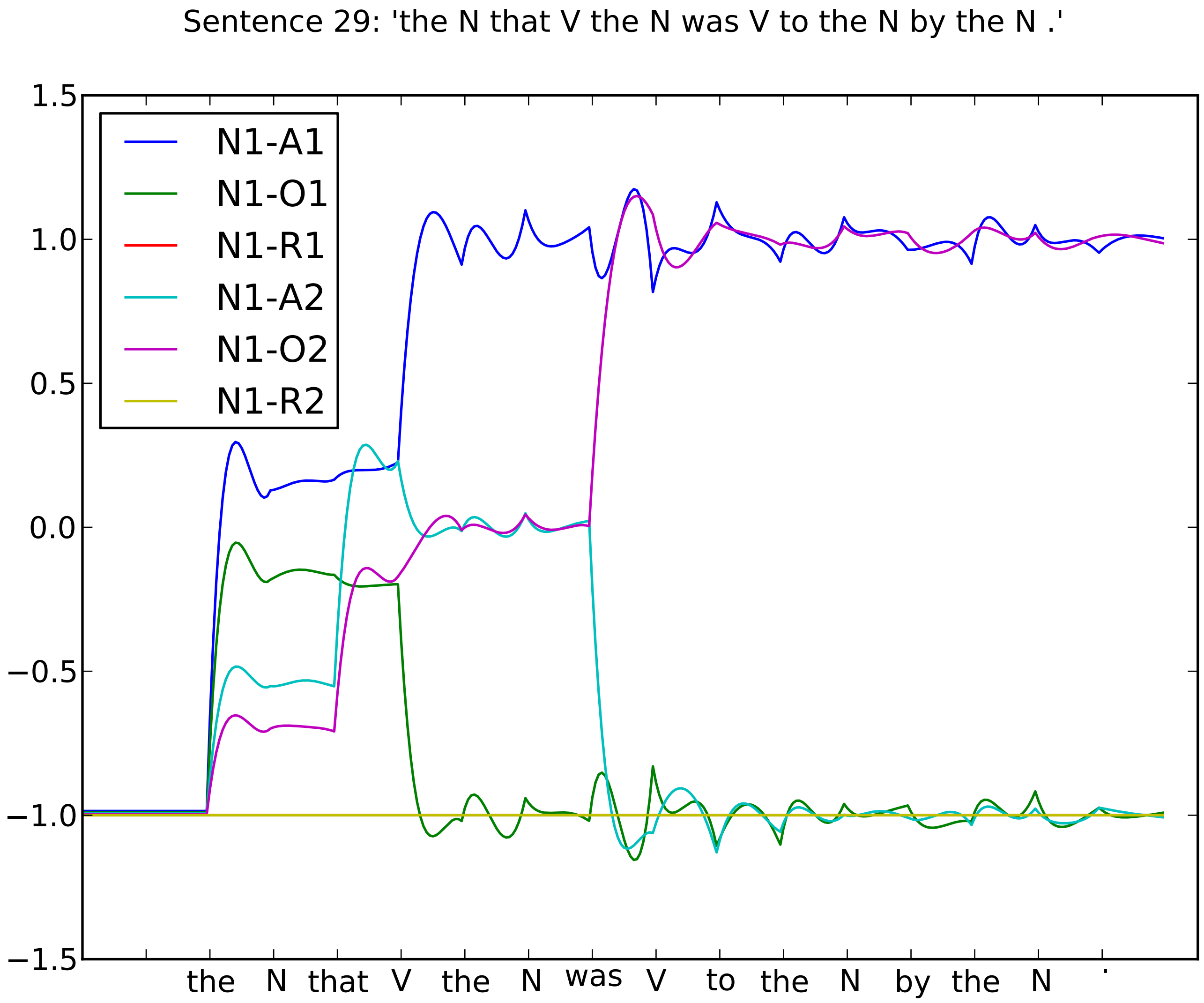

Sentence 29: 'the N that V the N was V to the N by the N .'
N1-A1
N1-O1
N1-R1
N1-A2
N1-O2
N1-R2
1.5
1.0
0.5
0.0
−0.5
−1.0
−1.5
the N that V the N was V to the N by the N .

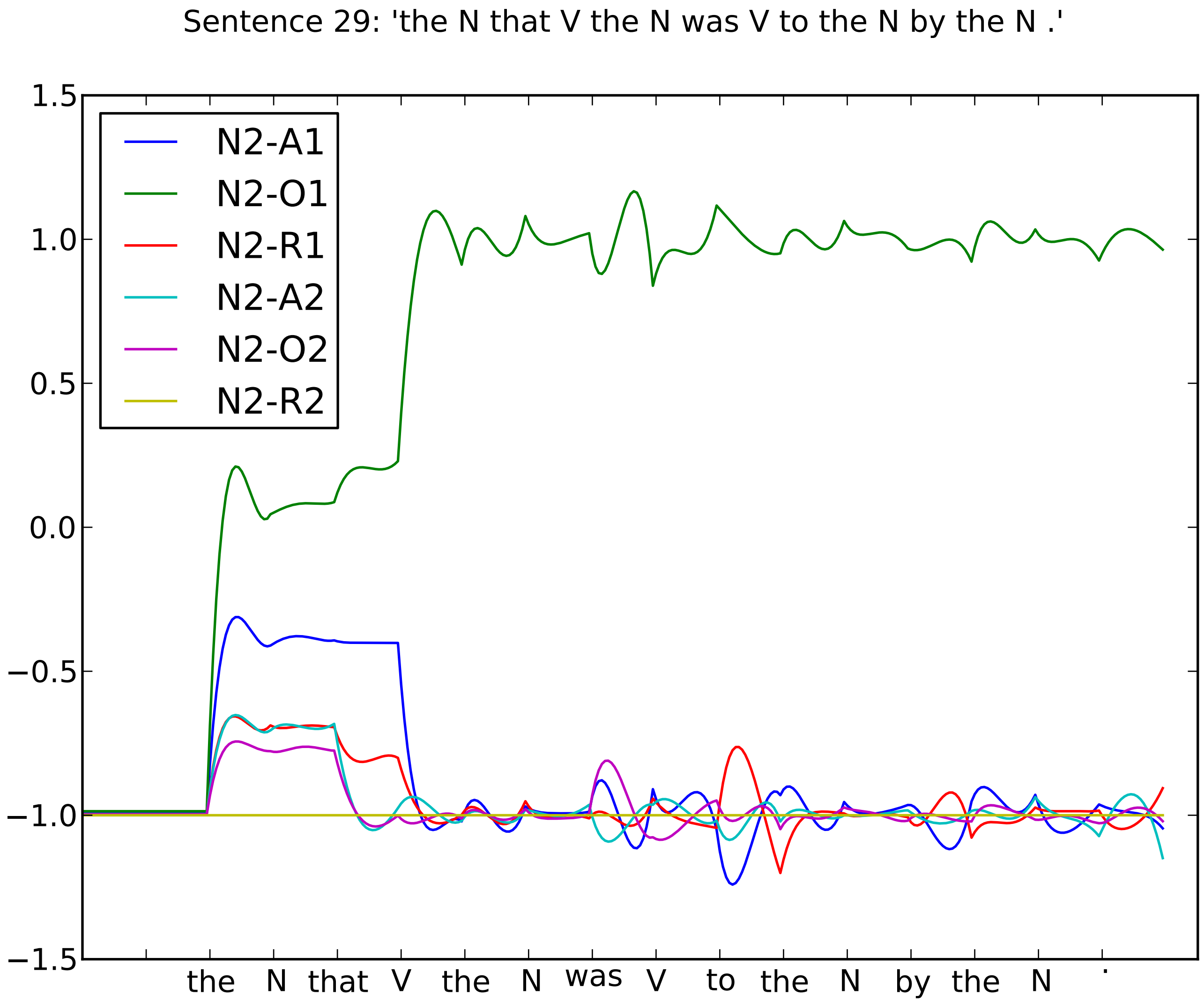
Sentence 29: 'the N that V the N was V to the N by the N .'
N2-A1
N2-O1
N2-R1
N2-A2
N2-O2
N2-R2
1.5
1.0
0.5
0.0
−0.5
−1.0
−1.5
the N that V the N was V to the N by the N .

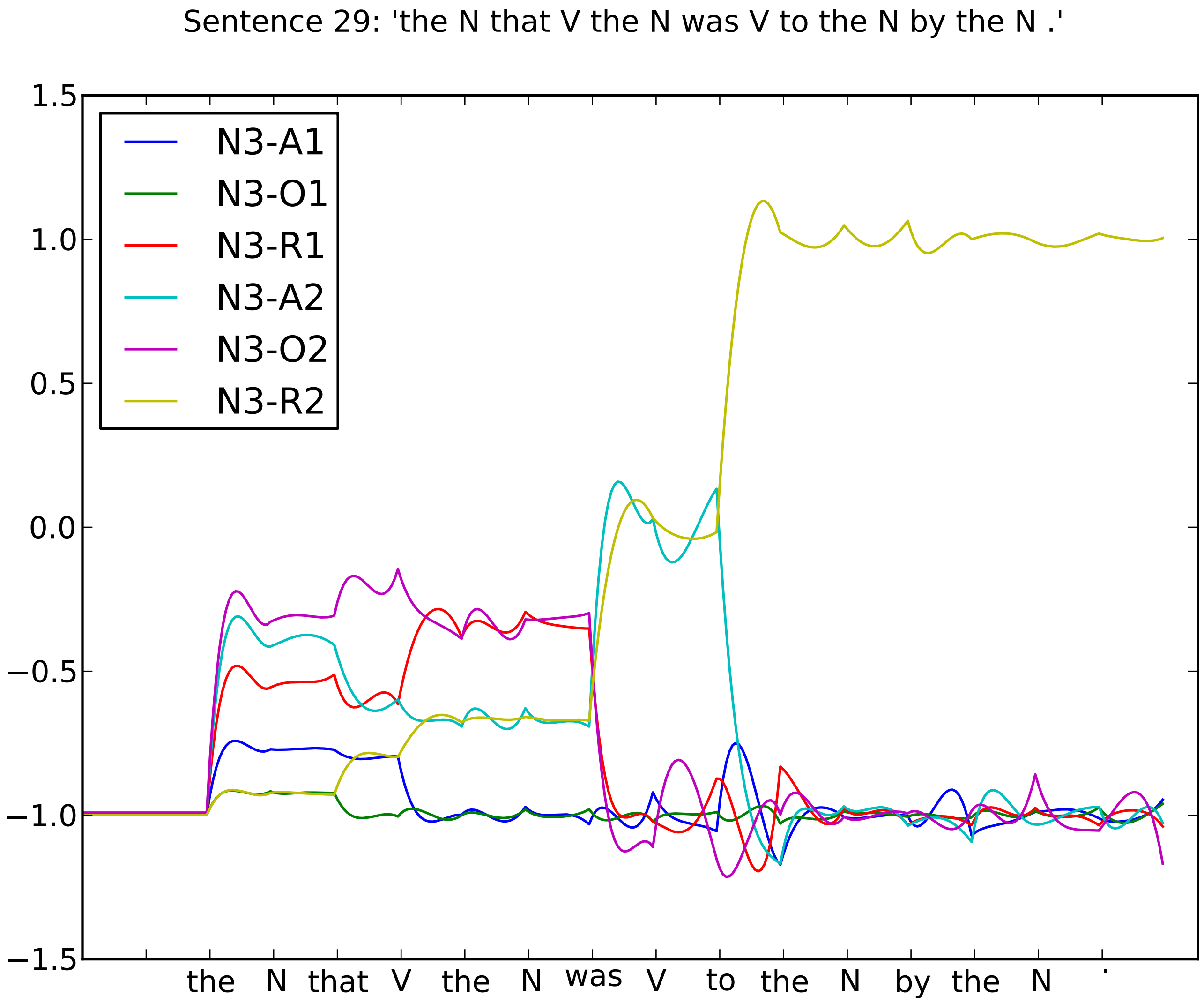

Sentence 29: 'the N that V the N was V to the N by the N .'
1.5
1.0
0.5
0.0
−0.5
−1.0
−1.5
N3-A1
N3-O1
N3-R1
N3-A2
N3-O2
N3-R2
the N that V the N was V to the N by the N .

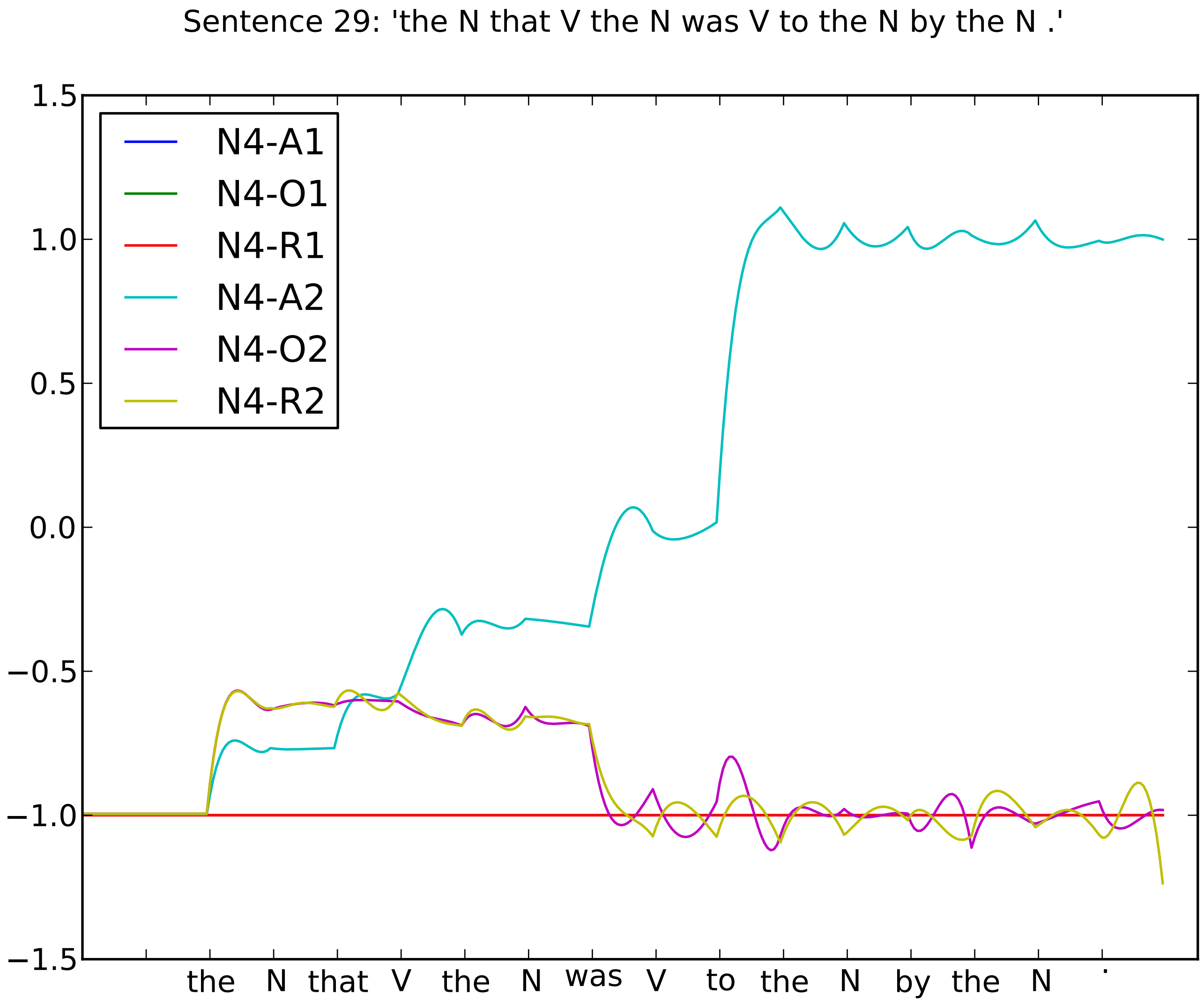

Sentence 29: 'the N that V the N was V to the N by the N .'
N4-A1
N4-O1
N4-R1
N4-A2
N4-O2
N4-R2
1.5
1.0
0.5
0.0
−0.5
−1.0
−1.5
the
N
that
V
the
N
was
V
to
the
N
by
the
N
.

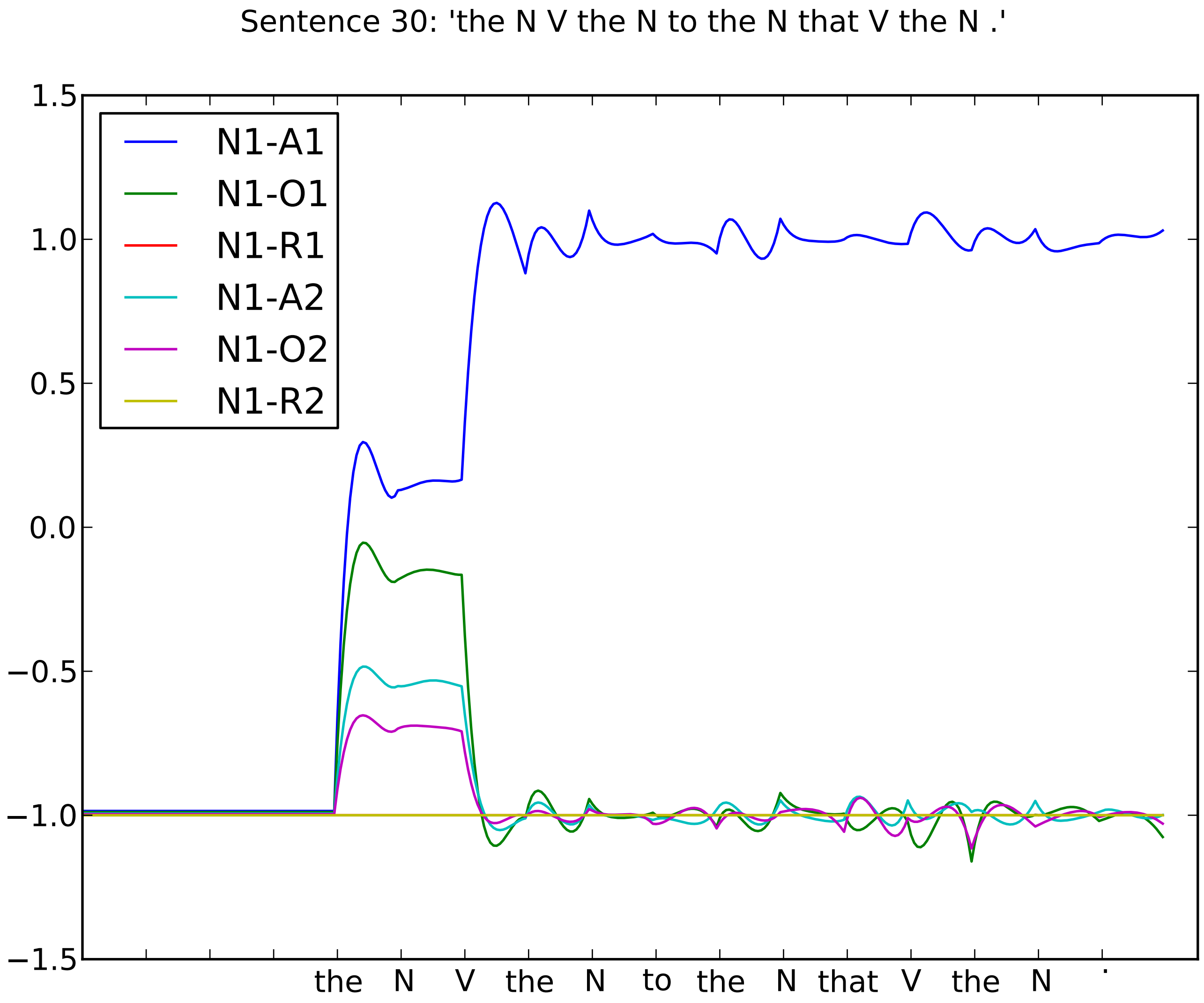

Sentence 30: 'the N V the N to the N that V the N .'
1.5
1.0
0.5
0.0
−0.5
−1.0
−1.5
N1-A1
N1-O1
N1-R1
N1-A2
N1-O2
N1-R2
the N V the N to the N that V the N .

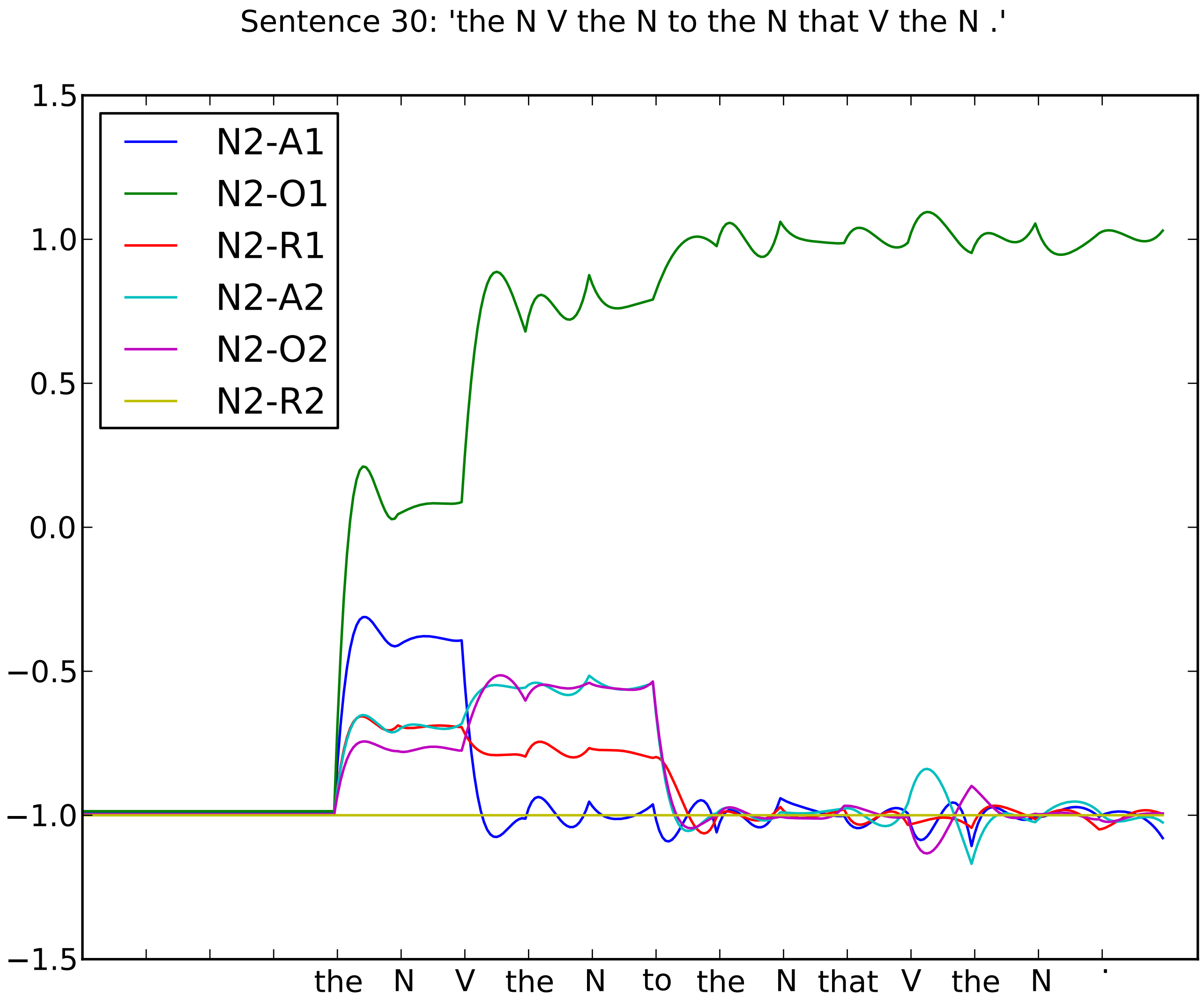
Sentence 30: 'the N V the N to the N that V the N .'
N2-A1
N2-O1
N2-R1
N2-A2
N2-O2
N2-R2
1.5
1.0
0.5
0.0
−0.5
−1.0
−1.5
the
N
V
the
N
to
the
N
that
V
the
N
.

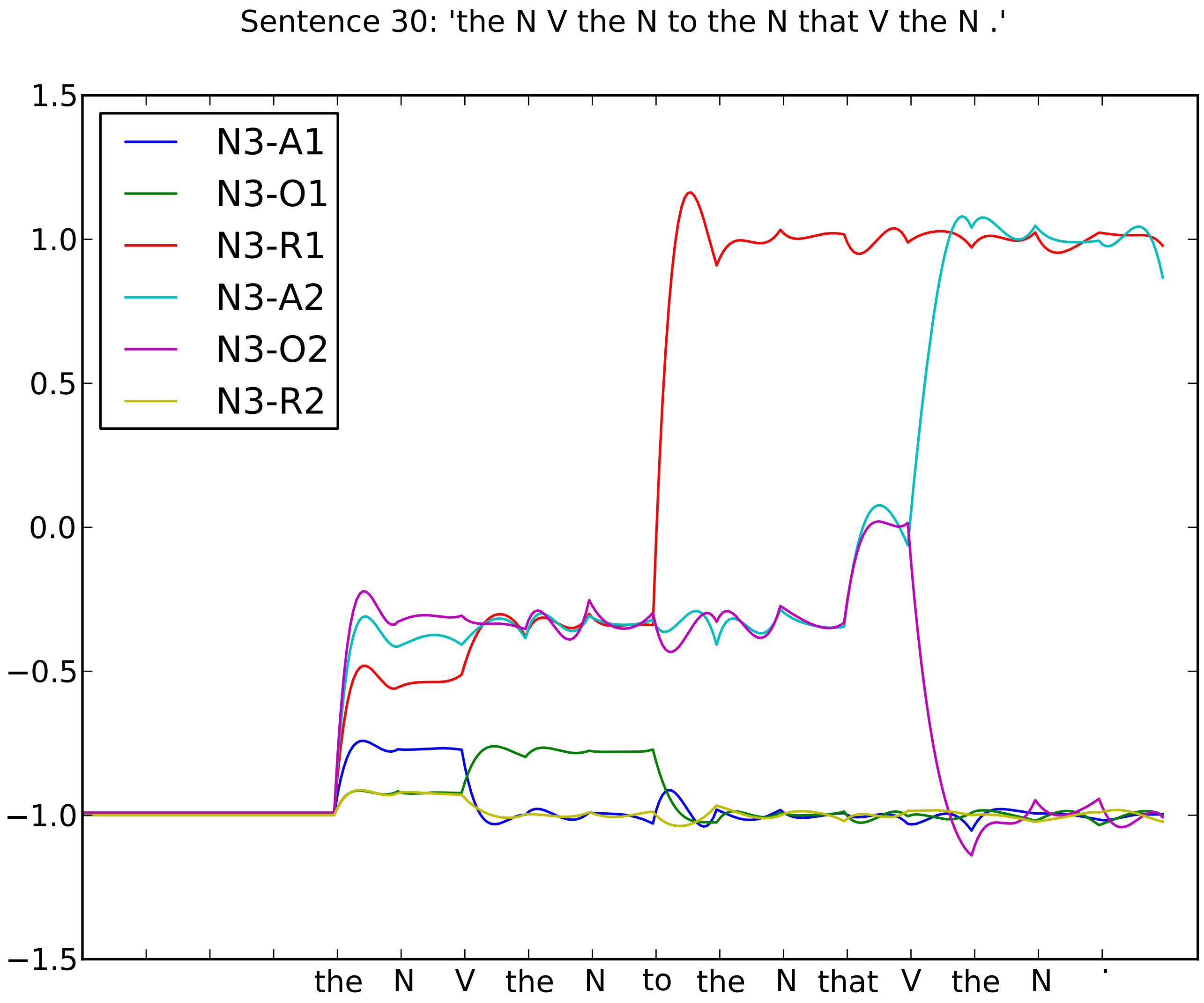
Sentence 30: 'the N V the N to the N that V the N .'
N3-A1
N3-O1
N3-R1
N3-A2
N3-O2
N3-R2
1.5
1.0
0.5
0.0
−0.5
−1.0
−1.5
the
N
V
the
N
to
the
N
that
V
the
N
.

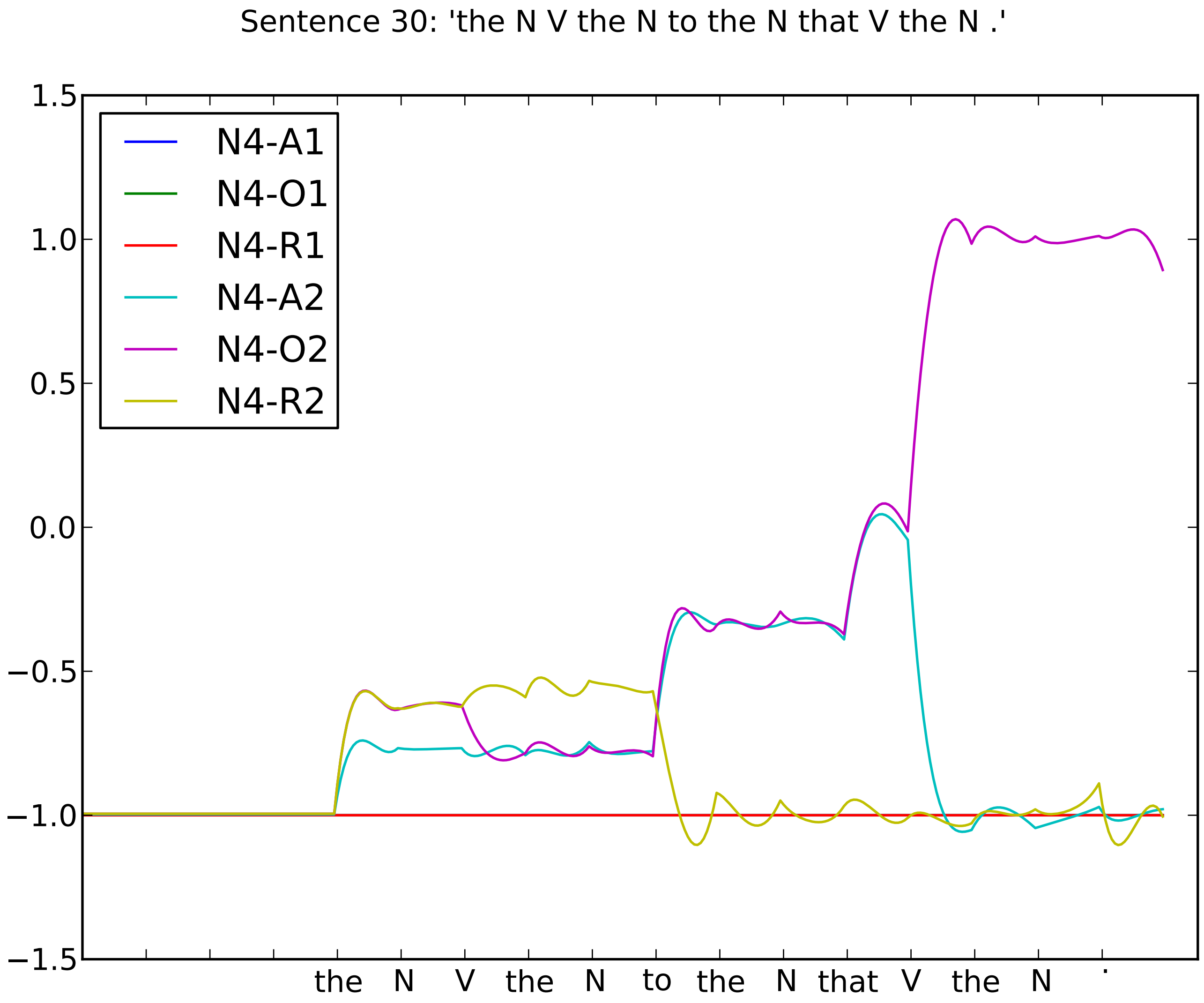
Sentence 30: 'the N V the N to the N that V the N .'
1.5
1.0
0.5
0.0
−0.5
−1.0
−1.5
N4-A1
N4-O1
N4-R1
N4-A2
N4-O2
N4-R2
the N V the N to the N that V the N .

Sentence 31: 'the N was V from the N to the N that V the N .'

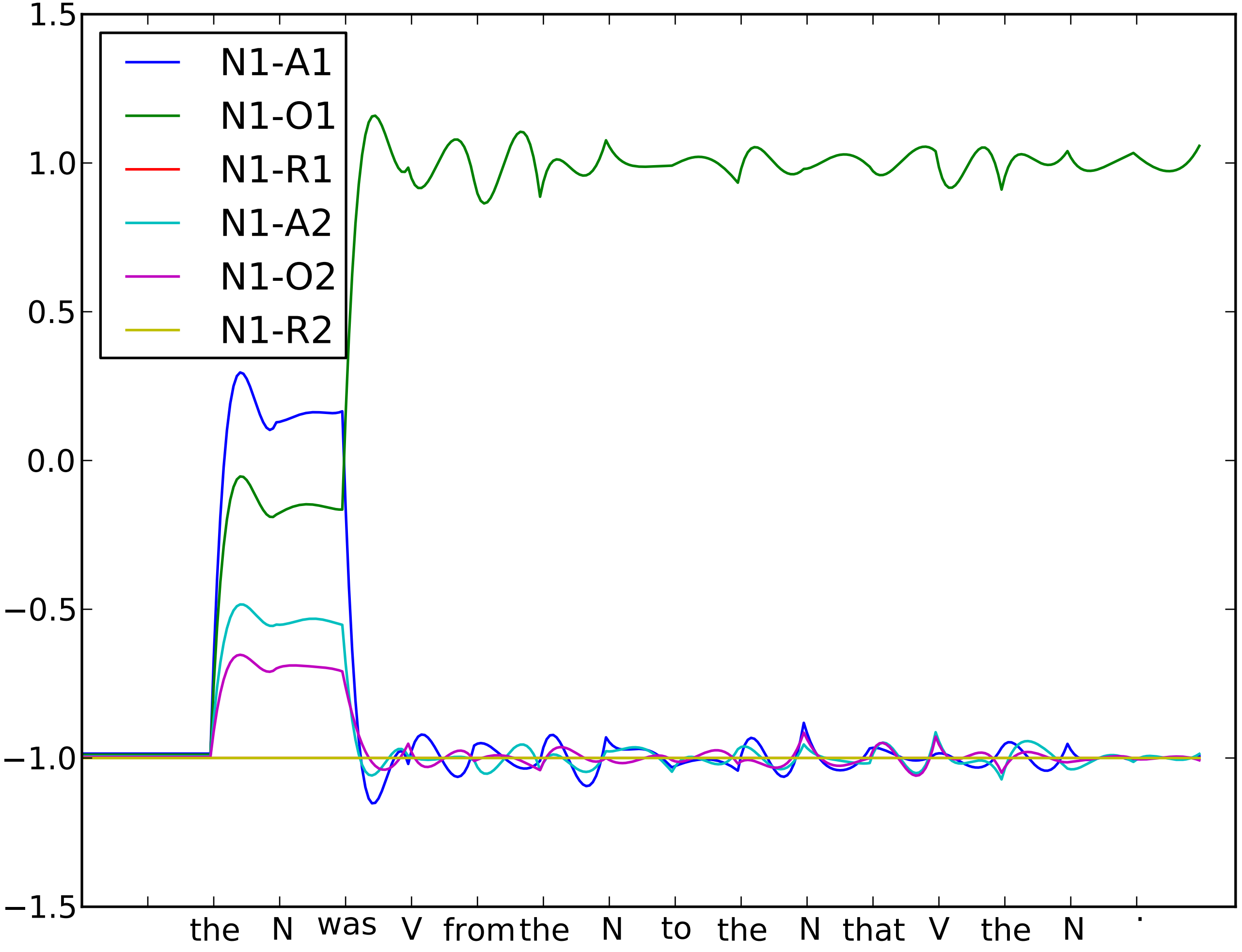

Sentence 31: 'the N was V from the N to the N that V the N .'

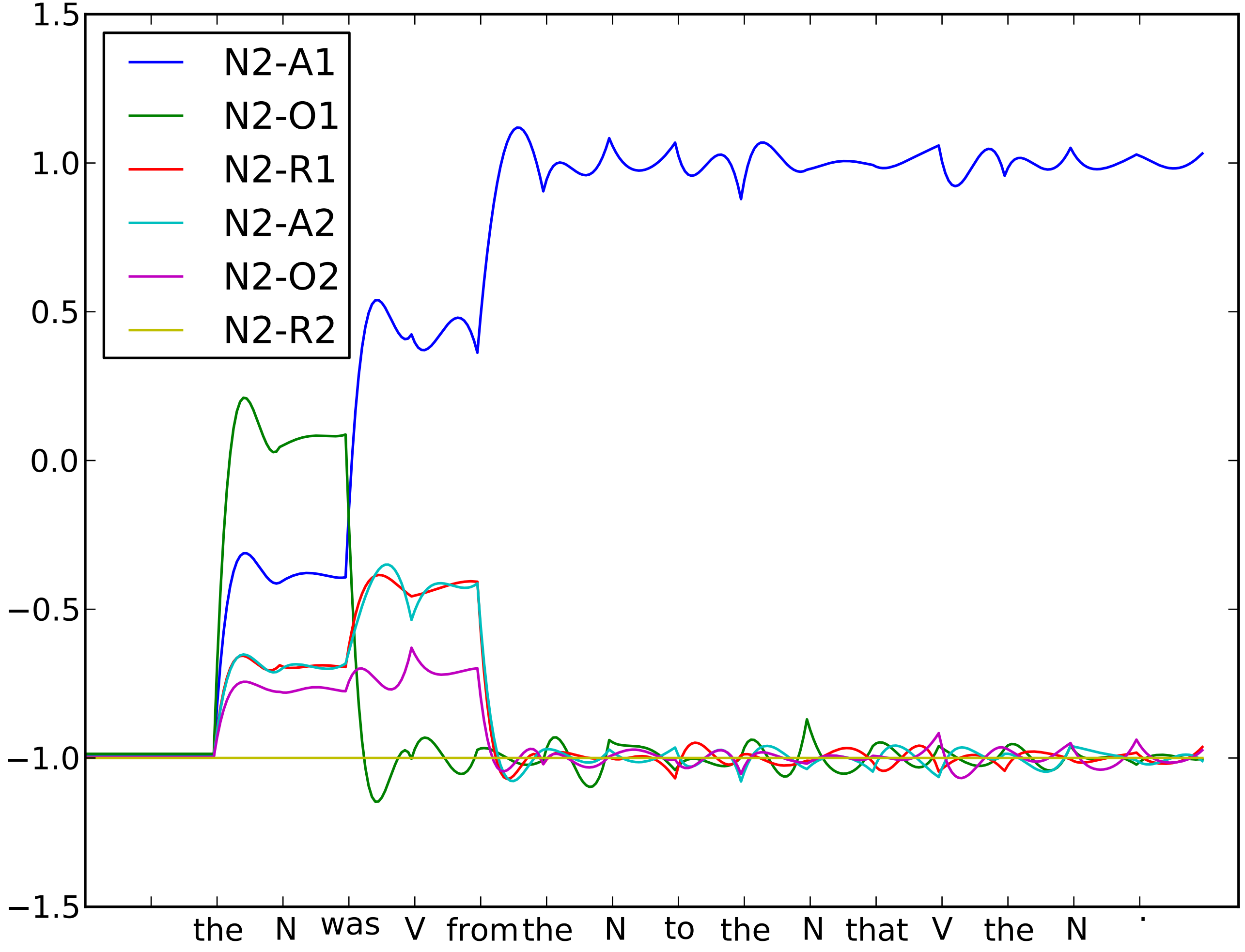

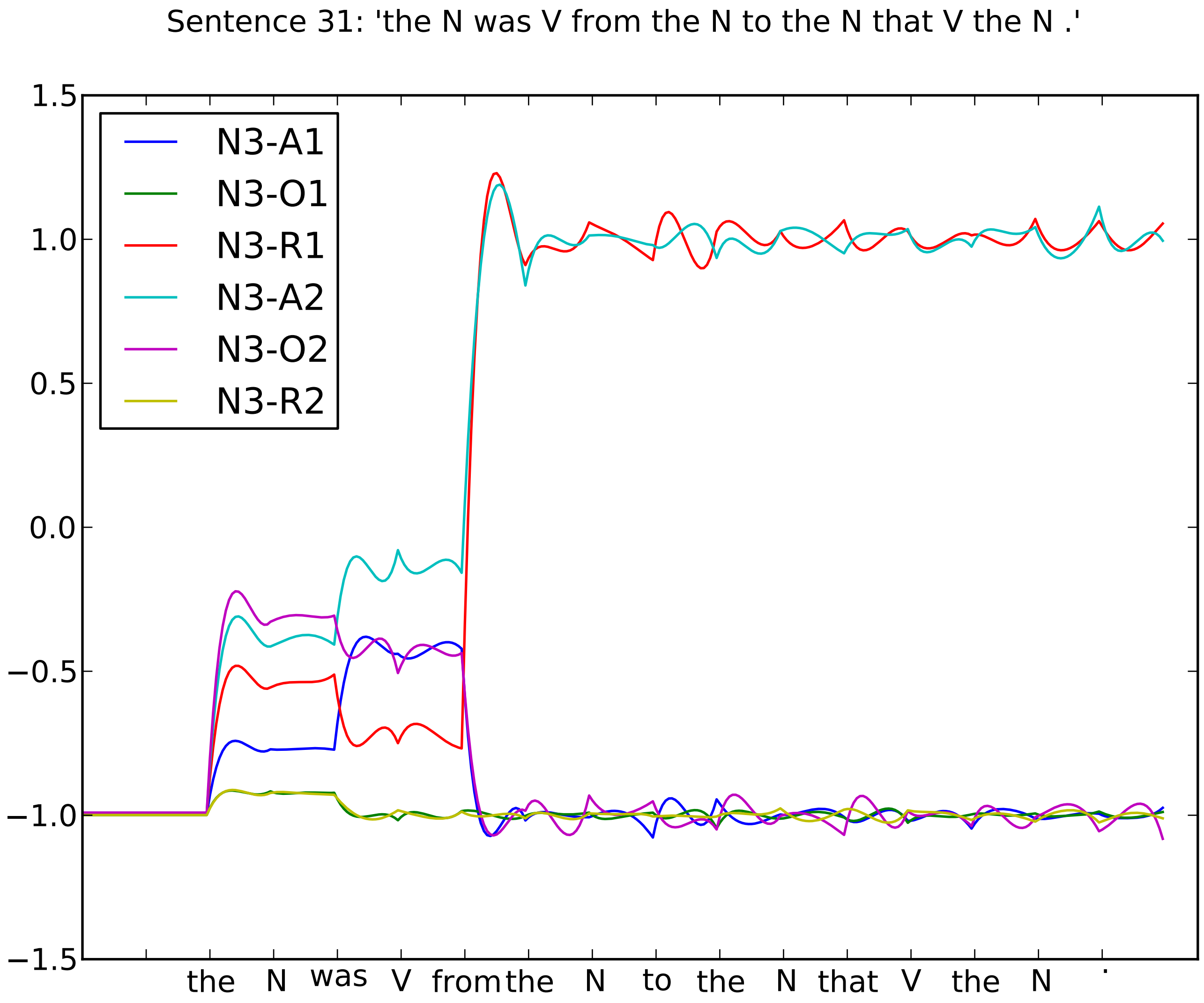

Sentence 31: 'the N was V from the N to the N that V the N .'
N3-A1
N3-O1
N3-R1
N3-A2
N3-O2
N3-R2
1.5
1.0
0.5
0.0
−0.5
−1.0
−1.5
the
N
was
V
from
the
N
to
the
N
that
V
the
N
.

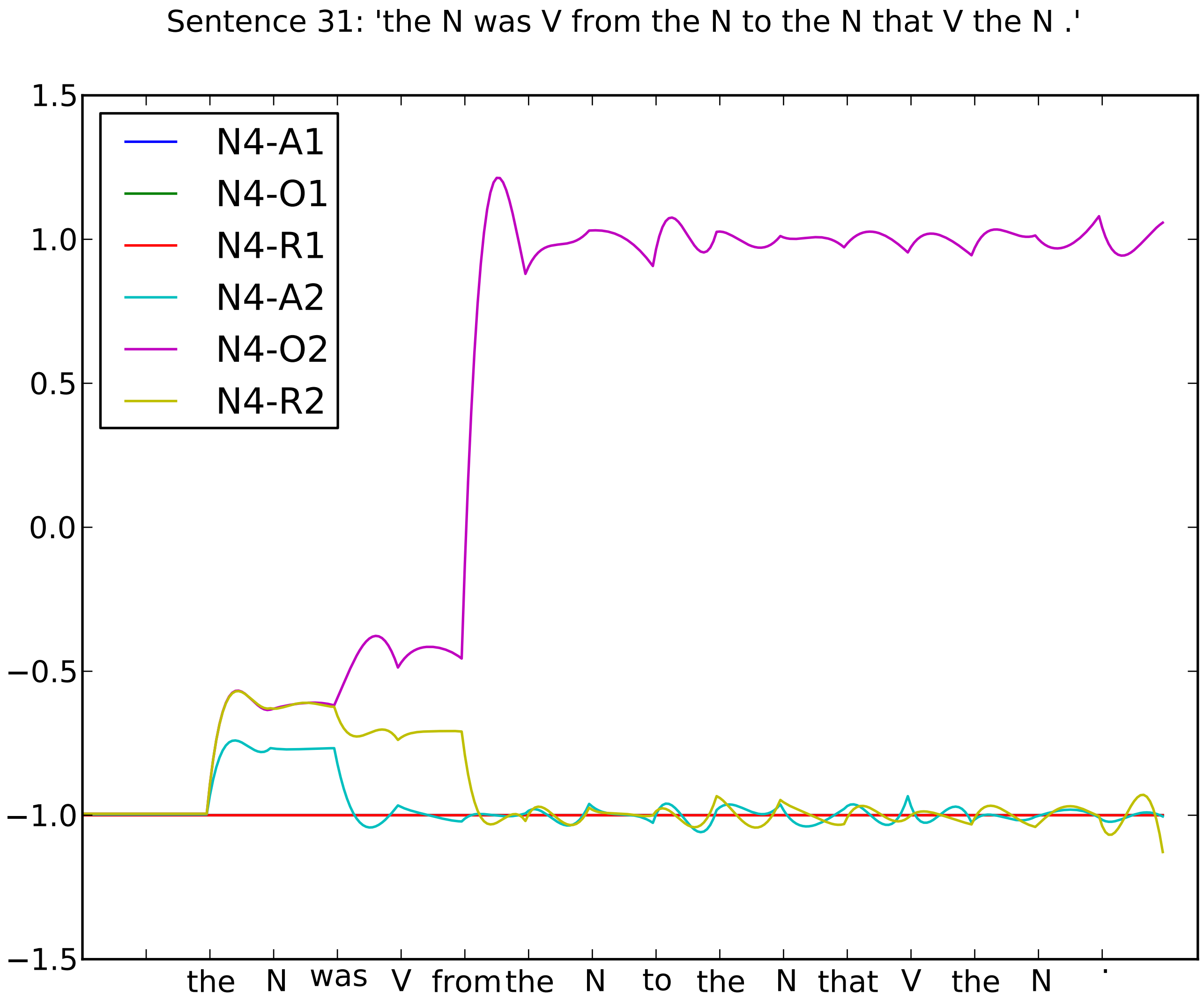
Sentence 31: 'the N was V from the N to the N that V the N .'
1.5
1.0
0.5
0.0
−0.5
−1.0
−1.5
N4-A1
N4-O1
N4-R1
N4-A2
N4-O2
N4-R2
the
N
was
V
from
the
N
to
the
N
that
V
the
N
.

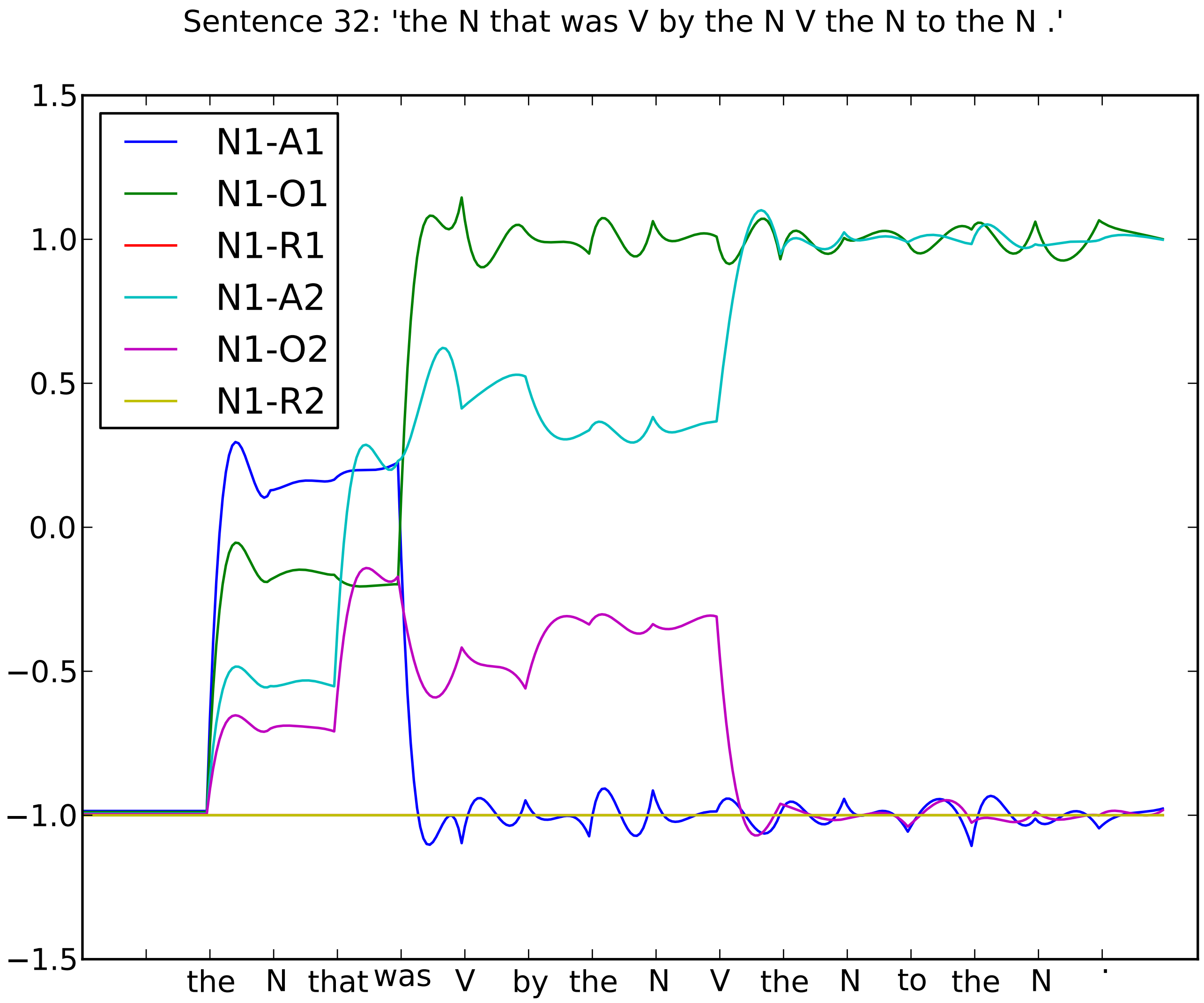
Sentence 32: 'the N that was V by the N V the N to the N .'
1.5
1.0
0.5
0.0
−0.5
−1.0
−1.5
N1-A1
N1-O1
N1-R1
N1-A2
N1-O2
N1-R2
the
N
that
was
V
by
the
N
V
the
N
to
the
N
.

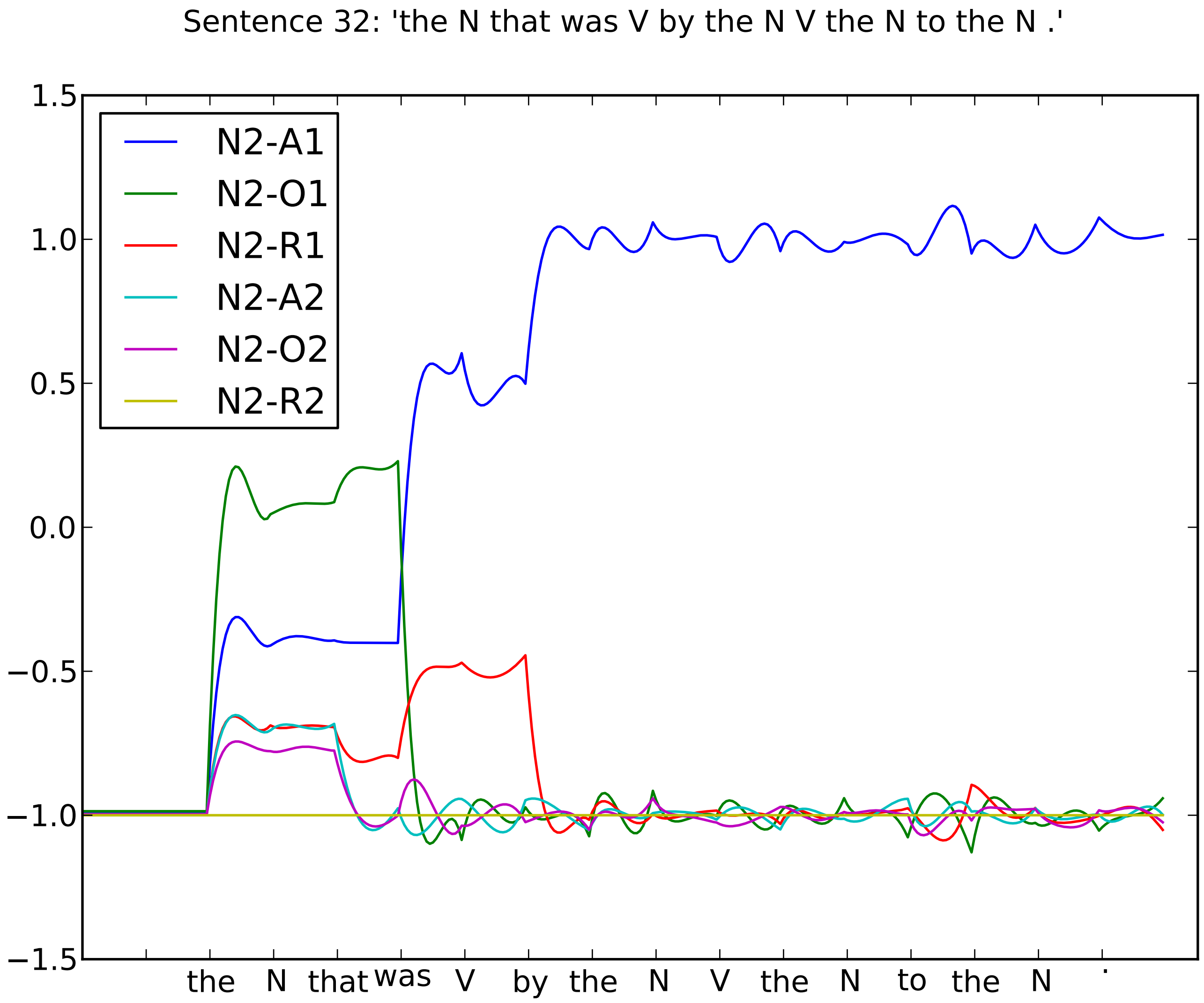

Sentence 32: 'the N that was V by the N V the N to the N .'
N2-A1
N2-O1
N2-R1
N2-A2
N2-O2
N2-R2
1.5
1.0
0.5
0.0
−0.5
−1.0
−1.5
the N that was V by the N V the N to the N .

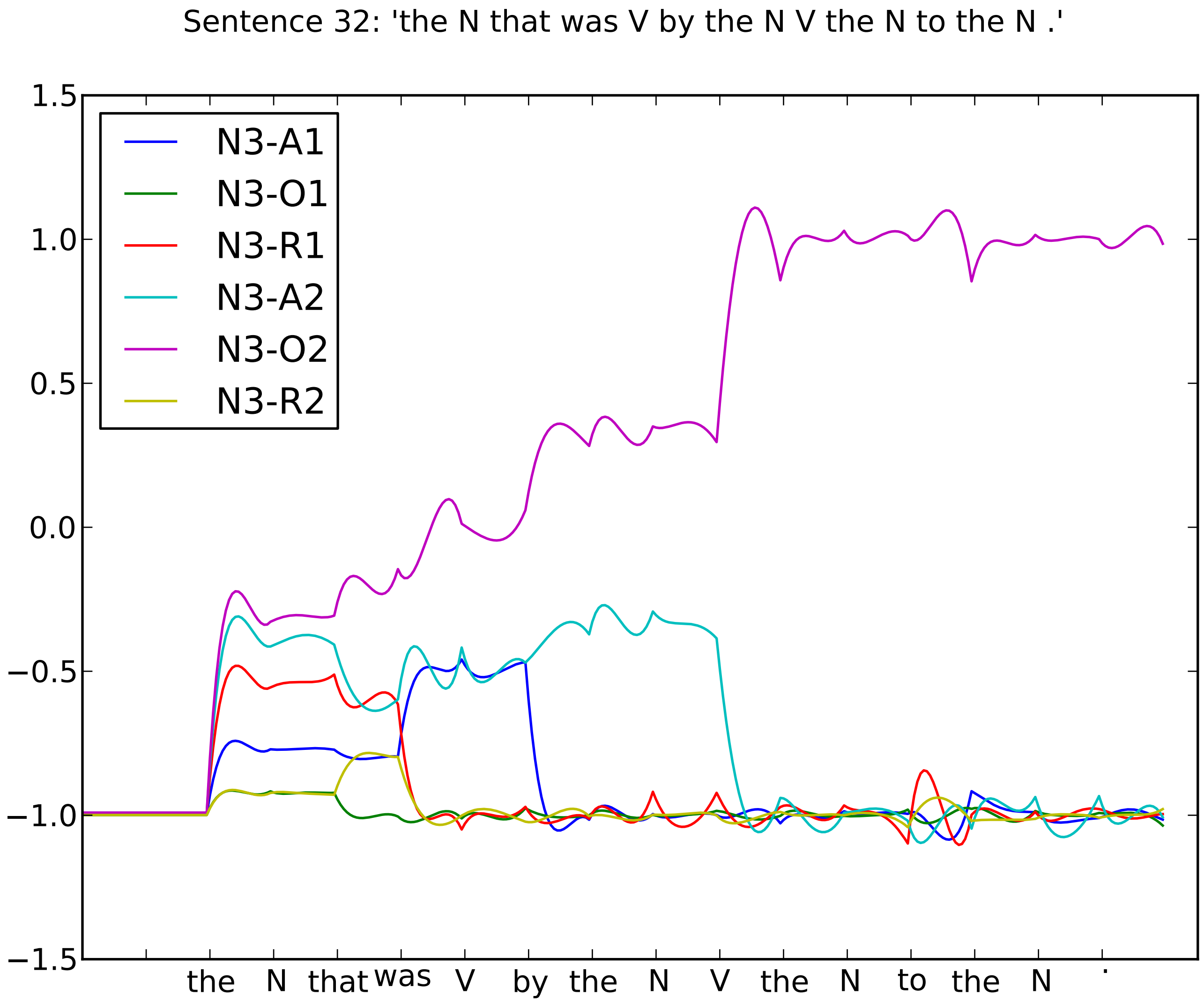
Sentence 32: 'the N that was V by the N V the N to the N .'
N3-A1
N3-O1
N3-R1
N3-A2
N3-O2
N3-R2
1.5
1.0
0.5
0.0
−0.5
−1.0
−1.5
the
N
that
was
V
by
the
N
V
the
N
to
the
N
.

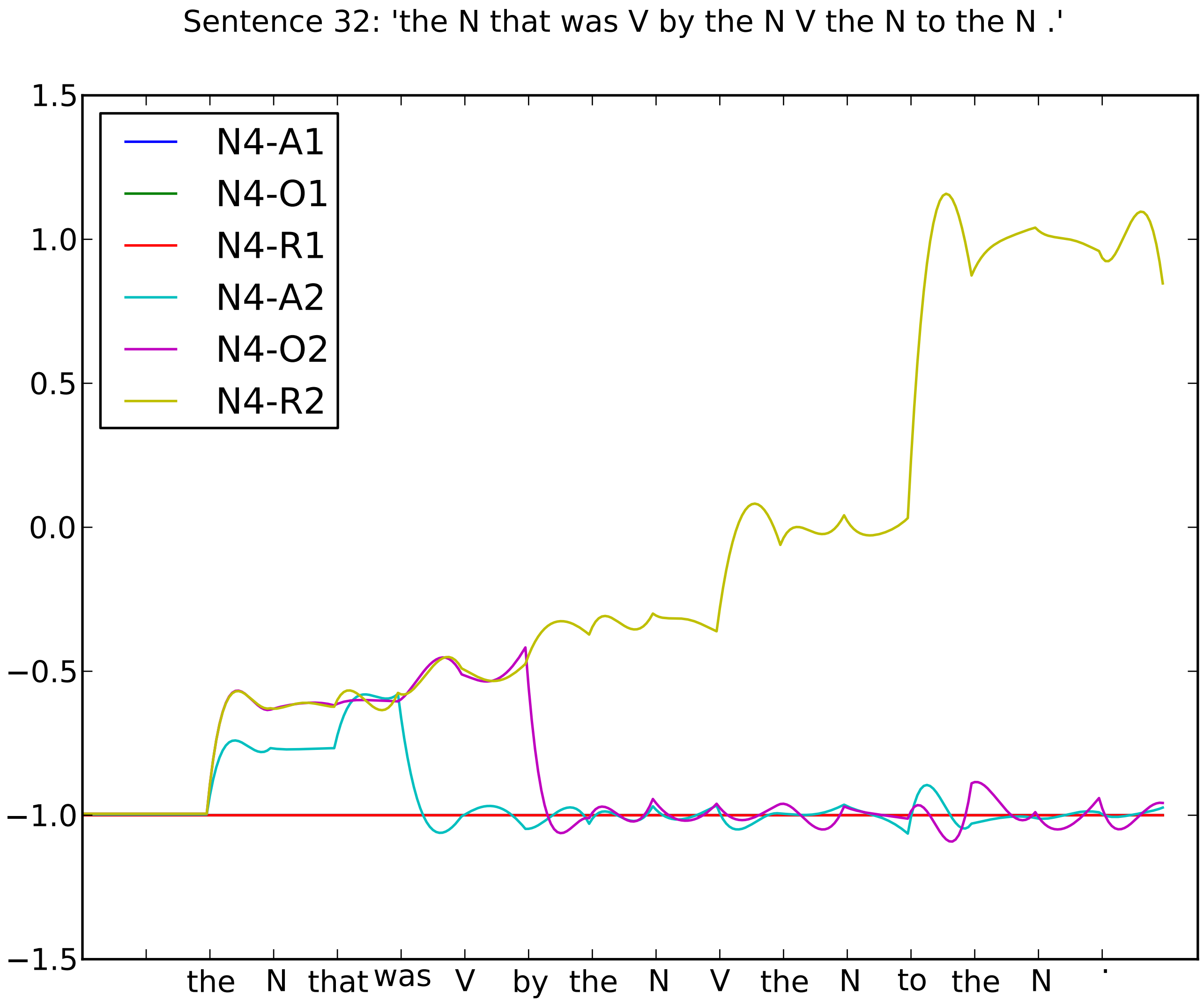
Sentence 32: 'the N that was V by the N V the N to the N .'
N4-A1
N4-O1
N4-R1
N4-A2
N4-O2
N4-R2
1.5
1.0
0.5
0.0
−0.5
−1.0
−1.5
the
N
that
was
V
by
the
N
V
the
N
to
the
N
.

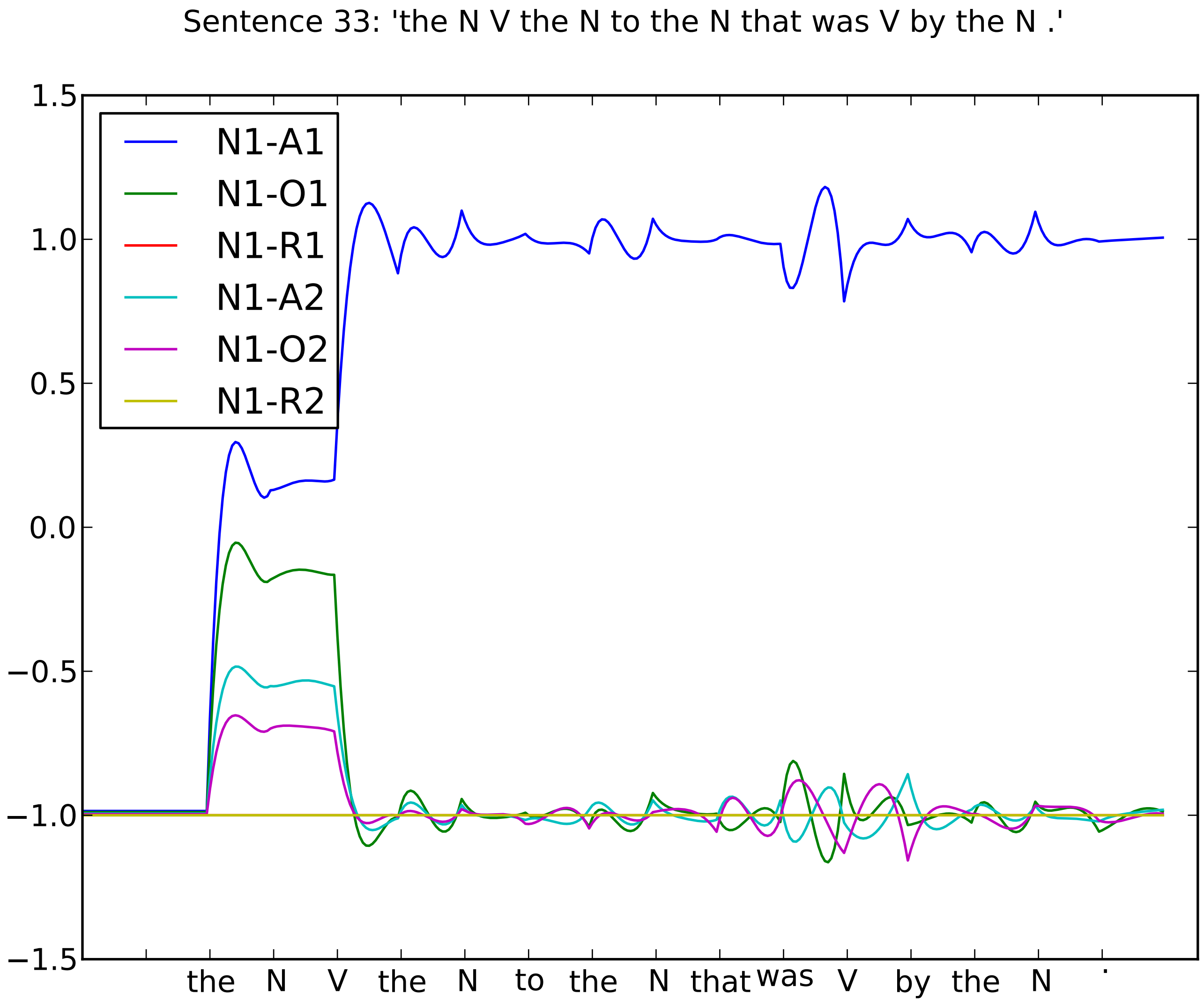

Sentence 33: 'the N V the N to the N that was V by the N .'
N1-A1
N1-O1
N1-R1
N1-A2
N1-O2
N1-R2
1.5
1.0
0.5
0.0
−0.5
−1.0
−1.5
the
N
V
the
N
to
the
N
that
was
V
by
the
N
.

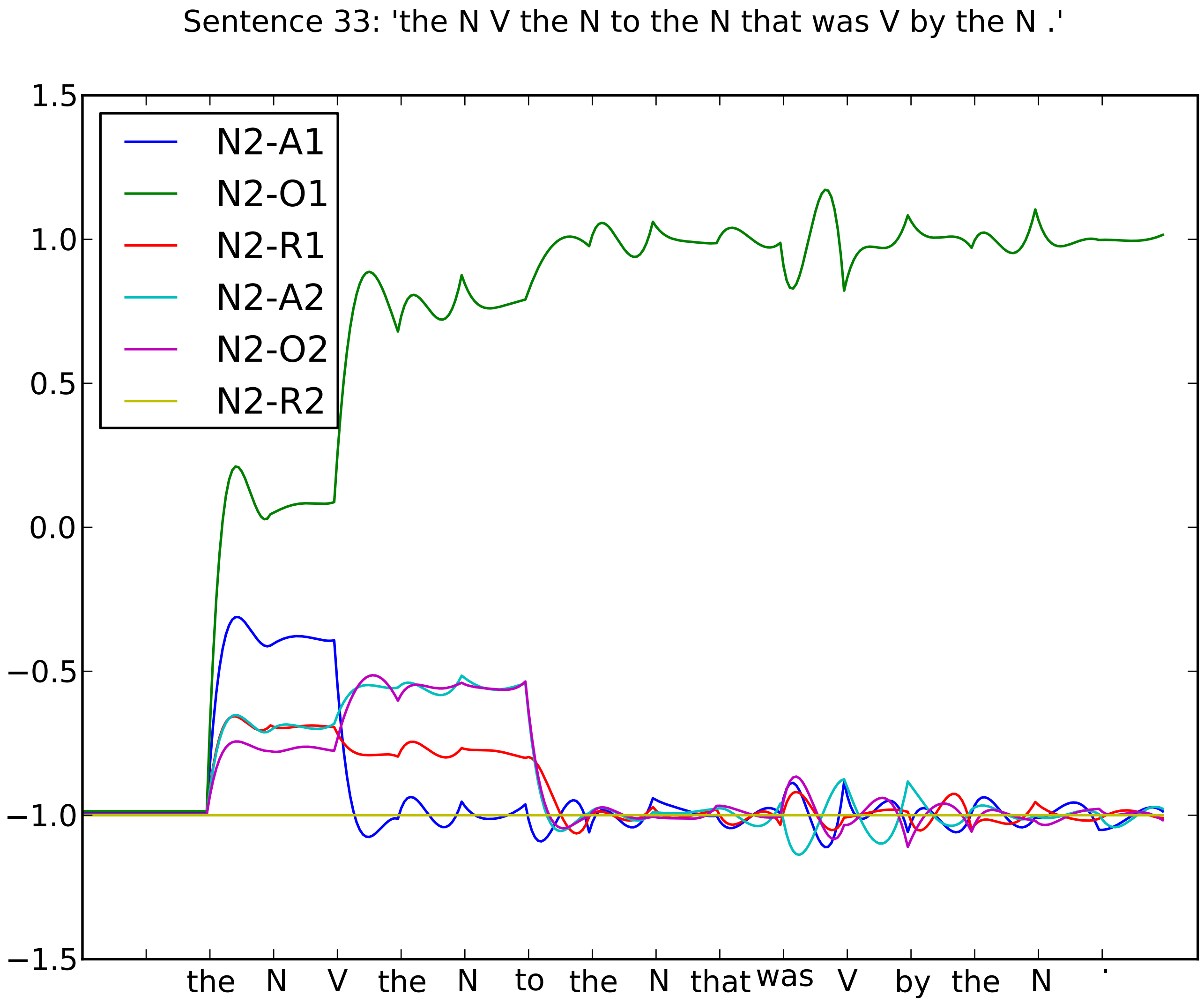

Sentence 33: 'the N V the N to the N that was V by the N .'
1.5
1.0
0.5
0.0
−0.5
−1.0
−1.5
N2-A1
N2-O1
N2-R1
N2-A2
N2-O2
N2-R2
the N V the N to the N that was V by the N .

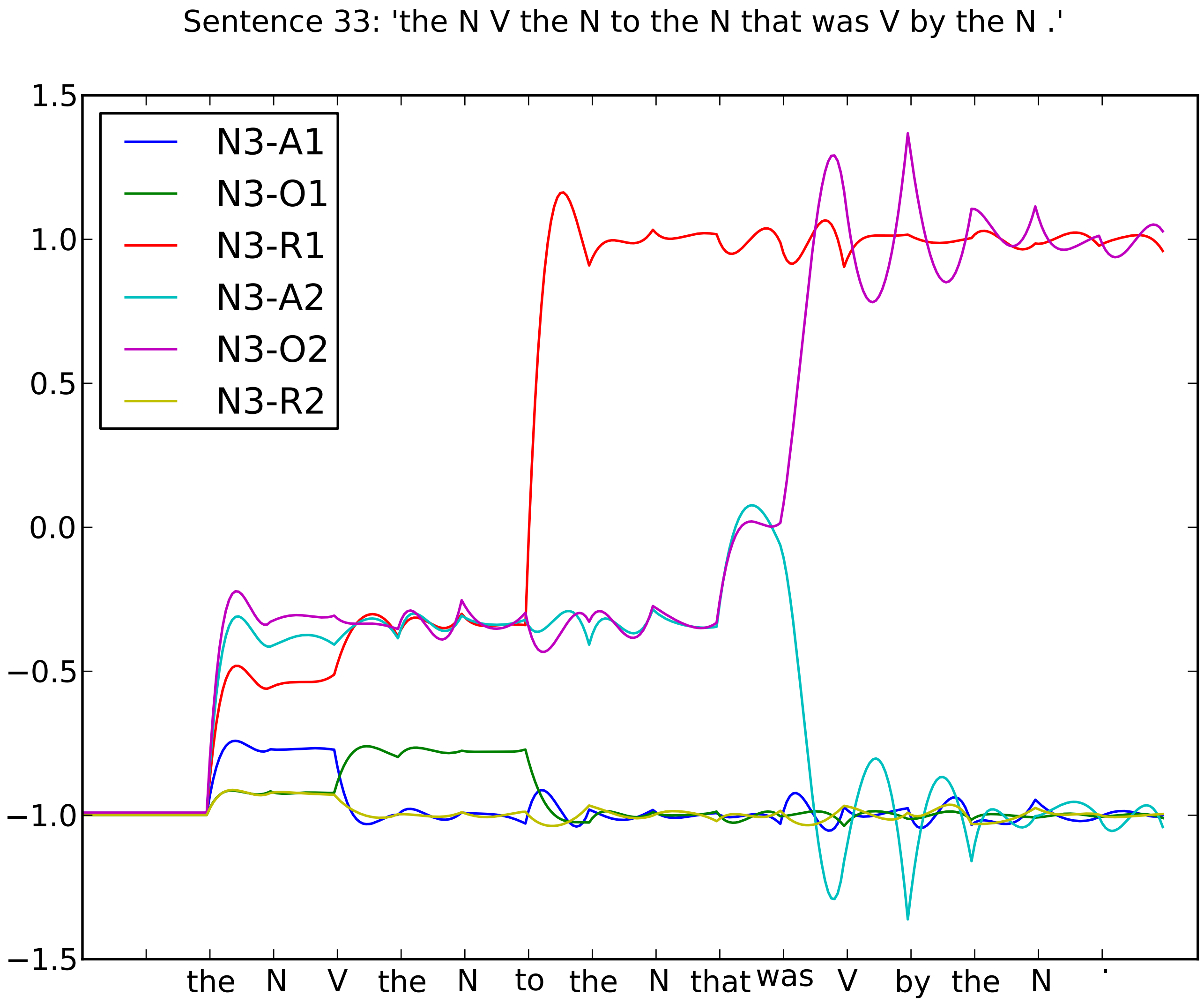
Sentence 33: 'the N V the N to the N that was V by the N .'
1.5
1.0
0.5
0.0
−0.5
−1.0
−1.5
N3-A1
N3-O1
N3-R1
N3-A2
N3-O2
N3-R2
the
N
V
the
N
to
the
N
that
was
V
by
the
N
.

Sentence 33: 'the N V the N to the N that was V by the N .'

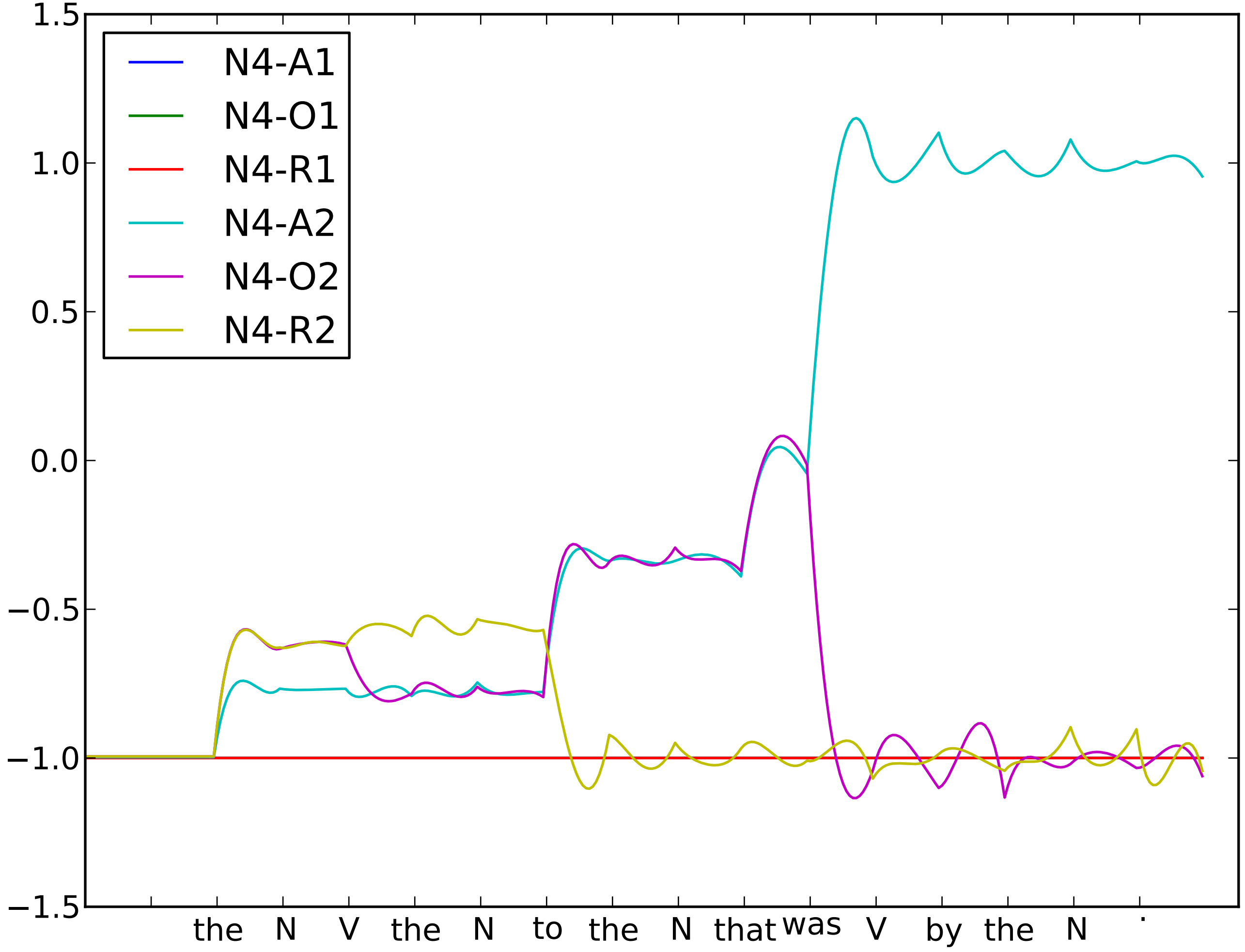

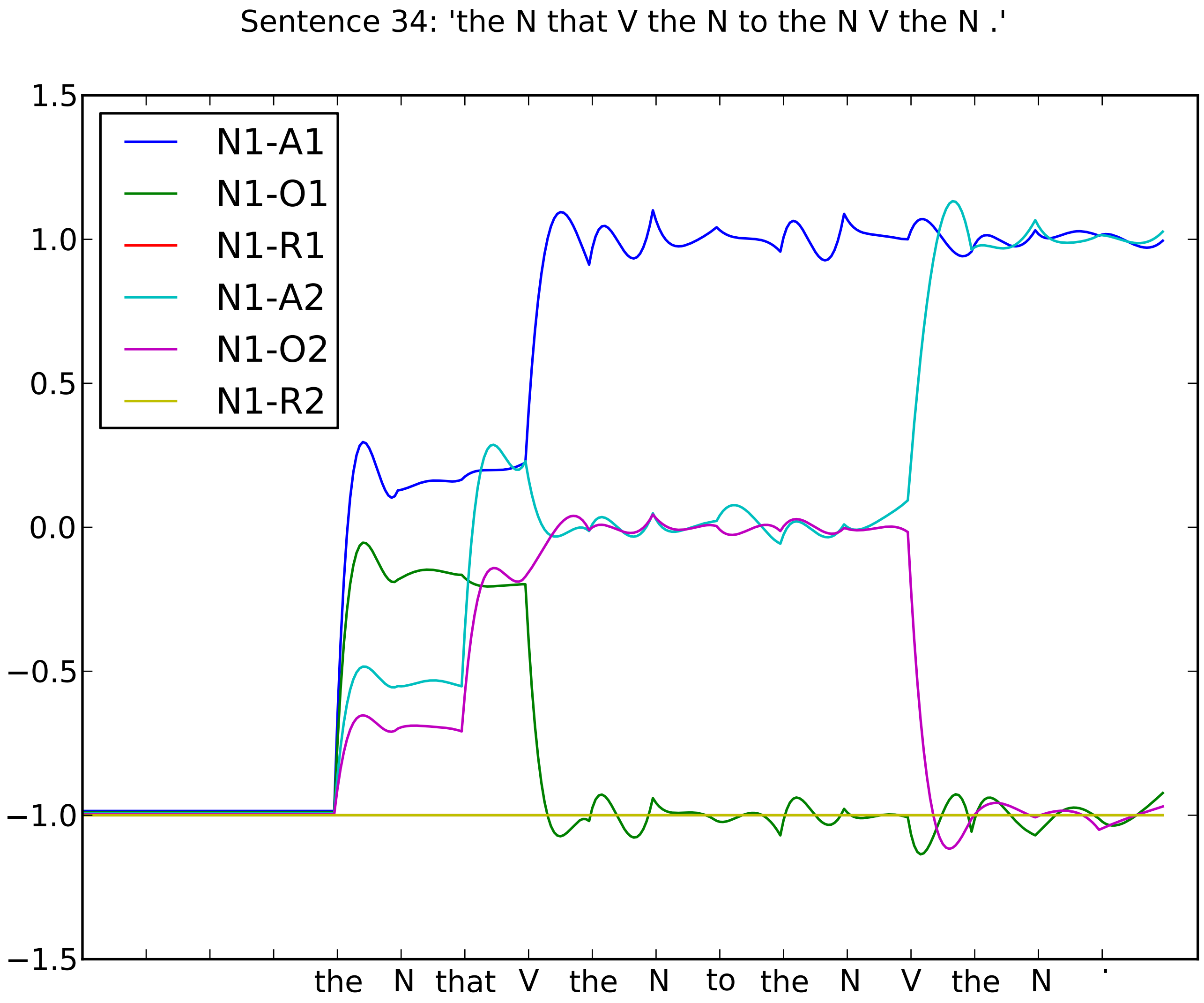
Sentence 34: 'the N that V the N to the N V the N .'
N1-A1
N1-O1
N1-R1
N1-A2
N1-O2
N1-R2
1.5
1.0
0.5
0.0
−0.5
−1.0
−1.5
the N that V the N to the N V the N .

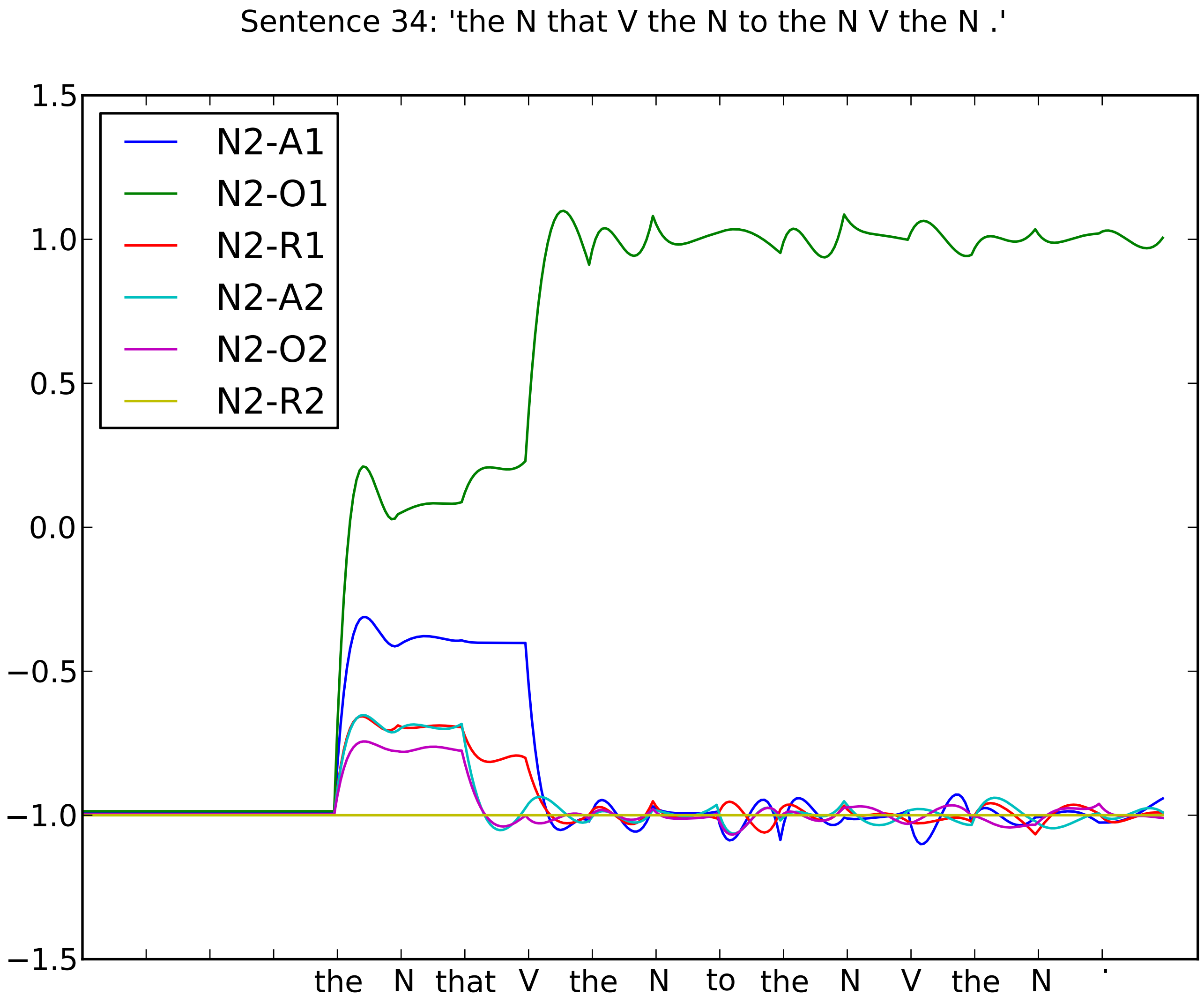
Sentence 34: 'the N that V the N to the N V the N .'
1.5
1.0
0.5
0.0
−0.5
−1.0
−1.5
N2-A1
N2-O1
N2-R1
N2-A2
N2-O2
N2-R2
the
N
that
V
the
N
to
the
N
V
the
N
.

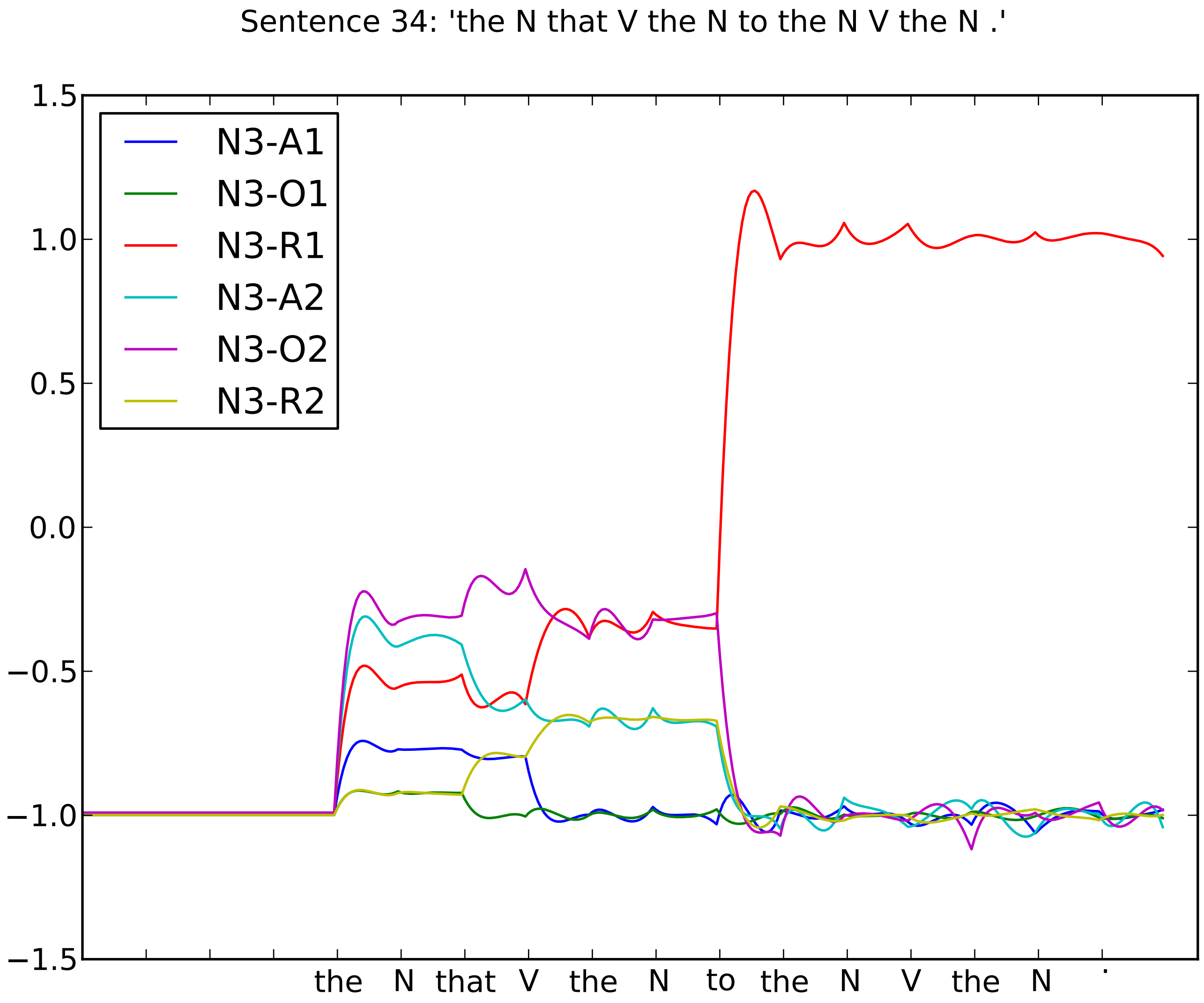

Sentence 34: 'the N that V the N to the N V the N .'
1.5
1.0
0.5
0.0
−0.5
−1.0
−1.5
N3-A1
N3-O1
N3-R1
N3-A2
N3-O2
N3-R2
the
N
that
V
the
N
to
the
N
V
the
N
.

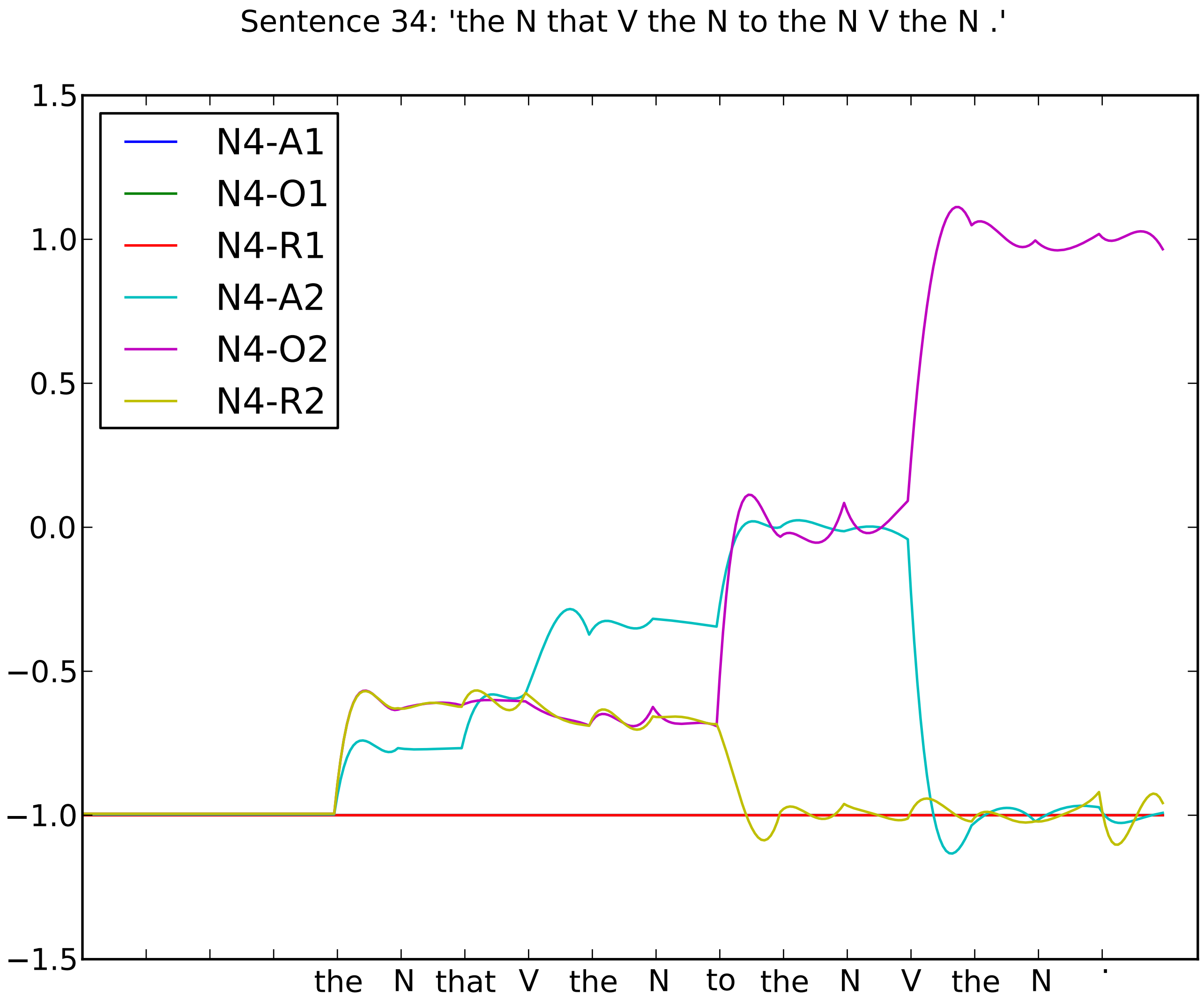
Sentence 34: 'the N that V the N to the N V the N .'
1.5
1.0
0.5
0.0
−0.5
−1.0
−1.5
N4-A1
N4-O1
N4-R1
N4-A2
N4-O2
N4-R2
the N that V the N to the N V the N .

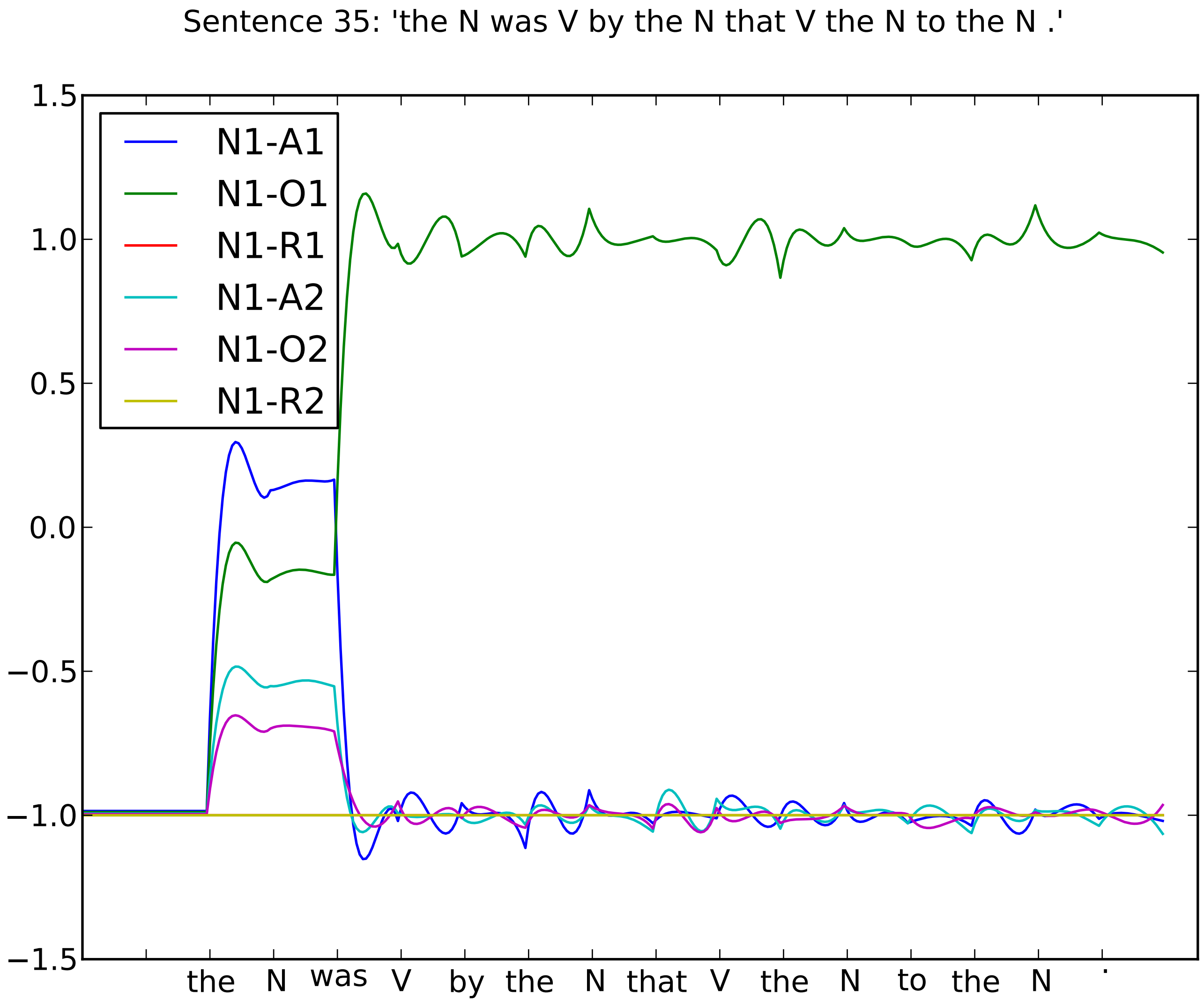
Sentence 35: 'the N was V by the N that V the N to the N .'
1.5
1.0
0.5
0.0
−0.5
−1.0
−1.5
N1-A1
N1-O1
N1-R1
N1-A2
N1-O2
N1-R2
the
N
was
V
by
the
N
that
V
the
N
to
the
N
.

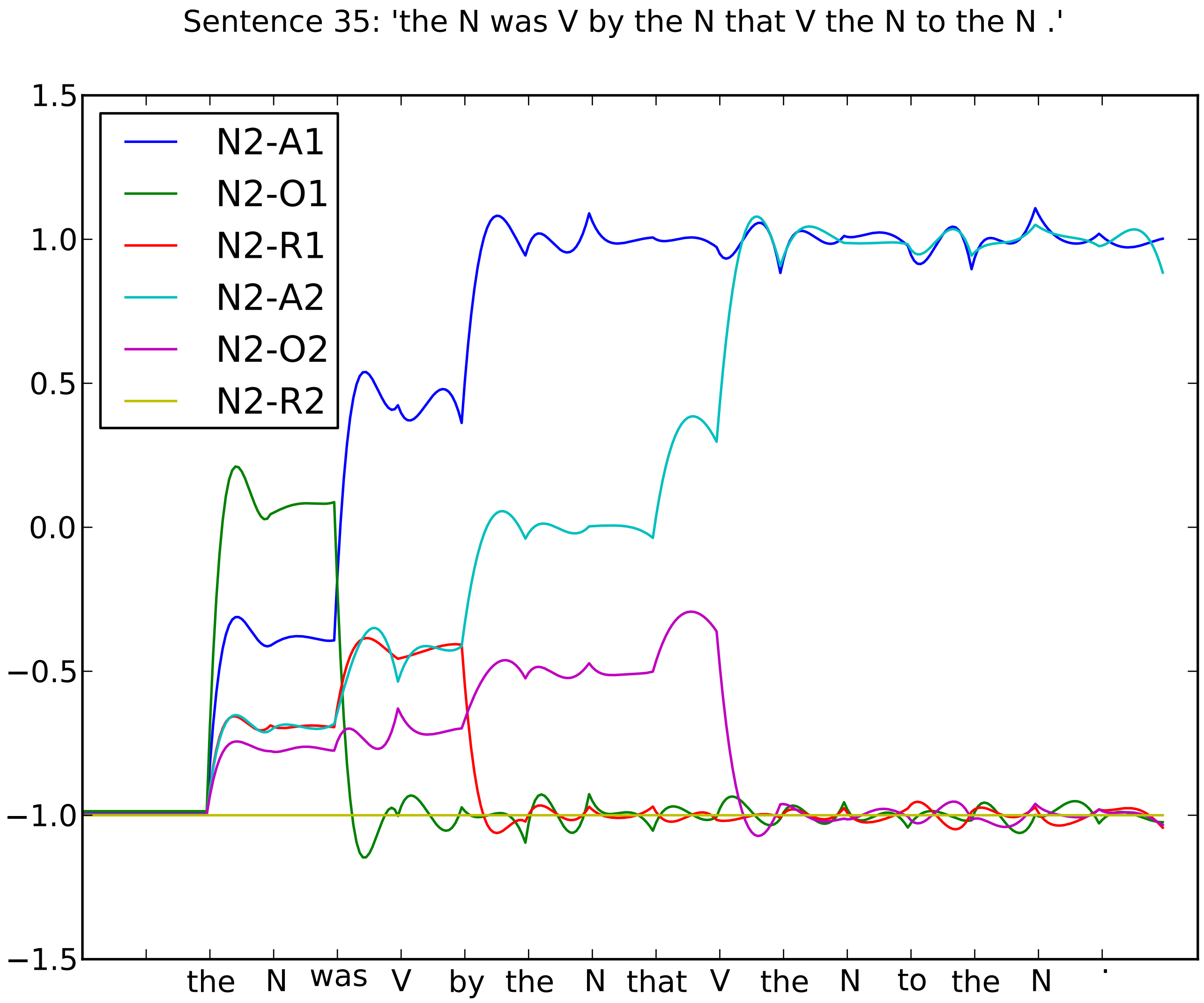

Sentence 35: 'the N was V by the N that V the N to the N .'
N2-A1
N2-O1
N2-R1
N2-A2
N2-O2
N2-R2
1.5
1.0
0.5
0.0
−0.5
−1.0
−1.5
the N was V by the N that V the N to the N .

Sentence 35: 'the N was V by the N that V the N to the N .'

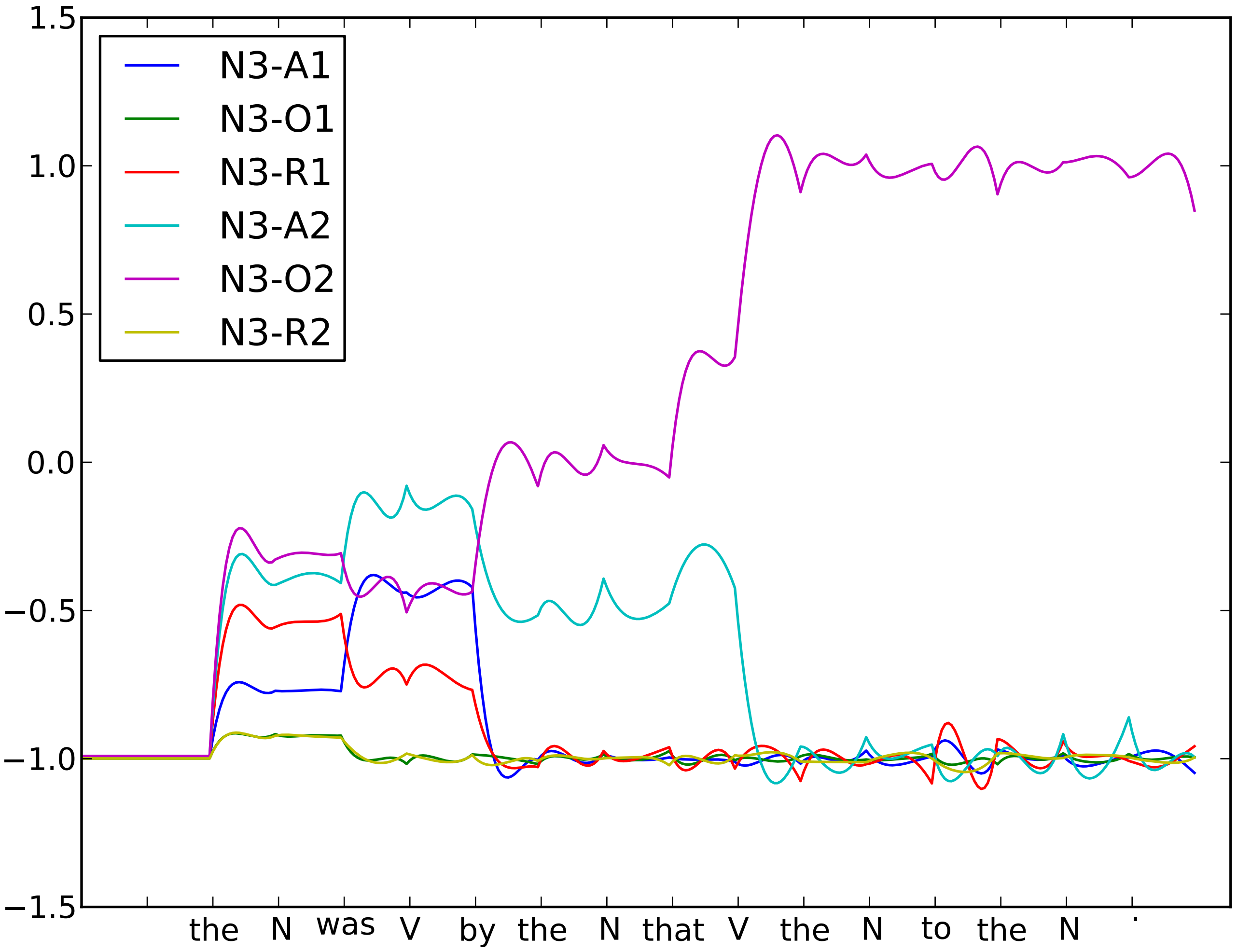

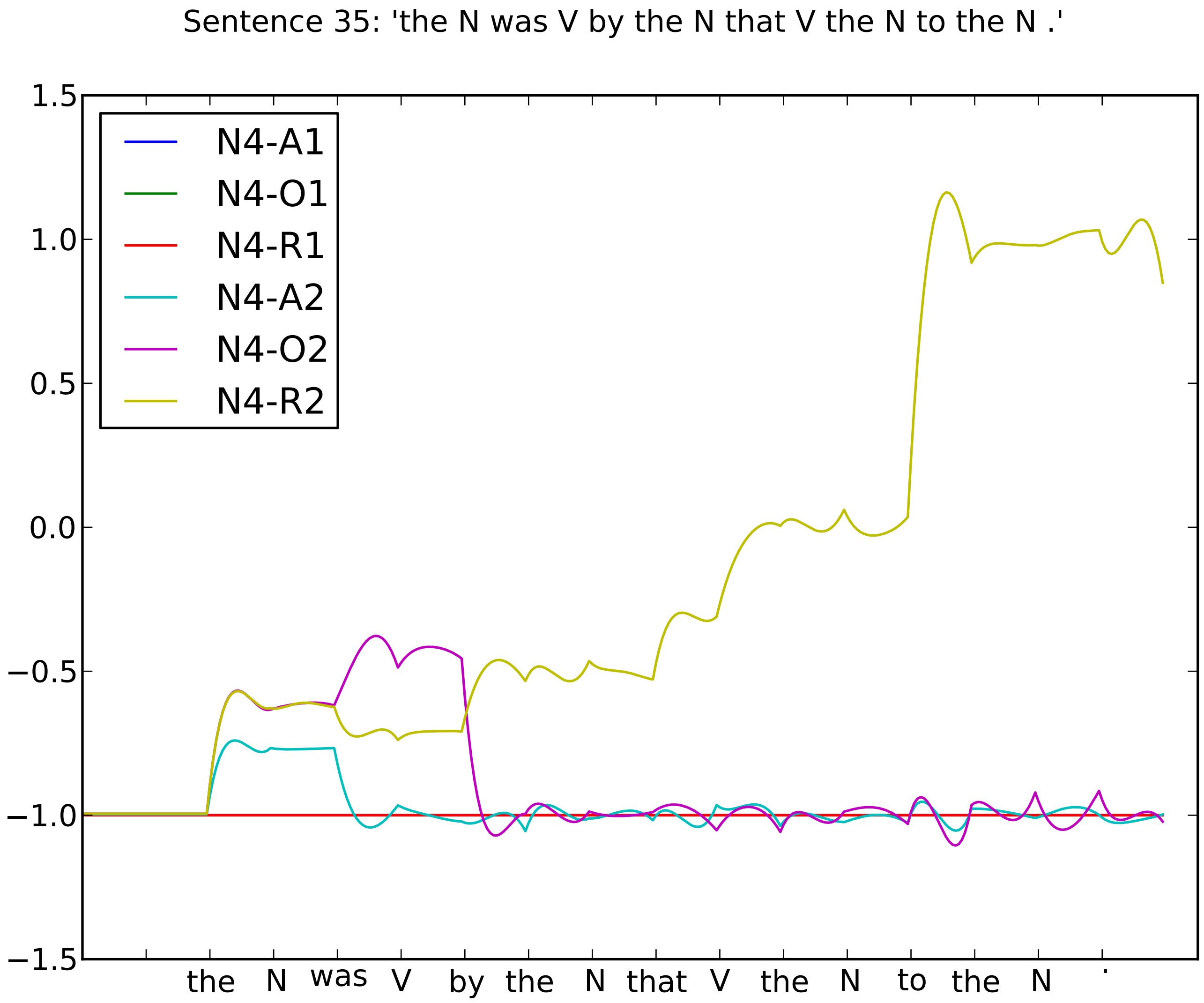
Sentence 35: 'the N was V by the N that V the N to the N .'
1.5
1.0
0.5
0.0
−0.5
−1.0
−1.5
N4-A1
N4-O1
N4-R1
N4-A2
N4-O2
N4-R2
the N was V by the N that V the N to the N .

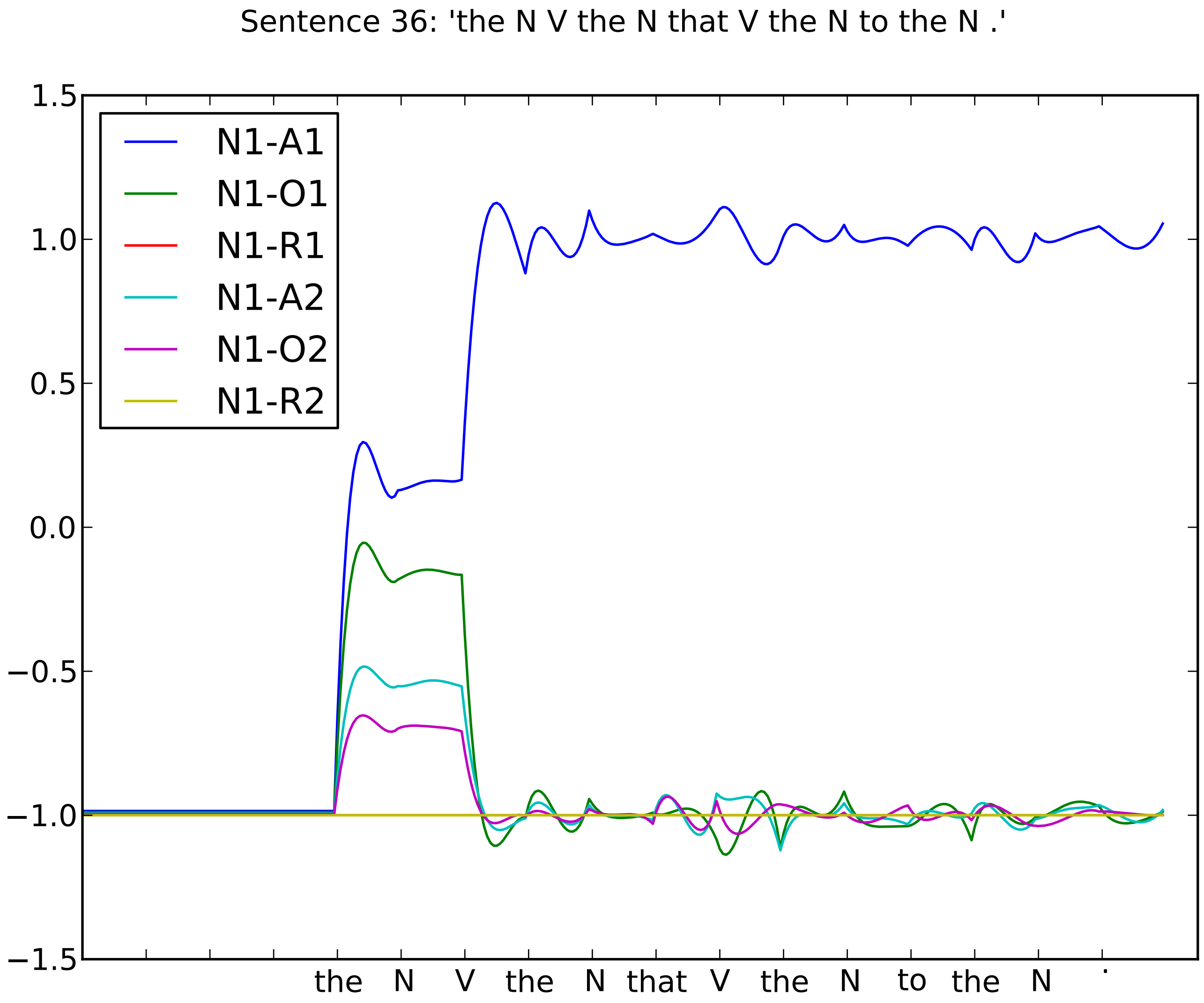

Sentence 36: 'the N V the N that V the N to the N .'
1.5
1.0
0.5
0.0
−0.5
−1.0
−1.5
N1-A1
N1-O1
N1-R1
N1-A2
N1-O2
N1-R2
the N V the N that V the N to the N .

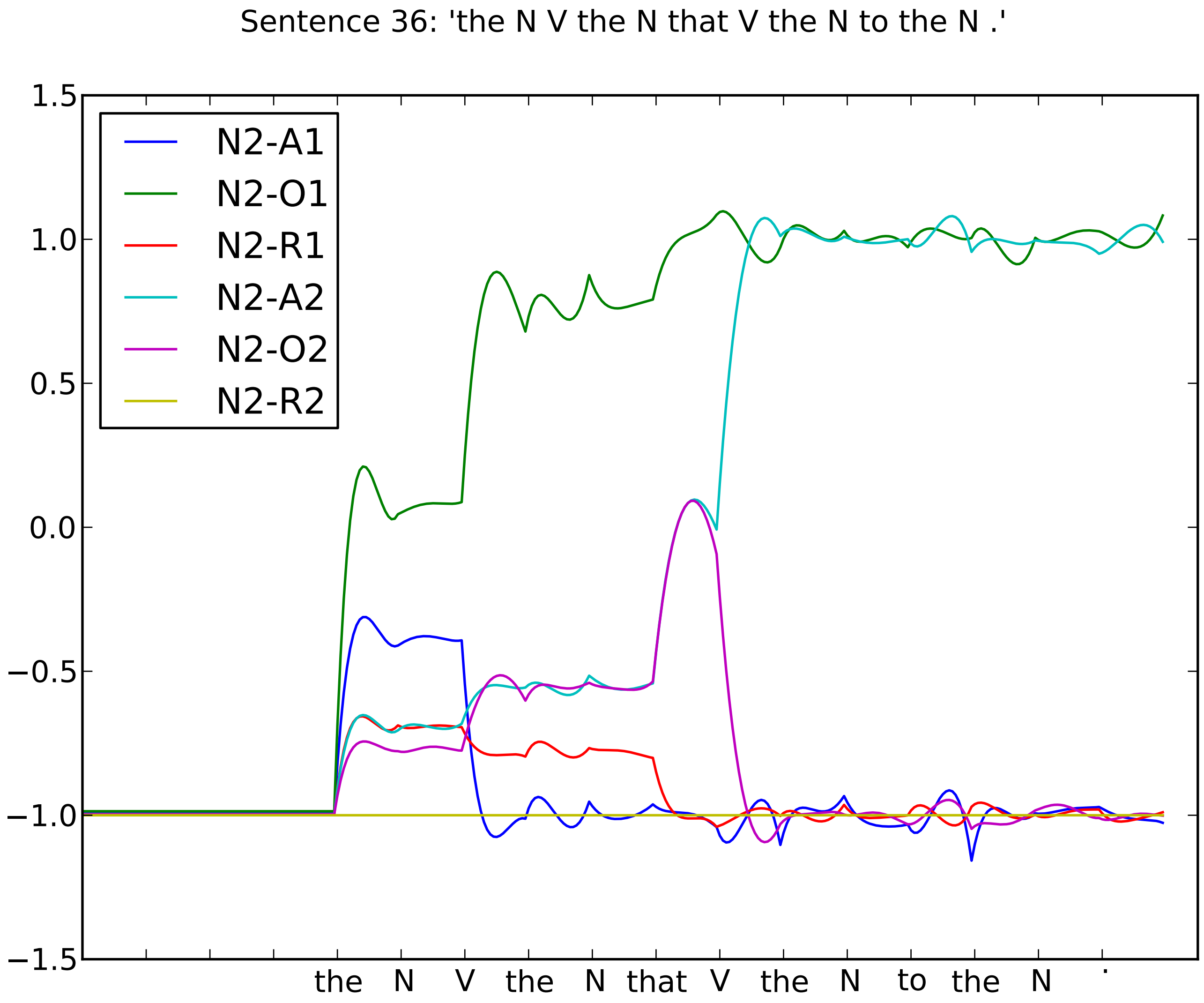
Sentence 36: 'the N V the N that V the N to the N .'
1.5
1.0
0.5
0.0
−0.5
−1.0
−1.5
N2-A1
N2-O1
N2-R1
N2-A2
N2-O2
N2-R2
the N V the N that V the N to the N .

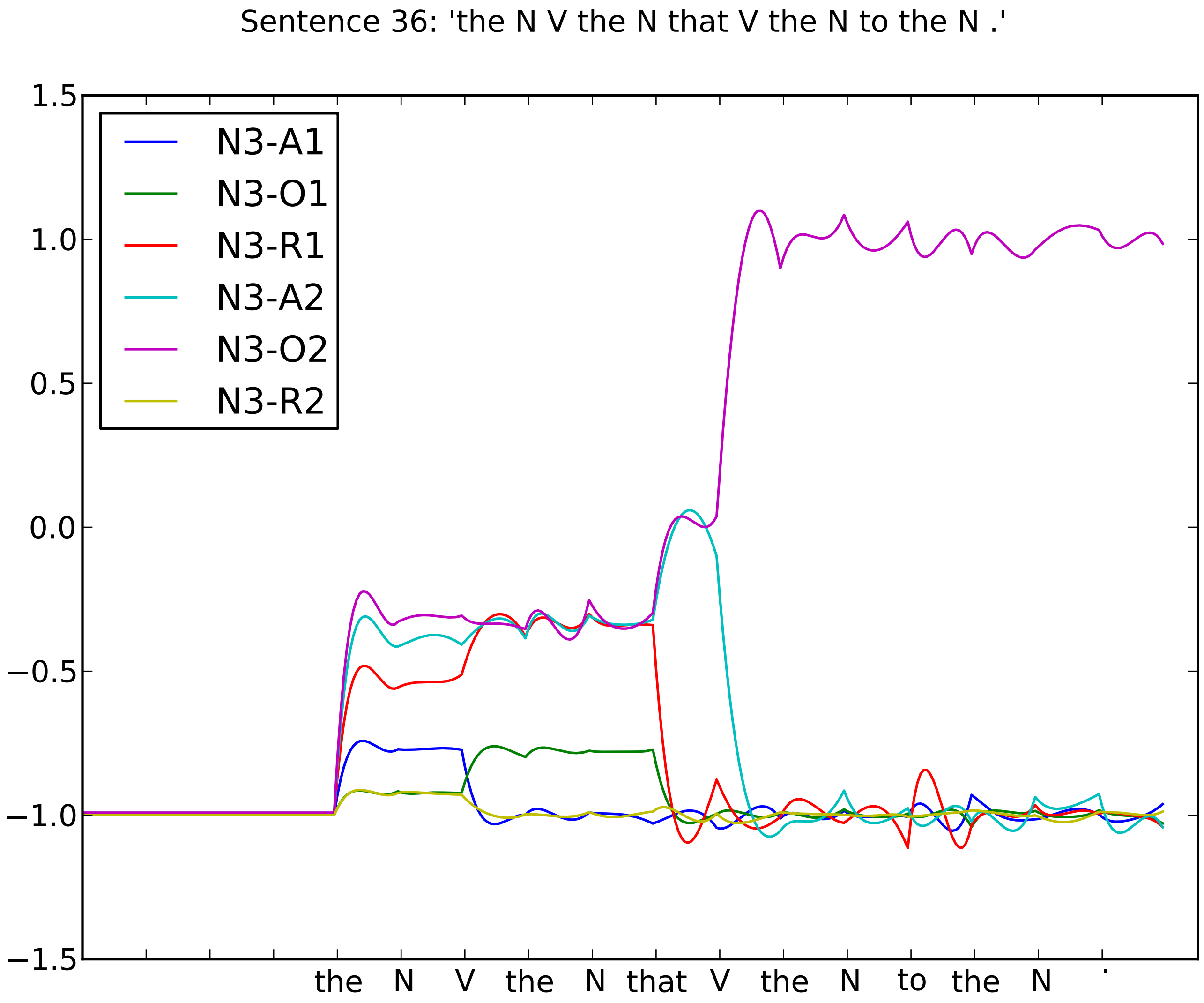

Sentence 36: 'the N V the N that V the N to the N .'
1.5
1.0
0.5
0.0
−0.5
−1.0
−1.5
N3-A1
N3-O1
N3-R1
N3-A2
N3-O2
N3-R2
the N V the N that V the N to the N .

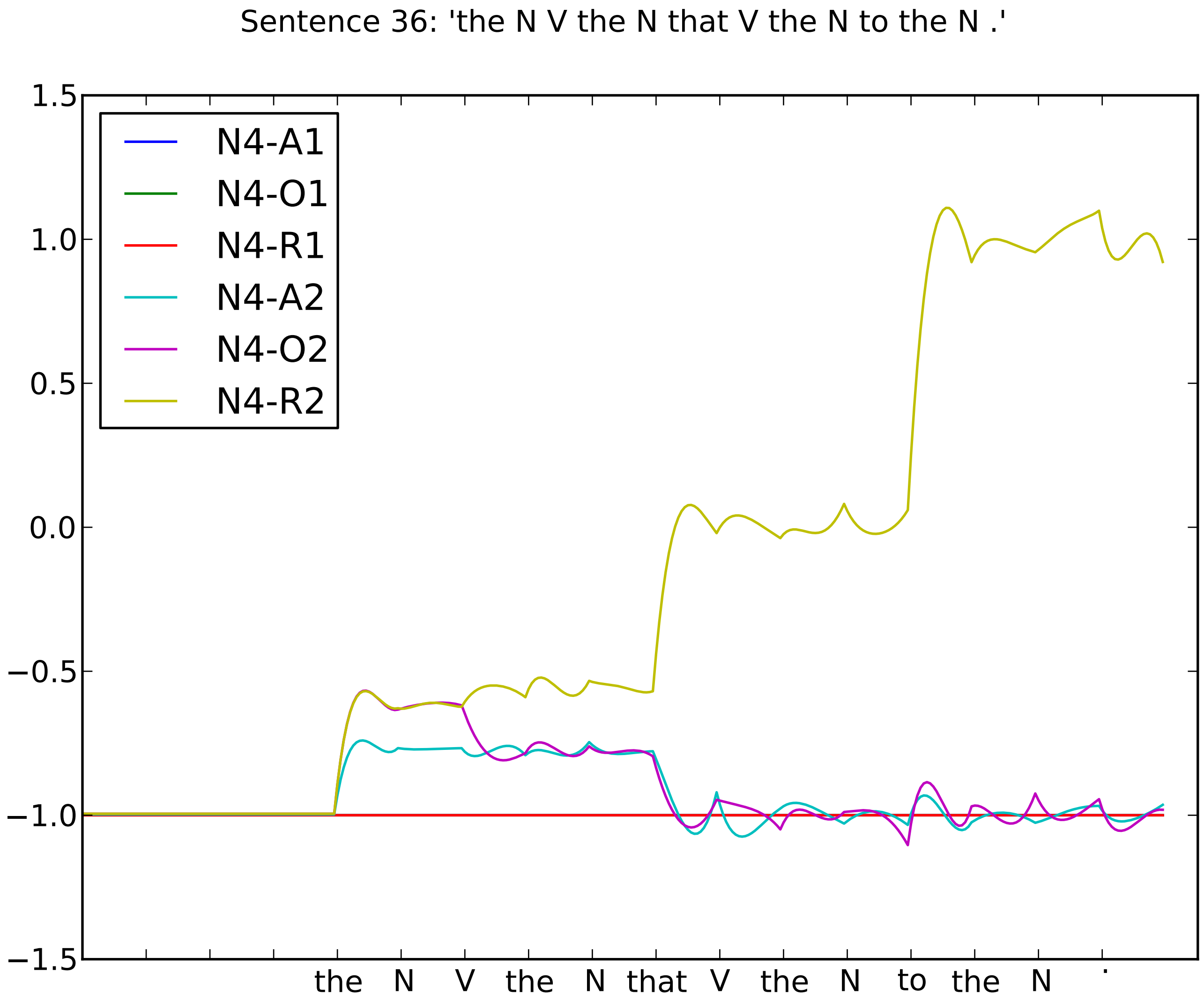
Sentence 36: 'the N V the N that V the N to the N .'
1.5
1.0
0.5
0.0
−0.5
−1.0
−1.5
N4-A1
N4-O1
N4-R1
N4-A2
N4-O2
N4-R2
the
N
V
the
N
that
V
the
N
to
the
N
.

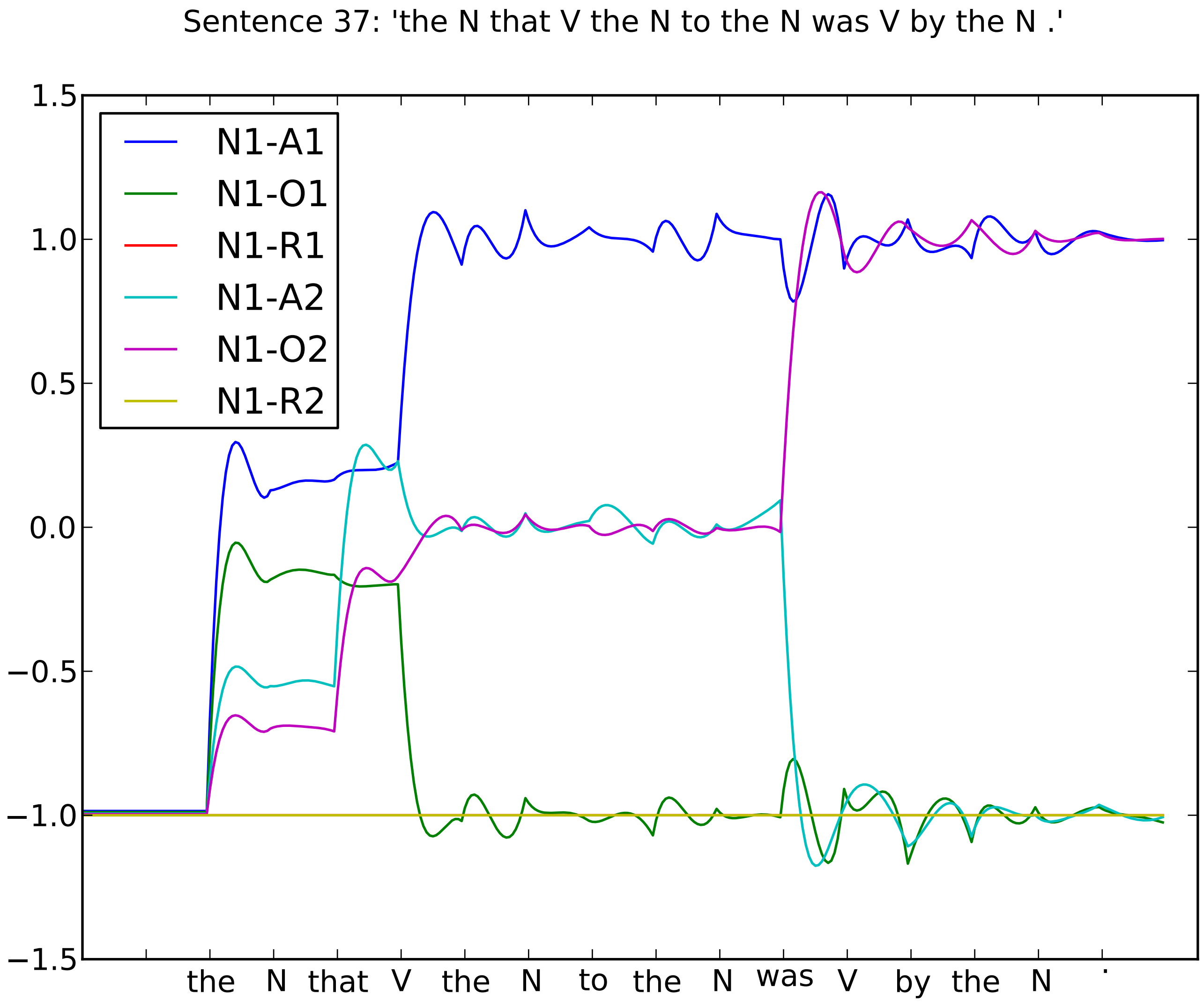

Sentence 37: 'the N that V the N to the N was V by the N .'
1.5
1.0
0.5
0.0
−0.5
−1.0
−1.5
N1-A1
N1-O1
N1-R1
N1-A2
N1-O2
N1-R2
the
N
that
V
the
N
to
the
N
was
V
by
the
N
.

Sentence 37: 'the N that V the N to the N was V by the N .'

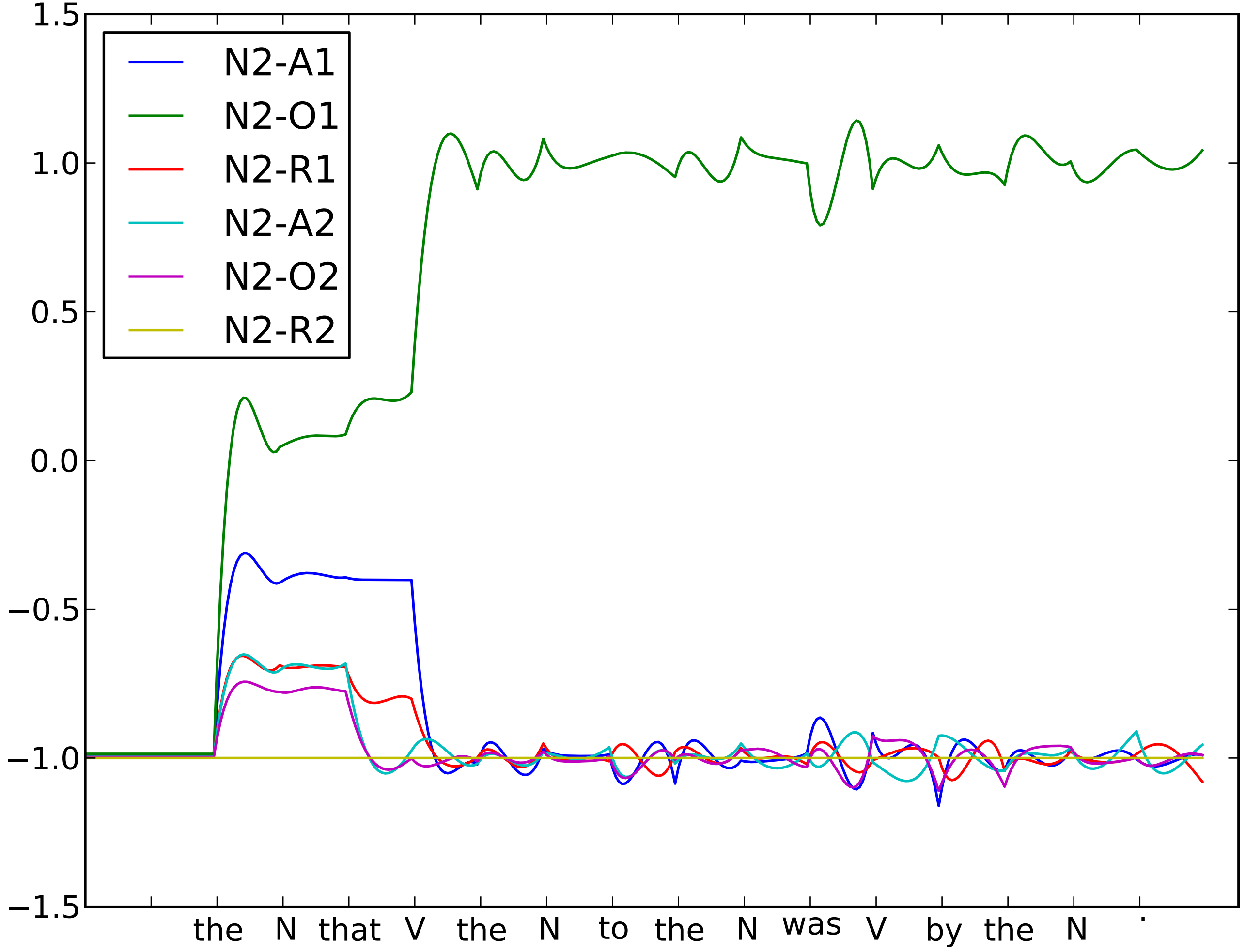

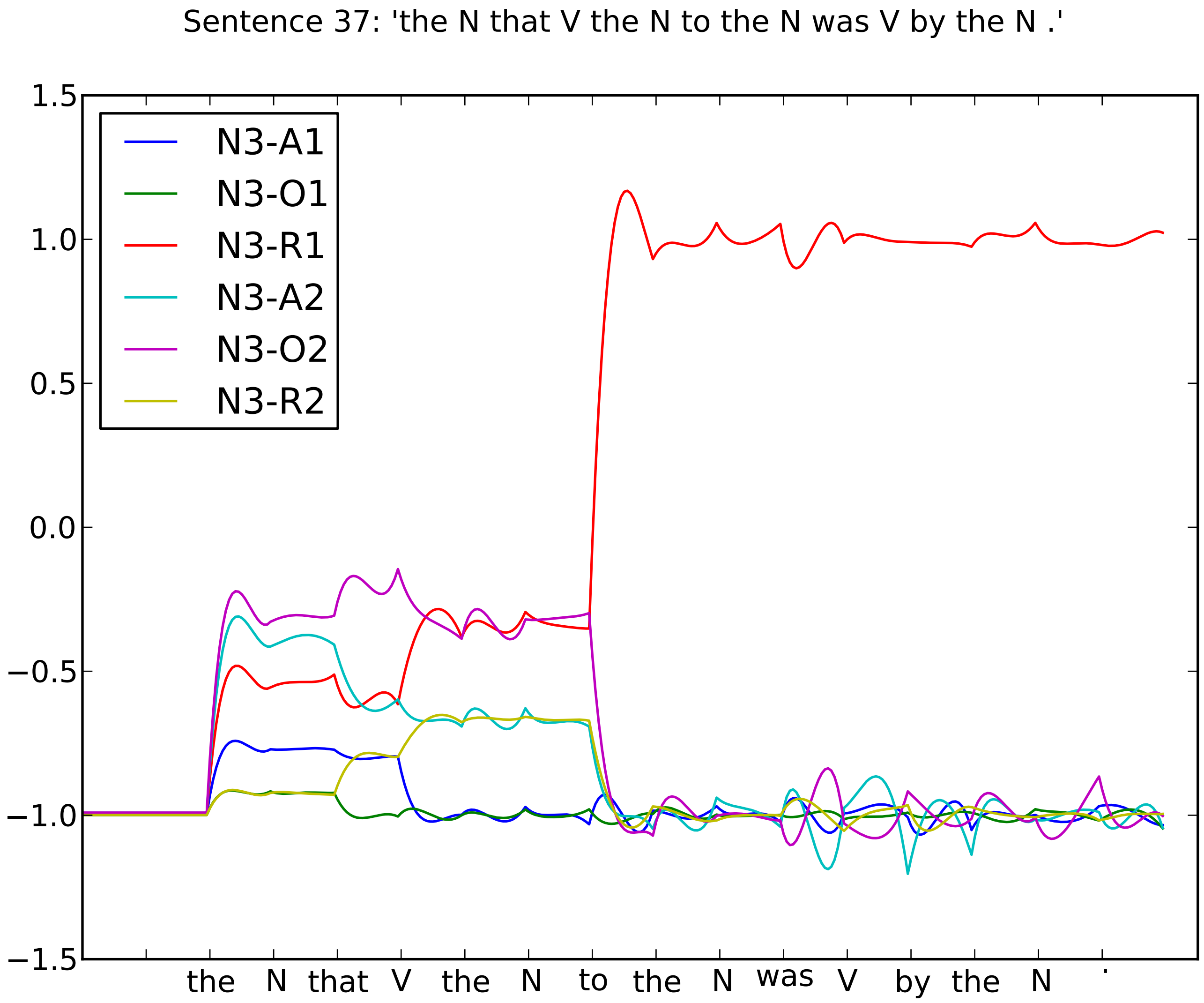

Sentence 37: 'the N that V the N to the N was V by the N .'
1.5
1.0
0.5
0.0
−0.5
−1.0
−1.5
N3-A1
N3-O1
N3-R1
N3-A2
N3-O2
N3-R2
the N that V the N to the N was V by the N .

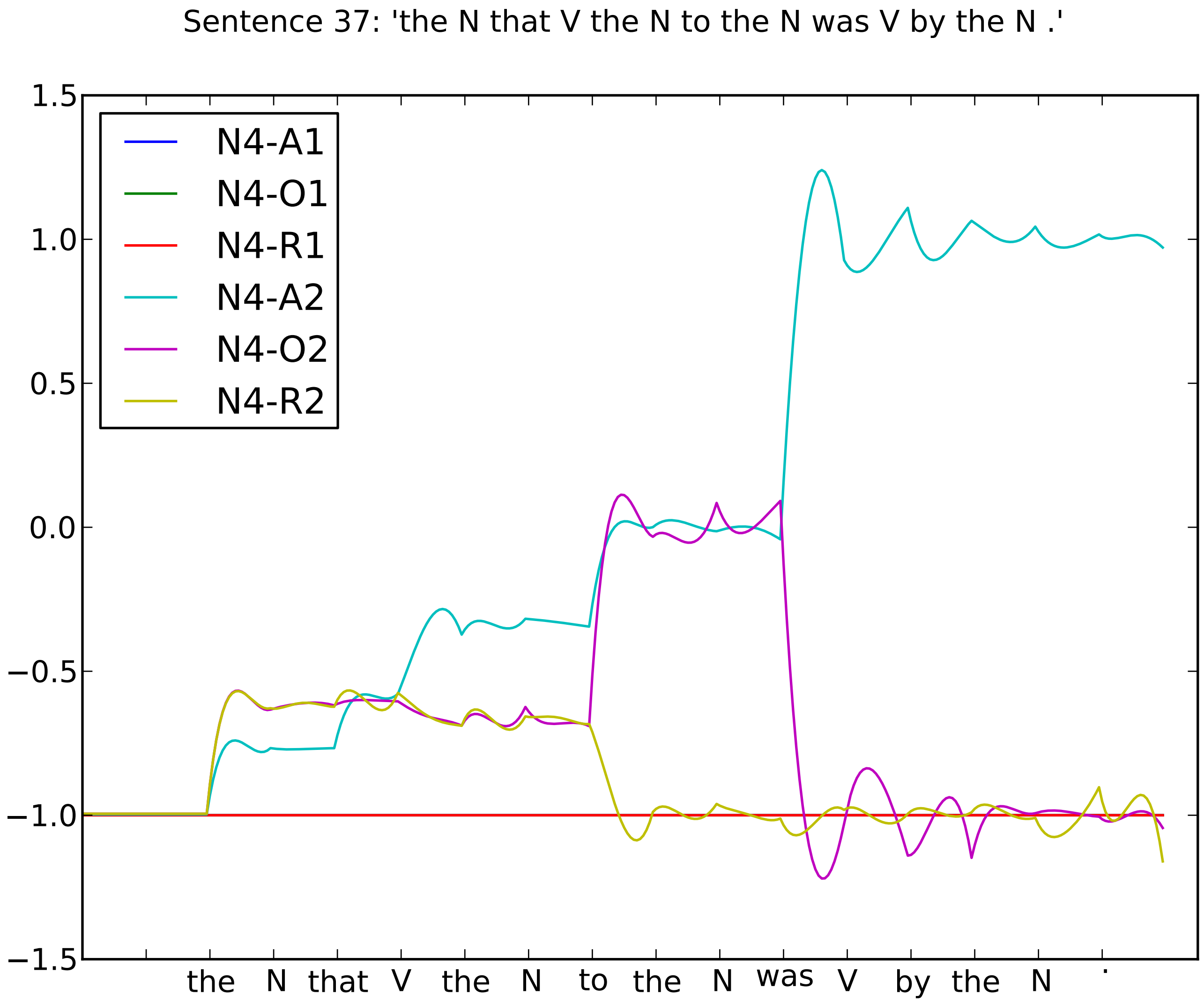
Sentence 37: 'the N that V the N to the N was V by the N .'
1.5
1.0
0.5
0.0
−0.5
−1.0
−1.5
N4-A1
N4-O1
N4-R1
N4-A2
N4-O2
N4-R2
the N that V the N to the N was V by the N .

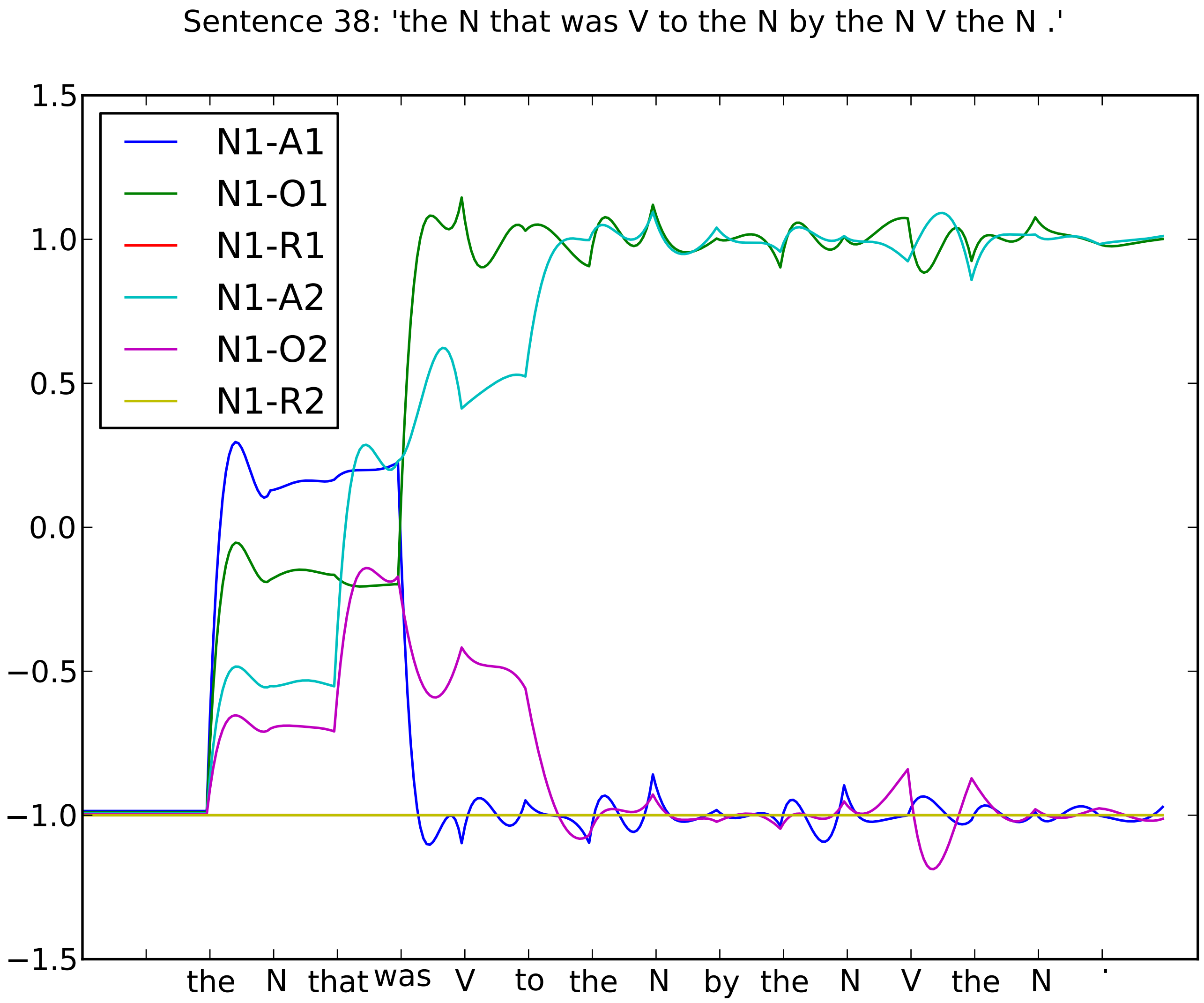
Sentence 38: 'the N that was V to the N by the N V the N .'
N1-A1
N1-O1
N1-R1
N1-A2
N1-O2
N1-R2
1.5
1.0
0.5
0.0
−0.5
−1.0
−1.5
the N that was V to the N by the N V the N .

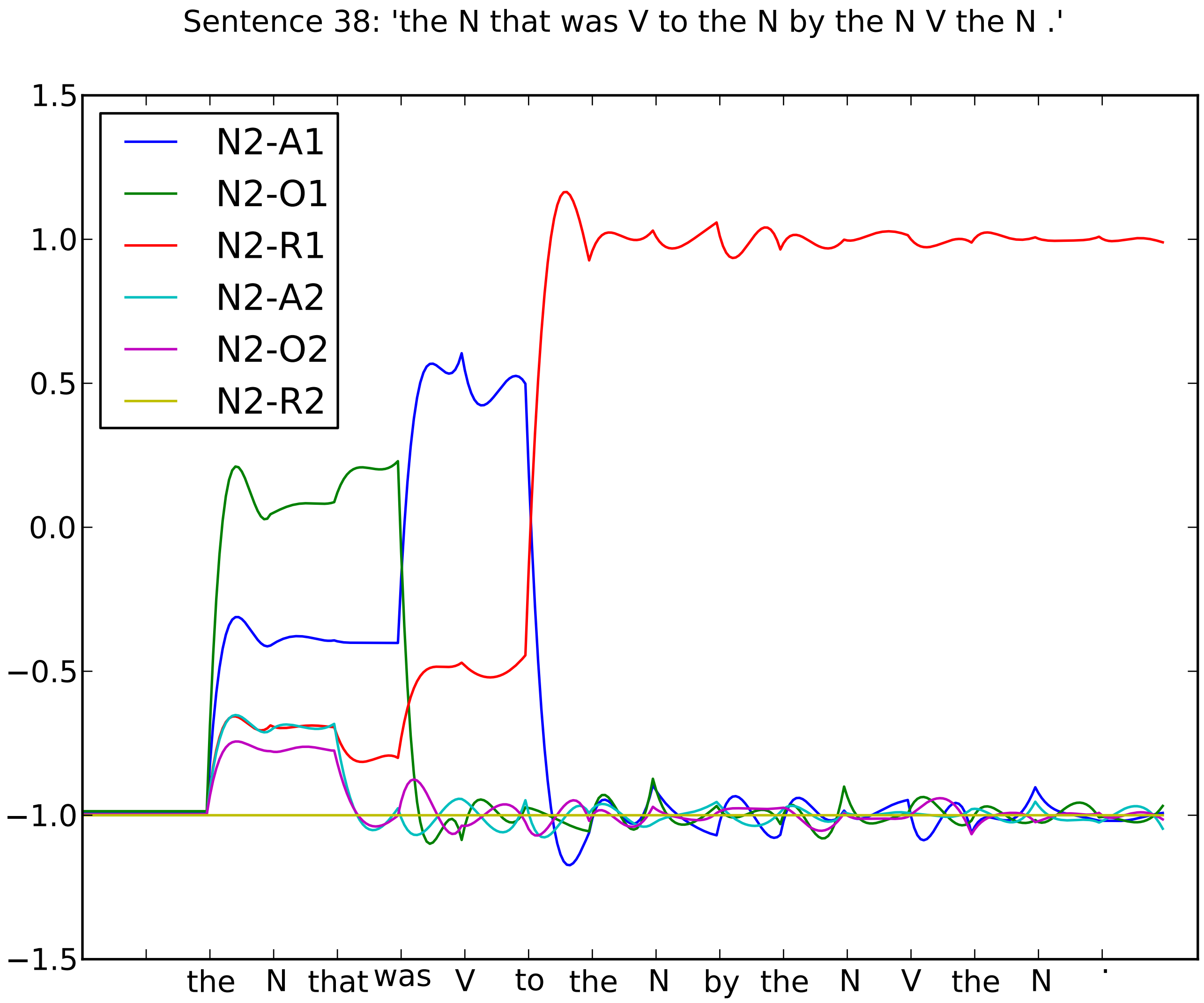
Sentence 38: 'the N that was V to the N by the N V the N .'
N2-A1
N2-O1
N2-R1
N2-A2
N2-O2
N2-R2
1.5
1.0
0.5
0.0
−0.5
−1.0
−1.5
the
N
that
was
V
to
the
N
by
the
N
V
the
N
.

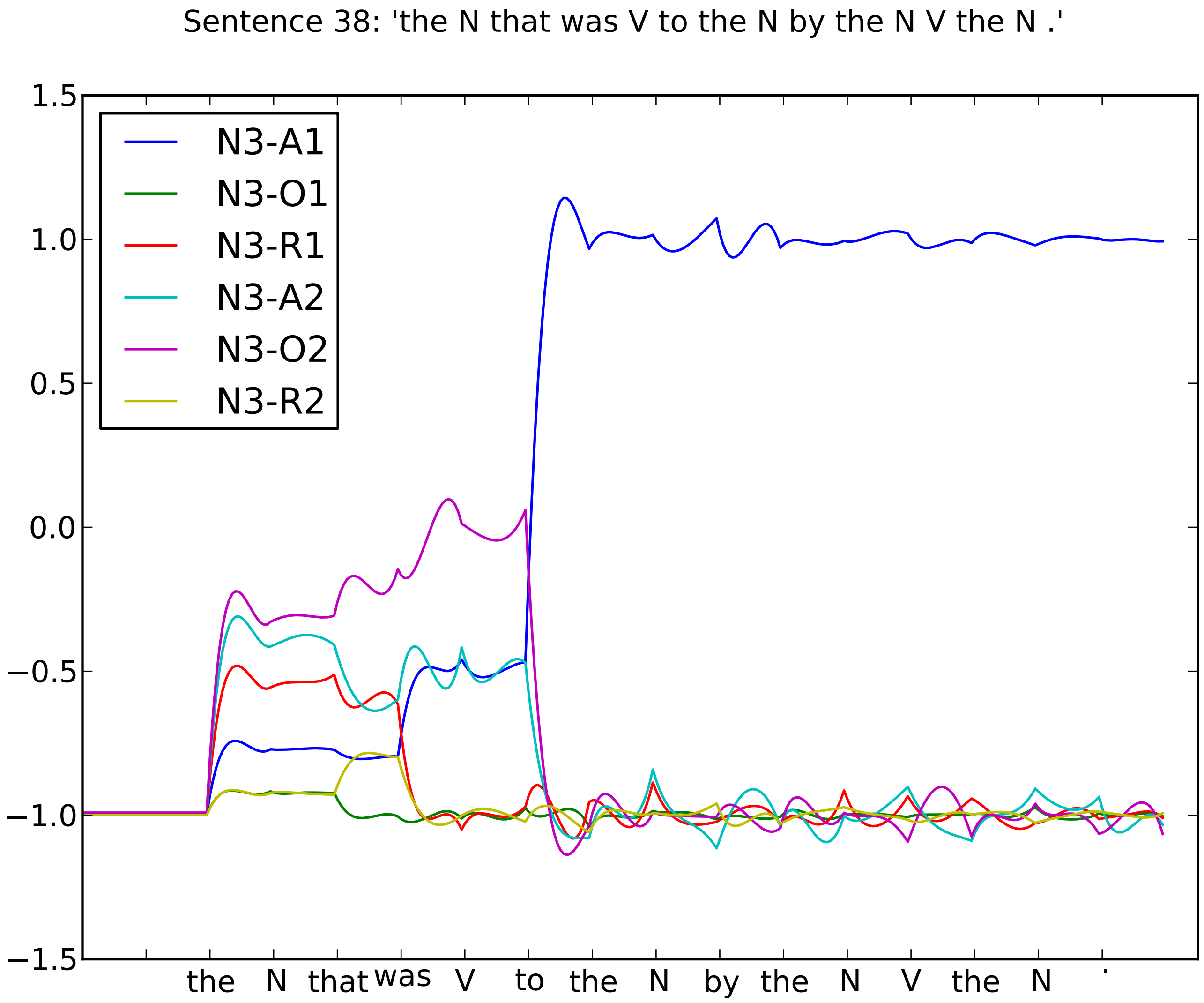
Sentence 38: 'the N that was V to the N by the N V the N .'
1.5
1.0
0.5
0.0
−0.5
−1.0
−1.5
N3-A1
N3-O1
N3-R1
N3-A2
N3-O2
N3-R2
the
N
that
was
V
to
the
N
by
the
N
V
the
N
.

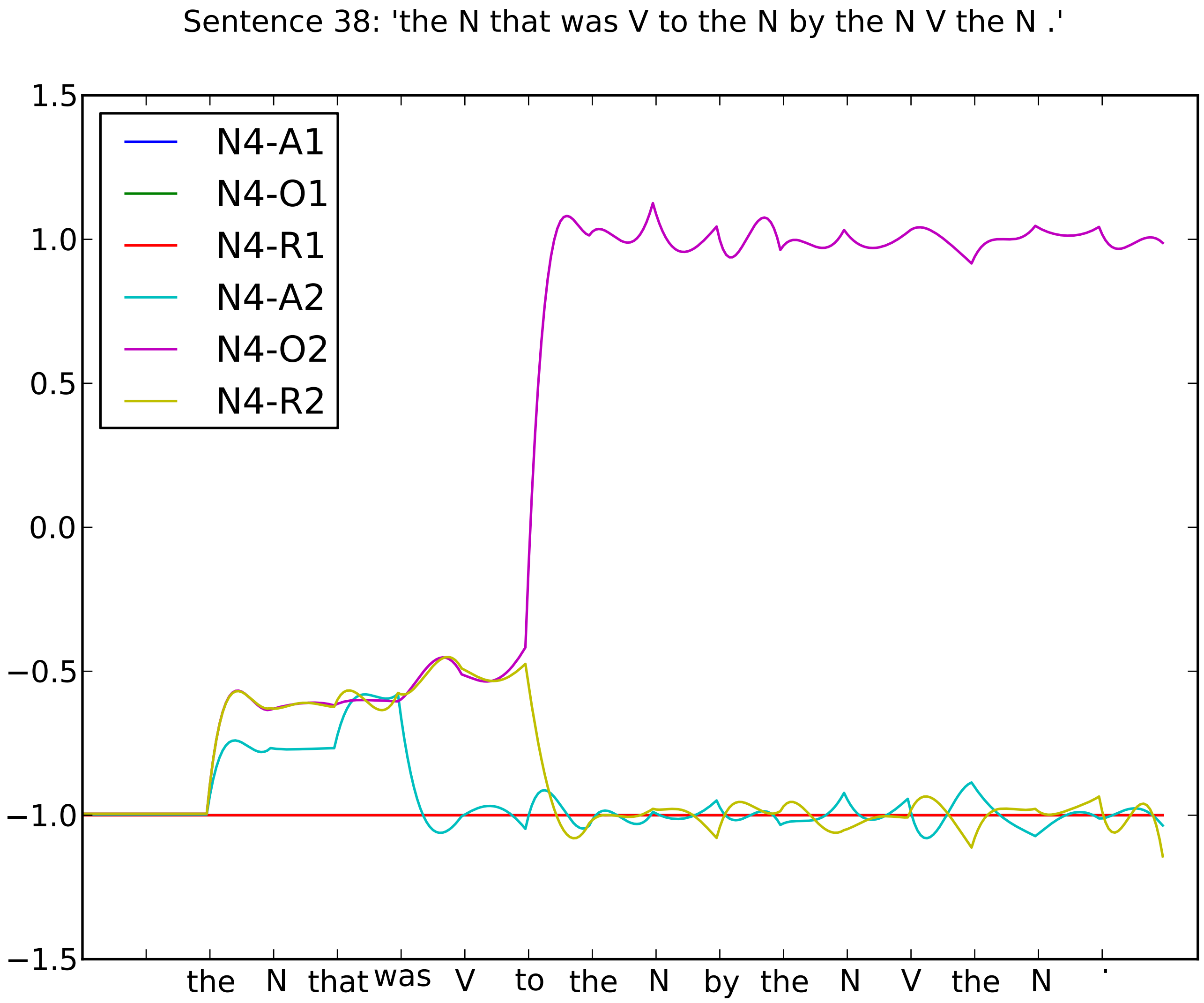

Sentence 38: 'the N that was V to the N by the N V the N .'
1.5
1.0
0.5
0.0
−0.5
−1.0
−1.5
N4-A1
N4-O1
N4-R1
N4-A2
N4-O2
N4-R2
the
N
that
was
V
to
the
N
by
the
N
V
the
N
.

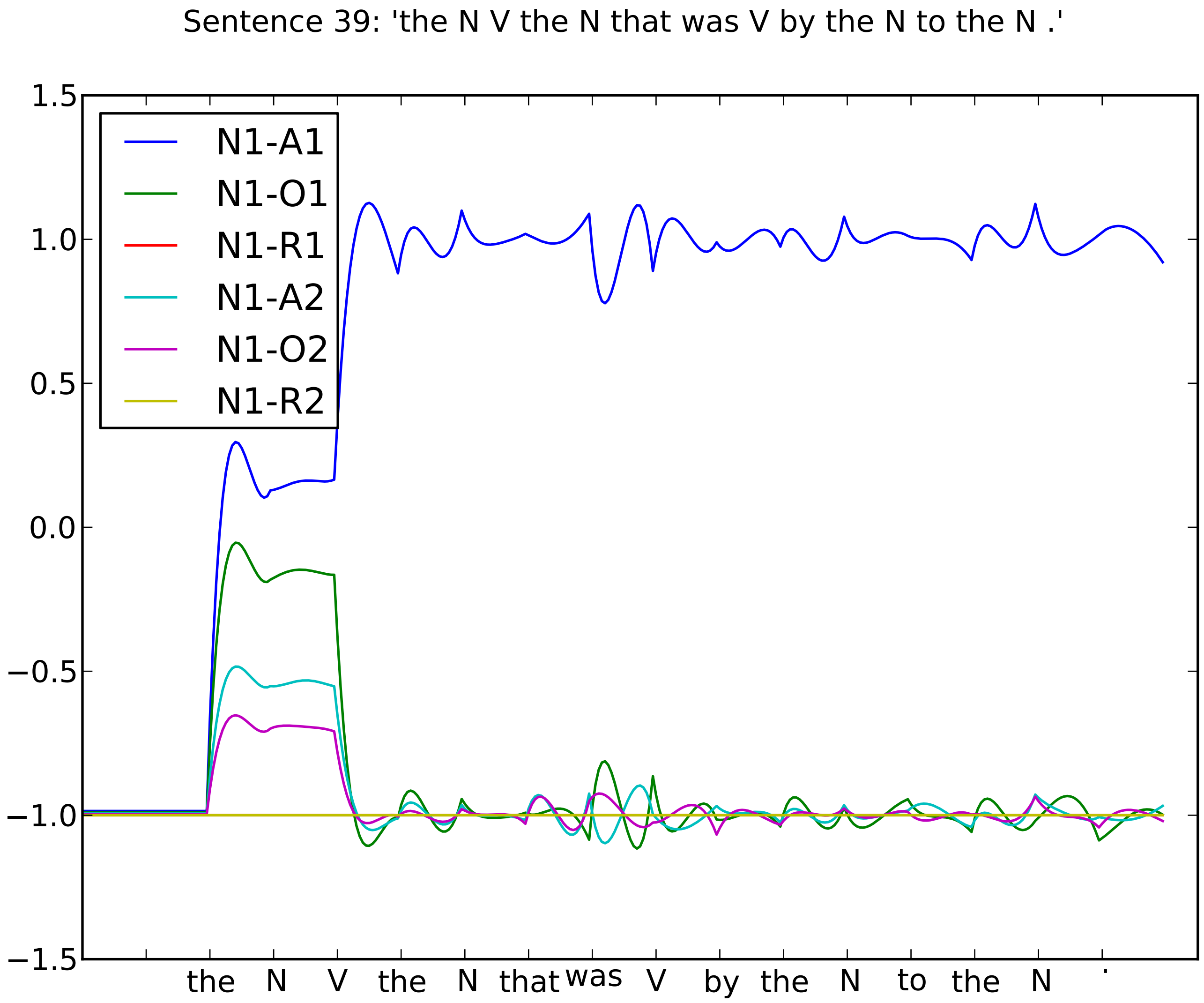
Sentence 39: 'the N V the N that was V by the N to the N .'
N1-A1
N1-O1
N1-R1
N1-A2
N1-O2
N1-R2
1.5
1.0
0.5
0.0
−0.5
−1.0
−1.5
the
N
V
the
N
that
was
V
by
the
N
to
the
N
.

Sentence 39: 'the N V the N that was V by the N to the N .'

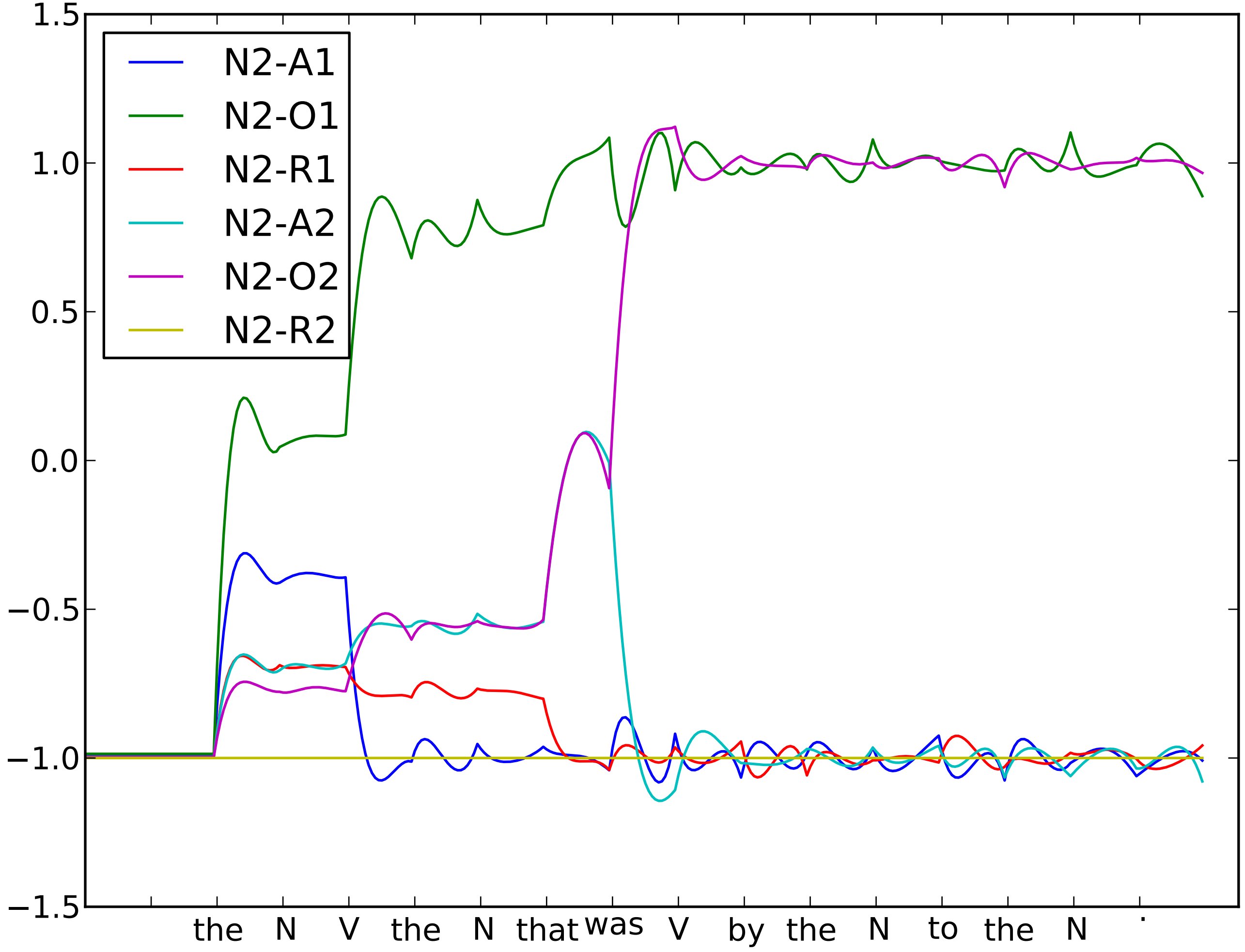

Sentence 39: 'the N V the N that was V by the N to the N .'

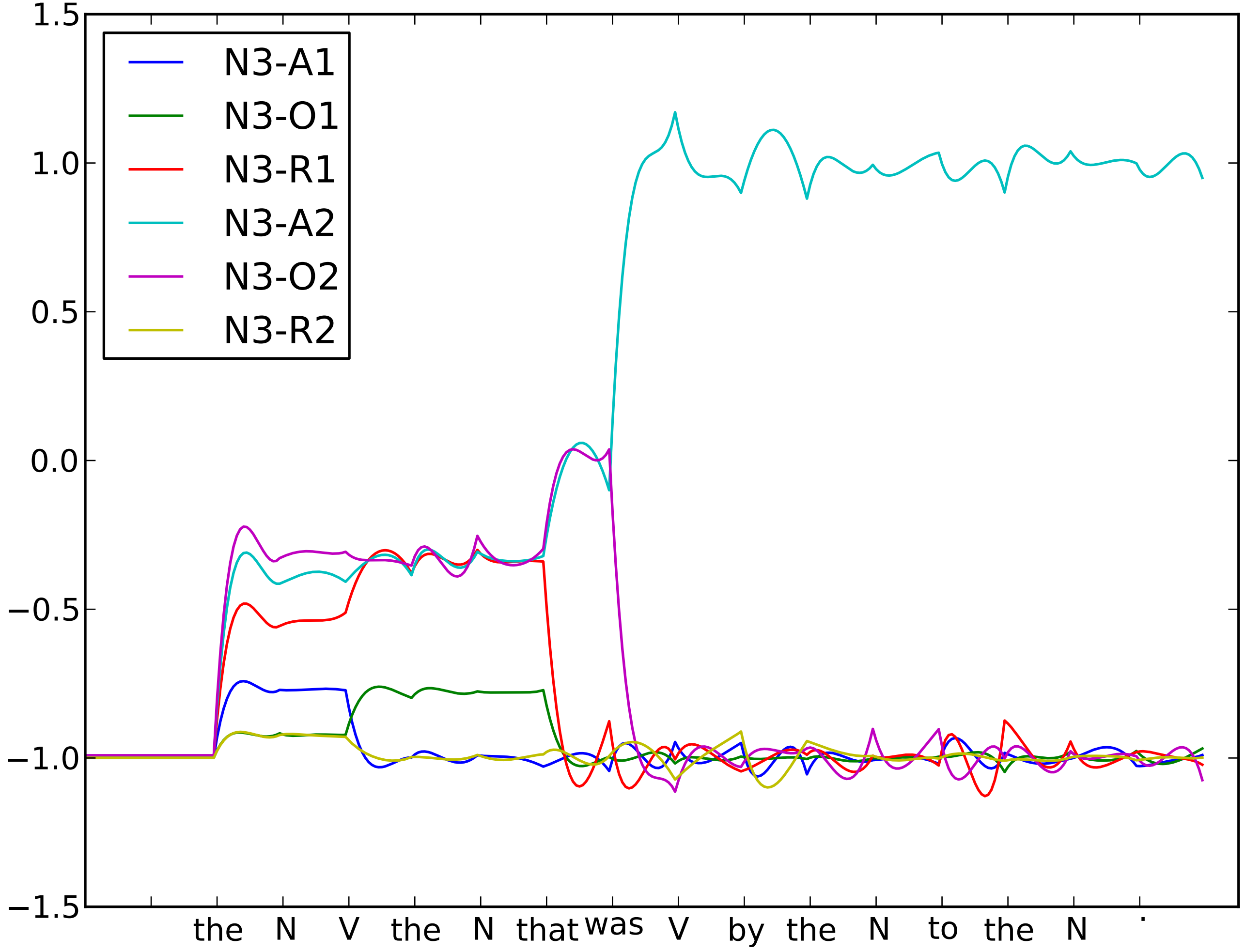

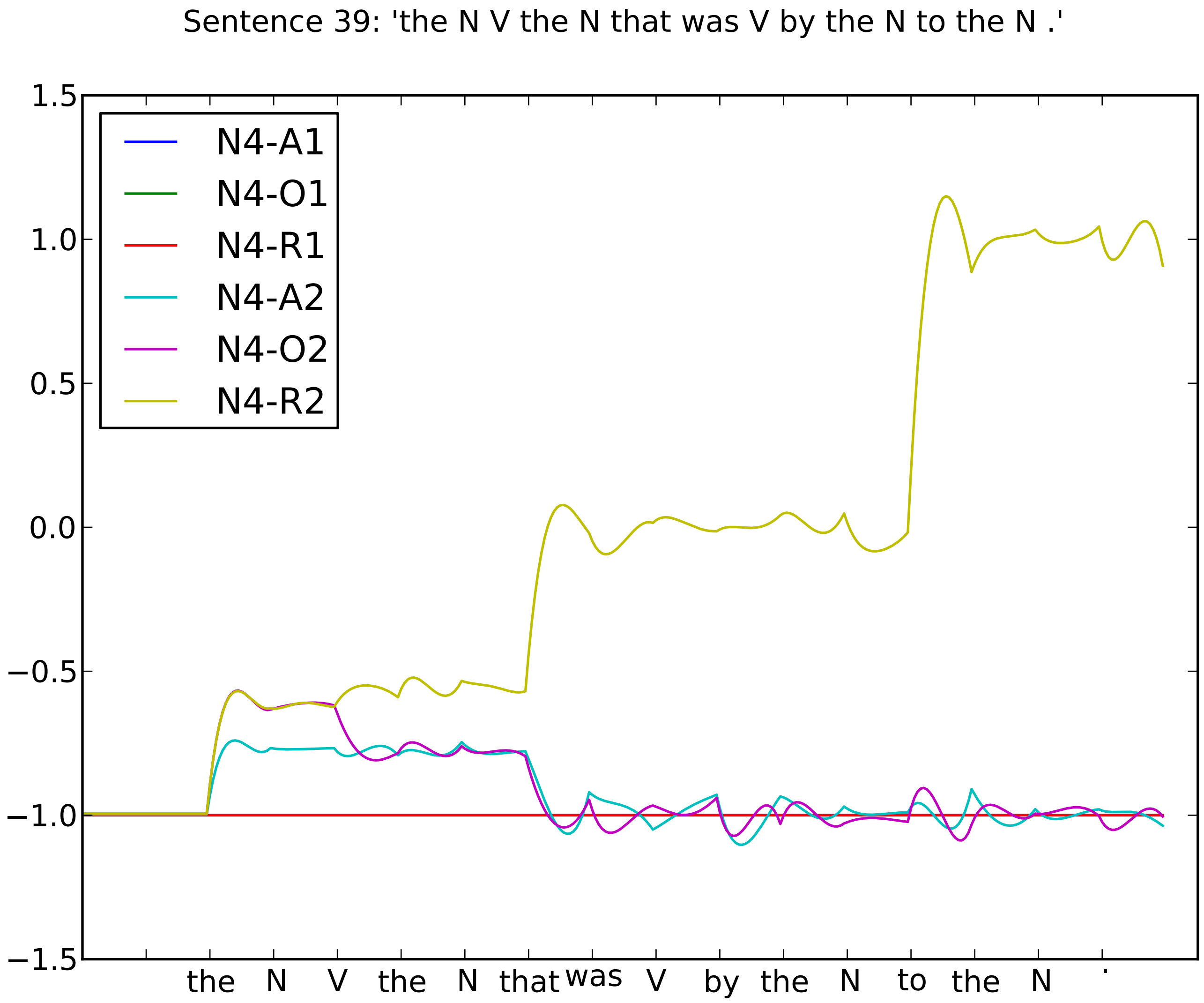

Sentence 39: 'the N V the N that was V by the N to the N .'
1.5
1.0
0.5
0.0
−0.5
−1.0
−1.5
N4-A1
N4-O1
N4-R1
N4-A2
N4-O2
N4-R2
the
N
V
the
N
that
was
V
by
the
N
to
the
N
.

Sentence 40: 'the N that V the N V the N to the N .'

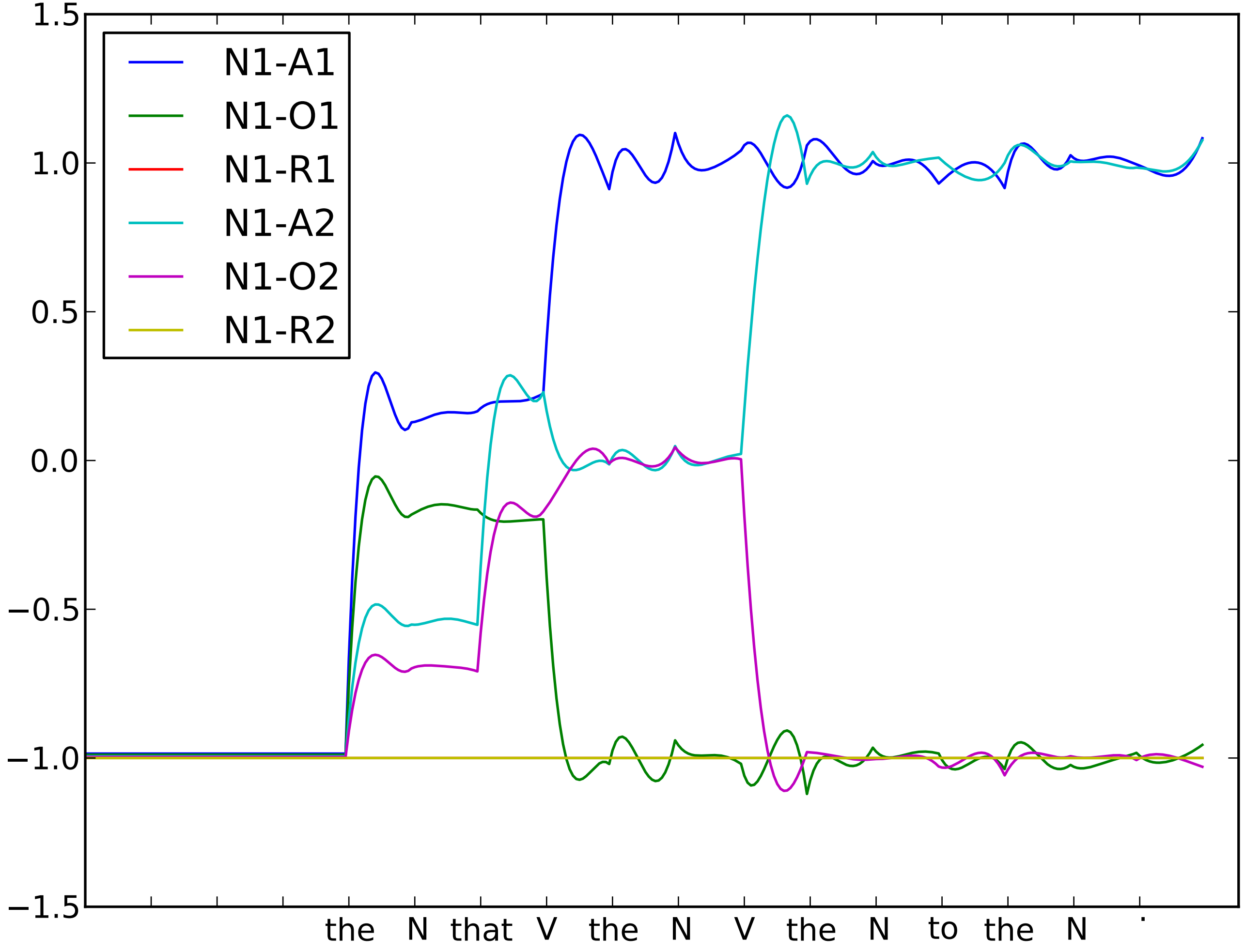

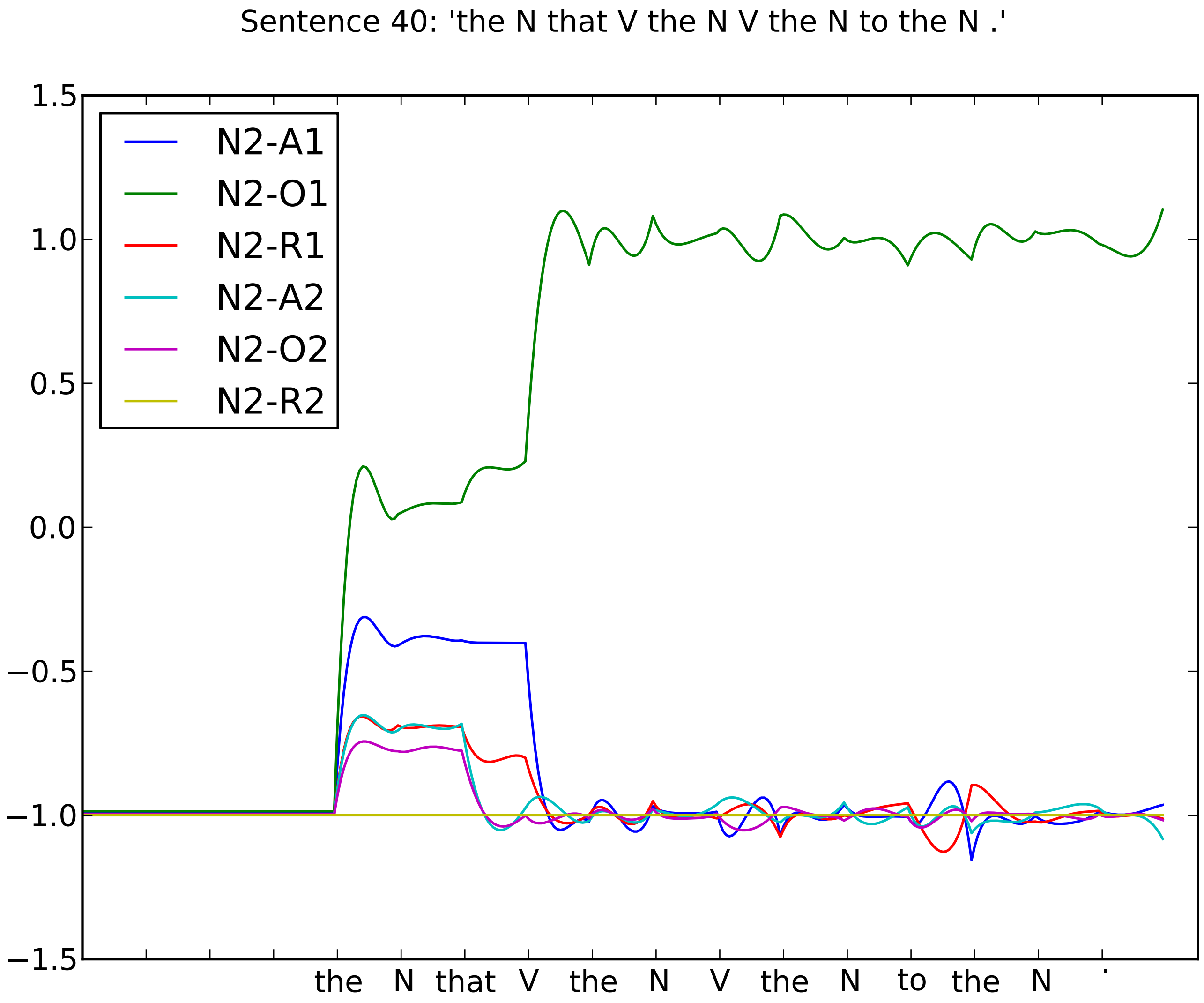
Sentence 40: 'the N that V the N V the N to the N .'
N2-A1
N2-O1
N2-R1
N2-A2
N2-O2
N2-R2
1.5
1.0
0.5
0.0
−0.5
−1.0
−1.5
the
N
that
V
the
N
V
the
N
to
the
N
.

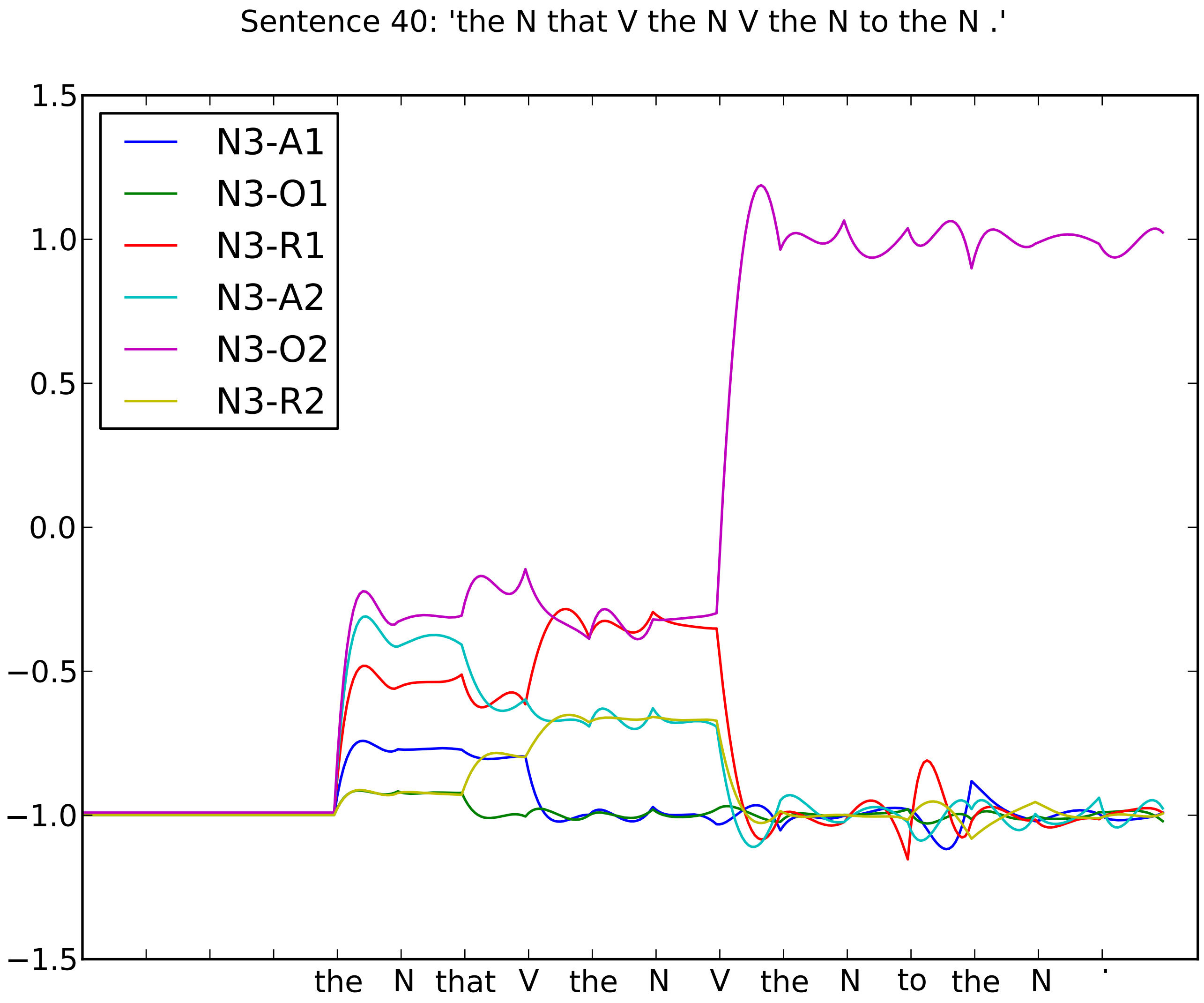

Sentence 40: 'the N that V the N V the N to the N .'
N3-A1
N3-O1
N3-R1
N3-A2
N3-O2
N3-R2
1.5
1.0
0.5
0.0
−0.5
−1.0
−1.5
the
N
that
V
the
N
V
the
N
to
the
N
.

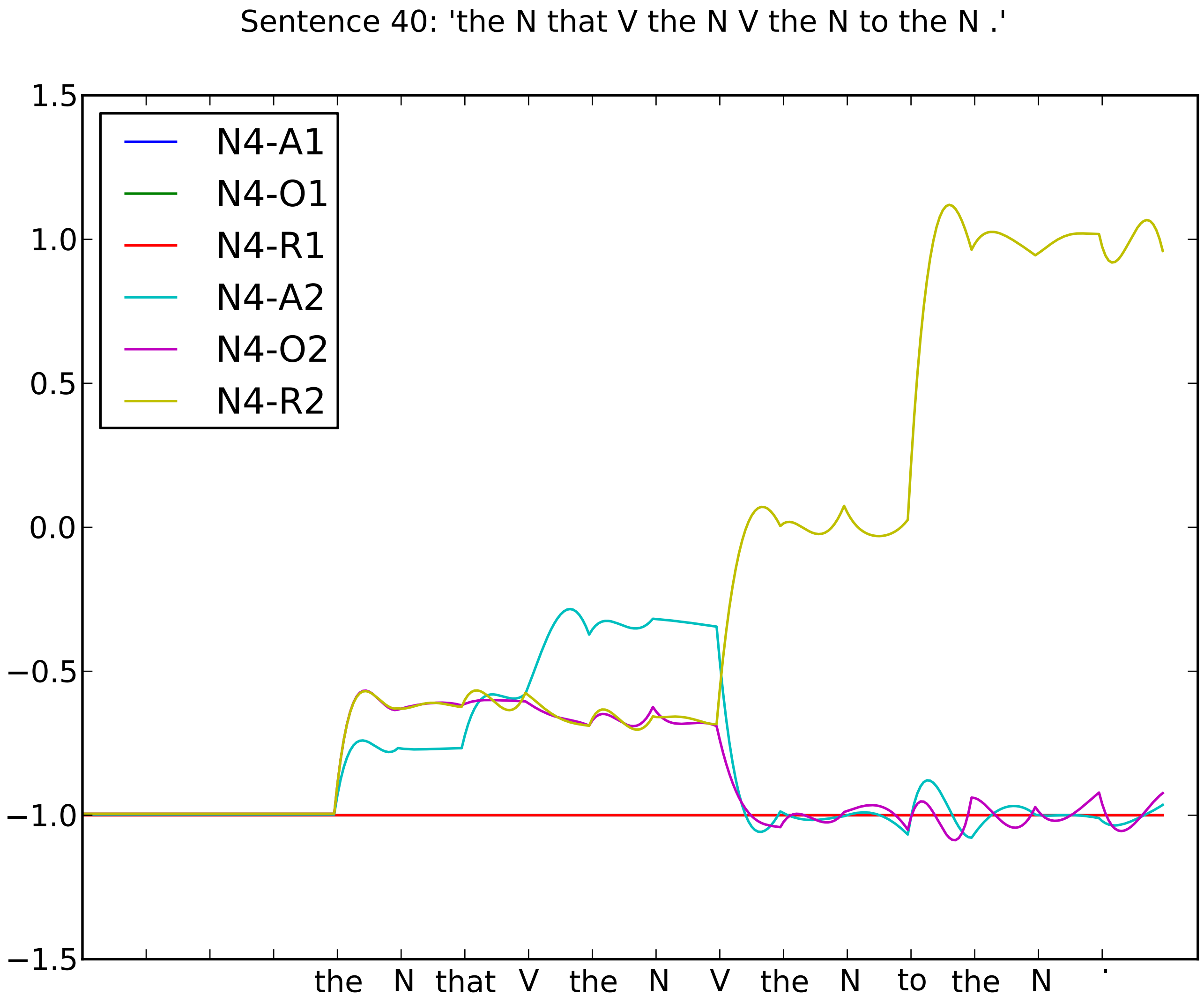
Sentence 40: 'the N that V the N V the N to the N .'
1.5
1.0
0.5
0.0
−0.5
−1.0
−1.5
N4-A1
N4-O1
N4-R1
N4-A2
N4-O2
N4-R2
the
N
that
V
the
N
V
the
N
to
the
N
.
