# Supplementary material for: Real-Time Parallel Processing of Grammatical Structure in the Fronto-Striatal System: A Recurrent Network Simulation Study Using Reservoir Computing
Source: PLoS One. 2013 Feb 1;8(2):e52946. doi: 10.1371/journal.pone.0052946 (PMC3562282; doi:10.1371/journal.pone.0052946)
Supplement: Text S4 — The 462 construction corpus. (RTF) [file pone.0052946.s007.rtf]

Supplementary Material 4 for Hinaut & Dominey

This describes the contents and structure of the 462 element corpus.

Each construction is described in four parts.  First, the “meaning” expressed with the open class words in the Predicate, Agent, Object, Recipient order.  Second, the ordered thematic roles as they will appear in the surface form.  Third, the surface form (sentence), and Fourth, the surface form with specific semantic words removed.:

Single verb construction:
n. walk giraffe <o> AP </o> ; the giraffe walk -s . # ['the', 'X', 'X', '-s', '.']

Two verb (relative) constructions: 
m. cut beaver fish , kiss guy fish <o> PAO , OPA </o> ; cut -ed by the beaver was the fish that was kiss -ed by the guy . # ['X', '-ed', 'by', 'the', 'X', 'was', 'the', 'X', 'that', 'was', 'X', '-ed', 'by', 'the', 'X', '.']

Construction number.  Main clause predicative meaning PAOR, optional relative clause predicative meaning <o> ordered thematic roles in the surface form </o>;  sentence . # sentence form with semantic words removed.

Corpus:
<closed class words>
-ed -ing -s by is it that the to was , .
</closed class words>
<train data>
1.	walk giraffe <o> AP </o> ; the giraffe walk -s . # ['the', 'X', 'X', '-s', '.']
2.	walk giraffe <o> PA </o> ; walk -ing was the giraffe . # ['X', '-ing', 'was', 'the', 'X', '.']
3.	cut beaver fish <o> APO </o> ; the beaver cut -s the fish . # ['the', 'X', 'X', '-s', 'the', 'X', '.']
4.	cut beaver fish <o> POA </o> ; cut -ed was the fish by the beaver . # ['X', '-ed', 'was', 'the', 'X', 'by', 'the', 'X', '.']
5.	cut beaver fish <o> OAP </o> ; the fish , the beaver cut -ed it . # ['the', 'X', ',', 'the', 'X', 'X', '-ed', 'it', '.']
6.	cut beaver fish <o> AOP </o> ; by the beaver the fish was cut -ed . # ['by', 'the', 'X', 'the', 'X', 'was', 'X', '-ed', '.']
7.	give dog cat mouse <o> APOR </o> ; the dog give -s the cat to the mouse . # ['the', 'X', 'X', '-s', 'the', 'X', 'to', 'the', 'X', '.']
8.	give dog cat mouse <o> APRO </o> ; the dog give -s to the mouse the cat . # ['the', 'X', 'X', '-s', 'to', 'the', 'X', 'the', 'X', '.']
9.	give dog cat mouse <o> ARPO </o> ; the dog to the mouse give -s the cat . # ['the', 'X', 'to', 'the', 'X', 'X', '-s', 'the', 'X', '.']
10.	walk giraffe , think giraffe <o> AP , AP </o> ; the giraffe that think -s walk -s . # ['the', 'X', 'that', 'X', '-s', 'X', '-s', '.']
11.	cut beaver fish <o> PAO </o> ; cut -ed by the beaver was the fish . # ['X', '-ed', 'by', 'the', 'X', 'was', 'the', 'X', '.']
12.	cut beaver fish <o> OPA </o> ; the fish was cut -ed by the beaver . # ['the', 'X', 'was', 'X', '-ed', 'by', 'the', 'X', '.']
13.	give dog cat mouse <o> PORA </o> ; give the cat to the mouse , the dog did . # ['X', 'the', 'X', 'to', 'the', 'X', ',', 'the', 'X', 'X', '.']
14.	give dog cat mouse <o> RAPO </o> ; to the mouse the dog give -s the cat . # ['to', 'the', 'X', 'the', 'X', 'X', '-s', 'the', 'X', '.']
15.	give dog cat mouse <o> PAOR </o> ; give -ed by the dog was the cat to the mouse . # ['X', '-ed', 'by', 'the', 'X', 'was', 'the', 'X', 'to', 'the', 'X', '.']
16.	give dog cat mouse <o> PARO </o> ; give -ed by the dog to the mouse was the cat . # ['X', '-ed', 'by', 'the', 'X', 'to', 'the', 'X', 'was', 'the', 'X', '.']
17.	give dog cat mouse <o> POAR </o> ; give -ed was the cat by the dog to the mouse . # ['X', '-ed', 'was', 'the', 'X', 'by', 'the', 'X', 'to', 'the', 'X', '.']
18.	give dog cat mouse <o> PRAO </o> ; give -ed to the mouse by the dog was the cat . # ['X', '-ed', 'to', 'the', 'X', 'by', 'the', 'X', 'was', 'the', 'X', '.']
19.	give dog cat mouse <o> PROA </o> ; give -ed to the mouse was the cat by the dog . # ['X', '-ed', 'to', 'the', 'X', 'was', 'the', 'X', 'by', 'the', 'X', '.']
20.	give dog cat mouse <o> OPAR </o> ; the cat was give -ed by the dog to the mouse . # ['the', 'X', 'was', 'X', '-ed', 'by', 'the', 'X', 'to', 'the', 'X', '.']
21.	give dog cat mouse <o> OPRA </o> ; the cat was give -ed to the mouse by the dog . # ['the', 'X', 'was', 'X', '-ed', 'to', 'the', 'X', 'by', 'the', 'X', '.']
22.	give dog cat mouse <o> OAPR </o> ; the cat by the dog was give -ed to the mouse . # ['the', 'X', 'by', 'the', 'X', 'was', 'X', '-ed', 'to', 'the', 'X', '.']
23.	give dog cat mouse <o> OARP </o> ; the cat by the dog to the mouse was give -ed . # ['the', 'X', 'by', 'the', 'X', 'to', 'the', 'X', 'was', 'X', '-ed', '.']
24.	give dog cat mouse <o> ORPA </o> ; the cat to the mouse was give -ed by the dog . # ['the', 'X', 'to', 'the', 'X', 'was', 'X', '-ed', 'by', 'the', 'X', '.']
25.	give dog cat mouse <o> ORAP </o> ; the cat to the mouse by the dog was give -ed . # ['the', 'X', 'to', 'the', 'X', 'by', 'the', 'X', 'was', 'X', '-ed', '.']
26.	walk giraffe , think giraffe <o> PA , AP </o> ; walk -ing was the giraffe that think -s . # ['X', '-ing', 'was', 'the', 'X', 'that', 'X', '-s', '.']
27.	give dog cat mouse <o> AOPR </o> ; by the dog the cat was give -ed to the mouse . # ['by', 'the', 'X', 'the', 'X', 'was', 'X', '-ed', 'to', 'the', 'X', '.']
28.	give dog cat mouse <o> AORP </o> ; by the dog the cat to the mouse was give -ed . # ['by', 'the', 'X', 'the', 'X', 'to', 'the', 'X', 'was', 'X', '-ed', '.']
29.	give dog cat mouse <o> AROP </o> ; by the dog to the mouse the cat was give -ed . # ['by', 'the', 'X', 'to', 'the', 'X', 'the', 'X', 'was', 'X', '-ed', '.']
30.	give dog cat mouse <o> RPAO </o> ; to the mouse was give -ed by the dog the cat . # ['to', 'the', 'X', 'was', 'X', '-ed', 'by', 'the', 'X', 'the', 'X', '.']
31.	give dog cat mouse <o> RPOA </o> ; to the mouse was give -ed the cat by the dog . # ['to', 'the', 'X', 'was', 'X', '-ed', 'the', 'X', 'by', 'the', 'X', '.']
32.	give dog cat mouse <o> RAOP </o> ; to the mouse by the dog the cat was give -ed . # ['to', 'the', 'X', 'by', 'the', 'X', 'the', 'X', 'was', 'X', '-ed', '.']
33.	give dog cat mouse <o> ROPA </o> ; to the mouse the cat was give -ed by the dog . # ['to', 'the', 'X', 'the', 'X', 'was', 'X', '-ed', 'by', 'the', 'X', '.']
34.	give dog cat mouse <o> ROAP </o> ; to the mouse the cat by the dog was give -ed . # ['to', 'the', 'X', 'the', 'X', 'by', 'the', 'X', 'was', 'X', '-ed', '.']
35.	cut beaver fish , think beaver <o> APO , AP </o> ; the beaver that think -s cut -s the fish . # ['the', 'X', 'that', 'X', '-s', 'X', '-s', 'the', 'X', '.']
36.	cut beaver fish , think fish <o> APO , AP </o> ; the beaver cut -s the fish that think -s . # ['the', 'X', 'X', '-s', 'the', 'X', 'that', 'X', '-s', '.']
37.	walk giraffe , kiss giraffe girl <o> AP , APO </o> ; the giraffe that kiss -s the girl walk -s . # ['the', 'X', 'that', 'X', '-s', 'the', 'X', 'X', '-s', '.']
38.	walk giraffe , kiss guy giraffe <o> AP , OAP </o> ; the giraffe that the guy kiss -s walk -s . # ['the', 'X', 'that', 'the', 'X', 'X', '-s', 'X', '-s', '.']
39.	cut beaver fish , think fish <o> POA , AP </o> ; cut -ed was the fish that think -s by the beaver . # ['X', '-ed', 'was', 'the', 'X', 'that', 'X', '-s', 'by', 'the', 'X', '.']
40.	cut beaver fish , think beaver <o> POA , AP </o> ; cut -ed was the fish by the beaver that think -s . # ['X', '-ed', 'was', 'the', 'X', 'by', 'the', 'X', 'that', 'X', '-s', '.']
41.	cut beaver fish , think fish <o> OAP , AP </o> ; the fish that think -s , the beaver cut -ed it . # ['the', 'X', 'that', 'X', '-s', ',', 'the', 'X', 'X', '-ed', 'it', '.']
42.	cut beaver fish , think beaver <o> OAP , AP </o> ; the fish , the beaver that think -s cut -ed it . # ['the', 'X', ',', 'the', 'X', 'that', 'X', '-s', 'X', '-ed', 'it', '.']
43.	walk giraffe , kiss guy giraffe <o> AP , OPA </o> ; the giraffe that was kiss -ed by the guy walk -s . # ['the', 'X', 'that', 'was', 'X', '-ed', 'by', 'the', 'X', 'X', '-s', '.']
44.	walk giraffe , kiss giraffe girl <o> PA , APO </o> ; walk -ing was the giraffe that kiss -s the girl . # ['X', '-ing', 'was', 'the', 'X', 'that', 'X', '-s', 'the', 'X', '.']
45.	walk giraffe , kiss guy giraffe <o> PA , OAP </o> ; walk -ing was the giraffe that the guy kiss -s . # ['X', '-ing', 'was', 'the', 'X', 'that', 'the', 'X', 'X', '-s', '.']
46.	cut beaver fish , think beaver <o> AOP , AP </o> ; by the beaver that think -s the fish was cut -ed . # ['by', 'the', 'X', 'that', 'X', '-s', 'the', 'X', 'was', 'X', '-ed', '.']
47.	cut beaver fish , think fish <o> AOP , AP </o> ; by the beaver the fish that think -s was cut -ed . # ['by', 'the', 'X', 'the', 'X', 'that', 'X', '-s', 'was', 'X', '-ed', '.']
48.	give dog cat mouse , think dog <o> APOR , AP </o> ; the dog that think -s give -s the cat to the mouse . # ['the', 'X', 'that', 'X', '-s', 'X', '-s', 'the', 'X', 'to', 'the', 'X', '.']
49.	give dog cat mouse , think cat <o> APOR , AP </o> ; the dog give -s the cat that think -s to the mouse . # ['the', 'X', 'X', '-s', 'the', 'X', 'that', 'X', '-s', 'to', 'the', 'X', '.']
50.	give dog cat mouse , think mouse <o> APOR , AP </o> ; the dog give -s the cat to the mouse that think -s . # ['the', 'X', 'X', '-s', 'the', 'X', 'to', 'the', 'X', 'that', 'X', '-s', '.']
51.	give dog cat mouse , think dog <o> APRO , AP </o> ; the dog that think -s give -s to the mouse the cat . # ['the', 'X', 'that', 'X', '-s', 'X', '-s', 'to', 'the', 'X', 'the', 'X', '.']
52.	give dog cat mouse , think mouse <o> APRO , AP </o> ; the dog give -s to the mouse that think -s the cat . # ['the', 'X', 'X', '-s', 'to', 'the', 'X', 'that', 'X', '-s', 'the', 'X', '.']
53.	give dog cat mouse , think cat <o> APRO , AP </o> ; the dog give -s to the mouse the cat that think -s . # ['the', 'X', 'X', '-s', 'to', 'the', 'X', 'the', 'X', 'that', 'X', '-s', '.']
54.	give dog cat mouse , think dog <o> ARPO , AP </o> ; the dog that think -s to the mouse give -s the cat . # ['the', 'X', 'that', 'X', '-s', 'to', 'the', 'X', 'X', '-s', 'the', 'X', '.']
55.	give dog cat mouse , think mouse <o> ARPO , AP </o> ; the dog to the mouse that think -s give -s the cat . # ['the', 'X', 'to', 'the', 'X', 'that', 'X', '-s', 'X', '-s', 'the', 'X', '.']
56.	give dog cat mouse , think cat <o> ARPO , AP </o> ; the dog to the mouse give -s the cat that think -s . # ['the', 'X', 'to', 'the', 'X', 'X', '-s', 'the', 'X', 'that', 'X', '-s', '.']
57.	walk giraffe , kiss giraffe girl <o> AP , AOP </o> ; the giraffe that the girl was kiss -ed by walk -s . # ['the', 'X', 'that', 'the', 'X', 'was', 'X', '-ed', 'by', 'X', '-s', '.']
58.	cut beaver fish , kiss beaver girl <o> APO , APO </o> ; the beaver that kiss -s the girl cut -s the fish . # ['the', 'X', 'that', 'X', '-s', 'the', 'X', 'X', '-s', 'the', 'X', '.']
59.	cut beaver fish , kiss guy beaver <o> APO , OAP </o> ; the beaver that the guy kiss -s cut -s the fish . # ['the', 'X', 'that', 'the', 'X', 'X', '-s', 'X', '-s', 'the', 'X', '.']
60.	cut beaver fish , kiss fish girl <o> APO , APO </o> ; the beaver cut -s the fish that kiss -s the girl . # ['the', 'X', 'X', '-s', 'the', 'X', 'that', 'X', '-s', 'the', 'X', '.']
61.	cut beaver fish , kiss guy fish <o> APO , OAP </o> ; the beaver cut -s the fish that the guy kiss -s . # ['the', 'X', 'X', '-s', 'the', 'X', 'that', 'the', 'X', 'X', '-s', '.']
62.	cut beaver fish , think beaver <o> PAO , AP </o> ; cut -ed by the beaver that think -s was the fish . # ['X', '-ed', 'by', 'the', 'X', 'that', 'X', '-s', 'was', 'the', 'X', '.']
63.	cut beaver fish , think fish <o> PAO , AP </o> ; cut -ed by the beaver was the fish that think -s . # ['X', '-ed', 'by', 'the', 'X', 'was', 'the', 'X', 'that', 'X', '-s', '.']
64.	cut beaver fish , think fish <o> OPA , AP </o> ; the fish that think -s was cut -ed by the beaver . # ['the', 'X', 'that', 'X', '-s', 'was', 'X', '-ed', 'by', 'the', 'X', '.']
65.	cut beaver fish , think beaver <o> OPA , AP </o> ; the fish was cut -ed by the beaver that think -s . # ['the', 'X', 'was', 'X', '-ed', 'by', 'the', 'X', 'that', 'X', '-s', '.']
66.	give dog cat mouse , think cat <o> PORA , AP </o> ; give the cat that think -s to the mouse , the dog did . # ['X', 'the', 'X', 'that', 'X', '-s', 'to', 'the', 'X', ',', 'the', 'X', 'X', '.']
67.	give dog cat mouse , think mouse <o> PORA , AP </o> ; give the cat to the mouse that think -s , the dog did . # ['X', 'the', 'X', 'to', 'the', 'X', 'that', 'X', '-s', ',', 'the', 'X', 'X', '.']
68.	give dog cat mouse , think dog <o> PORA , AP </o> ; give the cat to the mouse , the dog that think -s did . # ['X', 'the', 'X', 'to', 'the', 'X', ',', 'the', 'X', 'that', 'X', '-s', 'X', '.']
69.	give dog cat mouse , think mouse <o> RAPO , AP </o> ; to the mouse that think -s the dog give -s the cat . # ['to', 'the', 'X', 'that', 'X', '-s', 'the', 'X', 'X', '-s', 'the', 'X', '.']
70.	give dog cat mouse , think dog <o> RAPO , AP </o> ; to the mouse the dog that think -s give -s the cat . # ['to', 'the', 'X', 'the', 'X', 'that', 'X', '-s', 'X', '-s', 'the', 'X', '.']
71.	give dog cat mouse , think cat <o> RAPO , AP </o> ; to the mouse the dog give -s the cat that think -s . # ['to', 'the', 'X', 'the', 'X', 'X', '-s', 'the', 'X', 'that', 'X', '-s', '.']
72.	walk giraffe , kiss guy giraffe <o> PA , OPA </o> ; walk -ing was the giraffe that was kiss -ed by the guy . # ['X', '-ing', 'was', 'the', 'X', 'that', 'was', 'X', '-ed', 'by', 'the', 'X', '.']
73.	give dog cat mouse , think dog <o> PAOR , AP </o> ; give -ed by the dog that think -s was the cat to the mouse . # ['X', '-ed', 'by', 'the', 'X', 'that', 'X', '-s', 'was', 'the', 'X', 'to', 'the', 'X', '.']
74.	give dog cat mouse , think cat <o> PAOR , AP </o> ; give -ed by the dog was the cat that think -s to the mouse . # ['X', '-ed', 'by', 'the', 'X', 'was', 'the', 'X', 'that', 'X', '-s', 'to', 'the', 'X', '.']
75.	give dog cat mouse , think mouse <o> PAOR , AP </o> ; give -ed by the dog was the cat to the mouse that think -s . # ['X', '-ed', 'by', 'the', 'X', 'was', 'the', 'X', 'to', 'the', 'X', 'that', 'X', '-s', '.']
76.	give dog cat mouse , think dog <o> PARO , AP </o> ; give -ed by the dog that think -s to the mouse was the cat . # ['X', '-ed', 'by', 'the', 'X', 'that', 'X', '-s', 'to', 'the', 'X', 'was', 'the', 'X', '.']
77.	give dog cat mouse , think mouse <o> PARO , AP </o> ; give -ed by the dog to the mouse that think -s was the cat . # ['X', '-ed', 'by', 'the', 'X', 'to', 'the', 'X', 'that', 'X', '-s', 'was', 'the', 'X', '.']
78.	give dog cat mouse , think cat <o> PARO , AP </o> ; give -ed by the dog to the mouse was the cat that think -s . # ['X', '-ed', 'by', 'the', 'X', 'to', 'the', 'X', 'was', 'the', 'X', 'that', 'X', '-s', '.']
79.	give dog cat mouse , think cat <o> POAR , AP </o> ; give -ed was the cat that think -s by the dog to the mouse . # ['X', '-ed', 'was', 'the', 'X', 'that', 'X', '-s', 'by', 'the', 'X', 'to', 'the', 'X', '.']
80.	give dog cat mouse , think dog <o> POAR , AP </o> ; give -ed was the cat by the dog that think -s to the mouse . # ['X', '-ed', 'was', 'the', 'X', 'by', 'the', 'X', 'that', 'X', '-s', 'to', 'the', 'X', '.']
81.	give dog cat mouse , think mouse <o> POAR , AP </o> ; give -ed was the cat by the dog to the mouse that think -s . # ['X', '-ed', 'was', 'the', 'X', 'by', 'the', 'X', 'to', 'the', 'X', 'that', 'X', '-s', '.']
82.	give dog cat mouse , think mouse <o> PRAO , AP </o> ; give -ed to the mouse that think -s by the dog was the cat . # ['X', '-ed', 'to', 'the', 'X', 'that', 'X', '-s', 'by', 'the', 'X', 'was', 'the', 'X', '.']
83.	give dog cat mouse , think dog <o> PRAO , AP </o> ; give -ed to the mouse by the dog that think -s was the cat . # ['X', '-ed', 'to', 'the', 'X', 'by', 'the', 'X', 'that', 'X', '-s', 'was', 'the', 'X', '.']
84.	give dog cat mouse , think cat <o> PRAO , AP </o> ; give -ed to the mouse by the dog was the cat that think -s . # ['X', '-ed', 'to', 'the', 'X', 'by', 'the', 'X', 'was', 'the', 'X', 'that', 'X', '-s', '.']
85.	give dog cat mouse , think mouse <o> PROA , AP </o> ; give -ed to the mouse that think -s was the cat by the dog . # ['X', '-ed', 'to', 'the', 'X', 'that', 'X', '-s', 'was', 'the', 'X', 'by', 'the', 'X', '.']
86.	give dog cat mouse , think cat <o> PROA , AP </o> ; give -ed to the mouse was the cat that think -s by the dog . # ['X', '-ed', 'to', 'the', 'X', 'was', 'the', 'X', 'that', 'X', '-s', 'by', 'the', 'X', '.']
87.	give dog cat mouse , think dog <o> PROA , AP </o> ; give -ed to the mouse was the cat by the dog that think -s . # ['X', '-ed', 'to', 'the', 'X', 'was', 'the', 'X', 'by', 'the', 'X', 'that', 'X', '-s', '.']
88.	give dog cat mouse , think cat <o> OPAR , AP </o> ; the cat that think -s was give -ed by the dog to the mouse . # ['the', 'X', 'that', 'X', '-s', 'was', 'X', '-ed', 'by', 'the', 'X', 'to', 'the', 'X', '.']
89.	give dog cat mouse , think dog <o> OPAR , AP </o> ; the cat was give -ed by the dog that think -s to the mouse . # ['the', 'X', 'was', 'X', '-ed', 'by', 'the', 'X', 'that', 'X', '-s', 'to', 'the', 'X', '.']
90.	give dog cat mouse , think mouse <o> OPAR , AP </o> ; the cat was give -ed by the dog to the mouse that think -s . # ['the', 'X', 'was', 'X', '-ed', 'by', 'the', 'X', 'to', 'the', 'X', 'that', 'X', '-s', '.']
91.	give dog cat mouse , think cat <o> OPRA , AP </o> ; the cat that think -s was give -ed to the mouse by the dog . # ['the', 'X', 'that', 'X', '-s', 'was', 'X', '-ed', 'to', 'the', 'X', 'by', 'the', 'X', '.']
92.	give dog cat mouse , think mouse <o> OPRA , AP </o> ; the cat was give -ed to the mouse that think -s by the dog . # ['the', 'X', 'was', 'X', '-ed', 'to', 'the', 'X', 'that', 'X', '-s', 'by', 'the', 'X', '.']
93.	give dog cat mouse , think dog <o> OPRA , AP </o> ; the cat was give -ed to the mouse by the dog that think -s . # ['the', 'X', 'was', 'X', '-ed', 'to', 'the', 'X', 'by', 'the', 'X', 'that', 'X', '-s', '.']
94.	give dog cat mouse , think cat <o> OAPR , AP </o> ; the cat that think -s by the dog was give -ed to the mouse . # ['the', 'X', 'that', 'X', '-s', 'by', 'the', 'X', 'was', 'X', '-ed', 'to', 'the', 'X', '.']
95.	give dog cat mouse , think dog <o> OAPR , AP </o> ; the cat by the dog that think -s was give -ed to the mouse . # ['the', 'X', 'by', 'the', 'X', 'that', 'X', '-s', 'was', 'X', '-ed', 'to', 'the', 'X', '.']
96.	give dog cat mouse , think mouse <o> OAPR , AP </o> ; the cat by the dog was give -ed to the mouse that think -s . # ['the', 'X', 'by', 'the', 'X', 'was', 'X', '-ed', 'to', 'the', 'X', 'that', 'X', '-s', '.']
97.	give dog cat mouse , think cat <o> OARP , AP </o> ; the cat that think -s by the dog to the mouse was give -ed . # ['the', 'X', 'that', 'X', '-s', 'by', 'the', 'X', 'to', 'the', 'X', 'was', 'X', '-ed', '.']
98.	give dog cat mouse , think dog <o> OARP , AP </o> ; the cat by the dog that think -s to the mouse was give -ed . # ['the', 'X', 'by', 'the', 'X', 'that', 'X', '-s', 'to', 'the', 'X', 'was', 'X', '-ed', '.']
99.	give dog cat mouse , think mouse <o> OARP , AP </o> ; the cat by the dog to the mouse that think -s was give -ed . # ['the', 'X', 'by', 'the', 'X', 'to', 'the', 'X', 'that', 'X', '-s', 'was', 'X', '-ed', '.']
100.	give dog cat mouse , think cat <o> ORPA , AP </o> ; the cat that think -s to the mouse was give -ed by the dog . # ['the', 'X', 'that', 'X', '-s', 'to', 'the', 'X', 'was', 'X', '-ed', 'by', 'the', 'X', '.']
101.	give dog cat mouse , think mouse <o> ORPA , AP </o> ; the cat to the mouse that think -s was give -ed by the dog . # ['the', 'X', 'to', 'the', 'X', 'that', 'X', '-s', 'was', 'X', '-ed', 'by', 'the', 'X', '.']
102.	give dog cat mouse , think dog <o> ORPA , AP </o> ; the cat to the mouse was give -ed by the dog that think -s . # ['the', 'X', 'to', 'the', 'X', 'was', 'X', '-ed', 'by', 'the', 'X', 'that', 'X', '-s', '.']
103.	give dog cat mouse , think cat <o> ORAP , AP </o> ; the cat that think -s to the mouse by the dog was give -ed . # ['the', 'X', 'that', 'X', '-s', 'to', 'the', 'X', 'by', 'the', 'X', 'was', 'X', '-ed', '.']
104.	give dog cat mouse , think mouse <o> ORAP , AP </o> ; the cat to the mouse that think -s by the dog was give -ed . # ['the', 'X', 'to', 'the', 'X', 'that', 'X', '-s', 'by', 'the', 'X', 'was', 'X', '-ed', '.']
105.	give dog cat mouse , think dog <o> ORAP , AP </o> ; the cat to the mouse by the dog that think -s was give -ed . # ['the', 'X', 'to', 'the', 'X', 'by', 'the', 'X', 'that', 'X', '-s', 'was', 'X', '-ed', '.']
106.	walk giraffe , kiss giraffe girl <o> PA , AOP </o> ; walk -ing was the giraffe that the girl was kiss -ed by . # ['X', '-ing', 'was', 'the', 'X', 'that', 'the', 'X', 'was', 'X', '-ed', 'by', '.']
107.	cut beaver fish , kiss guy beaver <o> APO , OPA </o> ; the beaver that was kiss -ed by the guy cut -s the fish . # ['the', 'X', 'that', 'was', 'X', '-ed', 'by', 'the', 'X', 'X', '-s', 'the', 'X', '.']
108.	cut beaver fish , kiss guy fish <o> APO , OPA </o> ; the beaver cut -s the fish that was kiss -ed by the guy . # ['the', 'X', 'X', '-s', 'the', 'X', 'that', 'was', 'X', '-ed', 'by', 'the', 'X', '.']
109.	cut beaver fish , kiss fish girl <o> POA , APO </o> ; cut -ed was the fish that kiss -s the girl by the beaver . # ['X', '-ed', 'was', 'the', 'X', 'that', 'X', '-s', 'the', 'X', 'by', 'the', 'X', '.']
110.	cut beaver fish , kiss guy fish <o> POA , OAP </o> ; cut -ed was the fish that the guy kiss -s by the beaver . # ['X', '-ed', 'was', 'the', 'X', 'that', 'the', 'X', 'X', '-s', 'by', 'the', 'X', '.']
111.	cut beaver fish , kiss beaver girl <o> POA , APO </o> ; cut -ed was the fish by the beaver that kiss -s the girl . # ['X', '-ed', 'was', 'the', 'X', 'by', 'the', 'X', 'that', 'X', '-s', 'the', 'X', '.']
112.	cut beaver fish , kiss guy beaver <o> POA , OAP </o> ; cut -ed was the fish by the beaver that the guy kiss -s . # ['X', '-ed', 'was', 'the', 'X', 'by', 'the', 'X', 'that', 'the', 'X', 'X', '-s', '.']
113.	cut beaver fish , kiss fish girl <o> OAP , APO </o> ; the fish that kiss -s the girl , the beaver cut -ed it . # ['the', 'X', 'that', 'X', '-s', 'the', 'X', ',', 'the', 'X', 'X', '-ed', 'it', '.']
114.	cut beaver fish , kiss guy fish <o> OAP , OAP </o> ; the fish that the guy kiss -s , the beaver cut -ed it . # ['the', 'X', 'that', 'the', 'X', 'X', '-s', ',', 'the', 'X', 'X', '-ed', 'it', '.']
115.	cut beaver fish , kiss beaver girl <o> OAP , APO </o> ; the fish , the beaver that kiss -s the girl cut -ed it . # ['the', 'X', ',', 'the', 'X', 'that', 'X', '-s', 'the', 'X', 'X', '-ed', 'it', '.']
116.	cut beaver fish , kiss guy beaver <o> OAP , OAP </o> ; the fish , the beaver that the guy kiss -s cut -ed it . # ['the', 'X', ',', 'the', 'X', 'that', 'the', 'X', 'X', '-s', 'X', '-ed', 'it', '.']
117.	give dog cat mouse , think dog <o> AOPR , AP </o> ; by the dog that think -s the cat was give -ed to the mouse . # ['by', 'the', 'X', 'that', 'X', '-s', 'the', 'X', 'was', 'X', '-ed', 'to', 'the', 'X', '.']
118.	give dog cat mouse , think cat <o> AOPR , AP </o> ; by the dog the cat that think -s was give -ed to the mouse . # ['by', 'the', 'X', 'the', 'X', 'that', 'X', '-s', 'was', 'X', '-ed', 'to', 'the', 'X', '.']
119.	give dog cat mouse , think mouse <o> AOPR , AP </o> ; by the dog the cat was give -ed to the mouse that think -s . # ['by', 'the', 'X', 'the', 'X', 'was', 'X', '-ed', 'to', 'the', 'X', 'that', 'X', '-s', '.']
120.	give dog cat mouse , think dog <o> AORP , AP </o> ; by the dog that think -s the cat to the mouse was give -ed . # ['by', 'the', 'X', 'that', 'X', '-s', 'the', 'X', 'to', 'the', 'X', 'was', 'X', '-ed', '.']
121.	give dog cat mouse , think cat <o> AORP , AP </o> ; by the dog the cat that think -s to the mouse was give -ed . # ['by', 'the', 'X', 'the', 'X', 'that', 'X', '-s', 'to', 'the', 'X', 'was', 'X', '-ed', '.']
122.	give dog cat mouse , think mouse <o> AORP , AP </o> ; by the dog the cat to the mouse that think -s was give -ed . # ['by', 'the', 'X', 'the', 'X', 'to', 'the', 'X', 'that', 'X', '-s', 'was', 'X', '-ed', '.']
123.	give dog cat mouse , think dog <o> AROP , AP </o> ; by the dog that think -s to the mouse the cat was give -ed . # ['by', 'the', 'X', 'that', 'X', '-s', 'to', 'the', 'X', 'the', 'X', 'was', 'X', '-ed', '.']
124.	give dog cat mouse , think mouse <o> AROP , AP </o> ; by the dog to the mouse that think -s the cat was give -ed . # ['by', 'the', 'X', 'to', 'the', 'X', 'that', 'X', '-s', 'the', 'X', 'was', 'X', '-ed', '.']
125.	give dog cat mouse , think cat <o> AROP , AP </o> ; by the dog to the mouse the cat that think -s was give -ed . # ['by', 'the', 'X', 'to', 'the', 'X', 'the', 'X', 'that', 'X', '-s', 'was', 'X', '-ed', '.']
126.	give dog cat mouse , think mouse <o> RPAO , AP </o> ; to the mouse that think -s was give -ed by the dog the cat . # ['to', 'the', 'X', 'that', 'X', '-s', 'was', 'X', '-ed', 'by', 'the', 'X', 'the', 'X', '.']
127.	give dog cat mouse , think dog <o> RPAO , AP </o> ; to the mouse was give -ed by the dog that think -s the cat . # ['to', 'the', 'X', 'was', 'X', '-ed', 'by', 'the', 'X', 'that', 'X', '-s', 'the', 'X', '.']
128.	give dog cat mouse , think cat <o> RPAO , AP </o> ; to the mouse was give -ed by the dog the cat that think -s . # ['to', 'the', 'X', 'was', 'X', '-ed', 'by', 'the', 'X', 'the', 'X', 'that', 'X', '-s', '.']
129.	give dog cat mouse , think mouse <o> RPOA , AP </o> ; to the mouse that think -s was give -ed the cat by the dog . # ['to', 'the', 'X', 'that', 'X', '-s', 'was', 'X', '-ed', 'the', 'X', 'by', 'the', 'X', '.']
130.	give dog cat mouse , think cat <o> RPOA , AP </o> ; to the mouse was give -ed the cat that think -s by the dog . # ['to', 'the', 'X', 'was', 'X', '-ed', 'the', 'X', 'that', 'X', '-s', 'by', 'the', 'X', '.']
131.	give dog cat mouse , think dog <o> RPOA , AP </o> ; to the mouse was give -ed the cat by the dog that think -s . # ['to', 'the', 'X', 'was', 'X', '-ed', 'the', 'X', 'by', 'the', 'X', 'that', 'X', '-s', '.']
132.	give dog cat mouse , think mouse <o> RAOP , AP </o> ; to the mouse that think -s by the dog the cat was give -ed . # ['to', 'the', 'X', 'that', 'X', '-s', 'by', 'the', 'X', 'the', 'X', 'was', 'X', '-ed', '.']
133.	give dog cat mouse , think dog <o> RAOP , AP </o> ; to the mouse by the dog that think -s the cat was give -ed . # ['to', 'the', 'X', 'by', 'the', 'X', 'that', 'X', '-s', 'the', 'X', 'was', 'X', '-ed', '.']
134.	give dog cat mouse , think cat <o> RAOP , AP </o> ; to the mouse by the dog the cat that think -s was give -ed . # ['to', 'the', 'X', 'by', 'the', 'X', 'the', 'X', 'that', 'X', '-s', 'was', 'X', '-ed', '.']
135.	give dog cat mouse , think mouse <o> ROPA , AP </o> ; to the mouse that think -s the cat was give -ed by the dog . # ['to', 'the', 'X', 'that', 'X', '-s', 'the', 'X', 'was', 'X', '-ed', 'by', 'the', 'X', '.']
136.	give dog cat mouse , think cat <o> ROPA , AP </o> ; to the mouse the cat that think -s was give -ed by the dog . # ['to', 'the', 'X', 'the', 'X', 'that', 'X', '-s', 'was', 'X', '-ed', 'by', 'the', 'X', '.']
137.	give dog cat mouse , think dog <o> ROPA , AP </o> ; to the mouse the cat was give -ed by the dog that think -s . # ['to', 'the', 'X', 'the', 'X', 'was', 'X', '-ed', 'by', 'the', 'X', 'that', 'X', '-s', '.']
138.	give dog cat mouse , think mouse <o> ROAP , AP </o> ; to the mouse that think -s the cat by the dog was give -ed . # ['to', 'the', 'X', 'that', 'X', '-s', 'the', 'X', 'by', 'the', 'X', 'was', 'X', '-ed', '.']
139.	give dog cat mouse , think cat <o> ROAP , AP </o> ; to the mouse the cat that think -s by the dog was give -ed . # ['to', 'the', 'X', 'the', 'X', 'that', 'X', '-s', 'by', 'the', 'X', 'was', 'X', '-ed', '.']
140.	give dog cat mouse , think dog <o> ROAP , AP </o> ; to the mouse the cat by the dog that think -s was give -ed . # ['to', 'the', 'X', 'the', 'X', 'by', 'the', 'X', 'that', 'X', '-s', 'was', 'X', '-ed', '.']
141.	cut beaver fish , kiss beaver girl <o> APO , AOP </o> ; the beaver that the girl was kiss -ed by cut -s the fish . # ['the', 'X', 'that', 'the', 'X', 'was', 'X', '-ed', 'by', 'X', '-s', 'the', 'X', '.']
142.	cut beaver fish , kiss fish girl <o> APO , AOP </o> ; the beaver cut -s the fish that the girl was kiss -ed by . # ['the', 'X', 'X', '-s', 'the', 'X', 'that', 'the', 'X', 'was', 'X', '-ed', 'by', '.']
143.	cut beaver fish , kiss beaver girl <o> AOP , APO </o> ; by the beaver that kiss -s the girl the fish was cut -ed . # ['by', 'the', 'X', 'that', 'X', '-s', 'the', 'X', 'the', 'X', 'was', 'X', '-ed', '.']
144.	cut beaver fish , kiss guy beaver <o> AOP , OAP </o> ; by the beaver that the guy kiss -s the fish was cut -ed . # ['by', 'the', 'X', 'that', 'the', 'X', 'X', '-s', 'the', 'X', 'was', 'X', '-ed', '.']
145.	cut beaver fish , kiss fish girl <o> AOP , APO </o> ; by the beaver the fish that kiss -s the girl was cut -ed . # ['by', 'the', 'X', 'the', 'X', 'that', 'X', '-s', 'the', 'X', 'was', 'X', '-ed', '.']
146.	cut beaver fish , kiss guy fish <o> AOP , OAP </o> ; by the beaver the fish that the guy kiss -s was cut -ed . # ['by', 'the', 'X', 'the', 'X', 'that', 'the', 'X', 'X', '-s', 'was', 'X', '-ed', '.']
147.	give dog cat mouse , kiss dog girl <o> APOR , APO </o> ; the dog that kiss -s the girl give -s the cat to the mouse . # ['the', 'X', 'that', 'X', '-s', 'the', 'X', 'X', '-s', 'the', 'X', 'to', 'the', 'X', '.']
148.	give dog cat mouse , kiss guy dog <o> APOR , OAP </o> ; the dog that the guy kiss -s give -s the cat to the mouse . # ['the', 'X', 'that', 'the', 'X', 'X', '-s', 'X', '-s', 'the', 'X', 'to', 'the', 'X', '.']
149.	give dog cat mouse , kiss cat girl <o> APOR , APO </o> ; the dog give -s the cat that kiss -s the girl to the mouse . # ['the', 'X', 'X', '-s', 'the', 'X', 'that', 'X', '-s', 'the', 'X', 'to', 'the', 'X', '.']
150.	give dog cat mouse , kiss guy cat <o> APOR , OAP </o> ; the dog give -s the cat that the guy kiss -s to the mouse . # ['the', 'X', 'X', '-s', 'the', 'X', 'that', 'the', 'X', 'X', '-s', 'to', 'the', 'X', '.']
151.	give dog cat mouse , kiss mouse girl <o> APOR , APO </o> ; the dog give -s the cat to the mouse that kiss -s the girl . # ['the', 'X', 'X', '-s', 'the', 'X', 'to', 'the', 'X', 'that', 'X', '-s', 'the', 'X', '.']
152.	give dog cat mouse , kiss guy mouse <o> APOR , OAP </o> ; the dog give -s the cat to the mouse that the guy kiss -s . # ['the', 'X', 'X', '-s', 'the', 'X', 'to', 'the', 'X', 'that', 'the', 'X', 'X', '-s', '.']
153.	give dog cat mouse , kiss dog girl <o> APRO , APO </o> ; the dog that kiss -s the girl give -s to the mouse the cat . # ['the', 'X', 'that', 'X', '-s', 'the', 'X', 'X', '-s', 'to', 'the', 'X', 'the', 'X', '.']
154.	give dog cat mouse , kiss guy dog <o> APRO , OAP </o> ; the dog that the guy kiss -s give -s to the mouse the cat . # ['the', 'X', 'that', 'the', 'X', 'X', '-s', 'X', '-s', 'to', 'the', 'X', 'the', 'X', '.']
155.	give dog cat mouse , kiss mouse girl <o> APRO , APO </o> ; the dog give -s to the mouse that kiss -s the girl the cat . # ['the', 'X', 'X', '-s', 'to', 'the', 'X', 'that', 'X', '-s', 'the', 'X', 'the', 'X', '.']
156.	give dog cat mouse , kiss guy mouse <o> APRO , OAP </o> ; the dog give -s to the mouse that the guy kiss -s the cat . # ['the', 'X', 'X', '-s', 'to', 'the', 'X', 'that', 'the', 'X', 'X', '-s', 'the', 'X', '.']
157.	give dog cat mouse , kiss cat girl <o> APRO , APO </o> ; the dog give -s to the mouse the cat that kiss -s the girl . # ['the', 'X', 'X', '-s', 'to', 'the', 'X', 'the', 'X', 'that', 'X', '-s', 'the', 'X', '.']
158.	give dog cat mouse , kiss guy cat <o> APRO , OAP </o> ; the dog give -s to the mouse the cat that the guy kiss -s . # ['the', 'X', 'X', '-s', 'to', 'the', 'X', 'the', 'X', 'that', 'the', 'X', 'X', '-s', '.']
159.	give dog cat mouse , kiss dog girl <o> ARPO , APO </o> ; the dog that kiss -s the girl to the mouse give -s the cat . # ['the', 'X', 'that', 'X', '-s', 'the', 'X', 'to', 'the', 'X', 'X', '-s', 'the', 'X', '.']
160.	give dog cat mouse , kiss guy dog <o> ARPO , OAP </o> ; the dog that the guy kiss -s to the mouse give -s the cat . # ['the', 'X', 'that', 'the', 'X', 'X', '-s', 'to', 'the', 'X', 'X', '-s', 'the', 'X', '.']
161.	give dog cat mouse , kiss mouse girl <o> ARPO , APO </o> ; the dog to the mouse that kiss -s the girl give -s the cat . # ['the', 'X', 'to', 'the', 'X', 'that', 'X', '-s', 'the', 'X', 'X', '-s', 'the', 'X', '.']
162.	give dog cat mouse , kiss guy mouse <o> ARPO , OAP </o> ; the dog to the mouse that the guy kiss -s give -s the cat . # ['the', 'X', 'to', 'the', 'X', 'that', 'the', 'X', 'X', '-s', 'X', '-s', 'the', 'X', '.']
163.	give dog cat mouse , kiss cat girl <o> ARPO , APO </o> ; the dog to the mouse give -s the cat that kiss -s the girl . # ['the', 'X', 'to', 'the', 'X', 'X', '-s', 'the', 'X', 'that', 'X', '-s', 'the', 'X', '.']
164.	give dog cat mouse , kiss guy cat <o> ARPO , OAP </o> ; the dog to the mouse give -s the cat that the guy kiss -s . # ['the', 'X', 'to', 'the', 'X', 'X', '-s', 'the', 'X', 'that', 'the', 'X', 'X', '-s', '.']
165.	cut beaver fish , kiss beaver girl <o> PAO , APO </o> ; cut -ed by the beaver that kiss -s the girl was the fish . # ['X', '-ed', 'by', 'the', 'X', 'that', 'X', '-s', 'the', 'X', 'was', 'the', 'X', '.']
166.	cut beaver fish , kiss guy beaver <o> PAO , OAP </o> ; cut -ed by the beaver that the guy kiss -s was the fish . # ['X', '-ed', 'by', 'the', 'X', 'that', 'the', 'X', 'X', '-s', 'was', 'the', 'X', '.']
167.	cut beaver fish , kiss fish girl <o> PAO , APO </o> ; cut -ed by the beaver was the fish that kiss -s the girl . # ['X', '-ed', 'by', 'the', 'X', 'was', 'the', 'X', 'that', 'X', '-s', 'the', 'X', '.']
168.	cut beaver fish , kiss guy fish <o> PAO , OAP </o> ; cut -ed by the beaver was the fish that the guy kiss -s . # ['X', '-ed', 'by', 'the', 'X', 'was', 'the', 'X', 'that', 'the', 'X', 'X', '-s', '.']
169.	cut beaver fish , kiss guy fish <o> POA , OPA </o> ; cut -ed was the fish that was kiss -ed by the guy by the beaver . # ['X', '-ed', 'was', 'the', 'X', 'that', 'was', 'X', '-ed', 'by', 'the', 'X', 'by', 'the', 'X', '.']
170.	cut beaver fish , kiss guy beaver <o> POA , OPA </o> ; cut -ed was the fish by the beaver that was kiss -ed by the guy . # ['X', '-ed', 'was', 'the', 'X', 'by', 'the', 'X', 'that', 'was', 'X', '-ed', 'by', 'the', 'X', '.']
171.	cut beaver fish , kiss guy fish <o> OAP , OPA </o> ; the fish that was kiss -ed by the guy , the beaver cut -ed it . # ['the', 'X', 'that', 'was', 'X', '-ed', 'by', 'the', 'X', ',', 'the', 'X', 'X', '-ed', 'it', '.']
172.	cut beaver fish , kiss guy beaver <o> OAP , OPA </o> ; the fish , the beaver that was kiss -ed by the guy cut -ed it . # ['the', 'X', ',', 'the', 'X', 'that', 'was', 'X', '-ed', 'by', 'the', 'X', 'X', '-ed', 'it', '.']
173.	cut beaver fish , kiss fish girl <o> OPA , APO </o> ; the fish that kiss -s the girl was cut -ed by the beaver . # ['the', 'X', 'that', 'X', '-s', 'the', 'X', 'was', 'X', '-ed', 'by', 'the', 'X', '.']
174.	cut beaver fish , kiss guy fish <o> OPA , OAP </o> ; the fish that the guy kiss -s was cut -ed by the beaver . # ['the', 'X', 'that', 'the', 'X', 'X', '-s', 'was', 'X', '-ed', 'by', 'the', 'X', '.']
175.	cut beaver fish , kiss beaver girl <o> OPA , APO </o> ; the fish was cut -ed by the beaver that kiss -s the girl . # ['the', 'X', 'was', 'X', '-ed', 'by', 'the', 'X', 'that', 'X', '-s', 'the', 'X', '.']
176.	cut beaver fish , kiss guy beaver <o> OPA , OAP </o> ; the fish was cut -ed by the beaver that the guy kiss -s . # ['the', 'X', 'was', 'X', '-ed', 'by', 'the', 'X', 'that', 'the', 'X', 'X', '-s', '.']
177.	give dog cat mouse , kiss cat girl <o> PORA , APO </o> ; give the cat that kiss -s the girl to the mouse , the dog did . # ['X', 'the', 'X', 'that', 'X', '-s', 'the', 'X', 'to', 'the', 'X', ',', 'the', 'X', 'X', '.']
178.	give dog cat mouse , kiss guy cat <o> PORA , OAP </o> ; give the cat that the guy kiss -s to the mouse , the dog did . # ['X', 'the', 'X', 'that', 'the', 'X', 'X', '-s', 'to', 'the', 'X', ',', 'the', 'X', 'X', '.']
179.	give dog cat mouse , kiss mouse girl <o> PORA , APO </o> ; give the cat to the mouse that kiss -s the girl , the dog did . # ['X', 'the', 'X', 'to', 'the', 'X', 'that', 'X', '-s', 'the', 'X', ',', 'the', 'X', 'X', '.']
180.	give dog cat mouse , kiss guy mouse <o> PORA , OAP </o> ; give the cat to the mouse that the guy kiss -s , the dog did . # ['X', 'the', 'X', 'to', 'the', 'X', 'that', 'the', 'X', 'X', '-s', ',', 'the', 'X', 'X', '.']
181.	give dog cat mouse , kiss dog girl <o> PORA , APO </o> ; give the cat to the mouse , the dog that kiss -s the girl did . # ['X', 'the', 'X', 'to', 'the', 'X', ',', 'the', 'X', 'that', 'X', '-s', 'the', 'X', 'X', '.']
182.	give dog cat mouse , kiss guy dog <o> PORA , OAP </o> ; give the cat to the mouse , the dog that the guy kiss -s did . # ['X', 'the', 'X', 'to', 'the', 'X', ',', 'the', 'X', 'that', 'the', 'X', 'X', '-s', 'X', '.']
183.	give dog cat mouse , kiss mouse girl <o> RAPO , APO </o> ; to the mouse that kiss -s the girl the dog give -s the cat . # ['to', 'the', 'X', 'that', 'X', '-s', 'the', 'X', 'the', 'X', 'X', '-s', 'the', 'X', '.']
184.	give dog cat mouse , kiss guy mouse <o> RAPO , OAP </o> ; to the mouse that the guy kiss -s the dog give -s the cat . # ['to', 'the', 'X', 'that', 'the', 'X', 'X', '-s', 'the', 'X', 'X', '-s', 'the', 'X', '.']
185.	give dog cat mouse , kiss dog girl <o> RAPO , APO </o> ; to the mouse the dog that kiss -s the girl give -s the cat . # ['to', 'the', 'X', 'the', 'X', 'that', 'X', '-s', 'the', 'X', 'X', '-s', 'the', 'X', '.']
186.	give dog cat mouse , kiss guy dog <o> RAPO , OAP </o> ; to the mouse the dog that the guy kiss -s give -s the cat . # ['to', 'the', 'X', 'the', 'X', 'that', 'the', 'X', 'X', '-s', 'X', '-s', 'the', 'X', '.']
187.	give dog cat mouse , kiss cat girl <o> RAPO , APO </o> ; to the mouse the dog give -s the cat that kiss -s the girl . # ['to', 'the', 'X', 'the', 'X', 'X', '-s', 'the', 'X', 'that', 'X', '-s', 'the', 'X', '.']
188.	give dog cat mouse , kiss guy cat <o> RAPO , OAP </o> ; to the mouse the dog give -s the cat that the guy kiss -s . # ['to', 'the', 'X', 'the', 'X', 'X', '-s', 'the', 'X', 'that', 'the', 'X', 'X', '-s', '.']
189.	cut beaver fish , kiss guy beaver <o> AOP , OPA </o> ; by the beaver that was kiss -ed by the guy the fish was cut -ed . # ['by', 'the', 'X', 'that', 'was', 'X', '-ed', 'by', 'the', 'X', 'the', 'X', 'was', 'X', '-ed', '.']
190.	cut beaver fish , kiss guy fish <o> AOP , OPA </o> ; by the beaver the fish that was kiss -ed by the guy was cut -ed . # ['by', 'the', 'X', 'the', 'X', 'that', 'was', 'X', '-ed', 'by', 'the', 'X', 'was', 'X', '-ed', '.']
191.	cut beaver fish , kiss fish girl <o> POA , AOP </o> ; cut -ed was the fish that the girl was kiss -ed by by the beaver . # ['X', '-ed', 'was', 'the', 'X', 'that', 'the', 'X', 'was', 'X', '-ed', 'by', 'by', 'the', 'X', '.']
192.	cut beaver fish , kiss beaver girl <o> POA , AOP </o> ; cut -ed was the fish by the beaver that the girl was kiss -ed by . # ['X', '-ed', 'was', 'the', 'X', 'by', 'the', 'X', 'that', 'the', 'X', 'was', 'X', '-ed', 'by', '.']
193.	cut beaver fish , kiss fish girl <o> OAP , AOP </o> ; the fish that the girl was kiss -ed by , the beaver cut -ed it . # ['the', 'X', 'that', 'the', 'X', 'was', 'X', '-ed', 'by', ',', 'the', 'X', 'X', '-ed', 'it', '.']
194.	cut beaver fish , kiss beaver girl <o> OAP , AOP </o> ; the fish , the beaver that the girl was kiss -ed by cut -ed it . # ['the', 'X', ',', 'the', 'X', 'that', 'the', 'X', 'was', 'X', '-ed', 'by', 'X', '-ed', 'it', '.']
195.	give dog cat mouse , kiss dog girl <o> PAOR , APO </o> ; give -ed by the dog that kiss -s the girl was the cat to the mouse . # ['X', '-ed', 'by', 'the', 'X', 'that', 'X', '-s', 'the', 'X', 'was', 'the', 'X', 'to', 'the', 'X', '.']
196.	give dog cat mouse , kiss guy dog <o> PAOR , OAP </o> ; give -ed by the dog that the guy kiss -s was the cat to the mouse . # ['X', '-ed', 'by', 'the', 'X', 'that', 'the', 'X', 'X', '-s', 'was', 'the', 'X', 'to', 'the', 'X', '.']
197.	give dog cat mouse , kiss cat girl <o> PAOR , APO </o> ; give -ed by the dog was the cat that kiss -s the girl to the mouse . # ['X', '-ed', 'by', 'the', 'X', 'was', 'the', 'X', 'that', 'X', '-s', 'the', 'X', 'to', 'the', 'X', '.']
198.	give dog cat mouse , kiss guy cat <o> PAOR , OAP </o> ; give -ed by the dog was the cat that the guy kiss -s to the mouse . # ['X', '-ed', 'by', 'the', 'X', 'was', 'the', 'X', 'that', 'the', 'X', 'X', '-s', 'to', 'the', 'X', '.']
199.	give dog cat mouse , kiss mouse girl <o> PAOR , APO </o> ; give -ed by the dog was the cat to the mouse that kiss -s the girl . # ['X', '-ed', 'by', 'the', 'X', 'was', 'the', 'X', 'to', 'the', 'X', 'that', 'X', '-s', 'the', 'X', '.']
200.	give dog cat mouse , kiss guy mouse <o> PAOR , OAP </o> ; give -ed by the dog was the cat to the mouse that the guy kiss -s . # ['X', '-ed', 'by', 'the', 'X', 'was', 'the', 'X', 'to', 'the', 'X', 'that', 'the', 'X', 'X', '-s', '.']
201.	give dog cat mouse , kiss dog girl <o> PARO , APO </o> ; give -ed by the dog that kiss -s the girl to the mouse was the cat . # ['X', '-ed', 'by', 'the', 'X', 'that', 'X', '-s', 'the', 'X', 'to', 'the', 'X', 'was', 'the', 'X', '.']
202.	give dog cat mouse , kiss guy dog <o> PARO , OAP </o> ; give -ed by the dog that the guy kiss -s to the mouse was the cat . # ['X', '-ed', 'by', 'the', 'X', 'that', 'the', 'X', 'X', '-s', 'to', 'the', 'X', 'was', 'the', 'X', '.']
203.	give dog cat mouse , kiss mouse girl <o> PARO , APO </o> ; give -ed by the dog to the mouse that kiss -s the girl was the cat . # ['X', '-ed', 'by', 'the', 'X', 'to', 'the', 'X', 'that', 'X', '-s', 'the', 'X', 'was', 'the', 'X', '.']
204.	give dog cat mouse , kiss guy mouse <o> PARO , OAP </o> ; give -ed by the dog to the mouse that the guy kiss -s was the cat . # ['X', '-ed', 'by', 'the', 'X', 'to', 'the', 'X', 'that', 'the', 'X', 'X', '-s', 'was', 'the', 'X', '.']
205.	give dog cat mouse , kiss cat girl <o> PARO , APO </o> ; give -ed by the dog to the mouse was the cat that kiss -s the girl . # ['X', '-ed', 'by', 'the', 'X', 'to', 'the', 'X', 'was', 'the', 'X', 'that', 'X', '-s', 'the', 'X', '.']
206.	give dog cat mouse , kiss guy cat <o> PARO , OAP </o> ; give -ed by the dog to the mouse was the cat that the guy kiss -s . # ['X', '-ed', 'by', 'the', 'X', 'to', 'the', 'X', 'was', 'the', 'X', 'that', 'the', 'X', 'X', '-s', '.']
207.	give dog cat mouse , kiss cat girl <o> POAR , APO </o> ; give -ed was the cat that kiss -s the girl by the dog to the mouse . # ['X', '-ed', 'was', 'the', 'X', 'that', 'X', '-s', 'the', 'X', 'by', 'the', 'X', 'to', 'the', 'X', '.']
208.	give dog cat mouse , kiss guy cat <o> POAR , OAP </o> ; give -ed was the cat that the guy kiss -s by the dog to the mouse . # ['X', '-ed', 'was', 'the', 'X', 'that', 'the', 'X', 'X', '-s', 'by', 'the', 'X', 'to', 'the', 'X', '.']
209.	give dog cat mouse , kiss dog girl <o> POAR , APO </o> ; give -ed was the cat by the dog that kiss -s the girl to the mouse . # ['X', '-ed', 'was', 'the', 'X', 'by', 'the', 'X', 'that', 'X', '-s', 'the', 'X', 'to', 'the', 'X', '.']
210.	give dog cat mouse , kiss guy dog <o> POAR , OAP </o> ; give -ed was the cat by the dog that the guy kiss -s to the mouse . # ['X', '-ed', 'was', 'the', 'X', 'by', 'the', 'X', 'that', 'the', 'X', 'X', '-s', 'to', 'the', 'X', '.']
211.	give dog cat mouse , kiss mouse girl <o> POAR , APO </o> ; give -ed was the cat by the dog to the mouse that kiss -s the girl . # ['X', '-ed', 'was', 'the', 'X', 'by', 'the', 'X', 'to', 'the', 'X', 'that', 'X', '-s', 'the', 'X', '.']
212.	give dog cat mouse , kiss guy mouse <o> POAR , OAP </o> ; give -ed was the cat by the dog to the mouse that the guy kiss -s . # ['X', '-ed', 'was', 'the', 'X', 'by', 'the', 'X', 'to', 'the', 'X', 'that', 'the', 'X', 'X', '-s', '.']
213.	give dog cat mouse , kiss mouse girl <o> PRAO , APO </o> ; give -ed to the mouse that kiss -s the girl by the dog was the cat . # ['X', '-ed', 'to', 'the', 'X', 'that', 'X', '-s', 'the', 'X', 'by', 'the', 'X', 'was', 'the', 'X', '.']
214.	give dog cat mouse , kiss guy mouse <o> PRAO , OAP </o> ; give -ed to the mouse that the guy kiss -s by the dog was the cat . # ['X', '-ed', 'to', 'the', 'X', 'that', 'the', 'X', 'X', '-s', 'by', 'the', 'X', 'was', 'the', 'X', '.']
215.	give dog cat mouse , kiss dog girl <o> PRAO , APO </o> ; give -ed to the mouse by the dog that kiss -s the girl was the cat . # ['X', '-ed', 'to', 'the', 'X', 'by', 'the', 'X', 'that', 'X', '-s', 'the', 'X', 'was', 'the', 'X', '.']
216.	give dog cat mouse , kiss guy dog <o> PRAO , OAP </o> ; give -ed to the mouse by the dog that the guy kiss -s was the cat . # ['X', '-ed', 'to', 'the', 'X', 'by', 'the', 'X', 'that', 'the', 'X', 'X', '-s', 'was', 'the', 'X', '.']
217.	give dog cat mouse , kiss cat girl <o> PRAO , APO </o> ; give -ed to the mouse by the dog was the cat that kiss -s the girl . # ['X', '-ed', 'to', 'the', 'X', 'by', 'the', 'X', 'was', 'the', 'X', 'that', 'X', '-s', 'the', 'X', '.']
218.	give dog cat mouse , kiss guy cat <o> PRAO , OAP </o> ; give -ed to the mouse by the dog was the cat that the guy kiss -s . # ['X', '-ed', 'to', 'the', 'X', 'by', 'the', 'X', 'was', 'the', 'X', 'that', 'the', 'X', 'X', '-s', '.']
219.	give dog cat mouse , kiss mouse girl <o> PROA , APO </o> ; give -ed to the mouse that kiss -s the girl was the cat by the dog . # ['X', '-ed', 'to', 'the', 'X', 'that', 'X', '-s', 'the', 'X', 'was', 'the', 'X', 'by', 'the', 'X', '.']
220.	give dog cat mouse , kiss guy mouse <o> PROA , OAP </o> ; give -ed to the mouse that the guy kiss -s was the cat by the dog . # ['X', '-ed', 'to', 'the', 'X', 'that', 'the', 'X', 'X', '-s', 'was', 'the', 'X', 'by', 'the', 'X', '.']
221.	give dog cat mouse , kiss cat girl <o> PROA , APO </o> ; give -ed to the mouse was the cat that kiss -s the girl by the dog . # ['X', '-ed', 'to', 'the', 'X', 'was', 'the', 'X', 'that', 'X', '-s', 'the', 'X', 'by', 'the', 'X', '.']
222.	give dog cat mouse , kiss guy cat <o> PROA , OAP </o> ; give -ed to the mouse was the cat that the guy kiss -s by the dog . # ['X', '-ed', 'to', 'the', 'X', 'was', 'the', 'X', 'that', 'the', 'X', 'X', '-s', 'by', 'the', 'X', '.']
223.	give dog cat mouse , kiss dog girl <o> PROA , APO </o> ; give -ed to the mouse was the cat by the dog that kiss -s the girl . # ['X', '-ed', 'to', 'the', 'X', 'was', 'the', 'X', 'by', 'the', 'X', 'that', 'X', '-s', 'the', 'X', '.']
224.	give dog cat mouse , kiss guy dog <o> PROA , OAP </o> ; give -ed to the mouse was the cat by the dog that the guy kiss -s . # ['X', '-ed', 'to', 'the', 'X', 'was', 'the', 'X', 'by', 'the', 'X', 'that', 'the', 'X', 'X', '-s', '.']
225.	give dog cat mouse , kiss guy dog <o> APOR , OPA </o> ; the dog that was kiss -ed by the guy give -s the cat to the mouse . # ['the', 'X', 'that', 'was', 'X', '-ed', 'by', 'the', 'X', 'X', '-s', 'the', 'X', 'to', 'the', 'X', '.']
226.	give dog cat mouse , kiss guy cat <o> APOR , OPA </o> ; the dog give -s the cat that was kiss -ed by the guy to the mouse . # ['the', 'X', 'X', '-s', 'the', 'X', 'that', 'was', 'X', '-ed', 'by', 'the', 'X', 'to', 'the', 'X', '.']
227.	give dog cat mouse , kiss guy mouse <o> APOR , OPA </o> ; the dog give -s the cat to the mouse that was kiss -ed by the guy . # ['the', 'X', 'X', '-s', 'the', 'X', 'to', 'the', 'X', 'that', 'was', 'X', '-ed', 'by', 'the', 'X', '.']
228.	give dog cat mouse , kiss guy dog <o> APRO , OPA </o> ; the dog that was kiss -ed by the guy give -s to the mouse the cat . # ['the', 'X', 'that', 'was', 'X', '-ed', 'by', 'the', 'X', 'X', '-s', 'to', 'the', 'X', 'the', 'X', '.']
229.	give dog cat mouse , kiss guy mouse <o> APRO , OPA </o> ; the dog give -s to the mouse that was kiss -ed by the guy the cat . # ['the', 'X', 'X', '-s', 'to', 'the', 'X', 'that', 'was', 'X', '-ed', 'by', 'the', 'X', 'the', 'X', '.']
230.	give dog cat mouse , kiss guy cat <o> APRO , OPA </o> ; the dog give -s to the mouse the cat that was kiss -ed by the guy . # ['the', 'X', 'X', '-s', 'to', 'the', 'X', 'the', 'X', 'that', 'was', 'X', '-ed', 'by', 'the', 'X', '.']
231.	give dog cat mouse , kiss guy dog <o> ARPO , OPA </o> ; the dog that was kiss -ed by the guy to the mouse give -s the cat . # ['the', 'X', 'that', 'was', 'X', '-ed', 'by', 'the', 'X', 'to', 'the', 'X', 'X', '-s', 'the', 'X', '.']
232.	give dog cat mouse , kiss guy mouse <o> ARPO , OPA </o> ; the dog to the mouse that was kiss -ed by the guy give -s the cat . # ['the', 'X', 'to', 'the', 'X', 'that', 'was', 'X', '-ed', 'by', 'the', 'X', 'X', '-s', 'the', 'X', '.']
233.	give dog cat mouse , kiss guy cat <o> ARPO , OPA </o> ; the dog to the mouse give -s the cat that was kiss -ed by the guy . # ['the', 'X', 'to', 'the', 'X', 'X', '-s', 'the', 'X', 'that', 'was', 'X', '-ed', 'by', 'the', 'X', '.']
234.	give dog cat mouse , kiss cat girl <o> OPAR , APO </o> ; the cat that kiss -s the girl was give -ed by the dog to the mouse . # ['the', 'X', 'that', 'X', '-s', 'the', 'X', 'was', 'X', '-ed', 'by', 'the', 'X', 'to', 'the', 'X', '.']
235.	give dog cat mouse , kiss guy cat <o> OPAR , OAP </o> ; the cat that the guy kiss -s was give -ed by the dog to the mouse . # ['the', 'X', 'that', 'the', 'X', 'X', '-s', 'was', 'X', '-ed', 'by', 'the', 'X', 'to', 'the', 'X', '.']
236.	give dog cat mouse , kiss dog girl <o> OPAR , APO </o> ; the cat was give -ed by the dog that kiss -s the girl to the mouse . # ['the', 'X', 'was', 'X', '-ed', 'by', 'the', 'X', 'that', 'X', '-s', 'the', 'X', 'to', 'the', 'X', '.']
237.	give dog cat mouse , kiss guy dog <o> OPAR , OAP </o> ; the cat was give -ed by the dog that the guy kiss -s to the mouse . # ['the', 'X', 'was', 'X', '-ed', 'by', 'the', 'X', 'that', 'the', 'X', 'X', '-s', 'to', 'the', 'X', '.']
238.	give dog cat mouse , kiss mouse girl <o> OPAR , APO </o> ; the cat was give -ed by the dog to the mouse that kiss -s the girl . # ['the', 'X', 'was', 'X', '-ed', 'by', 'the', 'X', 'to', 'the', 'X', 'that', 'X', '-s', 'the', 'X', '.']
239.	give dog cat mouse , kiss guy mouse <o> OPAR , OAP </o> ; the cat was give -ed by the dog to the mouse that the guy kiss -s . # ['the', 'X', 'was', 'X', '-ed', 'by', 'the', 'X', 'to', 'the', 'X', 'that', 'the', 'X', 'X', '-s', '.']
240.	give dog cat mouse , kiss cat girl <o> OPRA , APO </o> ; the cat that kiss -s the girl was give -ed to the mouse by the dog . # ['the', 'X', 'that', 'X', '-s', 'the', 'X', 'was', 'X', '-ed', 'to', 'the', 'X', 'by', 'the', 'X', '.']
241.	give dog cat mouse , kiss guy cat <o> OPRA , OAP </o> ; the cat that the guy kiss -s was give -ed to the mouse by the dog . # ['the', 'X', 'that', 'the', 'X', 'X', '-s', 'was', 'X', '-ed', 'to', 'the', 'X', 'by', 'the', 'X', '.']
242.	give dog cat mouse , kiss mouse girl <o> OPRA , APO </o> ; the cat was give -ed to the mouse that kiss -s the girl by the dog . # ['the', 'X', 'was', 'X', '-ed', 'to', 'the', 'X', 'that', 'X', '-s', 'the', 'X', 'by', 'the', 'X', '.']
243.	give dog cat mouse , kiss guy mouse <o> OPRA , OAP </o> ; the cat was give -ed to the mouse that the guy kiss -s by the dog . # ['the', 'X', 'was', 'X', '-ed', 'to', 'the', 'X', 'that', 'the', 'X', 'X', '-s', 'by', 'the', 'X', '.']
244.	give dog cat mouse , kiss dog girl <o> OPRA , APO </o> ; the cat was give -ed to the mouse by the dog that kiss -s the girl . # ['the', 'X', 'was', 'X', '-ed', 'to', 'the', 'X', 'by', 'the', 'X', 'that', 'X', '-s', 'the', 'X', '.']
245.	give dog cat mouse , kiss guy dog <o> OPRA , OAP </o> ; the cat was give -ed to the mouse by the dog that the guy kiss -s . # ['the', 'X', 'was', 'X', '-ed', 'to', 'the', 'X', 'by', 'the', 'X', 'that', 'the', 'X', 'X', '-s', '.']
246.	give dog cat mouse , kiss cat girl <o> OAPR , APO </o> ; the cat that kiss -s the girl by the dog was give -ed to the mouse . # ['the', 'X', 'that', 'X', '-s', 'the', 'X', 'by', 'the', 'X', 'was', 'X', '-ed', 'to', 'the', 'X', '.']
247.	give dog cat mouse , kiss guy cat <o> OAPR , OAP </o> ; the cat that the guy kiss -s by the dog was give -ed to the mouse . # ['the', 'X', 'that', 'the', 'X', 'X', '-s', 'by', 'the', 'X', 'was', 'X', '-ed', 'to', 'the', 'X', '.']
248.	give dog cat mouse , kiss dog girl <o> OAPR , APO </o> ; the cat by the dog that kiss -s the girl was give -ed to the mouse . # ['the', 'X', 'by', 'the', 'X', 'that', 'X', '-s', 'the', 'X', 'was', 'X', '-ed', 'to', 'the', 'X', '.']
249.	give dog cat mouse , kiss guy dog <o> OAPR , OAP </o> ; the cat by the dog that the guy kiss -s was give -ed to the mouse . # ['the', 'X', 'by', 'the', 'X', 'that', 'the', 'X', 'X', '-s', 'was', 'X', '-ed', 'to', 'the', 'X', '.']
250.	give dog cat mouse , kiss mouse girl <o> OAPR , APO </o> ; the cat by the dog was give -ed to the mouse that kiss -s the girl . # ['the', 'X', 'by', 'the', 'X', 'was', 'X', '-ed', 'to', 'the', 'X', 'that', 'X', '-s', 'the', 'X', '.']
251.	give dog cat mouse , kiss guy mouse <o> OAPR , OAP </o> ; the cat by the dog was give -ed to the mouse that the guy kiss -s . # ['the', 'X', 'by', 'the', 'X', 'was', 'X', '-ed', 'to', 'the', 'X', 'that', 'the', 'X', 'X', '-s', '.']
252.	give dog cat mouse , kiss cat girl <o> OARP , APO </o> ; the cat that kiss -s the girl by the dog to the mouse was give -ed . # ['the', 'X', 'that', 'X', '-s', 'the', 'X', 'by', 'the', 'X', 'to', 'the', 'X', 'was', 'X', '-ed', '.']
253.	give dog cat mouse , kiss guy cat <o> OARP , OAP </o> ; the cat that the guy kiss -s by the dog to the mouse was give -ed . # ['the', 'X', 'that', 'the', 'X', 'X', '-s', 'by', 'the', 'X', 'to', 'the', 'X', 'was', 'X', '-ed', '.']
254.	give dog cat mouse , kiss dog girl <o> OARP , APO </o> ; the cat by the dog that kiss -s the girl to the mouse was give -ed . # ['the', 'X', 'by', 'the', 'X', 'that', 'X', '-s', 'the', 'X', 'to', 'the', 'X', 'was', 'X', '-ed', '.']
255.	give dog cat mouse , kiss guy dog <o> OARP , OAP </o> ; the cat by the dog that the guy kiss -s to the mouse was give -ed . # ['the', 'X', 'by', 'the', 'X', 'that', 'the', 'X', 'X', '-s', 'to', 'the', 'X', 'was', 'X', '-ed', '.']
256.	give dog cat mouse , kiss mouse girl <o> OARP , APO </o> ; the cat by the dog to the mouse that kiss -s the girl was give -ed . # ['the', 'X', 'by', 'the', 'X', 'to', 'the', 'X', 'that', 'X', '-s', 'the', 'X', 'was', 'X', '-ed', '.']
257.	give dog cat mouse , kiss guy mouse <o> OARP , OAP </o> ; the cat by the dog to the mouse that the guy kiss -s was give -ed . # ['the', 'X', 'by', 'the', 'X', 'to', 'the', 'X', 'that', 'the', 'X', 'X', '-s', 'was', 'X', '-ed', '.']
258.	give dog cat mouse , kiss cat girl <o> ORPA , APO </o> ; the cat that kiss -s the girl to the mouse was give -ed by the dog . # ['the', 'X', 'that', 'X', '-s', 'the', 'X', 'to', 'the', 'X', 'was', 'X', '-ed', 'by', 'the', 'X', '.']
259.	give dog cat mouse , kiss guy cat <o> ORPA , OAP </o> ; the cat that the guy kiss -s to the mouse was give -ed by the dog . # ['the', 'X', 'that', 'the', 'X', 'X', '-s', 'to', 'the', 'X', 'was', 'X', '-ed', 'by', 'the', 'X', '.']
260.	give dog cat mouse , kiss mouse girl <o> ORPA , APO </o> ; the cat to the mouse that kiss -s the girl was give -ed by the dog . # ['the', 'X', 'to', 'the', 'X', 'that', 'X', '-s', 'the', 'X', 'was', 'X', '-ed', 'by', 'the', 'X', '.']
261.	give dog cat mouse , kiss guy mouse <o> ORPA , OAP </o> ; the cat to the mouse that the guy kiss -s was give -ed by the dog . # ['the', 'X', 'to', 'the', 'X', 'that', 'the', 'X', 'X', '-s', 'was', 'X', '-ed', 'by', 'the', 'X', '.']
262.	give dog cat mouse , kiss dog girl <o> ORPA , APO </o> ; the cat to the mouse was give -ed by the dog that kiss -s the girl . # ['the', 'X', 'to', 'the', 'X', 'was', 'X', '-ed', 'by', 'the', 'X', 'that', 'X', '-s', 'the', 'X', '.']
263.	give dog cat mouse , kiss guy dog <o> ORPA , OAP </o> ; the cat to the mouse was give -ed by the dog that the guy kiss -s . # ['the', 'X', 'to', 'the', 'X', 'was', 'X', '-ed', 'by', 'the', 'X', 'that', 'the', 'X', 'X', '-s', '.']
264.	give dog cat mouse , kiss cat girl <o> ORAP , APO </o> ; the cat that kiss -s the girl to the mouse by the dog was give -ed . # ['the', 'X', 'that', 'X', '-s', 'the', 'X', 'to', 'the', 'X', 'by', 'the', 'X', 'was', 'X', '-ed', '.']
265.	give dog cat mouse , kiss guy cat <o> ORAP , OAP </o> ; the cat that the guy kiss -s to the mouse by the dog was give -ed . # ['the', 'X', 'that', 'the', 'X', 'X', '-s', 'to', 'the', 'X', 'by', 'the', 'X', 'was', 'X', '-ed', '.']
266.	give dog cat mouse , kiss mouse girl <o> ORAP , APO </o> ; the cat to the mouse that kiss -s the girl by the dog was give -ed . # ['the', 'X', 'to', 'the', 'X', 'that', 'X', '-s', 'the', 'X', 'by', 'the', 'X', 'was', 'X', '-ed', '.']
267.	give dog cat mouse , kiss guy mouse <o> ORAP , OAP </o> ; the cat to the mouse that the guy kiss -s by the dog was give -ed . # ['the', 'X', 'to', 'the', 'X', 'that', 'the', 'X', 'X', '-s', 'by', 'the', 'X', 'was', 'X', '-ed', '.']
268.	give dog cat mouse , kiss dog girl <o> ORAP , APO </o> ; the cat to the mouse by the dog that kiss -s the girl was give -ed . # ['the', 'X', 'to', 'the', 'X', 'by', 'the', 'X', 'that', 'X', '-s', 'the', 'X', 'was', 'X', '-ed', '.']
269.	give dog cat mouse , kiss guy dog <o> ORAP , OAP </o> ; the cat to the mouse by the dog that the guy kiss -s was give -ed . # ['the', 'X', 'to', 'the', 'X', 'by', 'the', 'X', 'that', 'the', 'X', 'X', '-s', 'was', 'X', '-ed', '.']
270.	cut beaver fish , kiss beaver girl <o> AOP , AOP </o> ; by the beaver that the girl was kiss -ed by the fish was cut -ed . # ['by', 'the', 'X', 'that', 'the', 'X', 'was', 'X', '-ed', 'by', 'the', 'X', 'was', 'X', '-ed', '.']
271.	cut beaver fish , kiss fish girl <o> AOP , AOP </o> ; by the beaver the fish that the girl was kiss -ed by was cut -ed . # ['by', 'the', 'X', 'the', 'X', 'that', 'the', 'X', 'was', 'X', '-ed', 'by', 'was', 'X', '-ed', '.']
272.	cut beaver fish , kiss guy beaver <o> PAO , OPA </o> ; cut -ed by the beaver that was kiss -ed by the guy was the fish . # ['X', '-ed', 'by', 'the', 'X', 'that', 'was', 'X', '-ed', 'by', 'the', 'X', 'was', 'the', 'X', '.']
273.	cut beaver fish , kiss guy fish <o> PAO , OPA </o> ; cut -ed by the beaver was the fish that was kiss -ed by the guy . # ['X', '-ed', 'by', 'the', 'X', 'was', 'the', 'X', 'that', 'was', 'X', '-ed', 'by', 'the', 'X', '.']
274.	cut beaver fish , kiss guy fish <o> OPA , OPA </o> ; the fish that was kiss -ed by the guy was cut -ed by the beaver . # ['the', 'X', 'that', 'was', 'X', '-ed', 'by', 'the', 'X', 'was', 'X', '-ed', 'by', 'the', 'X', '.']
275.	cut beaver fish , kiss guy beaver <o> OPA , OPA </o> ; the fish was cut -ed by the beaver that was kiss -ed by the guy . # ['the', 'X', 'was', 'X', '-ed', 'by', 'the', 'X', 'that', 'was', 'X', '-ed', 'by', 'the', 'X', '.']
276.	give dog cat mouse , kiss guy cat <o> PORA , OPA </o> ; give the cat that was kiss -ed by the guy to the mouse , the dog did . # ['X', 'the', 'X', 'that', 'was', 'X', '-ed', 'by', 'the', 'X', 'to', 'the', 'X', ',', 'the', 'X', 'X', '.']
277.	give dog cat mouse , kiss guy mouse <o> PORA , OPA </o> ; give the cat to the mouse that was kiss -ed by the guy , the dog did . # ['X', 'the', 'X', 'to', 'the', 'X', 'that', 'was', 'X', '-ed', 'by', 'the', 'X', ',', 'the', 'X', 'X', '.']
278.	give dog cat mouse , kiss guy dog <o> PORA , OPA </o> ; give the cat to the mouse , the dog that was kiss -ed by the guy did . # ['X', 'the', 'X', 'to', 'the', 'X', ',', 'the', 'X', 'that', 'was', 'X', '-ed', 'by', 'the', 'X', 'X', '.']
279.	give dog cat mouse , kiss dog girl <o> APOR , AOP </o> ; the dog that the girl was kiss -ed by give -s the cat to the mouse . # ['the', 'X', 'that', 'the', 'X', 'was', 'X', '-ed', 'by', 'X', '-s', 'the', 'X', 'to', 'the', 'X', '.']
280.	give dog cat mouse , kiss cat girl <o> APOR , AOP </o> ; the dog give -s the cat that the girl was kiss -ed by to the mouse . # ['the', 'X', 'X', '-s', 'the', 'X', 'that', 'the', 'X', 'was', 'X', '-ed', 'by', 'to', 'the', 'X', '.']
281.	give dog cat mouse , kiss mouse girl <o> APOR , AOP </o> ; the dog give -s the cat to the mouse that the girl was kiss -ed by . # ['the', 'X', 'X', '-s', 'the', 'X', 'to', 'the', 'X', 'that', 'the', 'X', 'was', 'X', '-ed', 'by', '.']
282.	give dog cat mouse , kiss dog girl <o> APRO , AOP </o> ; the dog that the girl was kiss -ed by give -s to the mouse the cat . # ['the', 'X', 'that', 'the', 'X', 'was', 'X', '-ed', 'by', 'X', '-s', 'to', 'the', 'X', 'the', 'X', '.']
283.	give dog cat mouse , kiss mouse girl <o> APRO , AOP </o> ; the dog give -s to the mouse that the girl was kiss -ed by the cat . # ['the', 'X', 'X', '-s', 'to', 'the', 'X', 'that', 'the', 'X', 'was', 'X', '-ed', 'by', 'the', 'X', '.']
284.	give dog cat mouse , kiss cat girl <o> APRO , AOP </o> ; the dog give -s to the mouse the cat that the girl was kiss -ed by . # ['the', 'X', 'X', '-s', 'to', 'the', 'X', 'the', 'X', 'that', 'the', 'X', 'was', 'X', '-ed', 'by', '.']
285.	give dog cat mouse , kiss dog girl <o> AOPR , APO </o> ; by the dog that kiss -s the girl the cat was give -ed to the mouse . # ['by', 'the', 'X', 'that', 'X', '-s', 'the', 'X', 'the', 'X', 'was', 'X', '-ed', 'to', 'the', 'X', '.']
286.	give dog cat mouse , kiss guy dog <o> AOPR , OAP </o> ; by the dog that the guy kiss -s the cat was give -ed to the mouse . # ['by', 'the', 'X', 'that', 'the', 'X', 'X', '-s', 'the', 'X', 'was', 'X', '-ed', 'to', 'the', 'X', '.']
287.	give dog cat mouse , kiss cat girl <o> AOPR , APO </o> ; by the dog the cat that kiss -s the girl was give -ed to the mouse . # ['by', 'the', 'X', 'the', 'X', 'that', 'X', '-s', 'the', 'X', 'was', 'X', '-ed', 'to', 'the', 'X', '.']
288.	give dog cat mouse , kiss guy cat <o> AOPR , OAP </o> ; by the dog the cat that the guy kiss -s was give -ed to the mouse . # ['by', 'the', 'X', 'the', 'X', 'that', 'the', 'X', 'X', '-s', 'was', 'X', '-ed', 'to', 'the', 'X', '.']
289.	give dog cat mouse , kiss mouse girl <o> AOPR , APO </o> ; by the dog the cat was give -ed to the mouse that kiss -s the girl . # ['by', 'the', 'X', 'the', 'X', 'was', 'X', '-ed', 'to', 'the', 'X', 'that', 'X', '-s', 'the', 'X', '.']
290.	give dog cat mouse , kiss guy mouse <o> AOPR , OAP </o> ; by the dog the cat was give -ed to the mouse that the guy kiss -s . # ['by', 'the', 'X', 'the', 'X', 'was', 'X', '-ed', 'to', 'the', 'X', 'that', 'the', 'X', 'X', '-s', '.']
291.	give dog cat mouse , kiss dog girl <o> AORP , APO </o> ; by the dog that kiss -s the girl the cat to the mouse was give -ed . # ['by', 'the', 'X', 'that', 'X', '-s', 'the', 'X', 'the', 'X', 'to', 'the', 'X', 'was', 'X', '-ed', '.']
292.	give dog cat mouse , kiss guy dog <o> AORP , OAP </o> ; by the dog that the guy kiss -s the cat to the mouse was give -ed . # ['by', 'the', 'X', 'that', 'the', 'X', 'X', '-s', 'the', 'X', 'to', 'the', 'X', 'was', 'X', '-ed', '.']
293.	give dog cat mouse , kiss cat girl <o> AORP , APO </o> ; by the dog the cat that kiss -s the girl to the mouse was give -ed . # ['by', 'the', 'X', 'the', 'X', 'that', 'X', '-s', 'the', 'X', 'to', 'the', 'X', 'was', 'X', '-ed', '.']
294.	give dog cat mouse , kiss guy cat <o> AORP , OAP </o> ; by the dog the cat that the guy kiss -s to the mouse was give -ed . # ['by', 'the', 'X', 'the', 'X', 'that', 'the', 'X', 'X', '-s', 'to', 'the', 'X', 'was', 'X', '-ed', '.']
295.	give dog cat mouse , kiss mouse girl <o> AORP , APO </o> ; by the dog the cat to the mouse that kiss -s the girl was give -ed . # ['by', 'the', 'X', 'the', 'X', 'to', 'the', 'X', 'that', 'X', '-s', 'the', 'X', 'was', 'X', '-ed', '.']
296.	give dog cat mouse , kiss guy mouse <o> AORP , OAP </o> ; by the dog the cat to the mouse that the guy kiss -s was give -ed . # ['by', 'the', 'X', 'the', 'X', 'to', 'the', 'X', 'that', 'the', 'X', 'X', '-s', 'was', 'X', '-ed', '.']
297.	give dog cat mouse , kiss dog girl <o> ARPO , AOP </o> ; the dog that the girl was kiss -ed by to the mouse give -s the cat . # ['the', 'X', 'that', 'the', 'X', 'was', 'X', '-ed', 'by', 'to', 'the', 'X', 'X', '-s', 'the', 'X', '.']
298.	give dog cat mouse , kiss mouse girl <o> ARPO , AOP </o> ; the dog to the mouse that the girl was kiss -ed by give -s the cat . # ['the', 'X', 'to', 'the', 'X', 'that', 'the', 'X', 'was', 'X', '-ed', 'by', 'X', '-s', 'the', 'X', '.']
299.	give dog cat mouse , kiss cat girl <o> ARPO , AOP </o> ; the dog to the mouse give -s the cat that the girl was kiss -ed by . # ['the', 'X', 'to', 'the', 'X', 'X', '-s', 'the', 'X', 'that', 'the', 'X', 'was', 'X', '-ed', 'by', '.']
300.	give dog cat mouse , kiss dog girl <o> AROP , APO </o> ; by the dog that kiss -s the girl to the mouse the cat was give -ed . # ['by', 'the', 'X', 'that', 'X', '-s', 'the', 'X', 'to', 'the', 'X', 'the', 'X', 'was', 'X', '-ed', '.']
301.	give dog cat mouse , kiss guy dog <o> AROP , OAP </o> ; by the dog that the guy kiss -s to the mouse the cat was give -ed . # ['by', 'the', 'X', 'that', 'the', 'X', 'X', '-s', 'to', 'the', 'X', 'the', 'X', 'was', 'X', '-ed', '.']
302.	give dog cat mouse , kiss mouse girl <o> AROP , APO </o> ; by the dog to the mouse that kiss -s the girl the cat was give -ed . # ['by', 'the', 'X', 'to', 'the', 'X', 'that', 'X', '-s', 'the', 'X', 'the', 'X', 'was', 'X', '-ed', '.']
303.	give dog cat mouse , kiss guy mouse <o> AROP , OAP </o> ; by the dog to the mouse that the guy kiss -s the cat was give -ed . # ['by', 'the', 'X', 'to', 'the', 'X', 'that', 'the', 'X', 'X', '-s', 'the', 'X', 'was', 'X', '-ed', '.']
304.	give dog cat mouse , kiss cat girl <o> AROP , APO </o> ; by the dog to the mouse the cat that kiss -s the girl was give -ed . # ['by', 'the', 'X', 'to', 'the', 'X', 'the', 'X', 'that', 'X', '-s', 'the', 'X', 'was', 'X', '-ed', '.']
305.	give dog cat mouse , kiss guy cat <o> AROP , OAP </o> ; by the dog to the mouse the cat that the guy kiss -s was give -ed . # ['by', 'the', 'X', 'to', 'the', 'X', 'the', 'X', 'that', 'the', 'X', 'X', '-s', 'was', 'X', '-ed', '.']
306.	give dog cat mouse , kiss mouse girl <o> RPAO , APO </o> ; to the mouse that kiss -s the girl was give -ed by the dog the cat . # ['to', 'the', 'X', 'that', 'X', '-s', 'the', 'X', 'was', 'X', '-ed', 'by', 'the', 'X', 'the', 'X', '.']
307.	give dog cat mouse , kiss guy mouse <o> RPAO , OAP </o> ; to the mouse that the guy kiss -s was give -ed by the dog the cat . # ['to', 'the', 'X', 'that', 'the', 'X', 'X', '-s', 'was', 'X', '-ed', 'by', 'the', 'X', 'the', 'X', '.']
308.	give dog cat mouse , kiss dog girl <o> RPAO , APO </o> ; to the mouse was give -ed by the dog that kiss -s the girl the cat . # ['to', 'the', 'X', 'was', 'X', '-ed', 'by', 'the', 'X', 'that', 'X', '-s', 'the', 'X', 'the', 'X', '.']
309.	give dog cat mouse , kiss guy dog <o> RPAO , OAP </o> ; to the mouse was give -ed by the dog that the guy kiss -s the cat . # ['to', 'the', 'X', 'was', 'X', '-ed', 'by', 'the', 'X', 'that', 'the', 'X', 'X', '-s', 'the', 'X', '.']
310.	give dog cat mouse , kiss cat girl <o> RPAO , APO </o> ; to the mouse was give -ed by the dog the cat that kiss -s the girl . # ['to', 'the', 'X', 'was', 'X', '-ed', 'by', 'the', 'X', 'the', 'X', 'that', 'X', '-s', 'the', 'X', '.']
311.	give dog cat mouse , kiss guy cat <o> RPAO , OAP </o> ; to the mouse was give -ed by the dog the cat that the guy kiss -s . # ['to', 'the', 'X', 'was', 'X', '-ed', 'by', 'the', 'X', 'the', 'X', 'that', 'the', 'X', 'X', '-s', '.']
312.	give dog cat mouse , kiss mouse girl <o> RPOA , APO </o> ; to the mouse that kiss -s the girl was give -ed the cat by the dog . # ['to', 'the', 'X', 'that', 'X', '-s', 'the', 'X', 'was', 'X', '-ed', 'the', 'X', 'by', 'the', 'X', '.']
313.	give dog cat mouse , kiss guy mouse <o> RPOA , OAP </o> ; to the mouse that the guy kiss -s was give -ed the cat by the dog . # ['to', 'the', 'X', 'that', 'the', 'X', 'X', '-s', 'was', 'X', '-ed', 'the', 'X', 'by', 'the', 'X', '.']
314.	give dog cat mouse , kiss cat girl <o> RPOA , APO </o> ; to the mouse was give -ed the cat that kiss -s the girl by the dog . # ['to', 'the', 'X', 'was', 'X', '-ed', 'the', 'X', 'that', 'X', '-s', 'the', 'X', 'by', 'the', 'X', '.']
315.	give dog cat mouse , kiss guy cat <o> RPOA , OAP </o> ; to the mouse was give -ed the cat that the guy kiss -s by the dog . # ['to', 'the', 'X', 'was', 'X', '-ed', 'the', 'X', 'that', 'the', 'X', 'X', '-s', 'by', 'the', 'X', '.']
316.	give dog cat mouse , kiss dog girl <o> RPOA , APO </o> ; to the mouse was give -ed the cat by the dog that kiss -s the girl . # ['to', 'the', 'X', 'was', 'X', '-ed', 'the', 'X', 'by', 'the', 'X', 'that', 'X', '-s', 'the', 'X', '.']
317.	give dog cat mouse , kiss guy dog <o> RPOA , OAP </o> ; to the mouse was give -ed the cat by the dog that the guy kiss -s . # ['to', 'the', 'X', 'was', 'X', '-ed', 'the', 'X', 'by', 'the', 'X', 'that', 'the', 'X', 'X', '-s', '.']
318.	give dog cat mouse , kiss guy mouse <o> RAPO , OPA </o> ; to the mouse that was kiss -ed by the guy the dog give -s the cat . # ['to', 'the', 'X', 'that', 'was', 'X', '-ed', 'by', 'the', 'X', 'the', 'X', 'X', '-s', 'the', 'X', '.']
319.	give dog cat mouse , kiss guy dog <o> RAPO , OPA </o> ; to the mouse the dog that was kiss -ed by the guy give -s the cat . # ['to', 'the', 'X', 'the', 'X', 'that', 'was', 'X', '-ed', 'by', 'the', 'X', 'X', '-s', 'the', 'X', '.']
320.	give dog cat mouse , kiss guy cat <o> RAPO , OPA </o> ; to the mouse the dog give -s the cat that was kiss -ed by the guy . # ['to', 'the', 'X', 'the', 'X', 'X', '-s', 'the', 'X', 'that', 'was', 'X', '-ed', 'by', 'the', 'X', '.']
321.	give dog cat mouse , kiss mouse girl <o> RAOP , APO </o> ; to the mouse that kiss -s the girl by the dog the cat was give -ed . # ['to', 'the', 'X', 'that', 'X', '-s', 'the', 'X', 'by', 'the', 'X', 'the', 'X', 'was', 'X', '-ed', '.']
322.	give dog cat mouse , kiss guy mouse <o> RAOP , OAP </o> ; to the mouse that the guy kiss -s by the dog the cat was give -ed . # ['to', 'the', 'X', 'that', 'the', 'X', 'X', '-s', 'by', 'the', 'X', 'the', 'X', 'was', 'X', '-ed', '.']
323.	give dog cat mouse , kiss dog girl <o> RAOP , APO </o> ; to the mouse by the dog that kiss -s the girl the cat was give -ed . # ['to', 'the', 'X', 'by', 'the', 'X', 'that', 'X', '-s', 'the', 'X', 'the', 'X', 'was', 'X', '-ed', '.']
324.	give dog cat mouse , kiss guy dog <o> RAOP , OAP </o> ; to the mouse by the dog that the guy kiss -s the cat was give -ed . # ['to', 'the', 'X', 'by', 'the', 'X', 'that', 'the', 'X', 'X', '-s', 'the', 'X', 'was', 'X', '-ed', '.']
325.	give dog cat mouse , kiss cat girl <o> RAOP , APO </o> ; to the mouse by the dog the cat that kiss -s the girl was give -ed . # ['to', 'the', 'X', 'by', 'the', 'X', 'the', 'X', 'that', 'X', '-s', 'the', 'X', 'was', 'X', '-ed', '.']
326.	give dog cat mouse , kiss guy cat <o> RAOP , OAP </o> ; to the mouse by the dog the cat that the guy kiss -s was give -ed . # ['to', 'the', 'X', 'by', 'the', 'X', 'the', 'X', 'that', 'the', 'X', 'X', '-s', 'was', 'X', '-ed', '.']
327.	give dog cat mouse , kiss mouse girl <o> ROPA , APO </o> ; to the mouse that kiss -s the girl the cat was give -ed by the dog . # ['to', 'the', 'X', 'that', 'X', '-s', 'the', 'X', 'the', 'X', 'was', 'X', '-ed', 'by', 'the', 'X', '.']
328.	give dog cat mouse , kiss guy mouse <o> ROPA , OAP </o> ; to the mouse that the guy kiss -s the cat was give -ed by the dog . # ['to', 'the', 'X', 'that', 'the', 'X', 'X', '-s', 'the', 'X', 'was', 'X', '-ed', 'by', 'the', 'X', '.']
329.	give dog cat mouse , kiss cat girl <o> ROPA , APO </o> ; to the mouse the cat that kiss -s the girl was give -ed by the dog . # ['to', 'the', 'X', 'the', 'X', 'that', 'X', '-s', 'the', 'X', 'was', 'X', '-ed', 'by', 'the', 'X', '.']
330.	give dog cat mouse , kiss guy cat <o> ROPA , OAP </o> ; to the mouse the cat that the guy kiss -s was give -ed by the dog . # ['to', 'the', 'X', 'the', 'X', 'that', 'the', 'X', 'X', '-s', 'was', 'X', '-ed', 'by', 'the', 'X', '.']
331.	give dog cat mouse , kiss dog girl <o> ROPA , APO </o> ; to the mouse the cat was give -ed by the dog that kiss -s the girl . # ['to', 'the', 'X', 'the', 'X', 'was', 'X', '-ed', 'by', 'the', 'X', 'that', 'X', '-s', 'the', 'X', '.']
332.	give dog cat mouse , kiss guy dog <o> ROPA , OAP </o> ; to the mouse the cat was give -ed by the dog that the guy kiss -s . # ['to', 'the', 'X', 'the', 'X', 'was', 'X', '-ed', 'by', 'the', 'X', 'that', 'the', 'X', 'X', '-s', '.']
333.	give dog cat mouse , kiss mouse girl <o> ROAP , APO </o> ; to the mouse that kiss -s the girl the cat by the dog was give -ed . # ['to', 'the', 'X', 'that', 'X', '-s', 'the', 'X', 'the', 'X', 'by', 'the', 'X', 'was', 'X', '-ed', '.']
334.	give dog cat mouse , kiss guy mouse <o> ROAP , OAP </o> ; to the mouse that the guy kiss -s the cat by the dog was give -ed . # ['to', 'the', 'X', 'that', 'the', 'X', 'X', '-s', 'the', 'X', 'by', 'the', 'X', 'was', 'X', '-ed', '.']
335.	give dog cat mouse , kiss cat girl <o> ROAP , APO </o> ; to the mouse the cat that kiss -s the girl by the dog was give -ed . # ['to', 'the', 'X', 'the', 'X', 'that', 'X', '-s', 'the', 'X', 'by', 'the', 'X', 'was', 'X', '-ed', '.']
336.	give dog cat mouse , kiss guy cat <o> ROAP , OAP </o> ; to the mouse the cat that the guy kiss -s by the dog was give -ed . # ['to', 'the', 'X', 'the', 'X', 'that', 'the', 'X', 'X', '-s', 'by', 'the', 'X', 'was', 'X', '-ed', '.']
337.	give dog cat mouse , kiss dog girl <o> ROAP , APO </o> ; to the mouse the cat by the dog that kiss -s the girl was give -ed . # ['to', 'the', 'X', 'the', 'X', 'by', 'the', 'X', 'that', 'X', '-s', 'the', 'X', 'was', 'X', '-ed', '.']
338.	give dog cat mouse , kiss guy dog <o> ROAP , OAP </o> ; to the mouse the cat by the dog that the guy kiss -s was give -ed . # ['to', 'the', 'X', 'the', 'X', 'by', 'the', 'X', 'that', 'the', 'X', 'X', '-s', 'was', 'X', '-ed', '.']
339.	cut beaver fish , kiss beaver girl <o> PAO , AOP </o> ; cut -ed by the beaver that the girl was kiss -ed by was the fish . # ['X', '-ed', 'by', 'the', 'X', 'that', 'the', 'X', 'was', 'X', '-ed', 'by', 'was', 'the', 'X', '.']
340.	cut beaver fish , kiss fish girl <o> PAO , AOP </o> ; cut -ed by the beaver was the fish that the girl was kiss -ed by . # ['X', '-ed', 'by', 'the', 'X', 'was', 'the', 'X', 'that', 'the', 'X', 'was', 'X', '-ed', 'by', '.']
341.	cut beaver fish , kiss fish girl <o> OPA , AOP </o> ; the fish that the girl was kiss -ed by was cut -ed by the beaver . # ['the', 'X', 'that', 'the', 'X', 'was', 'X', '-ed', 'by', 'was', 'X', '-ed', 'by', 'the', 'X', '.']
342.	cut beaver fish , kiss beaver girl <o> OPA , AOP </o> ; the fish was cut -ed by the beaver that the girl was kiss -ed by . # ['the', 'X', 'was', 'X', '-ed', 'by', 'the', 'X', 'that', 'the', 'X', 'was', 'X', '-ed', 'by', '.']
343.	give dog cat mouse , kiss guy dog <o> PAOR , OPA </o> ; give -ed by the dog that was kiss -ed by the guy was the cat to the mouse . # ['X', '-ed', 'by', 'the', 'X', 'that', 'was', 'X', '-ed', 'by', 'the', 'X', 'was', 'the', 'X', 'to', 'the', 'X', '.']
344.	give dog cat mouse , kiss guy cat <o> PAOR , OPA </o> ; give -ed by the dog was the cat that was kiss -ed by the guy to the mouse . # ['X', '-ed', 'by', 'the', 'X', 'was', 'the', 'X', 'that', 'was', 'X', '-ed', 'by', 'the', 'X', 'to', 'the', 'X', '.']
345.	give dog cat mouse , kiss guy mouse <o> PAOR , OPA </o> ; give -ed by the dog was the cat to the mouse that was kiss -ed by the guy . # ['X', '-ed', 'by', 'the', 'X', 'was', 'the', 'X', 'to', 'the', 'X', 'that', 'was', 'X', '-ed', 'by', 'the', 'X', '.']
346.	give dog cat mouse , kiss guy dog <o> PARO , OPA </o> ; give -ed by the dog that was kiss -ed by the guy to the mouse was the cat . # ['X', '-ed', 'by', 'the', 'X', 'that', 'was', 'X', '-ed', 'by', 'the', 'X', 'to', 'the', 'X', 'was', 'the', 'X', '.']
347.	give dog cat mouse , kiss guy mouse <o> PARO , OPA </o> ; give -ed by the dog to the mouse that was kiss -ed by the guy was the cat . # ['X', '-ed', 'by', 'the', 'X', 'to', 'the', 'X', 'that', 'was', 'X', '-ed', 'by', 'the', 'X', 'was', 'the', 'X', '.']
348.	give dog cat mouse , kiss guy cat <o> PARO , OPA </o> ; give -ed by the dog to the mouse was the cat that was kiss -ed by the guy . # ['X', '-ed', 'by', 'the', 'X', 'to', 'the', 'X', 'was', 'the', 'X', 'that', 'was', 'X', '-ed', 'by', 'the', 'X', '.']
349.	give dog cat mouse , kiss guy cat <o> POAR , OPA </o> ; give -ed was the cat that was kiss -ed by the guy by the dog to the mouse . # ['X', '-ed', 'was', 'the', 'X', 'that', 'was', 'X', '-ed', 'by', 'the', 'X', 'by', 'the', 'X', 'to', 'the', 'X', '.']
350.	give dog cat mouse , kiss guy dog <o> POAR , OPA </o> ; give -ed was the cat by the dog that was kiss -ed by the guy to the mouse . # ['X', '-ed', 'was', 'the', 'X', 'by', 'the', 'X', 'that', 'was', 'X', '-ed', 'by', 'the', 'X', 'to', 'the', 'X', '.']
351.	give dog cat mouse , kiss guy mouse <o> POAR , OPA </o> ; give -ed was the cat by the dog to the mouse that was kiss -ed by the guy . # ['X', '-ed', 'was', 'the', 'X', 'by', 'the', 'X', 'to', 'the', 'X', 'that', 'was', 'X', '-ed', 'by', 'the', 'X', '.']
352.	give dog cat mouse , kiss cat girl <o> PORA , AOP </o> ; give the cat that the girl was kiss -ed by to the mouse , the dog did . # ['X', 'the', 'X', 'that', 'the', 'X', 'was', 'X', '-ed', 'by', 'to', 'the', 'X', ',', 'the', 'X', 'X', '.']
353.	give dog cat mouse , kiss mouse girl <o> PORA , AOP </o> ; give the cat to the mouse that the girl was kiss -ed by , the dog did . # ['X', 'the', 'X', 'to', 'the', 'X', 'that', 'the', 'X', 'was', 'X', '-ed', 'by', ',', 'the', 'X', 'X', '.']
354.	give dog cat mouse , kiss dog girl <o> PORA , AOP </o> ; give the cat to the mouse , the dog that the girl was kiss -ed by did . # ['X', 'the', 'X', 'to', 'the', 'X', ',', 'the', 'X', 'that', 'the', 'X', 'was', 'X', '-ed', 'by', 'X', '.']
355.	give dog cat mouse , kiss guy mouse <o> PRAO , OPA </o> ; give -ed to the mouse that was kiss -ed by the guy by the dog was the cat . # ['X', '-ed', 'to', 'the', 'X', 'that', 'was', 'X', '-ed', 'by', 'the', 'X', 'by', 'the', 'X', 'was', 'the', 'X', '.']
356.	give dog cat mouse , kiss guy dog <o> PRAO , OPA </o> ; give -ed to the mouse by the dog that was kiss -ed by the guy was the cat . # ['X', '-ed', 'to', 'the', 'X', 'by', 'the', 'X', 'that', 'was', 'X', '-ed', 'by', 'the', 'X', 'was', 'the', 'X', '.']
357.	give dog cat mouse , kiss guy cat <o> PRAO , OPA </o> ; give -ed to the mouse by the dog was the cat that was kiss -ed by the guy . # ['X', '-ed', 'to', 'the', 'X', 'by', 'the', 'X', 'was', 'the', 'X', 'that', 'was', 'X', '-ed', 'by', 'the', 'X', '.']
358.	give dog cat mouse , kiss guy mouse <o> PROA , OPA </o> ; give -ed to the mouse that was kiss -ed by the guy was the cat by the dog . # ['X', '-ed', 'to', 'the', 'X', 'that', 'was', 'X', '-ed', 'by', 'the', 'X', 'was', 'the', 'X', 'by', 'the', 'X', '.']
359.	give dog cat mouse , kiss guy cat <o> PROA , OPA </o> ; give -ed to the mouse was the cat that was kiss -ed by the guy by the dog . # ['X', '-ed', 'to', 'the', 'X', 'was', 'the', 'X', 'that', 'was', 'X', '-ed', 'by', 'the', 'X', 'by', 'the', 'X', '.']
360.	give dog cat mouse , kiss guy dog <o> PROA , OPA </o> ; give -ed to the mouse was the cat by the dog that was kiss -ed by the guy . # ['X', '-ed', 'to', 'the', 'X', 'was', 'the', 'X', 'by', 'the', 'X', 'that', 'was', 'X', '-ed', 'by', 'the', 'X', '.']
361.	give dog cat mouse , kiss guy cat <o> OPAR , OPA </o> ; the cat that was kiss -ed by the guy was give -ed by the dog to the mouse . # ['the', 'X', 'that', 'was', 'X', '-ed', 'by', 'the', 'X', 'was', 'X', '-ed', 'by', 'the', 'X', 'to', 'the', 'X', '.']
362.	give dog cat mouse , kiss guy dog <o> OPAR , OPA </o> ; the cat was give -ed by the dog that was kiss -ed by the guy to the mouse . # ['the', 'X', 'was', 'X', '-ed', 'by', 'the', 'X', 'that', 'was', 'X', '-ed', 'by', 'the', 'X', 'to', 'the', 'X', '.']
363.	give dog cat mouse , kiss guy mouse <o> OPAR , OPA </o> ; the cat was give -ed by the dog to the mouse that was kiss -ed by the guy . # ['the', 'X', 'was', 'X', '-ed', 'by', 'the', 'X', 'to', 'the', 'X', 'that', 'was', 'X', '-ed', 'by', 'the', 'X', '.']
364.	give dog cat mouse , kiss guy cat <o> OPRA , OPA </o> ; the cat that was kiss -ed by the guy was give -ed to the mouse by the dog . # ['the', 'X', 'that', 'was', 'X', '-ed', 'by', 'the', 'X', 'was', 'X', '-ed', 'to', 'the', 'X', 'by', 'the', 'X', '.']
365.	give dog cat mouse , kiss guy mouse <o> OPRA , OPA </o> ; the cat was give -ed to the mouse that was kiss -ed by the guy by the dog . # ['the', 'X', 'was', 'X', '-ed', 'to', 'the', 'X', 'that', 'was', 'X', '-ed', 'by', 'the', 'X', 'by', 'the', 'X', '.']
366.	give dog cat mouse , kiss guy dog <o> OPRA , OPA </o> ; the cat was give -ed to the mouse by the dog that was kiss -ed by the guy . # ['the', 'X', 'was', 'X', '-ed', 'to', 'the', 'X', 'by', 'the', 'X', 'that', 'was', 'X', '-ed', 'by', 'the', 'X', '.']
367.	give dog cat mouse , kiss guy cat <o> OAPR , OPA </o> ; the cat that was kiss -ed by the guy by the dog was give -ed to the mouse . # ['the', 'X', 'that', 'was', 'X', '-ed', 'by', 'the', 'X', 'by', 'the', 'X', 'was', 'X', '-ed', 'to', 'the', 'X', '.']
368.	give dog cat mouse , kiss guy dog <o> OAPR , OPA </o> ; the cat by the dog that was kiss -ed by the guy was give -ed to the mouse . # ['the', 'X', 'by', 'the', 'X', 'that', 'was', 'X', '-ed', 'by', 'the', 'X', 'was', 'X', '-ed', 'to', 'the', 'X', '.']
369.	give dog cat mouse , kiss guy mouse <o> OAPR , OPA </o> ; the cat by the dog was give -ed to the mouse that was kiss -ed by the guy . # ['the', 'X', 'by', 'the', 'X', 'was', 'X', '-ed', 'to', 'the', 'X', 'that', 'was', 'X', '-ed', 'by', 'the', 'X', '.']
370.	give dog cat mouse , kiss guy cat <o> OARP , OPA </o> ; the cat that was kiss -ed by the guy by the dog to the mouse was give -ed . # ['the', 'X', 'that', 'was', 'X', '-ed', 'by', 'the', 'X', 'by', 'the', 'X', 'to', 'the', 'X', 'was', 'X', '-ed', '.']
371.	give dog cat mouse , kiss guy dog <o> OARP , OPA </o> ; the cat by the dog that was kiss -ed by the guy to the mouse was give -ed . # ['the', 'X', 'by', 'the', 'X', 'that', 'was', 'X', '-ed', 'by', 'the', 'X', 'to', 'the', 'X', 'was', 'X', '-ed', '.']
372.	give dog cat mouse , kiss guy mouse <o> OARP , OPA </o> ; the cat by the dog to the mouse that was kiss -ed by the guy was give -ed . # ['the', 'X', 'by', 'the', 'X', 'to', 'the', 'X', 'that', 'was', 'X', '-ed', 'by', 'the', 'X', 'was', 'X', '-ed', '.']
373.	give dog cat mouse , kiss guy cat <o> ORPA , OPA </o> ; the cat that was kiss -ed by the guy to the mouse was give -ed by the dog . # ['the', 'X', 'that', 'was', 'X', '-ed', 'by', 'the', 'X', 'to', 'the', 'X', 'was', 'X', '-ed', 'by', 'the', 'X', '.']
374.	give dog cat mouse , kiss guy mouse <o> ORPA , OPA </o> ; the cat to the mouse that was kiss -ed by the guy was give -ed by the dog . # ['the', 'X', 'to', 'the', 'X', 'that', 'was', 'X', '-ed', 'by', 'the', 'X', 'was', 'X', '-ed', 'by', 'the', 'X', '.']
375.	give dog cat mouse , kiss guy dog <o> ORPA , OPA </o> ; the cat to the mouse was give -ed by the dog that was kiss -ed by the guy . # ['the', 'X', 'to', 'the', 'X', 'was', 'X', '-ed', 'by', 'the', 'X', 'that', 'was', 'X', '-ed', 'by', 'the', 'X', '.']
376.	give dog cat mouse , kiss guy cat <o> ORAP , OPA </o> ; the cat that was kiss -ed by the guy to the mouse by the dog was give -ed . # ['the', 'X', 'that', 'was', 'X', '-ed', 'by', 'the', 'X', 'to', 'the', 'X', 'by', 'the', 'X', 'was', 'X', '-ed', '.']
377.	give dog cat mouse , kiss guy mouse <o> ORAP , OPA </o> ; the cat to the mouse that was kiss -ed by the guy by the dog was give -ed . # ['the', 'X', 'to', 'the', 'X', 'that', 'was', 'X', '-ed', 'by', 'the', 'X', 'by', 'the', 'X', 'was', 'X', '-ed', '.']
378.	give dog cat mouse , kiss guy dog <o> ORAP , OPA </o> ; the cat to the mouse by the dog that was kiss -ed by the guy was give -ed . # ['the', 'X', 'to', 'the', 'X', 'by', 'the', 'X', 'that', 'was', 'X', '-ed', 'by', 'the', 'X', 'was', 'X', '-ed', '.']
379.	give dog cat mouse , kiss mouse girl <o> RAPO , AOP </o> ; to the mouse that the girl was kiss -ed by the dog give -s the cat . # ['to', 'the', 'X', 'that', 'the', 'X', 'was', 'X', '-ed', 'by', 'the', 'X', 'X', '-s', 'the', 'X', '.']
380.	give dog cat mouse , kiss dog girl <o> RAPO , AOP </o> ; to the mouse the dog that the girl was kiss -ed by give -s the cat . # ['to', 'the', 'X', 'the', 'X', 'that', 'the', 'X', 'was', 'X', '-ed', 'by', 'X', '-s', 'the', 'X', '.']
381.	give dog cat mouse , kiss cat girl <o> RAPO , AOP </o> ; to the mouse the dog give -s the cat that the girl was kiss -ed by . # ['to', 'the', 'X', 'the', 'X', 'X', '-s', 'the', 'X', 'that', 'the', 'X', 'was', 'X', '-ed', 'by', '.']
382.	give dog cat mouse , kiss dog girl <o> PAOR , AOP </o> ; give -ed by the dog that the girl was kiss -ed by was the cat to the mouse . # ['X', '-ed', 'by', 'the', 'X', 'that', 'the', 'X', 'was', 'X', '-ed', 'by', 'was', 'the', 'X', 'to', 'the', 'X', '.']
383.	give dog cat mouse , kiss cat girl <o> PAOR , AOP </o> ; give -ed by the dog was the cat that the girl was kiss -ed by to the mouse . # ['X', '-ed', 'by', 'the', 'X', 'was', 'the', 'X', 'that', 'the', 'X', 'was', 'X', '-ed', 'by', 'to', 'the', 'X', '.']
384.	give dog cat mouse , kiss mouse girl <o> PAOR , AOP </o> ; give -ed by the dog was the cat to the mouse that the girl was kiss -ed by . # ['X', '-ed', 'by', 'the', 'X', 'was', 'the', 'X', 'to', 'the', 'X', 'that', 'the', 'X', 'was', 'X', '-ed', 'by', '.']
385.	give dog cat mouse , kiss dog girl <o> PARO , AOP </o> ; give -ed by the dog that the girl was kiss -ed by to the mouse was the cat . # ['X', '-ed', 'by', 'the', 'X', 'that', 'the', 'X', 'was', 'X', '-ed', 'by', 'to', 'the', 'X', 'was', 'the', 'X', '.']
386.	give dog cat mouse , kiss mouse girl <o> PARO , AOP </o> ; give -ed by the dog to the mouse that the girl was kiss -ed by was the cat . # ['X', '-ed', 'by', 'the', 'X', 'to', 'the', 'X', 'that', 'the', 'X', 'was', 'X', '-ed', 'by', 'was', 'the', 'X', '.']
387.	give dog cat mouse , kiss cat girl <o> PARO , AOP </o> ; give -ed by the dog to the mouse was the cat that the girl was kiss -ed by . # ['X', '-ed', 'by', 'the', 'X', 'to', 'the', 'X', 'was', 'the', 'X', 'that', 'the', 'X', 'was', 'X', '-ed', 'by', '.']
388.	give dog cat mouse , kiss cat girl <o> POAR , AOP </o> ; give -ed was the cat that the girl was kiss -ed by by the dog to the mouse . # ['X', '-ed', 'was', 'the', 'X', 'that', 'the', 'X', 'was', 'X', '-ed', 'by', 'by', 'the', 'X', 'to', 'the', 'X', '.']
389.	give dog cat mouse , kiss dog girl <o> POAR , AOP </o> ; give -ed was the cat by the dog that the girl was kiss -ed by to the mouse . # ['X', '-ed', 'was', 'the', 'X', 'by', 'the', 'X', 'that', 'the', 'X', 'was', 'X', '-ed', 'by', 'to', 'the', 'X', '.']
390.	give dog cat mouse , kiss mouse girl <o> POAR , AOP </o> ; give -ed was the cat by the dog to the mouse that the girl was kiss -ed by . # ['X', '-ed', 'was', 'the', 'X', 'by', 'the', 'X', 'to', 'the', 'X', 'that', 'the', 'X', 'was', 'X', '-ed', 'by', '.']
391.	give dog cat mouse , kiss mouse girl <o> PRAO , AOP </o> ; give -ed to the mouse that the girl was kiss -ed by by the dog was the cat . # ['X', '-ed', 'to', 'the', 'X', 'that', 'the', 'X', 'was', 'X', '-ed', 'by', 'by', 'the', 'X', 'was', 'the', 'X', '.']
392.	give dog cat mouse , kiss dog girl <o> PRAO , AOP </o> ; give -ed to the mouse by the dog that the girl was kiss -ed by was the cat . # ['X', '-ed', 'to', 'the', 'X', 'by', 'the', 'X', 'that', 'the', 'X', 'was', 'X', '-ed', 'by', 'was', 'the', 'X', '.']
393.	give dog cat mouse , kiss cat girl <o> PRAO , AOP </o> ; give -ed to the mouse by the dog was the cat that the girl was kiss -ed by . # ['X', '-ed', 'to', 'the', 'X', 'by', 'the', 'X', 'was', 'the', 'X', 'that', 'the', 'X', 'was', 'X', '-ed', 'by', '.']
394.	give dog cat mouse , kiss mouse girl <o> PROA , AOP </o> ; give -ed to the mouse that the girl was kiss -ed by was the cat by the dog . # ['X', '-ed', 'to', 'the', 'X', 'that', 'the', 'X', 'was', 'X', '-ed', 'by', 'was', 'the', 'X', 'by', 'the', 'X', '.']
395.	give dog cat mouse , kiss cat girl <o> PROA , AOP </o> ; give -ed to the mouse was the cat that the girl was kiss -ed by by the dog . # ['X', '-ed', 'to', 'the', 'X', 'was', 'the', 'X', 'that', 'the', 'X', 'was', 'X', '-ed', 'by', 'by', 'the', 'X', '.']
396.	give dog cat mouse , kiss dog girl <o> PROA , AOP </o> ; give -ed to the mouse was the cat by the dog that the girl was kiss -ed by . # ['X', '-ed', 'to', 'the', 'X', 'was', 'the', 'X', 'by', 'the', 'X', 'that', 'the', 'X', 'was', 'X', '-ed', 'by', '.']
397.	give dog cat mouse , kiss guy dog <o> AOPR , OPA </o> ; by the dog that was kiss -ed by the guy the cat was give -ed to the mouse . # ['by', 'the', 'X', 'that', 'was', 'X', '-ed', 'by', 'the', 'X', 'the', 'X', 'was', 'X', '-ed', 'to', 'the', 'X', '.']
398.	give dog cat mouse , kiss guy cat <o> AOPR , OPA </o> ; by the dog the cat that was kiss -ed by the guy was give -ed to the mouse . # ['by', 'the', 'X', 'the', 'X', 'that', 'was', 'X', '-ed', 'by', 'the', 'X', 'was', 'X', '-ed', 'to', 'the', 'X', '.']
399.	give dog cat mouse , kiss guy mouse <o> AOPR , OPA </o> ; by the dog the cat was give -ed to the mouse that was kiss -ed by the guy . # ['by', 'the', 'X', 'the', 'X', 'was', 'X', '-ed', 'to', 'the', 'X', 'that', 'was', 'X', '-ed', 'by', 'the', 'X', '.']
400.	give dog cat mouse , kiss guy dog <o> AORP , OPA </o> ; by the dog that was kiss -ed by the guy the cat to the mouse was give -ed . # ['by', 'the', 'X', 'that', 'was', 'X', '-ed', 'by', 'the', 'X', 'the', 'X', 'to', 'the', 'X', 'was', 'X', '-ed', '.']
401.	give dog cat mouse , kiss guy cat <o> AORP , OPA </o> ; by the dog the cat that was kiss -ed by the guy to the mouse was give -ed . # ['by', 'the', 'X', 'the', 'X', 'that', 'was', 'X', '-ed', 'by', 'the', 'X', 'to', 'the', 'X', 'was', 'X', '-ed', '.']
402.	give dog cat mouse , kiss guy mouse <o> AORP , OPA </o> ; by the dog the cat to the mouse that was kiss -ed by the guy was give -ed . # ['by', 'the', 'X', 'the', 'X', 'to', 'the', 'X', 'that', 'was', 'X', '-ed', 'by', 'the', 'X', 'was', 'X', '-ed', '.']
403.	give dog cat mouse , kiss guy dog <o> AROP , OPA </o> ; by the dog that was kiss -ed by the guy to the mouse the cat was give -ed . # ['by', 'the', 'X', 'that', 'was', 'X', '-ed', 'by', 'the', 'X', 'to', 'the', 'X', 'the', 'X', 'was', 'X', '-ed', '.']
404.	give dog cat mouse , kiss guy mouse <o> AROP , OPA </o> ; by the dog to the mouse that was kiss -ed by the guy the cat was give -ed . # ['by', 'the', 'X', 'to', 'the', 'X', 'that', 'was', 'X', '-ed', 'by', 'the', 'X', 'the', 'X', 'was', 'X', '-ed', '.']
405.	give dog cat mouse , kiss guy cat <o> AROP , OPA </o> ; by the dog to the mouse the cat that was kiss -ed by the guy was give -ed . # ['by', 'the', 'X', 'to', 'the', 'X', 'the', 'X', 'that', 'was', 'X', '-ed', 'by', 'the', 'X', 'was', 'X', '-ed', '.']
406.	give dog cat mouse , kiss cat girl <o> OPAR , AOP </o> ; the cat that the girl was kiss -ed by was give -ed by the dog to the mouse . # ['the', 'X', 'that', 'the', 'X', 'was', 'X', '-ed', 'by', 'was', 'X', '-ed', 'by', 'the', 'X', 'to', 'the', 'X', '.']
407.	give dog cat mouse , kiss dog girl <o> OPAR , AOP </o> ; the cat was give -ed by the dog that the girl was kiss -ed by to the mouse . # ['the', 'X', 'was', 'X', '-ed', 'by', 'the', 'X', 'that', 'the', 'X', 'was', 'X', '-ed', 'by', 'to', 'the', 'X', '.']
408.	give dog cat mouse , kiss mouse girl <o> OPAR , AOP </o> ; the cat was give -ed by the dog to the mouse that the girl was kiss -ed by . # ['the', 'X', 'was', 'X', '-ed', 'by', 'the', 'X', 'to', 'the', 'X', 'that', 'the', 'X', 'was', 'X', '-ed', 'by', '.']
409.	give dog cat mouse , kiss cat girl <o> OPRA , AOP </o> ; the cat that the girl was kiss -ed by was give -ed to the mouse by the dog . # ['the', 'X', 'that', 'the', 'X', 'was', 'X', '-ed', 'by', 'was', 'X', '-ed', 'to', 'the', 'X', 'by', 'the', 'X', '.']
410.	give dog cat mouse , kiss mouse girl <o> OPRA , AOP </o> ; the cat was give -ed to the mouse that the girl was kiss -ed by by the dog . # ['the', 'X', 'was', 'X', '-ed', 'to', 'the', 'X', 'that', 'the', 'X', 'was', 'X', '-ed', 'by', 'by', 'the', 'X', '.']
411.	give dog cat mouse , kiss dog girl <o> OPRA , AOP </o> ; the cat was give -ed to the mouse by the dog that the girl was kiss -ed by . # ['the', 'X', 'was', 'X', '-ed', 'to', 'the', 'X', 'by', 'the', 'X', 'that', 'the', 'X', 'was', 'X', '-ed', 'by', '.']
412.	give dog cat mouse , kiss cat girl <o> OAPR , AOP </o> ; the cat that the girl was kiss -ed by by the dog was give -ed to the mouse . # ['the', 'X', 'that', 'the', 'X', 'was', 'X', '-ed', 'by', 'by', 'the', 'X', 'was', 'X', '-ed', 'to', 'the', 'X', '.']
413.	give dog cat mouse , kiss dog girl <o> OAPR , AOP </o> ; the cat by the dog that the girl was kiss -ed by was give -ed to the mouse . # ['the', 'X', 'by', 'the', 'X', 'that', 'the', 'X', 'was', 'X', '-ed', 'by', 'was', 'X', '-ed', 'to', 'the', 'X', '.']
414.	give dog cat mouse , kiss mouse girl <o> OAPR , AOP </o> ; the cat by the dog was give -ed to the mouse that the girl was kiss -ed by . # ['the', 'X', 'by', 'the', 'X', 'was', 'X', '-ed', 'to', 'the', 'X', 'that', 'the', 'X', 'was', 'X', '-ed', 'by', '.']
415.	give dog cat mouse , kiss cat girl <o> OARP , AOP </o> ; the cat that the girl was kiss -ed by by the dog to the mouse was give -ed . # ['the', 'X', 'that', 'the', 'X', 'was', 'X', '-ed', 'by', 'by', 'the', 'X', 'to', 'the', 'X', 'was', 'X', '-ed', '.']
416.	give dog cat mouse , kiss dog girl <o> OARP , AOP </o> ; the cat by the dog that the girl was kiss -ed by to the mouse was give -ed . # ['the', 'X', 'by', 'the', 'X', 'that', 'the', 'X', 'was', 'X', '-ed', 'by', 'to', 'the', 'X', 'was', 'X', '-ed', '.']
417.	give dog cat mouse , kiss mouse girl <o> OARP , AOP </o> ; the cat by the dog to the mouse that the girl was kiss -ed by was give -ed . # ['the', 'X', 'by', 'the', 'X', 'to', 'the', 'X', 'that', 'the', 'X', 'was', 'X', '-ed', 'by', 'was', 'X', '-ed', '.']
418.	give dog cat mouse , kiss cat girl <o> ORPA , AOP </o> ; the cat that the girl was kiss -ed by to the mouse was give -ed by the dog . # ['the', 'X', 'that', 'the', 'X', 'was', 'X', '-ed', 'by', 'to', 'the', 'X', 'was', 'X', '-ed', 'by', 'the', 'X', '.']
419.	give dog cat mouse , kiss mouse girl <o> ORPA , AOP </o> ; the cat to the mouse that the girl was kiss -ed by was give -ed by the dog . # ['the', 'X', 'to', 'the', 'X', 'that', 'the', 'X', 'was', 'X', '-ed', 'by', 'was', 'X', '-ed', 'by', 'the', 'X', '.']
420.	give dog cat mouse , kiss dog girl <o> ORPA , AOP </o> ; the cat to the mouse was give -ed by the dog that the girl was kiss -ed by . # ['the', 'X', 'to', 'the', 'X', 'was', 'X', '-ed', 'by', 'the', 'X', 'that', 'the', 'X', 'was', 'X', '-ed', 'by', '.']
421.	give dog cat mouse , kiss cat girl <o> ORAP , AOP </o> ; the cat that the girl was kiss -ed by to the mouse by the dog was give -ed . # ['the', 'X', 'that', 'the', 'X', 'was', 'X', '-ed', 'by', 'to', 'the', 'X', 'by', 'the', 'X', 'was', 'X', '-ed', '.']
422.	give dog cat mouse , kiss mouse girl <o> ORAP , AOP </o> ; the cat to the mouse that the girl was kiss -ed by by the dog was give -ed . # ['the', 'X', 'to', 'the', 'X', 'that', 'the', 'X', 'was', 'X', '-ed', 'by', 'by', 'the', 'X', 'was', 'X', '-ed', '.']
423.	give dog cat mouse , kiss dog girl <o> ORAP , AOP </o> ; the cat to the mouse by the dog that the girl was kiss -ed by was give -ed . # ['the', 'X', 'to', 'the', 'X', 'by', 'the', 'X', 'that', 'the', 'X', 'was', 'X', '-ed', 'by', 'was', 'X', '-ed', '.']
424.	give dog cat mouse , kiss guy mouse <o> RPAO , OPA </o> ; to the mouse that was kiss -ed by the guy was give -ed by the dog the cat . # ['to', 'the', 'X', 'that', 'was', 'X', '-ed', 'by', 'the', 'X', 'was', 'X', '-ed', 'by', 'the', 'X', 'the', 'X', '.']
425.	give dog cat mouse , kiss guy dog <o> RPAO , OPA </o> ; to the mouse was give -ed by the dog that was kiss -ed by the guy the cat . # ['to', 'the', 'X', 'was', 'X', '-ed', 'by', 'the', 'X', 'that', 'was', 'X', '-ed', 'by', 'the', 'X', 'the', 'X', '.']
426.	give dog cat mouse , kiss guy cat <o> RPAO , OPA </o> ; to the mouse was give -ed by the dog the cat that was kiss -ed by the guy . # ['to', 'the', 'X', 'was', 'X', '-ed', 'by', 'the', 'X', 'the', 'X', 'that', 'was', 'X', '-ed', 'by', 'the', 'X', '.']
427.	give dog cat mouse , kiss guy mouse <o> RPOA , OPA </o> ; to the mouse that was kiss -ed by the guy was give -ed the cat by the dog . # ['to', 'the', 'X', 'that', 'was', 'X', '-ed', 'by', 'the', 'X', 'was', 'X', '-ed', 'the', 'X', 'by', 'the', 'X', '.']
428.	give dog cat mouse , kiss guy cat <o> RPOA , OPA </o> ; to the mouse was give -ed the cat that was kiss -ed by the guy by the dog . # ['to', 'the', 'X', 'was', 'X', '-ed', 'the', 'X', 'that', 'was', 'X', '-ed', 'by', 'the', 'X', 'by', 'the', 'X', '.']
429.	give dog cat mouse , kiss guy dog <o> RPOA , OPA </o> ; to the mouse was give -ed the cat by the dog that was kiss -ed by the guy . # ['to', 'the', 'X', 'was', 'X', '-ed', 'the', 'X', 'by', 'the', 'X', 'that', 'was', 'X', '-ed', 'by', 'the', 'X', '.']
430.	give dog cat mouse , kiss guy mouse <o> RAOP , OPA </o> ; to the mouse that was kiss -ed by the guy by the dog the cat was give -ed . # ['to', 'the', 'X', 'that', 'was', 'X', '-ed', 'by', 'the', 'X', 'by', 'the', 'X', 'the', 'X', 'was', 'X', '-ed', '.']
431.	give dog cat mouse , kiss guy dog <o> RAOP , OPA </o> ; to the mouse by the dog that was kiss -ed by the guy the cat was give -ed . # ['to', 'the', 'X', 'by', 'the', 'X', 'that', 'was', 'X', '-ed', 'by', 'the', 'X', 'the', 'X', 'was', 'X', '-ed', '.']
432.	give dog cat mouse , kiss guy cat <o> RAOP , OPA </o> ; to the mouse by the dog the cat that was kiss -ed by the guy was give -ed . # ['to', 'the', 'X', 'by', 'the', 'X', 'the', 'X', 'that', 'was', 'X', '-ed', 'by', 'the', 'X', 'was', 'X', '-ed', '.']
433.	give dog cat mouse , kiss guy mouse <o> ROPA , OPA </o> ; to the mouse that was kiss -ed by the guy the cat was give -ed by the dog . # ['to', 'the', 'X', 'that', 'was', 'X', '-ed', 'by', 'the', 'X', 'the', 'X', 'was', 'X', '-ed', 'by', 'the', 'X', '.']
434.	give dog cat mouse , kiss guy cat <o> ROPA , OPA </o> ; to the mouse the cat that was kiss -ed by the guy was give -ed by the dog . # ['to', 'the', 'X', 'the', 'X', 'that', 'was', 'X', '-ed', 'by', 'the', 'X', 'was', 'X', '-ed', 'by', 'the', 'X', '.']
435.	give dog cat mouse , kiss guy dog <o> ROPA , OPA </o> ; to the mouse the cat was give -ed by the dog that was kiss -ed by the guy . # ['to', 'the', 'X', 'the', 'X', 'was', 'X', '-ed', 'by', 'the', 'X', 'that', 'was', 'X', '-ed', 'by', 'the', 'X', '.']
436.	give dog cat mouse , kiss guy mouse <o> ROAP , OPA </o> ; to the mouse that was kiss -ed by the guy the cat by the dog was give -ed . # ['to', 'the', 'X', 'that', 'was', 'X', '-ed', 'by', 'the', 'X', 'the', 'X', 'by', 'the', 'X', 'was', 'X', '-ed', '.']
437.	give dog cat mouse , kiss guy cat <o> ROAP , OPA </o> ; to the mouse the cat that was kiss -ed by the guy by the dog was give -ed . # ['to', 'the', 'X', 'the', 'X', 'that', 'was', 'X', '-ed', 'by', 'the', 'X', 'by', 'the', 'X', 'was', 'X', '-ed', '.']
438.	give dog cat mouse , kiss guy dog <o> ROAP , OPA </o> ; to the mouse the cat by the dog that was kiss -ed by the guy was give -ed . # ['to', 'the', 'X', 'the', 'X', 'by', 'the', 'X', 'that', 'was', 'X', '-ed', 'by', 'the', 'X', 'was', 'X', '-ed', '.']
439.	give dog cat mouse , kiss dog girl <o> AOPR , AOP </o> ; by the dog that the girl was kiss -ed by the cat was give -ed to the mouse . # ['by', 'the', 'X', 'that', 'the', 'X', 'was', 'X', '-ed', 'by', 'the', 'X', 'was', 'X', '-ed', 'to', 'the', 'X', '.']
440.	give dog cat mouse , kiss cat girl <o> AOPR , AOP </o> ; by the dog the cat that the girl was kiss -ed by was give -ed to the mouse . # ['by', 'the', 'X', 'the', 'X', 'that', 'the', 'X', 'was', 'X', '-ed', 'by', 'was', 'X', '-ed', 'to', 'the', 'X', '.']
441.	give dog cat mouse , kiss mouse girl <o> AOPR , AOP </o> ; by the dog the cat was give -ed to the mouse that the girl was kiss -ed by . # ['by', 'the', 'X', 'the', 'X', 'was', 'X', '-ed', 'to', 'the', 'X', 'that', 'the', 'X', 'was', 'X', '-ed', 'by', '.']
442.	give dog cat mouse , kiss dog girl <o> AORP , AOP </o> ; by the dog that the girl was kiss -ed by the cat to the mouse was give -ed . # ['by', 'the', 'X', 'that', 'the', 'X', 'was', 'X', '-ed', 'by', 'the', 'X', 'to', 'the', 'X', 'was', 'X', '-ed', '.']
443.	give dog cat mouse , kiss cat girl <o> AORP , AOP </o> ; by the dog the cat that the girl was kiss -ed by to the mouse was give -ed . # ['by', 'the', 'X', 'the', 'X', 'that', 'the', 'X', 'was', 'X', '-ed', 'by', 'to', 'the', 'X', 'was', 'X', '-ed', '.']
444.	give dog cat mouse , kiss mouse girl <o> AORP , AOP </o> ; by the dog the cat to the mouse that the girl was kiss -ed by was give -ed . # ['by', 'the', 'X', 'the', 'X', 'to', 'the', 'X', 'that', 'the', 'X', 'was', 'X', '-ed', 'by', 'was', 'X', '-ed', '.']
445.	give dog cat mouse , kiss dog girl <o> AROP , AOP </o> ; by the dog that the girl was kiss -ed by to the mouse the cat was give -ed . # ['by', 'the', 'X', 'that', 'the', 'X', 'was', 'X', '-ed', 'by', 'to', 'the', 'X', 'the', 'X', 'was', 'X', '-ed', '.']
446.	give dog cat mouse , kiss mouse girl <o> AROP , AOP </o> ; by the dog to the mouse that the girl was kiss -ed by the cat was give -ed . # ['by', 'the', 'X', 'to', 'the', 'X', 'that', 'the', 'X', 'was', 'X', '-ed', 'by', 'the', 'X', 'was', 'X', '-ed', '.']
447.	give dog cat mouse , kiss cat girl <o> AROP , AOP </o> ; by the dog to the mouse the cat that the girl was kiss -ed by was give -ed . # ['by', 'the', 'X', 'to', 'the', 'X', 'the', 'X', 'that', 'the', 'X', 'was', 'X', '-ed', 'by', 'was', 'X', '-ed', '.']
448.	give dog cat mouse , kiss mouse girl <o> RPAO , AOP </o> ; to the mouse that the girl was kiss -ed by was give -ed by the dog the cat . # ['to', 'the', 'X', 'that', 'the', 'X', 'was', 'X', '-ed', 'by', 'was', 'X', '-ed', 'by', 'the', 'X', 'the', 'X', '.']
449.	give dog cat mouse , kiss dog girl <o> RPAO , AOP </o> ; to the mouse was give -ed by the dog that the girl was kiss -ed by the cat . # ['to', 'the', 'X', 'was', 'X', '-ed', 'by', 'the', 'X', 'that', 'the', 'X', 'was', 'X', '-ed', 'by', 'the', 'X', '.']
450.	give dog cat mouse , kiss cat girl <o> RPAO , AOP </o> ; to the mouse was give -ed by the dog the cat that the girl was kiss -ed by . # ['to', 'the', 'X', 'was', 'X', '-ed', 'by', 'the', 'X', 'the', 'X', 'that', 'the', 'X', 'was', 'X', '-ed', 'by', '.']
451.	give dog cat mouse , kiss mouse girl <o> RPOA , AOP </o> ; to the mouse that the girl was kiss -ed by was give -ed the cat by the dog . # ['to', 'the', 'X', 'that', 'the', 'X', 'was', 'X', '-ed', 'by', 'was', 'X', '-ed', 'the', 'X', 'by', 'the', 'X', '.']
452.	give dog cat mouse , kiss cat girl <o> RPOA , AOP </o> ; to the mouse was give -ed the cat that the girl was kiss -ed by by the dog . # ['to', 'the', 'X', 'was', 'X', '-ed', 'the', 'X', 'that', 'the', 'X', 'was', 'X', '-ed', 'by', 'by', 'the', 'X', '.']
453.	give dog cat mouse , kiss dog girl <o> RPOA , AOP </o> ; to the mouse was give -ed the cat by the dog that the girl was kiss -ed by . # ['to', 'the', 'X', 'was', 'X', '-ed', 'the', 'X', 'by', 'the', 'X', 'that', 'the', 'X', 'was', 'X', '-ed', 'by', '.']
454.	give dog cat mouse , kiss mouse girl <o> RAOP , AOP </o> ; to the mouse that the girl was kiss -ed by by the dog the cat was give -ed . # ['to', 'the', 'X', 'that', 'the', 'X', 'was', 'X', '-ed', 'by', 'by', 'the', 'X', 'the', 'X', 'was', 'X', '-ed', '.']
455.	give dog cat mouse , kiss dog girl <o> RAOP , AOP </o> ; to the mouse by the dog that the girl was kiss -ed by the cat was give -ed . # ['to', 'the', 'X', 'by', 'the', 'X', 'that', 'the', 'X', 'was', 'X', '-ed', 'by', 'the', 'X', 'was', 'X', '-ed', '.']
456.	give dog cat mouse , kiss cat girl <o> RAOP , AOP </o> ; to the mouse by the dog the cat that the girl was kiss -ed by was give -ed . # ['to', 'the', 'X', 'by', 'the', 'X', 'the', 'X', 'that', 'the', 'X', 'was', 'X', '-ed', 'by', 'was', 'X', '-ed', '.']
457.	give dog cat mouse , kiss mouse girl <o> ROPA , AOP </o> ; to the mouse that the girl was kiss -ed by the cat was give -ed by the dog . # ['to', 'the', 'X', 'that', 'the', 'X', 'was', 'X', '-ed', 'by', 'the', 'X', 'was', 'X', '-ed', 'by', 'the', 'X', '.']
458.	give dog cat mouse , kiss cat girl <o> ROPA , AOP </o> ; to the mouse the cat that the girl was kiss -ed by was give -ed by the dog . # ['to', 'the', 'X', 'the', 'X', 'that', 'the', 'X', 'was', 'X', '-ed', 'by', 'was', 'X', '-ed', 'by', 'the', 'X', '.']
459.	give dog cat mouse , kiss dog girl <o> ROPA , AOP </o> ; to the mouse the cat was give -ed by the dog that the girl was kiss -ed by . # ['to', 'the', 'X', 'the', 'X', 'was', 'X', '-ed', 'by', 'the', 'X', 'that', 'the', 'X', 'was', 'X', '-ed', 'by', '.']
460.	give dog cat mouse , kiss mouse girl <o> ROAP , AOP </o> ; to the mouse that the girl was kiss -ed by the cat by the dog was give -ed . # ['to', 'the', 'X', 'that', 'the', 'X', 'was', 'X', '-ed', 'by', 'the', 'X', 'by', 'the', 'X', 'was', 'X', '-ed', '.']
461.	give dog cat mouse , kiss cat girl <o> ROAP , AOP </o> ; to the mouse the cat that the girl was kiss -ed by by the dog was give -ed . # ['to', 'the', 'X', 'the', 'X', 'that', 'the', 'X', 'was', 'X', '-ed', 'by', 'by', 'the', 'X', 'was', 'X', '-ed', '.']
462.	give dog cat mouse , kiss dog girl <o> ROAP , AOP </o> ; to the mouse the cat by the dog that the girl was kiss -ed by was give -ed . # ['to', 'the', 'X', 'the', 'X', 'by', 'the', 'X', 'that', 'the', 'X', 'was', 'X', '-ed', 'by', 'was', 'X', '-ed', '.']
</train data>
